# Epigenome-wide association study for atrazine induced transgenerational DNA methylation and histone retention sperm epigenetic biomarkers for disease

**DOI:** 10.1371/journal.pone.0239380

**Published:** 2020-12-16

**Authors:** Jennifer L. M. Thorson, Daniel Beck, Millissia Ben Maamar, Eric E. Nilsson, Margaux McBirney, Michael K. Skinner

**Affiliations:** Center for Reproductive Biology, School of Biological Sciences, Washington State University, Pullman, Washington, United States of America; University of Hyderabad, INDIA

## Abstract

Atrazine is a common agricultural herbicide previously shown to promote epigenetic transgenerational inheritance of disease to subsequent generations. The current study was designed as an epigenome-wide association study (EWAS) to identify transgenerational sperm disease associated differential DNA methylation regions (DMRs) and differential histone retention regions (DHRs). Gestating female F0 generation rats were transiently exposed to atrazine during the period of embryonic gonadal sex determination, and then subsequent F1, F2, and F3 generations obtained in the absence of any continued exposure. The transgenerational F3 generation males were assessed for disease and sperm collected for epigenetic analysis. Pathology was observed in pubertal onset and for testis disease, prostate disease, kidney disease, lean pathology, and multiple disease. For these pathologies, sufficient numbers of individual males with only a single specific disease were identified. The sperm DNA and chromatin were isolated from adult one-year animals with the specific diseases and analyzed for DMRs with methylated DNA immunoprecipitation (MeDIP) sequencing and DHRs with histone chromatin immunoprecipitation (ChIP) sequencing. Transgenerational F3 generation males with or without disease were compared to identify the disease specific epimutation biomarkers. All pathologies were found to have disease specific DMRs and DHRs which were found to predominantly be distinct for each disease. No common DMRs or DHRs were found among all the pathologies. Epimutation gene associations were identified and found to correlate to previously known disease linked genes. This is one of the first observations of potential sperm disease biomarkers for histone retention sites. Although further studies with expanded animal numbers are required, the current study provides evidence the EWAS analysis is effective for the identification of potential pathology epimutation biomarkers for disease susceptibility.

## Introduction

Chronic exposure to ecological toxicants can be highly detrimental to health outcomes of animal and human populations. Environmental exposures such as endocrine disrupting chemicals are a major source of concern for overall wildlife and human health [1, 2]. Environmental exposures during early development such as fetal or early postnatal periods have higher impact risk than later adult periods [3]. The herbicide atrazine is widely used in agriculture for the control of broadleaf and grassy weeds in corn, sorghum, sugarcane and other crops. Atrazine is found in many ground water sources in North America where the aquatic ecological effects are a source of concern, as well as impacts on human health [4]. The compound atrazine (2 chloro-4-ethylamino-6-isopropyl-amino-s-triazine) is persistent in the environment, has been shown to act as an endocrine disruptor (e.g. disrupts estrous cycle in rats), and promotes disease susceptibility such as mammary tumor development [5]. Hayes et al, 2011 surveyed the effects of atrazine on male gonads across all vertebrate classes, and found that the compound strongly demasculinizes and feminizes male gonads while decreasing androgen levels and inducing synthesis of estrogen [6]. Exposure to high doses of atrazine also results in increased weight loss of exposed progeny [7]. When gestating female rats were exposed to atrazine the offspring exhibited reduced birth weights, higher mortality rates at birth, and delayed puberty [8]. Low doses of atrazine exposures have been shown to have negative effects in a wild rodent species (*Caolmys laucha*), including reduction of motility in sperm and an increase in damage to sperm DNA in males [9]. Exposure to atrazine can also affect learning and memory. Maternal exposure to atrazine resulted in the offspring having impaired spatial learning and memory, damage to the hippocampal morphology, and a reduction in expression of genes related to memory formation in the hippocampus [10].

The European Union made the decision in 2003 to ban the use of atrazine due to the prevalent contamination of water sources, while the United States Environmental Protection Agency (EPA) has continued to permit the use of atrazine. Watersheds affected by the agricultural application of atrazine routinely contain concentrations of 5 μg/L, but can reach levels as high as 20 μg/L [4]. The Maximum Contaminant Level (MCL) for atrazine in public drinking water as defined by the EPA is 3 μg/L [11]. The probability that drinking water levels in the Midwest exceed the MCL is 25–50% of the time [12]. The atrazine no observed adverse effect level (NOAEL) is 3.5 mg/kg/day in humans, and 50–100 mg/kg/day in rats. The lowest observable adverse effect level is 100–300 mg/kg/day in rats [13, 14]. The 25 mg/kg/day atrazine exposure level used in the current study is considered a low-level exposure.

In human populations, atrazine contamination of drinking water has been associated with adverse birth outcomes such as small for gestational age, low and very low birth weight, and preterm or very preterm birth [15]. In utero exposure of human females to atrazine resulted in a significantly early menarche and early pubertal onset [16]. Winston et al, 2016 found an association between maternal atrazine exposure and the prevalence of hypospadias, showing an impact on male reproductive development [17].

In addition to the physiological impacts of atrazine exposure on numerous pathologies and disease, the molecular impacts on the genome through epigenetic processes need to be assessed to understand the developmental and generational actions of exposure to the environmental toxicant atrazine [18–20]. Epigenetic alteration is the most common molecular impact of environmental exposures. Epigenetics is defined as molecular factors and processes around the DNA that regulate genome activity independent of DNA sequence, and are mitotically stable [21]. Epigenetic processes include DNA methylation, histone modifications, non-coding RNA, chromatin structure, and RNA methylation. A wide range of environmental factors such as nutrition, stress and toxicants have been found to promote the epigenetic transgenerational inheritance of disease and phenotypic variation through epigenetic changes in the germline (sperm or eggs) [21–24]. Multigenerational exposure is implicated when multiple generations are exposed, such as a gestating female and fetus, which represent the F0 and F1 generations, as well as the germline that will generate the F2 generation [21, 25]. When epigenetic alterations and phenotypes are transmitted through the sperm or egg, without continued direct exposure, then epigenetic transgenerational inheritance is implicated [21, 25]. The current study focused on the transgenerational F3 generation and not the direct exposed F1 and F2 generations to remove the confounding effects of direct exposure and identify transgenerational disease biomarkers.

The epigenetic effects of environmental toxicants, such as atrazine, have been studied for many toxicants and across multiple species. Epigenetic modifications which have been studied include alterations in DNA methylation, histone retention, non-coding RNAs (ncRNA) and alterations in histone methylation [26]. A range of environmental epigenetic effects have been observed on female reproductive systems and the diseases transmitted to future generations, including detrimental effects on the thyroid, ovary, and uterus [21, 27]. DNA methylation is widely recognized as regulating transcriptional activity. In response to exposure to endocrine-disrupting methoxychlor (an estrogenic compound) or vinclozolin (an anti-androgenic compound), differential DNA methylation was transmitted through the germlines of rats, and resulted in the epigenetic transgenerational inheritance of disease, such as male infertility [28]. Di-2-ethylhexyl phthalate (DEHP) was found to have a germline-dependent inheritance pattern with paternally-exposed offspring exhibiting higher body weight and gonadal weight, lower serum testosterone, and lower fertility rates [29]. Vinclozolin was further shown to result in differential methylation which transmitted deleterious effects to the male reproductive system in mice [30]. The pesticide and endocrine disruptor DDT (dichloro-diphenyl-trichloroethane) has also been shown to induce alterations in DNA methylation which are transmitted across multiple generations [31, 32]. Atrazine has been further shown to suppress genes associated with DNA methylation ultimately resulting in hypomethylation in an F0 generation of medaka (*Oryzias latipes*) [33]. In this same study, abnormal sperm counts and abnormal sperm motility were observed in the F2 generation representing a transgenerational effect of atrazine exposure. Since different endocrine disruptor compounds can have distinct mechanisms of action and impact a variety of different diseases, it is anticipated the epigenetic impacts and potential disease epigenetic biomarkers may be distinct for different compounds. The current study focused on atrazine impacts, but observations will need to be compared to other endocrine disruptor actions in the future.

Histone proteins are involved in the organization of DNA, and these proteins undergo a variety of post-translational modifications [34]. The effects of toxicants on the epigenetic transgenerational inheritance of histone alterations is relatively novel, and is known to involve differential histone retention (DHR) sites [35]. DDT and vinclozolin have been shown to induce the epigenetic transgenerational inheritance of concurrent alterations in DHR, DMRs and non-coding RNA expression [26, 35]. Non-coding RNAs (ncRNAs) are a group of RNAs that do not encode functional proteins, but play an important regulatory role in epigenetic control [36]. Long and small ncRNAs are proposed as important regulators of epigenetic transgenerational inheritance, particularly in response to exposure to toxicants and stress [26, 37]. Both DDT and vinclozolin exposure have been shown to result in transgenerationally inherited alterations in rats [26, 28]. The action of the herbicide atrazine has been previously shown to promote the epigenetic transgenerational inheritance of increased testis disease, mammary tumors, early onset of puberty, and a lean phenotype in the F3 generation of rats, where the gestating female F0 generation was exposed [38]. The current study extends this analysis to investigate the induced transgenerational disease specific epigenetic sperm DMRs and DHRs.

The identification of unique sets of DMRs which can be associated with a particular disease provides potential biomarkers of transgenerational disease [39, 40]. Such epigenetic biomarkers have the potential to facilitate diagnosis of both disease susceptibility and individual ancestral exposures. Previous experiments have identified the epigenetic transgenerational inheritance of pathology associated DNA methylation alterations, differential histone retention sites, and non-coding RNAs following gestational environmental exposures to vinclozolin [31] or DDT [32]. Differential DNA methylation epigenetic transgenerational biomarkers have also been identified following ancestral atrazine exposure [38]. The current investigation was designed to examine the presence of specific epigenetic alterations resulting from ancestral atrazine exposure which may serve as epigenetic biomarkers of transgenerational disease. Both alterations in DNA methylation and histone retention sites in the sperm were investigated in relation to atrazine induced epigenetic transgenerationally inherited disease.

## Results

### Animal model

As previously described [38], outbred Sprague Dawley gestating female rats (F0 generation) were administered an intraperitoneal dose of 25 mg/kg body weight of atrazine (4% of rat oral LD50 [41] and 50% of NOAEL [42]). These doses were administered at 90 days of age, during embryonic days 8–14 (E8-E14) of fetal gonadal sex determination. The F1 generation offspring was directly exposed as a fetus and F2 generation grand-offspring exposed as the germline in the F1 generation. These were each bred at 90 days of age within the lineage. The F3 generation great-grand-offspring is required to establish the transgenerational inheritance generation of ancestral exposure. This transgenerational generation was the focus of the current study. The F1 and F2 generations are examined in the previous publication [38]. A control lineage was established that used F0 gestating rats exposed to the vehicle control dimethyl sulfoxide (DMSO). Disease pathology was evaluated in atrazine exposure and control lineages at 1 year of age. The atrazine exposure lineage transgenerational individuals with specific disease or pathology were grouped as representatives of the pathology exhibited. The remaining individuals were grouped as “no disease.” Comparisons between these two groups were made during analysis of sperm DNA methylation and histone retention. The differentially methylated regions and differential histone retention site allows the identification of specific disease associated epigenetic biomarkers.

### Pathology analysis

As previously described [38] in the Methods, pathology analysis was assessed with histology sections of testis, kidney, prostate, and gonadal fat pads. The complete histological sections were analyzed by two different observers blinded to the exposure, unless they disagreed, and then an additional different third observer was used. The pathology parameters identified were as previously described in the Methods [38]. In brief, each counter records the incidence of abnormalities in each tissue. In testis, atrophy of a seminiferous tubule, the arrest of maturation of sperm (indicated by sloughed cells in the center of the tubule), and the presence of vacuoles were indicated disease pathologies. The abnormalities counted in kidney include a reduction in size of glomeruli, a thickening of the Bowman’s capsule, and the presence of cysts. Prostate abnormalities counted include atrophy of the epithelial cells, hyperplasia in the epithelial layer, and the presence of vacuoles within the epithelial layer of the prostatic glands. Obese and lean phenotypes were assigned following assessment of adipocyte size (area), body mass index (BMI) and abdominal adiposity. Late puberty was noted during development. The individual animals are listed in Table 1. A (+) indicates presence of disease and (-) indicates absence of disease for the current F3 generation atrazine lineage male pathology. The control lineage males were analyzed in a similar manner to allow a comparison to assess atrazine induced disease in the atrazine lineage, S1 Table. In contrast to the previous study [38], only the individuals with a single disease for a specific pathology were used for that pathology molecular analysis. Animals exhibiting more than one disease are all listed under the category “Multiple Disease.” Due to low prevalence of disease in the control animal groups, S1 Table, those animals were not used in the identification of epigenetic biomarkers.

**Table 1:** Individual animal pathology. F3 generation atrazine lineage males pathology. The individual animals for the atrazine lineage males are listed and a (+) indicates presence of disease and (-) absence of disease. The shaded boxes represent animals with a single disease (+) or no disease (0) that were used for the molecular analysis. The number of disease animals / total animals is presented.

Animal ID	Puberty		Testis	Prostate	Kidney	Lean	Obese	Tumor	Multiple Disease	Total Disease
	Early	Late								
AM1	**-**	**-**	**-**	**-**	**+**	**-**	**-**	**-**	**-**	**1**
AM2	**-**	**-**	**-**	**+**	**+**	**-**	**-**	**-**	**+**	**2**
AM3	**-**	**-**	**-**	**-**	**-**	**-**	**-**	**-**	**-**	**0**
AM4	**-**	**-**	**-**	**-**	**+**	**+**	**-**	**-**	**+**	**2**
AM5	**-**	**-**	**-**	**-**	**+**	**-**	**-**	**-**	**-**	**1**
AM6	**-**	**-**	**+**	**-**	**-**	**+**	**-**	**-**	**+**	**2**
AM7	**-**	**-**	**-**	**-**	**-**	**-**	**-**	**-**	**-**	**0**
AM8	**-**	**-**	**-**	**-**	**-**	**-**	**-**	**-**	**-**	**0**
AM9	**-**	**-**	**-**	**-**	**-**	**-**	**-**	**-**	**-**	**0**
AM10	**-**	**-**	**+**	**-**	**-**	**-**	**-**	**-**	**-**	**1**
AM11	**-**	**-**	**-**	**-**	**-**	**+**	**-**	**-**	**-**	**1**
AM12	**-**	**-**	**-**	**-**	**-**	**-**	**-**	**-**	**-**	**0**
AM13	**-**	**-**	**-**	**-**	**-**	**-**	**-**	**-**	**-**	**0**
AM14	**-**	**-**	**+**	**-**	**-**	**+**	**-**	**-**	**+**	**2**
AM15	**-**	**-**	**-**	**-**	**-**	**-**	**-**	**-**	**-**	**0**
AM16	**-**	**-**	**-**	**-**	**+**	**-**	**-**	**-**	**-**	**1**
AM17	**-**	**-**	**-**	**-**	**-**	**-**	**-**	**-**	**-**	**0**
AM18	**-**	**-**	**-**	**-**	**-**	**-**	**-**	**-**	**-**	**0**
AM19	**-**	**-**	**+**	**-**	**-**	**-**	**+**	**-**	**+**	**2**
AM20	**-**	**-**	**-**	**+**	**-**	**-**	**+**	**-**	**+**	**2**
AM21	**-**	**-**	**+**	**+**	**-**	**+**	**-**	**-**	**+**	**3**
AM22	**-**	**-**	**-**	**-**	**-**	**-**	**-**	**-**	**-**	**0**
AM23	**-**	**-**	**-**	**-**	**-**	**+**	**-**	**-**	**-**	**1**
AM24	**-**	**+**	**+**	**-**	**-**	**-**	**-**	**-**	**+**	**2**
AM25	**-**	**+**	**-**	**-**	**-**	**-**	**-**	**-**	**-**	**1**
AM26	**-**	**+**	**-**	**-**	**-**	**-**	**-**	**-**	**-**	**1**
AM27	**-**	**+**	**+**	**-**	**-**	**+**	**-**	**-**	**+**	**3**
AM28	**-**	**+**	**-**	**-**	**-**	**-**	**-**	**-**	**-**	**1**
AM29	**-**	**+**	**-**	**-**	**-**	**-**	**-**	**-**	**-**	**1**
AM30	**-**	**-**	**-**	**-**	**-**	**-**	**+**	**+**	**+**	**2**
AM31	**-**	**-**	**-**	**-**	**-**	**-**	**-**	**-**	**-**	**0**
AM32	**-**	**-**	**-**	**-**	**-**	**-**	**-**	**-**	**-**	**0**
AM33	**-**	**-**	**-**	**-**	**-**	**-**	**-**	**-**	**-**	**0**
AM34	**-**	**-**	**-**	**-**	**-**	**-**	**-**	**-**	**-**	**0**
AM35	**-**	**-**	**-**	**-**	**-**	**-**	**-**	**-**	**-**	**0**
AM36	**-**	**-**	**+**	**-**	**-**	**-**	**-**	**-**	**-**	**1**
AM37	**-**	**-**	**+**	**-**	**-**	**+**	**-**	**-**	**+**	**2**
AM38	**-**	**-**	**-**	**-**	**-**	**-**	**-**	**-**	**-**	**0**
AM39	**-**	**-**	**+**	**-**	**-**	**-**	**-**	**-**	**-**	**1**
AM40	**-**	**-**	**+**	**-**	**-**	**-**	**-**	**-**	**-**	**1**
AM41	**-**	**-**	**-**	**-**	**-**	**-**	**-**	**-**	**-**	**0**
AM42	**-**	**-**	**-**	**-**	**-**	**+**	**-**	**-**	**-**	**1**
AM43	**-**	**-**	**-**	**-**	**-**	**-**	**-**	**-**	**-**	**0**
AM44	**-**	**-**	**-**	**-**	**-**	**-**	**+**	**-**	**-**	**1**
AM45	**-**	**-**	**-**	**-**	**-**	**-**	**-**	**-**	**-**	**0**
AM46	**-**	**+**	**+**	**-**	**+**	**+**	**-**	**-**	**+**	**4**
AM47	**-**	**+**	**-**	**+**	**-**	**+**	**-**	**-**	**+**	**3**
AM48	**-**	**+**	**-**	**-**	**-**	**-**	**-**	**-**	**-**	**1**
AM49	**-**	**+**	**-**	**-**	**-**	**-**	**-**	**-**	**-**	**1**
AM50	**-**	**-**	**-**	**-**	**-**	**+**	**-**	**-**	**-**	**1**
AM51	**-**	**-**	**-**	**-**	**-**	**-**	**-**	**-**	**-**	**0**
AM52	**-**	**-**	**-**	**-**	**-**	**+**	**-**	**-**	**-**	**1**
AM53	**-**	**-**	**-**	**-**	**+**	**+**	**-**	**-**	**+**	**2**
AM54	**-**	**-**	**-**	**-**	**+**	**+**	**-**	**-**	**+**	**2**
# affected	**0**	**10**	**12**	**4**	**8**	**15**	**4**	**1**		
# evaluated	**55**	**55**	**55**	**55**	**55**	**55**	**55**	**55**		

### Sperm DNA methylation

**Fig 1:**
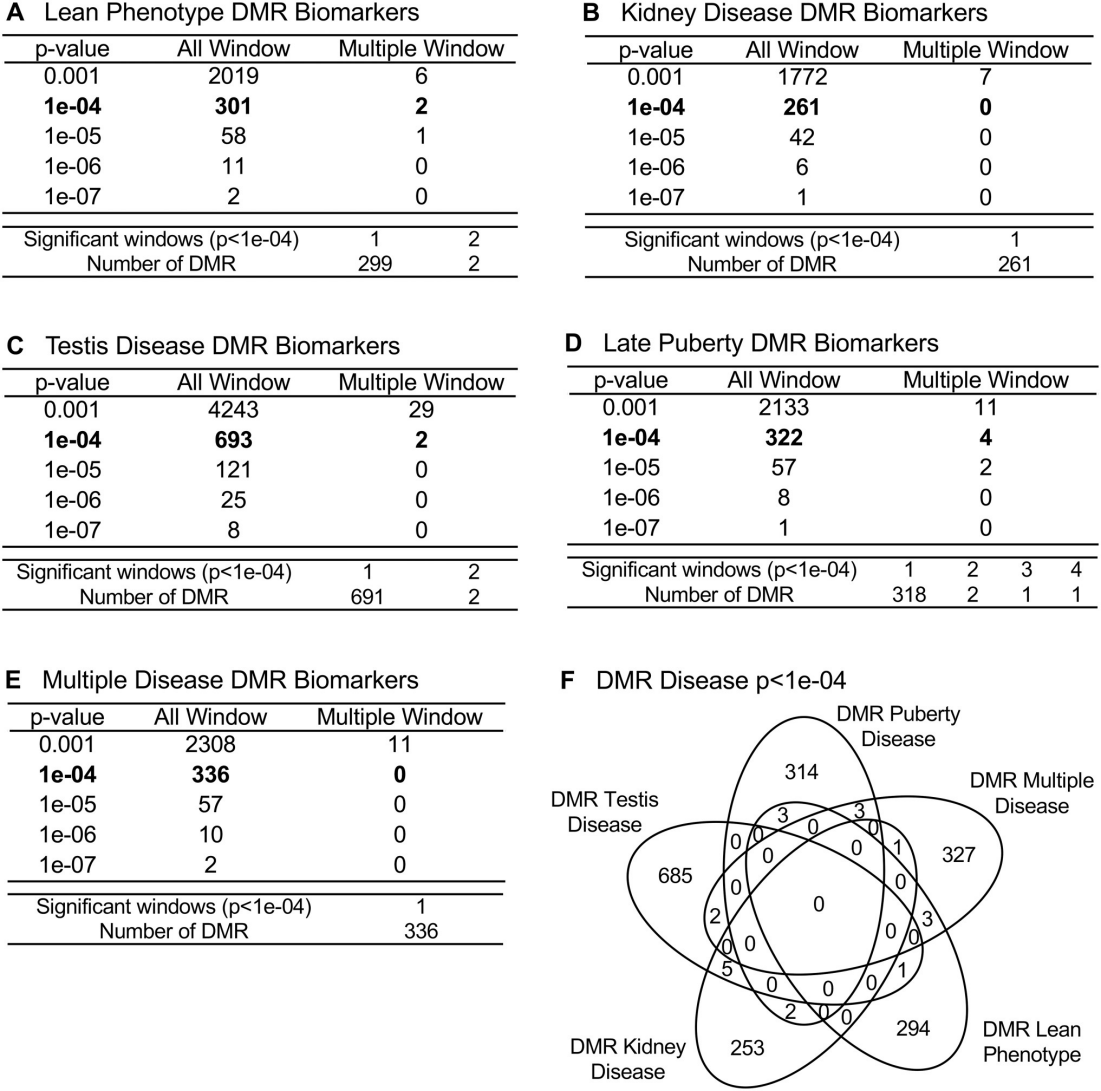
DMR identification and numbers. The number of DMRs found using different p-value cutoff thresholds. The All Window column shows all DMRs. The Multiple Window column shows the number of DMRs containing at least two nearby significant windows (1 kb each). The number of DMRs with the number of significant windows (1 kb per window) at a p-value threshold of p<1e-04 for DMR is bolded. **(A)** Lean phenotype DMRs; **(B)** Kidney disease DMRs; **(C)** Testis disease DMRs; **(D)** Late puberty DMRs; and **(E)** Multiple disease DMRs. **(F)** Venn diagram overlap disease specific DMR at p<1e-04.

The experimental design was focused on the identification of transgenerational DMRs and DHRs in sperm. Sperm were collected from the atrazine lineage F3 generation males for epigenetic analysis. DNA from the sperm was isolated and fragmented with sonication, as described in the Methods. The methylated DNA immunoprecipitation (MeDIP) using a methyl-cytosine antibody was used to identify alterations in DNA methylation. The methylated DNA fragments were then sequenced for an MeDIP-Seq analysis, as described in the Methods [31, 32]. The differential DNA methylation regions (DMRs) were identified between the disease versus non-disease within the atrazine lineage animals (Fig 1). The transgenerational sperm DMR numbers are presented in Fig 1 for different edgeR statistical p-value cutoff thresholds, and p<1e−04 (diseased versus non-diseased) for the atrazine lineage were selected as the threshold for subsequent analyses. Disease-specific DMRs were then identified among the atrazine treated animals exhibiting disease phenotypes, including lean phenotype, kidney disease, testis disease, late puberty, and multiple disease, compared against atrazine lineage individuals exhibiting no disease (Fig 1A–1E). The all windows represents all DMR windows, and multiple site are those with nearby 1 kb sites. Only the late puberty DMRs had multiple sites. In the current analysis 1000 bp windows were used in the identification of DMRs.

The previously reported transgenerational F3 generation sperm atrazine versus control lineage DMRs identified atrazine induced sperm differential DNA methylation [38]. A bioinformatics reanalysis of these sperm samples used updated method parameters, including a wider 1000 bp window size and increased read depth required for each window. The previous study [38] for pathology analysis utilized a different method to categorize animals as presenting disease. Any animals exhibiting any disease were listed in the group for that disease, whether these animals exhibited any other disease or not. An animal exhibiting two different pathologies, for example both lean phenotype and kidney disease, may present confounding influences for the identification of lean-phenotype specific epigenetic alterations. These confounding inputs may increase variability in the disease-specific epigenetic marks. In the current study, animals for each disease category were chosen only if they exhibit that single disease and no other. Any animals exhibiting multiple disease phenotypes were grouped in the multiple (≥ 2) disease category. A comparison of these two studies demonstrates a difference in the disease specific DMR sets, which were identified. For example, the lean phenotype was found to have 301 total DMRs at an edgeR p-value threshold of p<1e-4 with 2 of these having multiple neighboring windows (Fig 1A) in the current study, and 467 total DMRs at an edgeR p-value threshold of p<1e-5 with 7 having multiple neighboring windows [38] with the previous methodology. The overlap of these disease specific DMR sets was negligible with those of the previous disease DMRs [38]. By removing the confounding inputs from multiple diseases in one disease category, the current methodology should identify more accurate epigenetic marks associated with each individual pathology at the risk of lowering analysis power due to smaller sample sizes. The current study identified disease-specific DMRs (301 lean, 693 testis, 261 kidney, and 322 late puberty, and 336 multiple disease) at p<1e-04 that are presented in Fig 1 and listed in S2–S6 Tables. The log-fold change in DNA methylation is presented and an increase in methylation is associated with 27% pubertal abnormality DMRs, 56% testis disease DMRs, 43% kidney disease DMRs, 25% lean pathology DMRs, and 37% multiple disease DMRs. The others all had a decrease in DNA methylation. Observations indicate atrazine can promote germline epigenetic alterations in sperm DNA methylation that appear disease specific.

**Fig 2:**
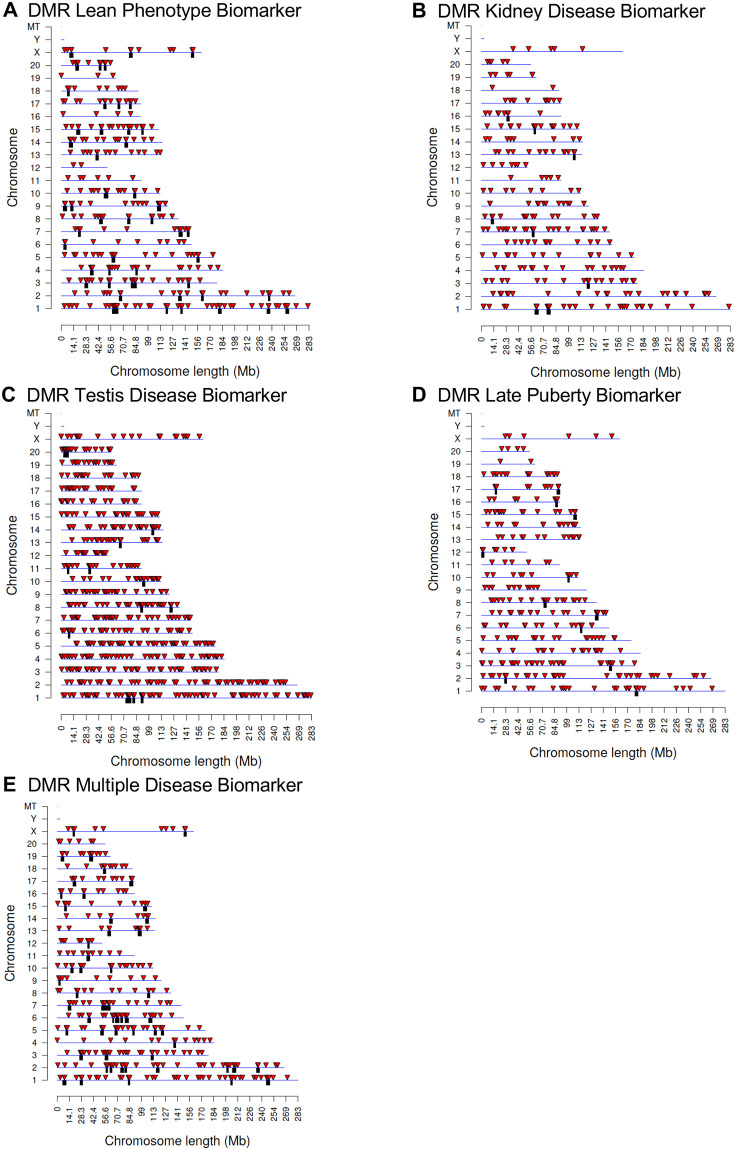
DMR chromosomal locations. The DMR locations on the individual chromosomes is represented with an arrowhead and a cluster of DMRs with a black box. All DMRs containing at least one significant window at a p-value threshold of p<1e-04 for DMR are shown. **(A)** Lean phenotype DMRs; **(B)** Kidney disease DMRs; **(C)** Testis disease DMRs; **(D)** Late puberty DMRs; and **(E)** Multiple disease DMRs. The chromosome number versus size (megabase) is presented.

Chromosomal locations of the DMRs are presented in Fig 2 with DMR represented as arrowheads and DMR clusters indicated by black boxes. DMRs are present on all chromosomes except the Y chromosome and mitochondrial DNA (MT). The wide distribution of DMRs across chromosomes provides evidence that the epigenetic effects of transgenerational atrazine exposure are genome-wide. The genomic features are detailed in S1 Fig. The CpG density of the DMRs is low with the majority of CpGs between 1 and 3 sites per 100 bp. The length of most DMRs is between 1,000 and 3,000 base pairs. The principal component analyses (PCA) of the RPKM adjusted read depths at differential DMR sites for each sample are shown in S2 Fig. The PCA plots shows how the DMR samples cluster according to disease compared to non-disease and indicate potential outliers in the data (none observed) when DMR sites are evaluated (S2 Fig).

### Sperm histone retention

**Fig 3:**
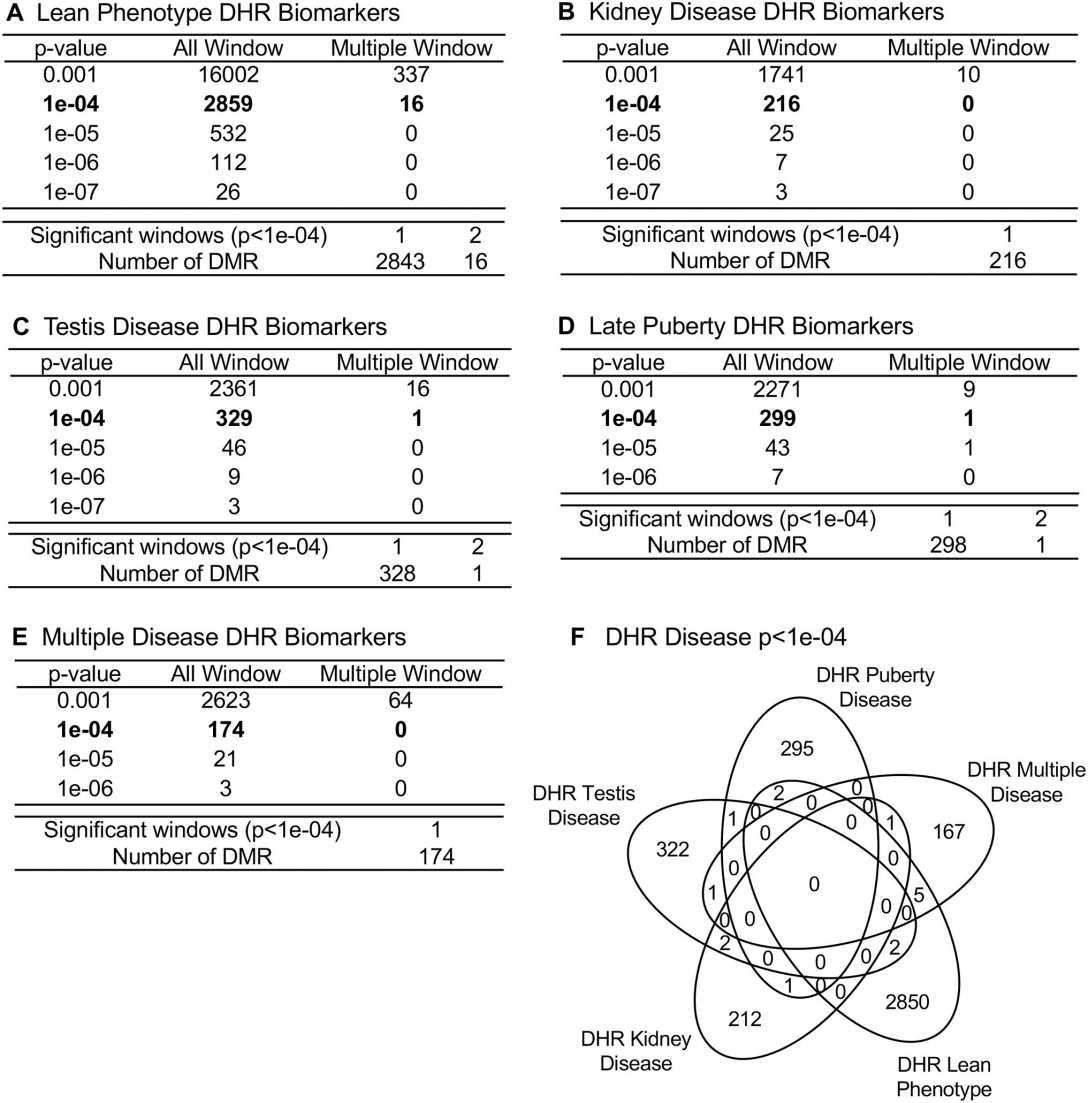
DHR identification and numbers. The number of DHRs found using different p-value cutoff thresholds. The All Window column shows all DHRs. The Multiple Window column shows the number of DHRs containing at least two nearby significant windows (1 kb each). The number of DMRs with the number of significant windows (1 kb per window) at a p-value threshold of p<1e-04 for DHR is presented. **(A)** Lean phenotype DHRs; **(B)** Kidney disease DHRs; **(C)** Testis disease DHRs; **(D)** Late puberty DHRs; and **(E)** Multiple disease DHRs.

Differential histone retention in sperm has previously been shown to be important in epigenetic transgenerational inheritance [35]. For the current study, sperm were collected from the atrazine lineage F3 generation males for analysis. Chromatin from the sperm was isolated and fragmented. A histone H3 antibody is used in a chromatin immunoprecipitation (ChIP) analysis. The retained fragments of DNA were then sequenced for a ChIP-Seq analysis, similar to the MeDIP-Seq analysis, as described in the Methods. This analysis yields the differential histone retention sites (DHRs), which were identified in the sperm using a comparison between the disease specific and non-disease atrazine exposure lineage males (Fig 3, S7–S11 Tables). The same sets of animals were used for the identification of DHRs associated with each disease as were used in the identification of DMRs associated with each disease. The lean phenotype exhibited the highest number of differentially retained histone sites, with 2859 found at edgeR p<1e-04, indicating the greatest amount of epigenetic shift was associated with individuals exhibiting a lean phenotype following transgenerational exposure to atrazine (Fig 3A). The other diseases each yield several hundred transgenerational DHRs at an edgeR p-value threshold of p<1e-4, Fig 3B–3E, S7–S11 Tables. The log-fold change in DHRs is presented and an increase in histone retention is associated with 44% of pubertal abnormality DHRs, 39% of testis disease DHRs, 36% of kidney disease DHRs, 35% of lean pathology DHRs, and 53% of multiple disease DHRs.

**Fig 4:**
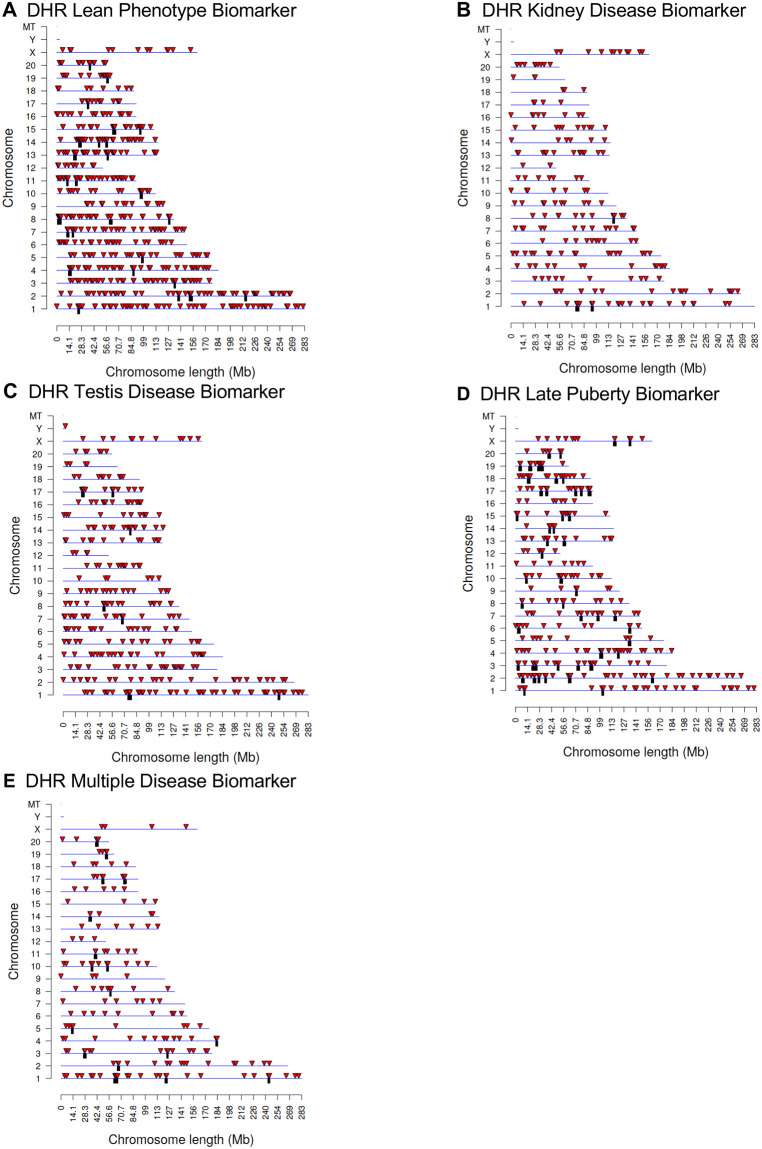
DHR chromosomal locations. The DHR locations on the individual chromosomes is represented with an arrowhead and a cluster of DHRs with a black box. All DHRs containing at least one significant window at a p-value threshold of 1e-04 for DHR are shown. **(A)** Lean phenotype DHRs (p<1e-5); **(B)** Kidney disease DHRs; **(C)** Testis disease DHRs; **(D)** Late puberty DHRs; and **(E)** Multiple disease DHRs.

A similar genome wide response is seen in the widespread distribution of DHRs across chromosomes (Fig 4) as was seen with the DMRs. Therefore, both epigenetic mechanisms examined, differential DNA methylation regions and differential histone retention regions, show this genome wide epigenetic response transgenerationally. The CpG density within differential histone retention sites is low with the majority of CpGs between 1 and 3 sites per 100 bp (S3 Fig). The length of most DHRs is between 1,000 and 3,000 base pairs are shown in S3 Fig. A principal component analysis (PCA) is presented for the disease specific DHRs (S4 Fig). There is distinct clustering and no outlier samples for each of the diseases analyzed compared to the non-disease when the RPKM read depth at DHR sites used in the analysis.

Although a previous study investigated the ability of atrazine to promote transgenerational sperm DMRs [38], no previous analysis of atrazine effects on DHRs has been reported. Therefore, in addition to the atrazine lineage disease specific DHR analyses, control lineage F3 generation (S1 Table) sperm was compared to the F3 generation atrazine lineage male sperm. The control versus atrazine lineage F3 generation sperm identified DHRs at a variety of statistical thresholds, with 786 DHRs at p<1e-04, S5A Fig and S12 Table. The majority were single 1 kb sites, but some multiple nearby 1 kb sites were also observed. The chromosomal locations demonstrated a genome-wide distribution, S5B Fig. The CpG density of the DHRs was predominantly 1–3 CpG/100 bp, S5C Fig, and size of the DHRs was predominantly 1–4 CpG, S5D Fig. A PCA demonstrated distinct clustering and no outliers of the control versus atrazine samples with read depths at DHR sites considered, S5E Fig. Therefore, in addition to atrazine induced transgenerational DMRs [38], there is also an induction of DHRs in the sperm. This provides additional support for a role of sperm DHRs in the sperm mediated epigenetic transgenerational inheritance phenomenon.

### Epimutation comparisons

A comparison of the different epigenetic data sets for each disease category among both DMRs and DHRs demonstrated only a handful of overlapping sites at the statistical threshold of p<1e-04 (Figs 1F and 3F). Most of the epimutations associated with each disease category are unique to either a differentially methylated region or a differential histone retention site at this statistical threshold. To more rigorously compare the different datasets, an extended overlap was performed. A comparison with a reduced statistical threshold of edgeR p<0.05 was used to further evaluate the potential overlap of the DMR and DHR data sets at p<1e-04. By lowering the stringency to a p<0.05 for the comparison (herein called the extended overlap), this procedure allows for increased overlap with higher p-values. The extended overlaps between the atrazine lineage puberty, testis, kidney, lean, and multiple disease DMRs and DHRs are shown in Table 2. A comparison of the p<1e-04 for the DMRs and DHRs between the different data sets at p< 0.05 demonstrates a much higher overlap between the various DMRs and DHRs identified than the Venn diagrams in Figs 1F and 3F. The extended overlap also shows any overlaps between DMRs and DHRs for the individual disease comparisons. The range of overlap between atrazine lineage disease DMR or DHR is 8–37%, Table 2. Comparison between the DMRs and DHRs for the specific diseases had a lower range of overlap with 2–17%, Table 2. The highest level of overlap was observed between the lean disease and multiple disease for both the DMRs and DHRs, Table 2. Therefore, the majority of the disease specific DMRs and DHRs at p<1e-04 are distinct, but an overlapping set of DMRs and DHRs are common between two diseases. An overlap of all the disease specific DMRs and DHRs was performed at p<0.05 and identified 75 DMRs and 36 DHRs that are common between puberty abnormalities, testis disease, kidney disease, lean pathology and multiple disease at p<0.05. A Venn diagram of this common set of DMRs and DHRs at p<0.05 with the specific diseases at p<1e-04 demonstrated no overlap in common with all pathologies, S6 Fig. Therefore, no common DMRs or DHRs were observed with all the different pathologies.

**Table 2:** Extended overlap disease DMRs and DHRs. The p-value DMR/DHR set at p<1e-04 for specific diseases are compared to the p<0.05 DMR/DHR to identify potential overlap between the different pathologies with DMR or DHR number and percentage of the total presented. The gray highlight is the expected 100% overlap.

**p<0.05**	**Lean DMR**	**Kidney DMR**	**Testis DMR**	**Late Puberty DMR**	**Multiple (2+) DMR**
**p<1e-04**					
**Lean DMR**	301 (100.0%)	42 (14.0%)	45 (15.0%)	83 (27.6%)	114 (37.9%)
**Kidney DMR**	42 (16.1%)	261 (100.0%)	62 (23.8%)	50 (19.2%)	48 (18.4%)
**Testis DMR**	64 (9.2%)	103 (14.9%)	693 (100.0%)	63 (9.1%)	122 (17.6%)
**Late Puberty DMR**	81 (25.2%)	44 (13.7%)	51 (15.8%)	322 (100.0%)	113 (35.1%)
**Multiple (2+) DMR**	124 (36.9%)	68 (20.2%)	79 (23.5%)	125 (37.2%)	336 (100.0%)
**Lean DHR**	150 (5.2%)	135 (4.7%)	256 (9.0%)	138 (4.8%)	177 (6.2%)
**Kidney DHR**	6 (2.8%)	14 (6.5%)	24 (11.1%)	5 (2.3%)	13 (6.0%)
**Testis DHR**	14 (4.3%)	17 (5.2%)	20 (6.1%)	14 (4.3%)	12 (3.6%)
**Late Puberty DHR**	8 (2.7%)	10 (3.3%)	10 (3.3%)	9 (3.0%)	20 (6.7%)
**Multiple (2+) DHR**	11 (6.3%)	10 (5.7%)	12 (6.9%)	9 (5.2%)	9 (5.2%)
**Lean DMR**	35 (11.6%)	9 (3.0%)	14 (4.7%)	10 (3.3%)	25 (8.3%)
**Kidney DMR**	34 (13.0%)	17 (6.5%)	20 (7.7%)	14 (5.4%)	35 (13.4%)
**Testis DMR**	111 (16.0%)	54 (7.8%)	54 (7.8%)	35 (5.1%)	93 (13.4%)
**Late Puberty DMR**	56 (17.4%)	14 (4.3%)	23 (7.1%)	27 (8.4%)	14 (4.3%)
**Multiple (2+) DMR**	51 (15.2%)	10 (3.0%)	16 (4.8%)	14 (4.2%)	20 (6.0%)
**Lean DHR**	2859 (100.0%)	241 (8.4%)	256 (9.0%)	297 (10.4%)	739 (25.8%)
**Kidney DHR**	39 (18.1%)	216 (100.0%)	30 (13.9%)	34 (15.7%)	33 (15.3%)
**Testis DHR**	40 (12.2%)	48 (14.6%)	329 (100.0%)	54 (16.4%)	62 (18.8%)
**Late Puberty DHR**	48 (16.1%)	36 (12.0%)	38 (12.7%)	299 (100.0%)	40 (13.4%)
**Multiple (2+) DHR**	63 (36.2%)	23 (13.2%)	34 (19.5%)	29 (16.7%)	174 (100.0%)

### Epimutation gene associations

**Fig 5:**
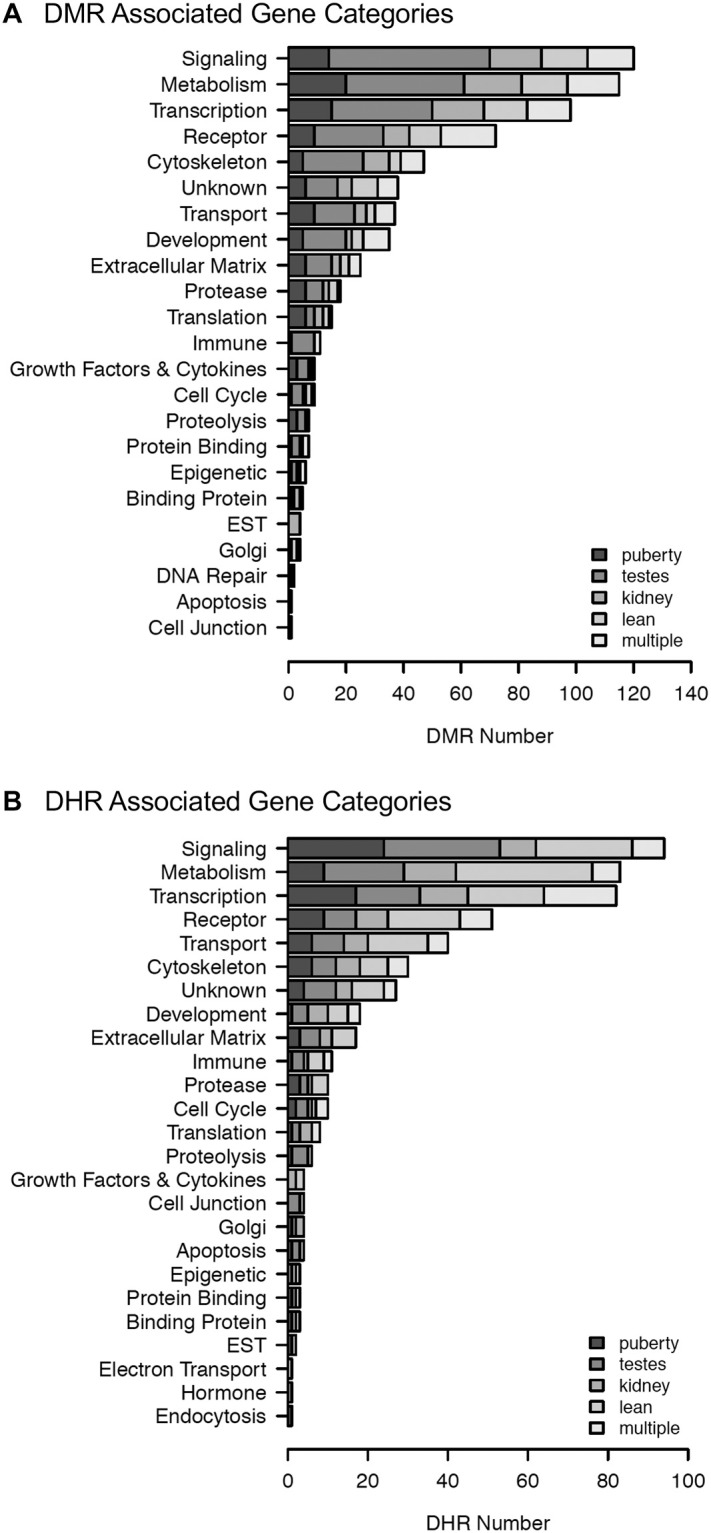
Associated gene categories. **(A)** DMR associated gene categories. **(B)** DHR associated gene categories. The gene categories and number of associated genes are presented for each disease group.

Between 49% and 55% of the DMRs and between 41% and 54% of the DHRs from the specific disease prevalence have epimutations associated with genes. These epimutation associated genes are presented in S2–S11 Tables. The gene associations were sorted into relevant functional categories for each specific disease biomarker dataset within the atrazine lineage (Fig 5). Disease specific epimutation associated genes identified in the analysis of DMRs are shown in Fig 5A, where the predominant categories identified were signaling, transcription and metabolism. The epimutation associated genes identified in the DHR analysis are shown in Fig 5B, where the same categories are predominant. The DMRs and DHRs associated with gene pathways are presented in S7 Fig. Although some of the pathways were common for the specific disease and pathways, generally the DMR pathways and DHR pathways were distinct.

**Fig 6:**
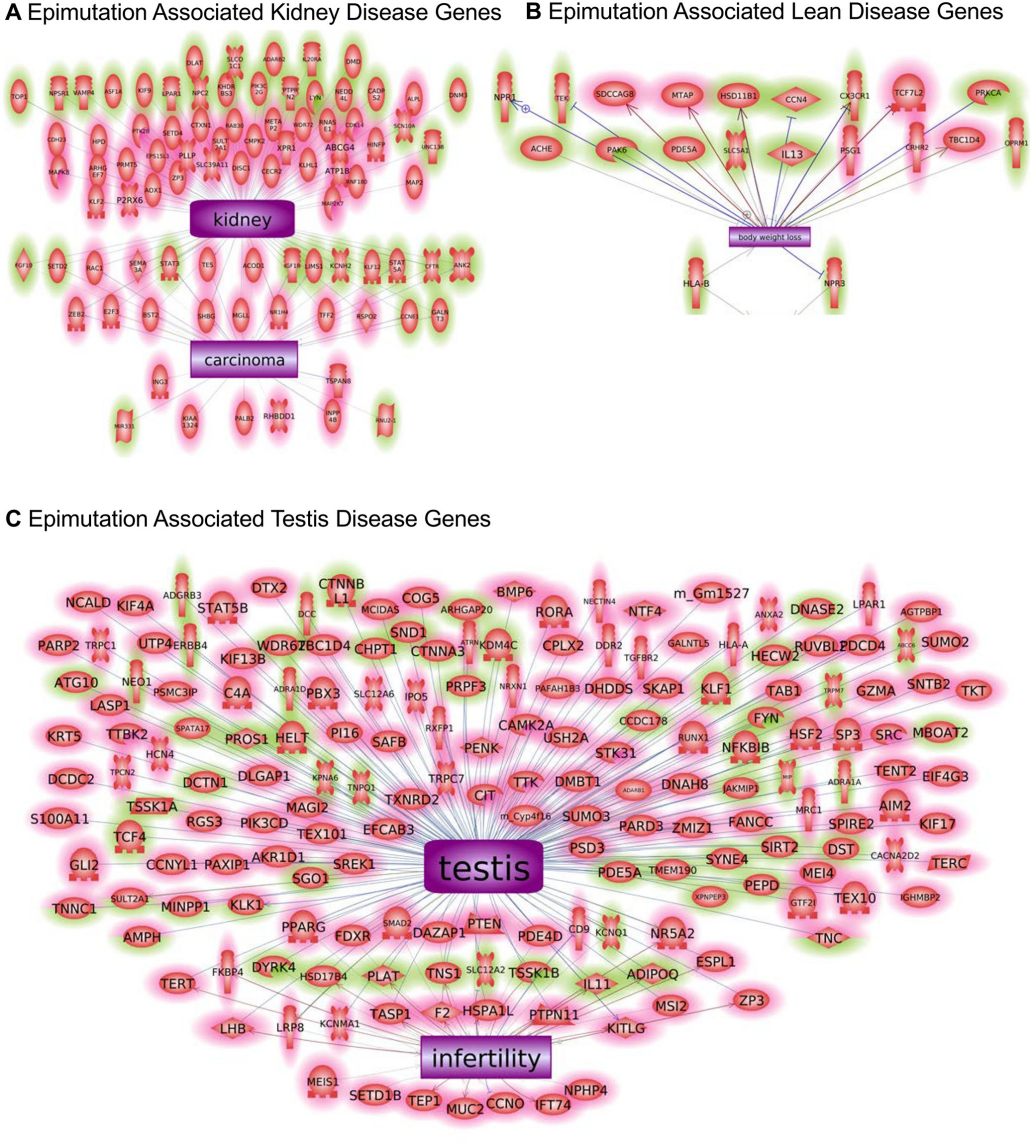
Epimutation associated previously identified disease genes. **(A)** Epimutation associated kidney disease genes. **(B)** Epimutation associated lean disease genes. **(C)** Epimutation associated testis disease genes. The green shading represents DMRs and pink shading DHRs. The gene shapes are identified as follows inset in Fig 7.

**Fig 7:**
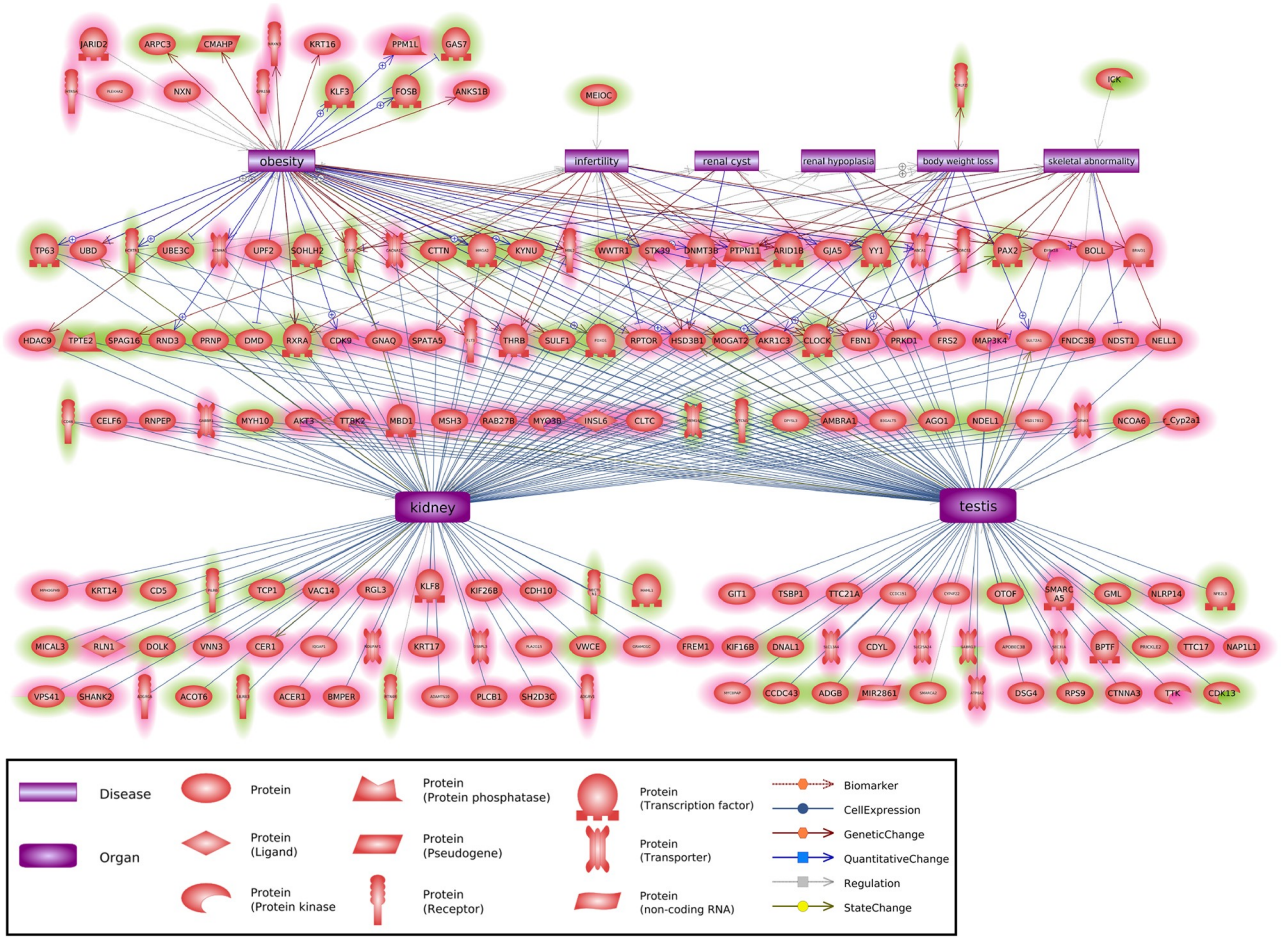
Multiple disease associated previously identified disease genes. The disease categories and gene identified shapes are presented. The green shading represents DMRs and pink shading DHRs.

The final analysis used a Pathway Studio gene network approach to associate previously identified disease specific associated genes with the disease specific DMRs and DHRs identified. A large number of previously identified kidney disease linked genes were found to be within the DMR and DHR associated genes, Fig 6A. A number of previously identified obesity and breast cancer-related genes were also associated with the DMR and DHR associated genes, Fig 6B. The highest number of previously identified genes was associated with testis disease and male infertility associated genes, Fig 6C. These observations help validate the DMR and DHR associated genes with the different specific diseases. Interestingly, the multiple disease associated DMRs and DHRs had disease genes previously identified for kidney disease, testis disease, obesity and male infertility, Fig 7. As expected, the multiple disease DMR and DHR associated genes had a mixture of the various diseases identified involved.

## Discussion

The human population is exposed to a large number of environmental and ecological toxicants throughout development, and many of these toxicants are endocrine disrupting chemicals (EDCs) [3]. While direct exposures to environmental toxicants are a cause for concern for the health of humans, as well as wildlife populations, the transgenerational inheritance of exposure to toxicants provides additional potential for generational negative health impacts [43, 44]. The different EDCs have distinct molecular actions and epigenetic impacts when altering health and pathology. Therefore, the actions of any individual EDC will need to be compared to those of others in future studies. The extensively used herbicide atrazine is a known endocrine disrupting chemical, and has been associated with a wide range of detrimental health effects [5–9]. The transgenerational inheritance of these detrimental health effects, or pathologies, [38] has critical relevance to the use and allowed levels of exposure to toxicants for both the human population and the environment. Therefore, the current study examines the association between pathologies resulting from transgenerational exposure to atrazine and the associated epigenetic alterations associated with these pathologies.

Atrazine exposure has previously been linked to epigenetic shifts in rats [38] and in humans [45]. The current study demonstrates not only DNA methylation alterations but also provides one of the first observations of disease-specific alterations in histone retention sites. These transgenerational epigenetic shifts are associated with ancestral exposure to the herbicide atrazine, and potential disease specific biomarkers for pathologies were identified. The pathologies observed with sufficient numbers of animals include the lean phenotype, kidney disease, testis disease, late puberty, and multiple disease where individuals exhibited two or more different pathologies. The pathologies examined in this study are relevant for humans, particularly prostate disease, which is one of the most prominent diseases in human males [46], and delayed puberty associated with exposure to EDCs is a known pathology in humans [47]. Among aging populations, kidney disease prevalence increases in human populations [48]. The phenotypic effects of shifts in BMI, either increased or decreased, due to exposure to toxicants can influence lifelong health trajectories and resulting pathologies [49]. The association of epigenetic biomarkers with these common disease pathologies may provide particularly valuable indicators of the presence of or susceptibility to disease in the human population. Genome-wide association studies (GWAS) have generally found associations with specific genetic mutations, but typically these appear in less than 1% of the disease population.

The frequency of epimutations tend to be much higher among individuals with disease [31, 32, 38, 50]. In the current study, the number of differential DNA methylated regions (DMRs) occurring in the transgenerational males is between 300 and 600 at an edgeR p<1e-04 threshold (Fig 1) for each pathology. This supports the prevalence of epigenetic alterations as important biomarkers of disease. There is a sub-population of DMRs and DHRs with overlap between the different individual disease pathologies (Table 2), suggesting some of the epimutations are less disease specific and indicative of multiple pathologies. This suggests some common epimutations may have a role in promoting generational disease susceptibility, however, no common DHRs or DMRs were found for all diseases. Therefore, the majority of epigenome-wide association study (EWAS) associated epimutations were disease specific. The DMR and DHR associated genes suggest the most affected gene categories were signaling, metabolism, and transcription. The analysis of previously identified disease associated genes yields a number of links with the diseases examined in the current study (Figs 5 and 6). The association of these previously identified disease genes with the disease specific epigenetic marks in the current study, including DMRs and DHRs, provides further validation of the disease biomarkers identified herein. In addition to the altered methylation observed among the atrazine lineage males, there were also alterations in differential histone retention regions (DHRs). This represents an additional transgenerational epigenetic response to toxicant exposure. The DHRs also provide a potential epigenetic biomarker for disease. The altered histone retention signature was in the range of 200–300 DHRs at an edgeR p<1e-04 threshold (Fig 3) for most disease pathologies. However, there were nearly 3,000 DHRs (Fig 3B) associated with the lean phenotype, indicating the lean physiology is a particularly strong phenotypic response to transgenerational atrazine exposure. Interestingly, some DMRs and DHRs had overlap between the different individual diseases (Table 2), suggesting some of the epimutations may act as biomarkers for susceptibility of multiple pathologies. Table 2 also shows the extended overlap between DMRs and DHRs. Late puberty associated DMR overlap 28% with lean phenotype DMR. A 24% overlap is found between testis and kidney disease associated DMR. Most of these overlaps including DMRs and DHRs are quite low. Therefore, transgenerational disease specific epigenetic biomarkers are present, along with some in common, S2–S11 Tables. The gene categories for the DHRs are quite similar to the DMR categories, with the same three processes most relevant. DHR associated disease genes are also shown in Figs 6 and 7. As with the DMRs, these previously identified disease associated genes may provide disease specific validation for the epigenetic biomarkers in the current study.

The atrazine induced transgenerational lean pathology was quite prevalent in the exposed population, with nearly a third of the animals presenting this pathology. The epigenetic signature was also strong, with 301 DMRs and 2,859 DHRs at an edgeR p<1e-04 threshold. A lean phenotype can be as significant an indicator of disease as an obese phenotype [51]. More importantly, the early developmental effects of exposure to EDCs could have magnified effects in later development and this may be particularly important with regards to metabolic disorder of any type [52, 53].

A limitation of the current study was the low numbers of animals with a specific individual disease. The total number of animals used was much higher, but the number of those with a specific disease was relatively low. Although a stringent edgeR p-value was used to identify and analyze the disease associated DMRs, further analyses adjusting for multiple testing using the false discovery rate (FDR) resulted in FDR p-values for the disease epimutations of >0.1 in all comparisons except the lean phenotype. The low sample number is likely the most important limitation in the current analysis. Potential higher variability in the data needs to be considered even though higher edgeR values were used, but this does not address multiple testing corrections. Future studies will need to use higher n-values and/or better statistical models to reduce this analysis limitation [54–59].

These results show that atrazine induced transgenerational DMRs and DHRs are present in sperm for specific disease pathologies. Thus, there is a potential for epigenetic biomarkers to be used to assess paternal transmission of various disease susceptibilities to the offspring. These epigenetic biomarkers may even be used in preconception diagnoses to determine disease susceptibilities for subsequent generations. This could lead to preventative therapeutics or lifestyles to be used in the mitigation of these disease susceptibilities. The previous study examining transgenerational exposure to the endocrine disruptor atrazine demonstrated epigenetic inheritance of sperm epimutation, as well as served as evidence for epigenetic biomarkers for disease to be identified and potentially used for diagnosis [38]. Here, we have included the report of potential epigenetic biomarkers in the form of differential DNA methylation regions and extended the epigenetic biomarkers to differential histone retention regions. With a relatively high prevalence of the epimutations in association with the disease pathologies, epigenetic diagnostics are poised to provide an important component of preventative medicine and disease management.

## Materials and methods

### Animal studies and breeding

As previously described [32, 50], female and male rats of an outbred strain Hsd:Sprague Dawley SD (Harlan) at 70 to 100 days of age were fed ad lib with a standard rat diet and ad lib tap water. Timed-pregnant females on days 8 through 14 of gestation [60] were administered daily intraperitoneal injections of atrazine (25 mg/kg BW/day dissolved in PBS) (Chem Service, Westchester PA) or dimethyl sulfoxide (DMSO) or as previously described [40]. Twenty-five mg/kg for atrazine is 4% of rat oral LD50 [41] and 50% of NOAEL [42].

As previously described [32], the gestating female rats treated were designated as the F0 generation. F1- F3 generation control and atrazine lineages were housed in the same room and racks with lighting, food and water as previously described [40, 61, 62]. All experimental protocols for the procedures with rats were pre-approved by the Washington State University Animal Care and Use Committee (protocol IACUC # 6252). All methods were performed in accordance with the relevant guidelines and regulations.

### Tissue harvest and histology processing

Rats were euthanized at 12 months of age by CO_2_ inhalation and cervical dislocation for tissue harvest. Testis, prostate, ovary, kidney, and gonadal fat pads were fixed in Bouin’s solution (Sigma) followed by 70% ethanol, then processed for paraffin embedding and hematoxylin, and eosin (H & E) staining by standard procedures for histopathological examination. Paraffin five micron sections were processed, stained, and provided by Nationwide Histology, Spokane WA, USA.

### Histopathology examination and disease classification

The oversight of the pathology analysis involved the co-author, Dr. Eric Nilsson, DVM/PhD, with over 20 years of pathology analysis in rats [38, 63]. The Washington Animal Disease Diagnostic Laboratory (WADDL) at the Washington State University College of Veterinary Medicine has board certified veterinary pathologists and assisted in initially establishing the criteria for the pathology analyses and identifying parameters to assess [61]. WADDL performed full necropsies as required on animals that died prior to the time of scheduled sacrifice at one year, and performed tumor classifications in the current study.

Upon dissection a brief examination of abdominal and thoracic organs was performed to look for obvious abnormalities. The tissues evaluated histologically were selected from previous literature showing them to have pathology in transgenerational models [28, 38, 61, 64–70], with an emphasis on reproductive organs. Histopathology readers were trained to recognize the specific abnormalities evaluated for this study in rat testis, ventral prostate and kidney (see below). Two different readers initially evaluated the tissues. If there was disagreement on whether an animal’s tissue showed disease, then a third pathology reader was used. Readers were blinded to the exposure groups. A set of quality control (QC) slides was generated for each tissue and was read by each reader prior to evaluating any set of experimental slides. These QC slide results are monitored for reader accuracy and concordance. WADDL was consulted when any questions developed. Previous studies by the laboratory help confirm and validate the pathology analysis [28, 38, 61, 64–70].

As previously described [21], testis histopathology criteria included the presence of vacuoles in the seminiferous tubules, azoospermic atretic seminiferous tubules, and ‘other’ abnormalities including sloughed spermatogenic cells in the center of the tubule and a lack of a tubule lumen. As previously described [63, 71], prostate histopathology criteria included the presence of vacuoles in the glandular epithelium, atrophic glandular epithelium and hyperplasia of prostatic gland epithelium (S2 Fig). Kidney histopathology criteria included reduced size of glomerulus, thickened Bowman’s capsule, and the presence of proteinaceous fluid-filled cysts >50 μm in diameter (S2 Fig). A cut-off was established to declare a tissue ‘diseased’ based on the mean number of histopathological abnormalities plus two standard deviations from the mean of control group tissues, as assessed by each of the individual readers. This number (i.e. greater than two standard deviations) was used to classify rats into those with and without testis, prostate, or kidney disease in the F3 generation lineage. A rat tissue section was finally declared ‘diseased’ only when at least two of the three readers marked the same tissue section ‘diseased’.

Lean phenotype was assessed with a decrease in adipocyte size (area), body mass index (BMI) and abdominal adiposity, as previously described [65, 69, 72–74]. BMI was calculated with weight (g) / length (cm)^2^ with the length of the animal measured from the nose to the base of the tail. Gonadal fat pad slides were imaged using a Nikon Eclipse E800 microscope (10x) with an AVT Prosilica GE1050C Color GigE camera. Five field of view image captures were taken per slide in varying parts of the fat pad. Adipocyte size was measured converting pixels into microns using Adiposoft [75]. Measurements of the 20 largest cells from each image for a total of 100 were averaged as hypertrophic cells are the most metabolically relevant and susceptible to cell death [76]. Obesity and lean phenotypes were determined utilizing the mean of the control population males and females, and a cut-off of 1.5 standard deviations above and below the mean.

#### Disease groups for biomarker analysis

The individual animals are listed and a (+) indicates presence of disease and (-) absence of disease for the current F3 generation atrazine lineage male pathology (Table 1). The F3 generation control lineage male pathology is listed in S1 Table. Table 1 shows which individual animals were used in each disease group. The animals within the treated lineage which exhibited no disease served as the “no disease” or control set in the biomarker analysis. These are the highlighted “0”s in the “Total Disease” column. For each disease, only animals exhibiting a single disease were placed in that disease group for the biomarker analysis, indicated by a highlighted “+” in the specific disease column. Any individual animals showing multiple diseases were included in the “multiple” disease category, indicated by a highlighted “+” in the “Multiple Disease” column. Due to low prevalence of disease in the control animal groups, these animals were not used in the identification of epigenetic biomarkers.

### Sperm epigenetic analysis

#### Epididymal sperm collection and DNA isolation

The protocol is described in detail in reference [32]. Briefly, the epididymis was dissected free of fat and connective tissue, then, after cutting open the cauda, placed into 6 ml of phosphate buffer saline (PBS) for 20 minutes at room temperature. Further incubation at 4°C will immobilize the sperm. The tissue was then minced, the released sperm pelleted at 4°C 3,000 x *g* for 10 min, then resuspended in NIM buffer and stored at -80°C for further processing.

An appropriate amount of rat sperm suspension was used for DNA extraction. Previous studies have shown mammalian sperm heads are resistant to sonication unlike somatic cells [77, 78]. Somatic cells and debris were therefore removed by brief sonication (Fisher Sonic Dismembrator, model 300, power 25), then centrifugation and washing 1–2 times in 1xPBS. The resulting pellet was resuspended in 820 μL DNA extraction buffer and 80 μl 0.1M DTT added, then incubated at 65°C for 15 minutes. 80 μl proteinase K (20 mg/ml) was added and the sample was incubated at 55°C for 2–4 hours under constant rotation. Protein was removed by addition of protein precipitation solution (300 μl, Promega A795A), incubation for 15 min on ice, then centrifugation at 13,500 rpm for 30 minutes at 4°C. One ml of the supernatant was precipitated with 2 μl of Glycoblue (Invitrogen, AM9516) and 1 ml of cold 100% isopropanol. After incubation, the sample was spun at 13,500 x g for 30 min at 4°C, then washed with 70% cold ethanol. The pellet was air-dried for about 5 minutes then resuspended in 100 μl of nuclease free water. For all generations, equal amounts of DNA from each individual’s sample was used to produce 6 different DNA pools per lineage and the pooled DNA used for methylated DNA immunoprecipitation (MeDIP).

#### Methylated DNA Immunoprecipitation (MeDIP)

The protocol is described in detail in reference [32]. Genomic DNA was sonicated and run on 1.5% agarose gel for fragment size verification. The sonicated DNA was then diluted with TE buffer to 400 μl, then heat-denatured for 10 min at 95°C, and immediately cooled on ice for 10 min to create single-stranded DNA fragments. Then 100 μl of 5X IP buffer and 5 μg of antibody (monoclonal mouse anti 5-methyl cytidine; Diagenode #C15200006) were added, and the mixture was incubated overnight on a rotator at 4°C. The following day magnetic beads (Dynabeads M-280 Sheep anti-Mouse IgG; Life Technologies 11201D) were pre-washed per manufacturer’s instructions, and 50 μl of beads were added to the 500 μl of DNA-antibody mixture from the overnight incubation, then incubated for 2h on a rotator at 4°C. After this incubation, the samples were washed three times with 1X IP buffer using a magnetic rack. The washed samples were then resuspended in 250 μl digestion buffer (5 mM Tris PH 8, 10 mM EDTA, 0.5% SDS) with 3.5 μl Proteinase K (20 mg/ml), and incubated for 2–3 hours on a rotator at 55°. DNA clean-up was performed using a Phenol-Chloroform-Isoamyalcohol extraction, and the supernatant precipitated with 2 μl of Glycoblue (20 mg/ml), 20 μl of 5M NaCl and 500 μl ethanol in -20°C freezer for one to several hours. The DNA precipitate was pelleted, washed with 70% ethanol, then dried and resuspended in 20 μl H_2_O or TE. DNA concentration was measured in Qubit (Life Technologies) with the ssDNA kit (Molecular Probes Q10212).

#### Chromatin Immunoprecipitation (ChIP)

As previously described in [79], Histone chromatin immunoprecipitation with genomic DNA was performed with a procedure previously described [35]. Individual rat sperm collections were generated, and the sperm counts were determined for each individual. Equal numbers of sperm were added from each individual for a total of 1.5 million sperm. To remove any somatic cell contamination sperm samples from each animal were sonicated for 10 seconds using a Sonic Dismembrator Model 300 (Thermo Scientific Fisher, USA) then centrifuged 1800x*g* for 5 min at 4°C then resuspended and counted individually on a Neubauer counting chamber (Propper manufacturing Co., Inc., New York, USA) prior to pooling. The sperm pools were reconstituted up to 1 ml with PBS (phosphate buffered saline). To reduce disulfide bonds, 50 μl of 1 M DTT was added to each pool and the pools were then incubated for 2 hours at room temperature under constant rotation. To quench any residual DTT (dithiothreitol, Fisher Scientific, NY USA) in the reaction, 120 μl of fresh 1 M NEM (N-Ethylmaleimide, Thermo Scientific, Rockford, USA) was then added and the samples were incubated for 30 min at room temperature under constant rotation. The sperm cells were pelleted at 450x*g* for 5 min at room temperature and the supernatant was discarded. Pellets were resuspended in PBS and then spun again at 450x*g* for 5 min at room temperature. The supernatant was discarded and resuspended in 130 μl of complete buffer supplemented with tergitol 0.5% and DOC 1%. The samples were then sonicated using the Covaris M220. Covaris was set to a 10 min “Chromatin shearing” program and the program was run for each tube in the experiment.

After the Covaris sonication, 10 μl of each sample was run on a 1.5% agarose gel to verify fragment size. Samples were then centrifuged at 12,500x*g* for 10 min at room temperature. The supernatant was transferred to a fresh microfuge tube. 65 μl of protease inhibitor cocktail (1 tablet dissolved in 500 μl, 20 × concentrated) (Roche, cat. no. 11 873 580 001) were added in each sample as well as 3 μl of antibody (anti-histone H3 pan-monoclonal antibody, cat no. 05–928, or anti-trimethyl-histone H3 (Lys27) polyclonal antibody, cat no. 07–449, both with broad spectrum species specifically form Millipore Corp, Temecula CA USA). The DNA-antibody mixture was incubated overnight on a rotator at 4°C. The following day, magnetic beads (ChIP-Grade protein G magnetic beads, Cell Signaling 9006) were pre-washed as follows: the beads were resuspended in the vial, then 30 μl per sample was transferred to a microfuge tube. The same volume of Washing Buffer (at least 1 ml) was added and the bead sample was resuspended. The tube was then placed into a magnetic rack for 1–2 min and the supernatant was discarded. The tube was removed from the magnetic rack and the beads were washed once. The washed beads were resuspended in the same volume of IP buffer as the initial volume of beads. 30 μl of beads were added to each DNA-antibody mixture from the overnight incubation, then incubated for 2 h on a rotator at 4°C. After the incubation, the bead-antibody-DNA complex was washed three times with IP buffer as follows: the tube was placed into a magnetic rack for 1–2 min and the supernatant was discarded, then washed with IP buffer 3 times. The washed bead-antibody-DNA solution was then resuspended in 300 μl of digestion buffer (1 M Tris HCI, pH 8.0, 0.5 M EDTA, 10% SDS) and 3 μl proteinase K (20 g/ml). The sample was incubated for 3 h on a rotator at 56°C. After incubation the samples were extracted with Phenol-Chloroform-Isoamylalcohol and precipitated with 2 μl of Glycoblue (20 mg/ml), a one-tenth volume of 3 M sodium acetate and two volumes of ethanol overnight at −20°C.

The precipitate was centrifuged at 18,000x*g* for 30 min at 4°C and the supernatant was removed, while not disturbing the pellet. The pellet was washed with 500 μl cold 70% ethanol, then centrifuged again at 18,000x*g* for 10 min at 4°C and the supernatant was discarded. The tube was spun briefly to collect residual ethanol to bottom of tube and as much liquid as possible was removed with a gel loading tip. Pellet was air-dried at RT until it looked dry (about 5 min), then resuspended in 20 μl H20. DNA concentration was measured in the Qubit (Life Technologies) with the BR dsDNA kit (Molecular Probes Q32853).

#### MeDIP-Seq/ ChIP-Seq analysis

MeDIP DNA was used to create libraries for next generation sequencing (NGS) using the NEBNext Ultra RNA Library Prep Kit for Illumina (San Diego, CA) starting at step 1.4 of the manufacturer’s protocol to generate double stranded DNA from the single-stranded DNA resulting from MeDIP. After this step the MeDIP DNA, and starting with the ChIP DNA, the manufacturer’s protocol was followed indexing each sample individually with NEBNext Multiplex Oligos for Illumina. The WSU Spokane Genomics Core sequenced the samples on the Illumina HiSeq 2500 at PE50, with a read size of approximately 50 bp and approximately 20 million reads per pool. Ten libraries were run in one lane.

#### Statistics and bioinformatics

The DMR and DHR identification and annotation methods follow those presented in previous published papers [38, 80]. Data quality was assessed using the FastQC program (https://www.bioinformatics.babraham.ac.uk/projects/fastqc/), and reads were cleaned and filtered to remove adapters and low quality bases using Trimmomatic [81]. The reads for each MeDIP and ChIP sample were mapped to the Rnor 6.0 rat genome using Bowtie2 [82] with default parameter options. The mapped read files were then converted to sorted BAM files using SAMtools [75]. The MEDIPS R package [76] was used to calculate differential coverage between control and exposure sample groups. The reference genome was broken into 1000 bp windows. Only genomic windows with at least an average of 10 reads per sample were kept for subsequent analysis. The edgeR p-value [83] was used to determine the relative difference between the two groups for each genomic window. Windows with an edgeR p-value less than an arbitrarily selected threshold were considered DMRs or DHRs. The DMR/DHR edges were extended until no genomic window with an edgeR p-value less than 0.1 remained within 1000 bp of the DMR/DHR.

DMRs and DHRs were annotated using the biomaRt R package [84] to access the Ensembl database [85]. The genes that associated with DMRs/DHRs were then input into the KEGG pathway search [86, 87] to identify associated pathways. The DMR/DHR associated genes were then automatically sorted into functional groups using information provided by the DAVID [88] and Panther [89] databases incorporated into an internal curated database (www.skinner.wsu.edu under genomic data). All molecular data has been deposited into the public database at NCBI (GEO # GSE156530) and R code computational tools available at GitHub (https://github.com/skinnerlab/MeDIP-seq) and www.skinner.wsu.edu.

## Supporting information

S1 Fig DMR genomic features. The number of DMRs at different CpG densities. All DMRs at a p-value threshold of p<1e-04 are shown. **(A)** Lean phenotype DMR CpG density; **(B)** Lean phenotype DMR length; **(C)** Kidney disease DMR CpG density; **(D)** Kidney disease DMR length; **(E)** Testis disease DMR CpG density; **(F)** Testis disease DMR length; **(G)** Late puberty DMR CpG density; **(H)** Late puberty DMR length; **(I)** Multiple disease DMR CpG density; and **(J)** Multiple disease DMR length.
(PDF)

S2 Fig DMR principal component analysis (PCA). The first two principal components used. The underlying data is the RPKM read depth for DMR associated genomic windows. **(A)** Lean phenotype DMRs PCA; **(B)** Kidney disease DMRs PCA; **(C)** Testis disease DMRs PCA; **(D)** Late puberty DMRs PCA; and **(E)** Multiple disease DMRs PCA.
(PDF)

S3 Fig DHR genomic features. The number of DHRs at different CpG densities. All DHRs at a p-value threshold of 1e-04 are shown. **(A)** Atrazine versus control DHR CpG density; **(B)** Atrazine versus control DHR lengths; **(C)** Lean phenotype DHR CpG density; **(D)** Lean phenotype DHR length; **(E)** Kidney disease DHR CpG density; **(F)** Kidney disease DHR length; **(G)** Testis disease DHR CpG density; **(H)** Testis disease DHR length; **(I)** Late puberty DHR CpG density; **(J)** Late puberty DHR length; **(K)** Multiple disease DHR CpG density; and **(L)** Multiple disease DHR length.
(PDF)

S4 Fig DHR principal component analysis (PCA). The first two principal components used. The underlying data is the RPKM read depth for DHR genomic windows. **(A)** Lean phenotype DHRs PCA; **(B)** Kidney disease DHRs PCA; **(C)** Testes disease DHRs PCA; **(D)** Late puberty DHRs PCA; and **(E)** Multiple disease DHRs PCA.
(PDF)

S5 Fig Transgenerational atrazine versus control lineage DHRs. **(A)** DHRs identified at various edgeR p-value thresholds for All Window (1 kb) and Multiple Window (≥2 nearby 1 kb) with the DHR numbers presented. The DHRs at p<1e-04 were selected for subsequent analysis. **(B)** DHR chromosomal locations with red arrowhead indicating location of DHRs and black box DHR clusters and different chromosome numbers versus chromosome size (megabase). **(C)** DHR CpG density for number of DHRs per number of CpG/100 bp. **(D)** DHR length with number of DHR versus DHR length (kb). **(E)** Principal component analysis (PCA) of DHR read depths for principal components 1 and 2 for control and autism DHRs.
(PDF)

S6 Fig Venn diagram DMR and DHR overlaps. **(A)** DMR overlaps with disease DMRs at p<1e-04 and all different diseases DMR overlaps at p<0.05 (All). **(B)** DHR overlaps at p<1e-04 and all different diseases DHR overlaps at p<0.05 (All).
(PDF)

S7 Fig Epimutation associated KEGG gene pathways. **(A)** DMR associated gene pathways for each disease DMR data set. **(B)** DHR associated gene pathways for each disease DHR data set. The pathway and number of associated DMR or DHR in brackets indicated.
(PDF)

S1 Table Individual animal pathology. F3 generation control lineage males pathology. The individual animals for the atrazine control lineage males are listed and a (+) indicates presence of disease and (-) absence of disease. The statistical increase in the atrazine lineage males was determined with a comparison of these control lineage pathology data.
(PDF)

S2 Table DMR site list lean p<1e-04. DMR name, chromosome, start, stop, length, number signature windows, minimum p-value, max log-fold change, CpG number, CpG density, gene annotation, and gene category are presented.
(PDF)

S3 Table DMR site list kidney p<1e-04. DMR name, chromosome, start, stop, length, number signature windows, minimum p-value, max log-fold change, CpG number, CpG density, gene annotation, and gene category are presented.
(PDF)

S4 Table DMR site list testis p<1e-04. DMR name, chromosome, start, stop, length, number signature windows, minimum p-value, max log-fold change, CpG number, CpG density, gene annotation, and gene category are presented.
(PDF)

S5 Table DMR site list puberty p<1e-04. DMR name, chromosome, start, stop, length, number signature windows, minimum p-value, max log-fold change, CpG number, CpG density, gene annotation, and gene category are presented.
(PDF)

S6 Table DMR site list multiple p<1e-04. DMR name, chromosome, start, stop, length, number signature windows, minimum p-value, max log-fold change, CpG number, CpG density, gene annotation, and gene category are presented.
(PDF)

S7 Table DHR site list lean p<1e-05. DHR name, chromosome, start, stop, length, number signature windows, minimum p-value, max log-fold change, CpG number, CpG density, gene annotation, and gene category are presented.
(PDF)

S8 Table DHR site list kidney p<1e-04. DHR name, chromosome, start, stop, length, number signature windows, minimum p-value, max log-fold change, CpG number, CpG density, gene annotation, and gene category are presented.
(PDF)

S9 Table DHR site list testis p<1e-04. DHR name, chromosome, start, stop, length, number signature windows, minimum p-value, max log-fold change, CpG number, CpG density, gene annotation, and gene category are presented.
(PDF)

S10 Table DHR site list puberty p<1e-04. DHR name, chromosome, start, stop, length, number signature windows, minimum p-value, max log-fold change, CpG number, CpG density, gene annotation, and gene category are presented.
(PDF)

S11 Table DHR site list multiple p<1e-04. DHR name, chromosome, start, stop, length, number signature windows, minimum p-value, max log-fold change, CpG number, CpG density, gene annotation, and gene category are presented.
(PDF)

S12 Table Atrazine versus control F3 generation lineage sperm DHRs. DHR site list for atrazine versus control sperm DHRs at p<1e-04. DHR name, chromosome, start, stop, length, number signature windows, minimum p-value, max log-fold change, CpG number, CpG density, gene annotation, and gene category are presented.
(PDF)
