# Supplementary material for: Epigenome-wide association study for atrazine induced transgenerational DNA methylation and histone retention sperm epigenetic biomarkers for disease
Source: PLoS One. 2020 Dec 16;15(12):e0239380. doi: 10.1371/journal.pone.0239380 (PMC7743986; doi:10.1371/journal.pone.0239380)

Supplemental Figure S1 DMR Genomic Features

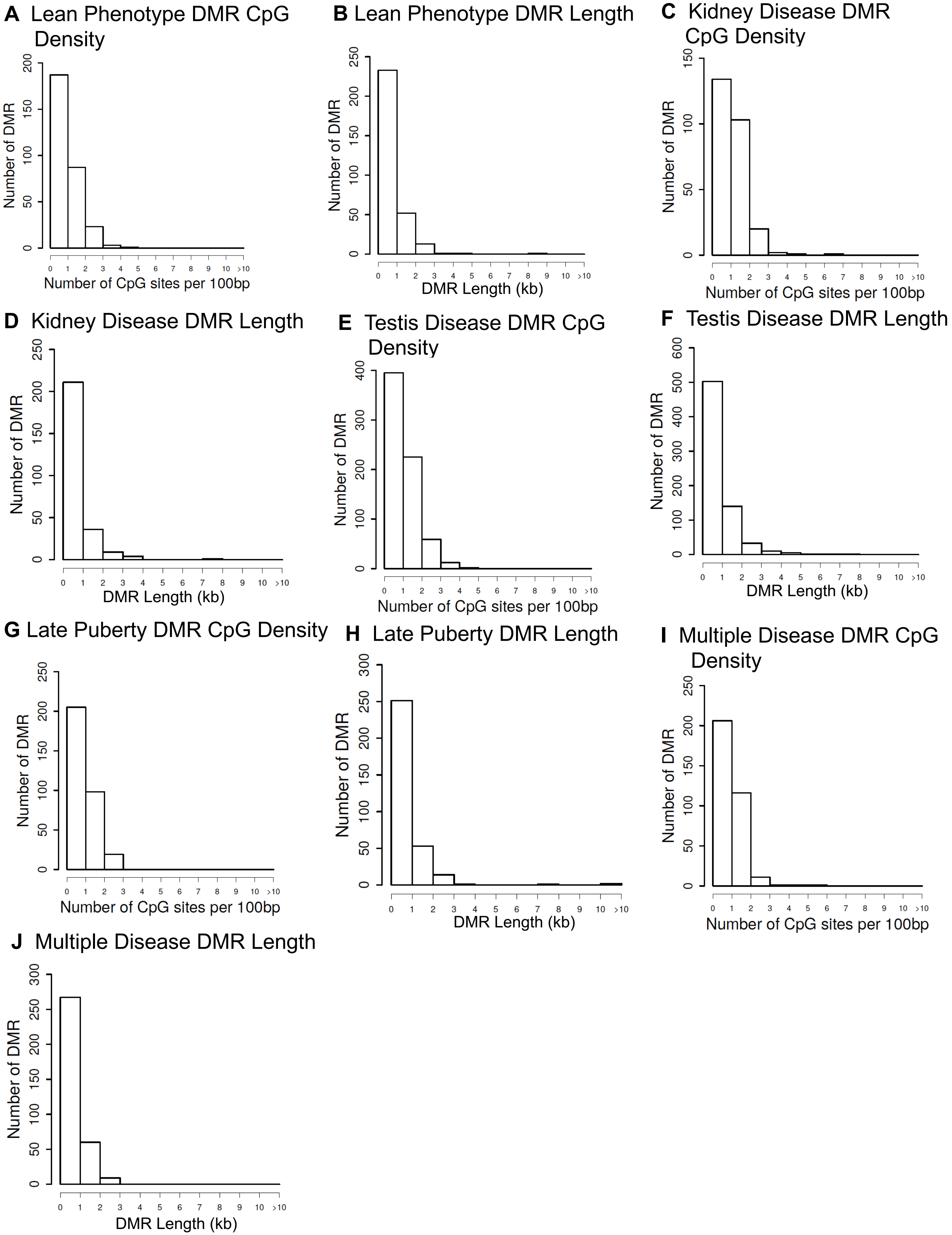

Supplement: S1 Fig — The number of DMRs at different CpG densities. All DMRs at a p-value threshold of p<1e-04 are shown. (A) Lean phenotype DMR CpG density; (B) Lean phenotype DMR length; (C) Kidney disease DMR CpG density; (D) Kidney disease DMR length; (E) Testis disease DMR CpG density; (F) Testis disease DMR length; (G) Late puberty DMR CpG density; (H) Late puberty DMR length; (I) Multiple disease DMR CpG density; and (J) Multiple disease DMR length. (PDF) [file pone.0239380.s001.pdf]
