# Supplementary material for: Epigenome-wide association study for atrazine induced transgenerational DNA methylation and histone retention sperm epigenetic biomarkers for disease
Source: PLoS One. 2020 Dec 16;15(12):e0239380. doi: 10.1371/journal.pone.0239380 (PMC7743986; doi:10.1371/journal.pone.0239380)

Supplemental Figure S2 (Color) DMR Principal Component Analysis

A Lean Phenotype DMR biomarkers

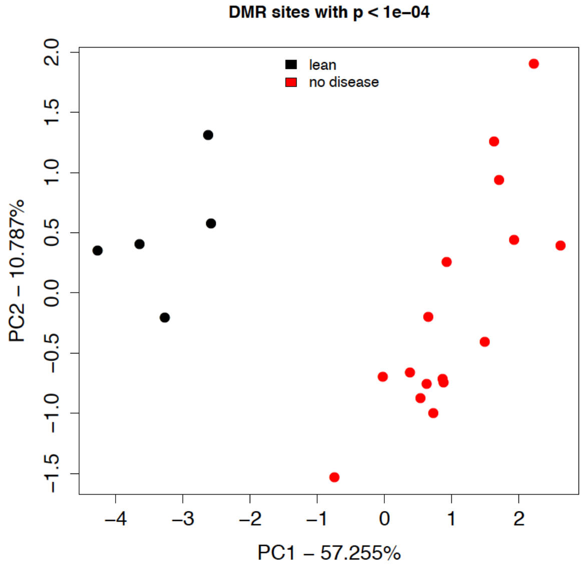

B Kidney disease DMR biomarkers

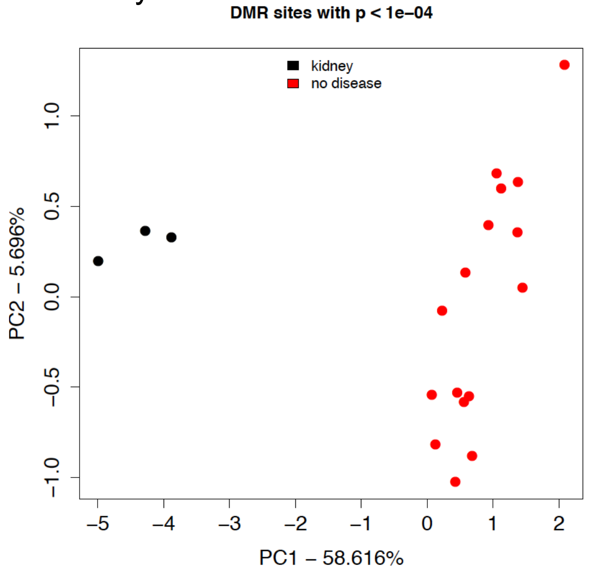

C Testis disease DMR biomarkers

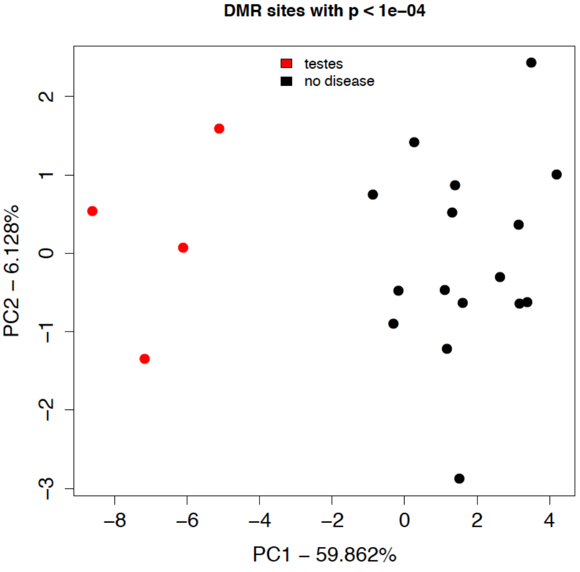

D Late Puberty DMR biomarkers

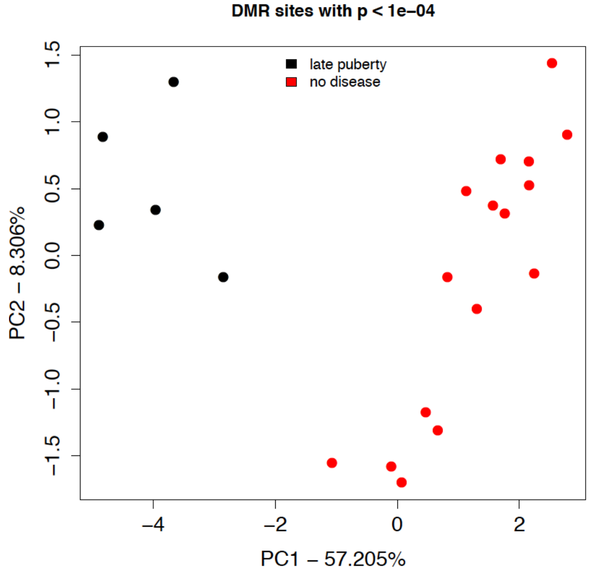

E Multiple disease DMR biomarkers

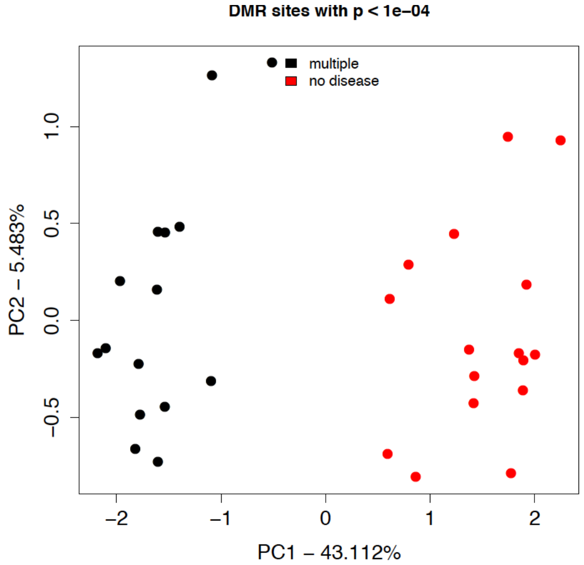

Supplement: S2 Fig — The first two principal components used. The underlying data is the RPKM read depth for DMR associated genomic windows. (A) Lean phenotype DMRs PCA; (B) Kidney disease DMRs PCA; (C) Testis disease DMRs PCA; (D) Late puberty DMRs PCA; and (E) Multiple disease DMRs PCA. (PDF) [file pone.0239380.s002.pdf]
