# Supplementary material for: Epigenome-wide association study for atrazine induced transgenerational DNA methylation and histone retention sperm epigenetic biomarkers for disease
Source: PLoS One. 2020 Dec 16;15(12):e0239380. doi: 10.1371/journal.pone.0239380 (PMC7743986; doi:10.1371/journal.pone.0239380)

Supplemental Figure S3 DHR Genomic Features

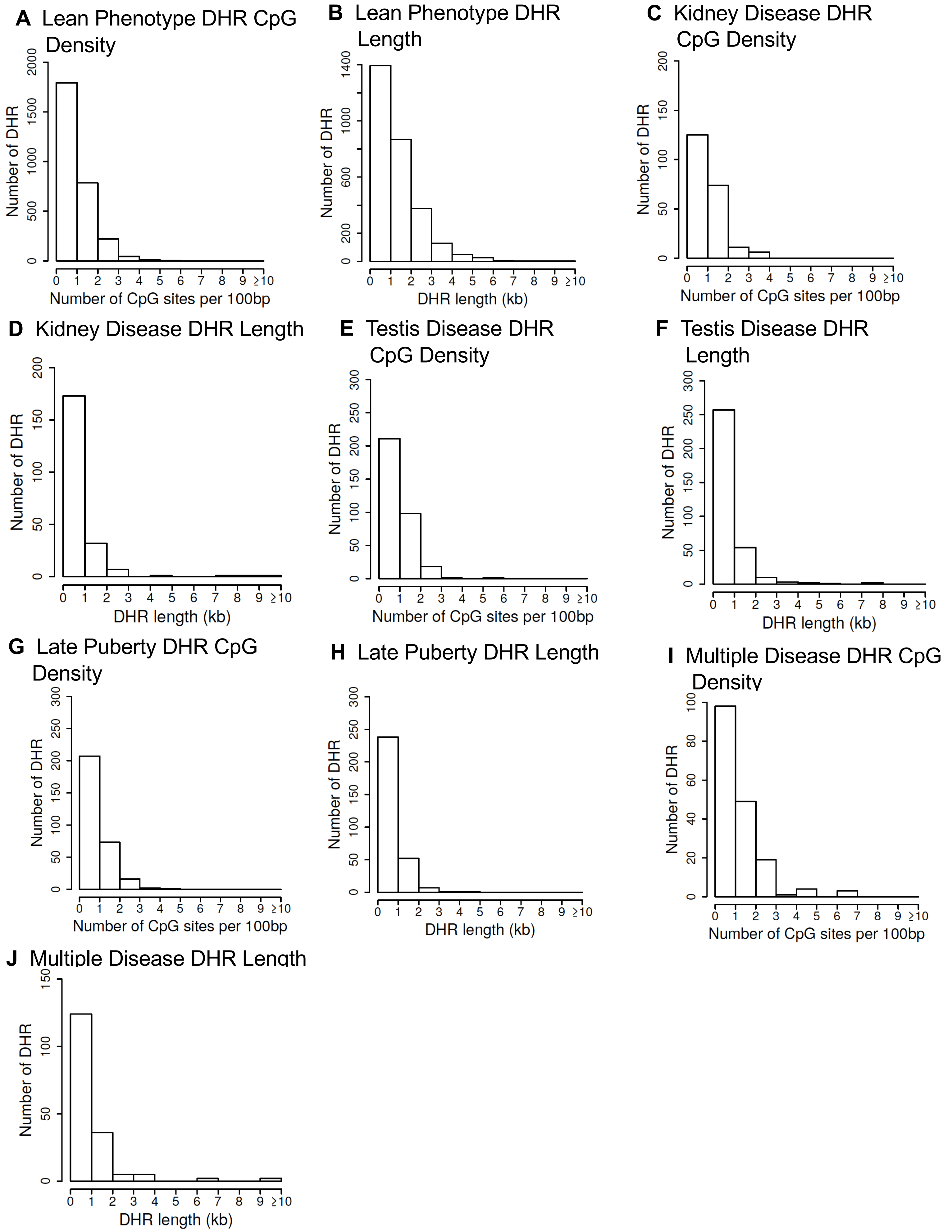

Supplement: S3 Fig — The number of DHRs at different CpG densities. All DHRs at a p-value threshold of 1e-04 are shown. (A) Atrazine versus control DHR CpG density; (B) Atrazine versus control DHR lengths; (C) Lean phenotype DHR CpG density; (D) Lean phenotype DHR length; (E) Kidney disease DHR CpG density; (F) Kidney disease DHR length; (G) Testis disease DHR CpG density; (H) Testis disease DHR length; (I) Late puberty DHR CpG density; (J) Late puberty DHR length; (K) Multiple disease DHR CpG density; and (L) Multiple disease DHR length. (PDF) [file pone.0239380.s003.pdf]
