# Supplementary material for: Epigenome-wide association study for atrazine induced transgenerational DNA methylation and histone retention sperm epigenetic biomarkers for disease
Source: PLoS One. 2020 Dec 16;15(12):e0239380. doi: 10.1371/journal.pone.0239380 (PMC7743986; doi:10.1371/journal.pone.0239380)

Supplemental Figure S4 (Color) DHR Principal Component Analysis

A Lean phenotype DHR biomarkers

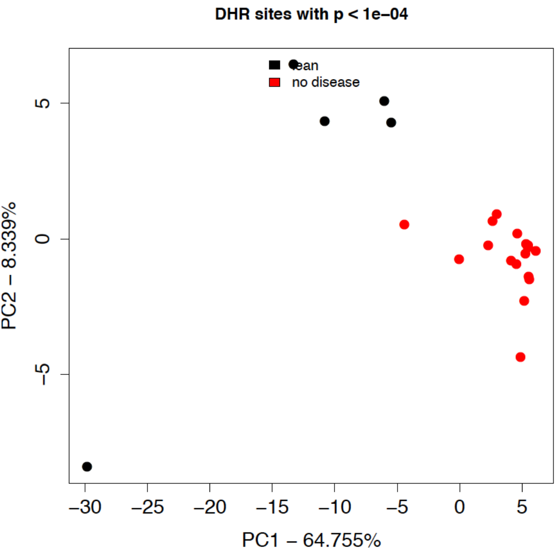

B Kidney disease DHR biomarkers

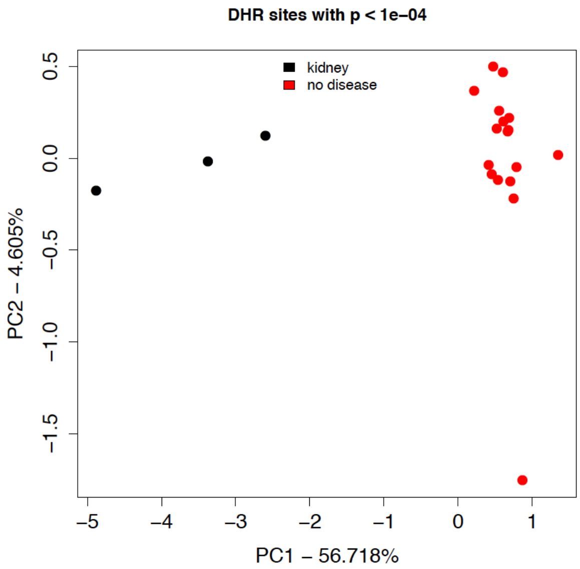

C Testis disease DHR biomarkers

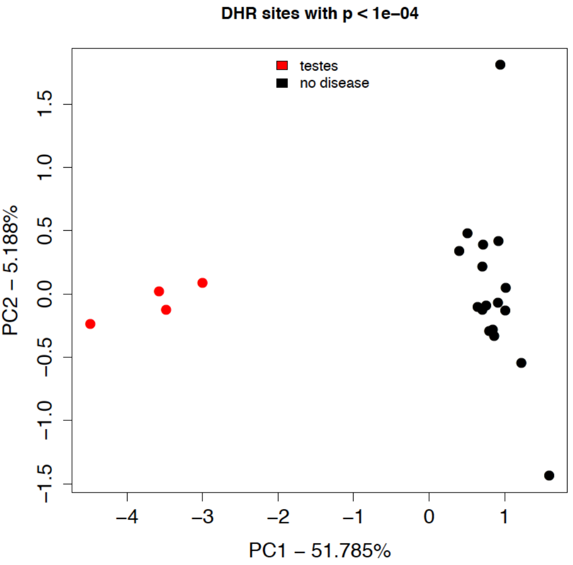

D Late puberty DHR biomarkers

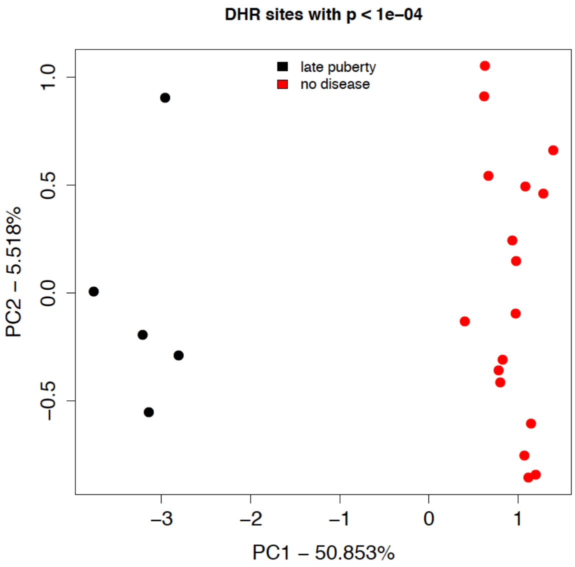

E Multiple disease DHR biomarkers

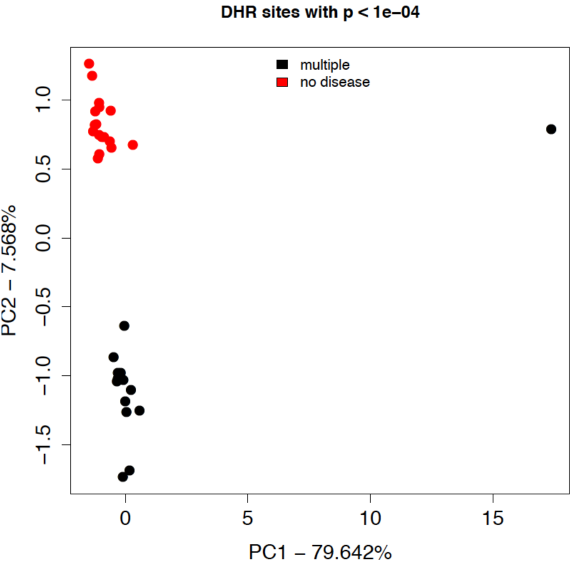

Supplement: S4 Fig — The first two principal components used. The underlying data is the RPKM read depth for DHR genomic windows. (A) Lean phenotype DHRs PCA; (B) Kidney disease DHRs PCA; (C) Testes disease DHRs PCA; (D) Late puberty DHRs PCA; and (E) Multiple disease DHRs PCA. (PDF) [file pone.0239380.s004.pdf]
