# Supplementary material for: Epigenome-wide association study for atrazine induced transgenerational DNA methylation and histone retention sperm epigenetic biomarkers for disease
Source: PLoS One. 2020 Dec 16;15(12):e0239380. doi: 10.1371/journal.pone.0239380 (PMC7743986; doi:10.1371/journal.pone.0239380)

Supplemental Figure S5 (Color) Transgenerational Atrazine versus Control Lineage DHRs

A Atrazine versus Control DHRs

p-value	All Window	Multiple Window
0.001	5616	176
**1e-04**	**786**	**18**
1e-05	130	7
1e-06	28	2
1e-07	2	0

Significant windows	1	2	3	≥5
Number of DMR	768	11	3	4

B DHR Chromosomal Locations
Atrazine versus Control DHR

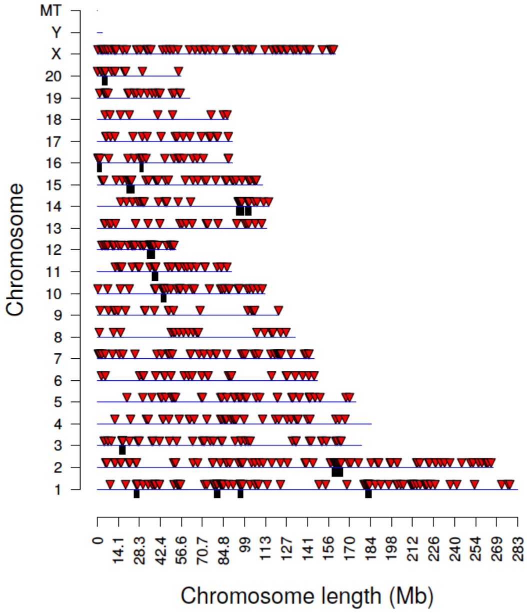

C DHR CpG Density

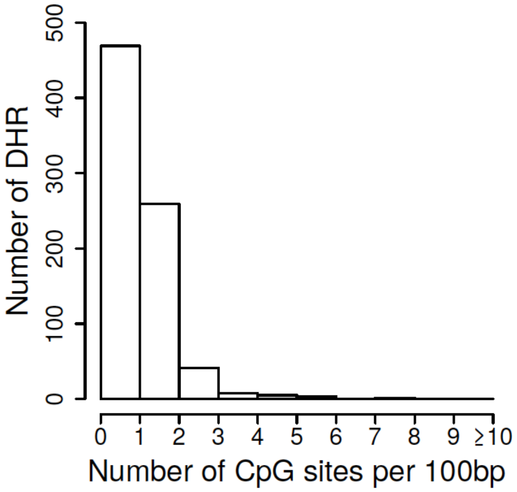

D DHR Length

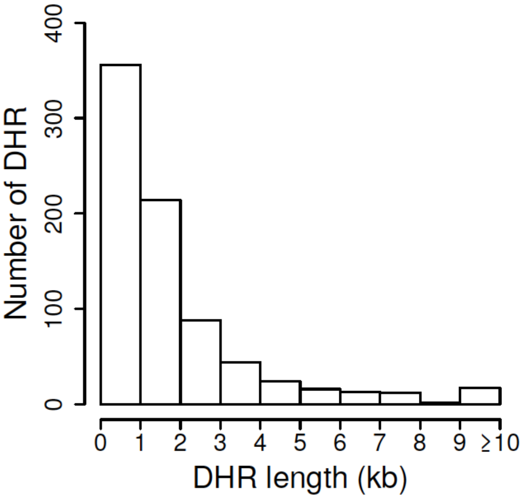

E Atrazine versus Control DHRs PCA

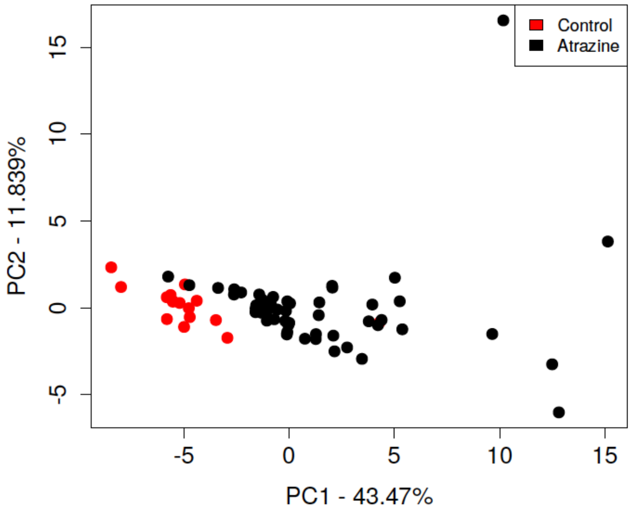

Supplement: S5 Fig — (A) DHRs identified at various edgeR p-value thresholds for All Window (1 kb) and Multiple Window (≥2 nearby 1 kb) with the DHR numbers presented. The DHRs at p<1e-04 were selected for subsequent analysis. (B) DHR chromosomal locations with red arrowhead indicating location of DHRs and black box DHR clusters and different chromosome numbers versus chromosome size (megabase). (C) DHR CpG density for number of DHRs per number of CpG/100 bp. (D) DHR length with number of DHR versus DHR length (kb). (E) Principal component analysis (PCA) of DHR read depths for principal components 1 and 2 for control and autism DHRs. (PDF) [file pone.0239380.s005.pdf]
