# Supplementary material for: Epigenome-wide association study for atrazine induced transgenerational DNA methylation and histone retention sperm epigenetic biomarkers for disease
Source: PLoS One. 2020 Dec 16;15(12):e0239380. doi: 10.1371/journal.pone.0239380 (PMC7743986; doi:10.1371/journal.pone.0239380)

Supplemental Figure S6

A DMR Venn Diagram Overlaps with p<0.05 Overlaps

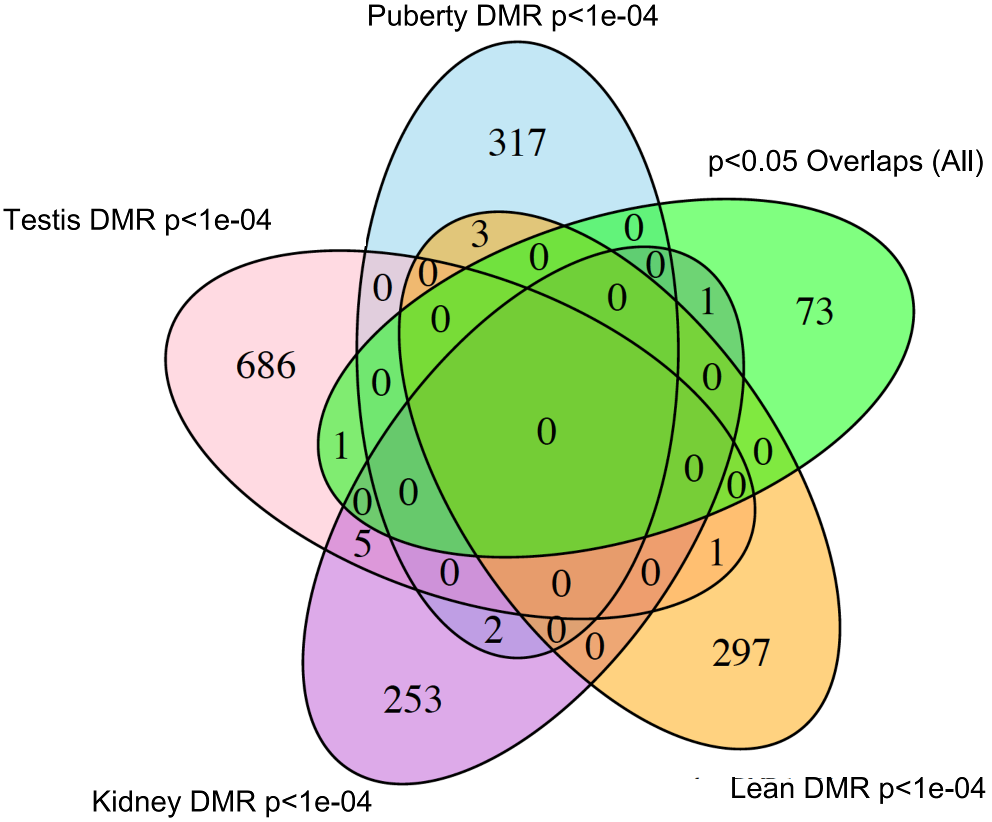

B DHR Venn Diagram Overlaps with p<0.05 Overlaps

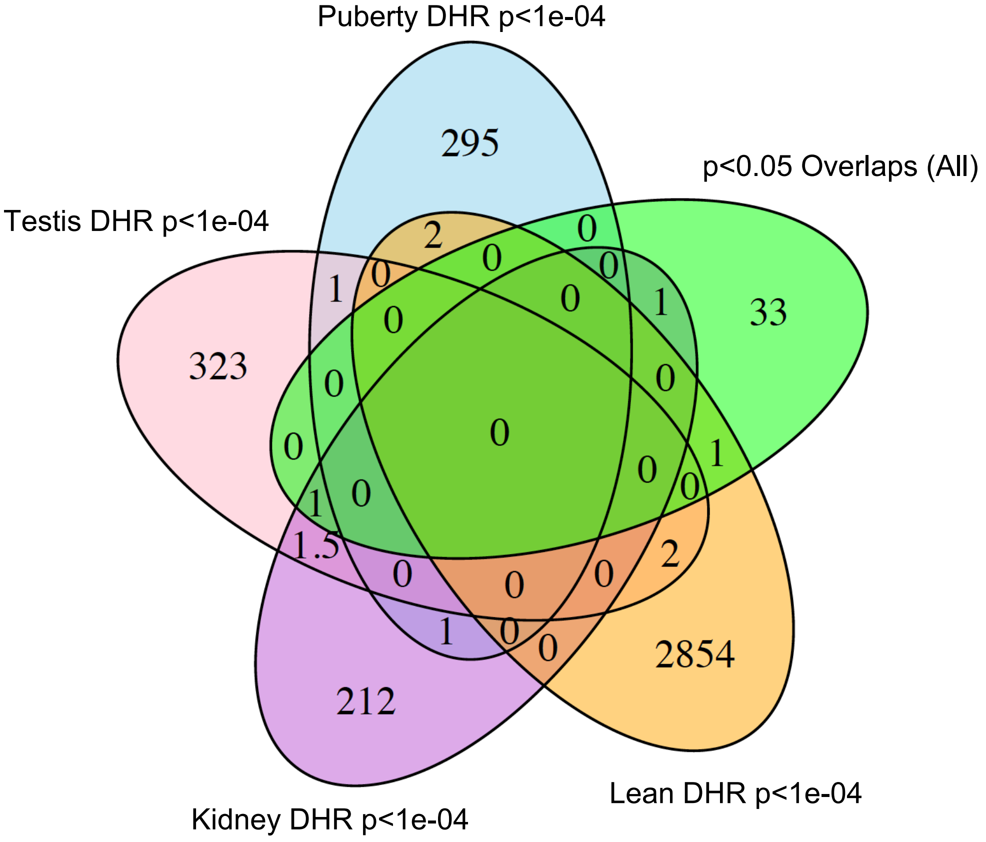

Supplement: S6 Fig — (A) DMR overlaps with disease DMRs at p<1e-04 and all different diseases DMR overlaps at p<0.05 (All). (B) DHR overlaps at p<1e-04 and all different diseases DHR overlaps at p<0.05 (All). (PDF) [file pone.0239380.s006.pdf]
