# Supplementary material for: Epigenome-wide association study for atrazine induced transgenerational DNA methylation and histone retention sperm epigenetic biomarkers for disease
Source: PLoS One. 2020 Dec 16;15(12):e0239380. doi: 10.1371/journal.pone.0239380 (PMC7743986; doi:10.1371/journal.pone.0239380)
Supplement: S7 Fig — (A) DMR associated gene pathways for each disease DMR data set. (B) DHR associated gene pathways for each disease DHR data set. The pathway and number of associated DMR or DHR in brackets indicated. (PDF) [file pone.0239380.s007.pdf]

Supplemental Figure S7

## Gene Pathways

### (A) DMR Associated Gene Pathways

**Atrazine DMRs 1e-07**
rno01100 Metabolic pathways (52)
rno05200 Pathways in cancer (19)
rno05165 Human papillomavirus infection (19)
rno04144 Endocytosis (18)
rno04360 Axon guidance (15)

**Lean DMRs 1e-04**
rno01100 Metabolic pathways (12)
rno04724 Glutamatergic synapse (5)
rno04740 Olfactory transduction (5)
rno04514 Cell adhesion molecules (CAMs) (4)
rno04934 Cushing syndrome (3)

**Kidney Disease DMRs 1e-04**
rno01100 Metabolic pathways (11)
rno05170 Human immunodeficiency virus 1 infection (6)
rno04723 Retrograde endocannabinoid signaling (4)
rno05169 Epstein-Barr virus infection (4)
rno05161 Hepatitis B (4)

**Testis Disease DMRs 1e-04**
rno05200 Pathways in cancer (17)
rno01100 Metabolic pathways (17)
rno04144 Endocytosis (13)
rno05165 Human papillomavirus infection (11)
rno04740 Olfactory transduction (11)

**Puberty DMRs 1e-04**
rno01100 Metabolic pathways (15)
rno04072 Phospholipase D signaling pathway (5)
rno04024 cAMP signaling pathway (5)
rno05032 Morphine addiction (4)
rno04530 Tight junction (4)

**Multiple Disease DMRs 1e-04**
rno01100 Metabolic pathways (9)
rno04915 Estrogen signaling pathway (8)
rno04080 Neuroactive ligand-receptor interaction (7)
rno04010 MAPK signaling pathway (6)
rno04724 Glutamatergic synapse (6)

### (B) DHR Associated Gene Pathways

**Atrazine DHRs 1e-04**
rno01100 Metabolic pathways (24)
rno05165 Human papillomavirus infection (17)
rno05200 Pathways in cancer (16)
rno05167 Kaposi sarcoma-associated herpesvirus infection (16)
rno05169 Epstein-Barr virus infection (15)

**Lean DHRs 1e-05**
rno01100 Metabolic pathways (22)
rno04740 Olfactory transduction (11)
rno05200 Pathways in cancer (10)
rno04360 Axon guidance (9)
rno04723 Retrograde endocannabinoid signaling (8)

**Kidney Disease DHRs 1e-04**
rno01100 Metabolic pathways (8)
rno05200 Pathways in cancer (5)
rno05203 Viral carcinogenesis (4)
rno04151 PI3K-Akt signaling pathway (4)
rno05161 Hepatitis B (3)

**Testis Disease DHRs 1e-04**
rno01100 Metabolic pathways (11)
rno04360 Axon guidance (8)
rno04724 Glutamatergic synapse (6)
rno05200 Pathways in cancer (6)
rno04725 Cholinergic synapse (6)

**Puberty DHRs 1e-04**
rno01100 Metabolic pathways (9)
rno04010 MAPK signaling pathway (4)
rno04722 Neurotrophin signaling pathway (4)
rno04080 Neuroactive ligand-receptor interaction (3)
rno04740 Olfactory transduction (3)

**Multiple Disease DHRs 1e-04**
rno05165 Human papillomavirus infection (5)
rno05202 Transcriptional misregulation in cancer (4)
rno01100 Metabolic pathways (4)
rno04514 Cell adhesion molecules (CAMs) (4)
rno04380 Osteoclast differentiation (3)
