# Supplementary material for: Epigenome-wide association study for atrazine induced transgenerational DNA methylation and histone retention sperm epigenetic biomarkers for disease
Source: PLoS One. 2020 Dec 16;15(12):e0239380. doi: 10.1371/journal.pone.0239380 (PMC7743986; doi:10.1371/journal.pone.0239380)
Supplement: S1 Table — F3 generation control lineage males pathology. The individual animals for the atrazine control lineage males are listed and a (+) indicates presence of disease and (-) absence of disease. The statistical increase in the atrazine lineage males was determined with a comparison of these control lineage pathology data. (PDF) [file pone.0239380.s008.pdf]

Supplemental Table S1 Individual Animal Pathology
F3 Generation Control Lineage Males

		Puberty		Testes	Prostate	Kidney	Lean	Obese	Tumor	Multiple	Total
	Sample ID	Early	Late								
CM1	**M15**	-	-	-	-	-	-	+	-	-	1
CM2	**M13**	-	-	-	-	-	-	-	-	-	
CM3	**M14**	-	-	-	-	-	-	-	-	-	
CM4	**M18**	-	-		-	-	-	-	-	-	
CM5	**M16**	-	-	-	-	-	-	-	-	-	
CM6	**M17**	-	-		-	+	+	-	-	+	2
CM7		-	-	-	-	-	+	-	-	-	1
CM8		-	-	-	-	-	-	-	-	-	
CM9		-	-	-	-	-	+	-	-	-	1
CM10		-	-	-	-	-	+	-	-	-	1
CM11		-	-	-	-	-		-	-	-	
CM12		-	-	+	+	-	-	-	-	+	2
CM13		-	-	-	-	-	-	-	-	-	
CM14		-	-	-	-	-	-	-	-	-	
CM16	**M1**	-	-	-	-	-	-	-	-	-	
GCM17	**M2**	-	-					-	-	-	
CM17	**M3**	-	+	-	-	-	-	+	-	+	2
CM18	**M4**	-	+	-	-	-	-	-	-	-	1
CM19	**M5**	-	-	-	-	-	-	+	-	-	1
CM20		-	-	-	-	-	-	-	-	-	
CM21	**M6**	-	-	-	-	-	-	-	-	-	
CM22	**M7**	-	-	-	-	+	-	-	-	-	1
CM23	**M10**	-	-	-	-	-	-	-	-	-	
CM24	**M8**	-	-	-	-	-	-	-	-	-	
CM25	**M9**	-	-	-	-	-	-	-	-	-	
CM26	**M11**	-	-	-	-	-	-	+	-	-	1
CM27	**M12**	-	-	-	-	-	-		-	-	0
**# affected**		0	2	1	1	2	4	4	0	3	
**# evaluated**		27	27	24	26	26	25	26	27		
