# Supplementary material for: Epigenome-wide association study for atrazine induced transgenerational DNA methylation and histone retention sperm epigenetic biomarkers for disease
Source: PLoS One. 2020 Dec 16;15(12):e0239380. doi: 10.1371/journal.pone.0239380 (PMC7743986; doi:10.1371/journal.pone.0239380)
Supplement: S2 Table — DMR name, chromosome, start, stop, length, number signature windows, minimum p-value, max log-fold change, CpG number, CpG density, gene annotation, and gene category are presented. (PDF) [file pone.0239380.s009.pdf]

Supplemental Table S2
DMR Site List Lean p<1e-04

DMR Name	Chr	Start	Stop	Length	# Sig Win	minP	maxLFC	CpG #	CpG Density	Gene Annotation	Gene Category
DMR1:3777001	1	3777001	3778000	1000	1	7.28E-05	-0.9856521	4	0.4		
DMR1:8898001	1	8898001	8899000	1000	1	9.73E-05	0.5716763	12	1.2		
DMR1:12570001	1	12570001	12572000	2000	1	1.63E-05	1.1615804	11	0.55		
DMR1:17672001	1	17672001	17673000	1000	1	4.20E-06	-1.0527158	11	1.1	Ptprk	Signaling
DMR1:32949001	1	32949001	32950000	1000	1	9.76E-06	1.0334133	8	0.8		
DMR1:51059001	1	51059001	51061000	2000	1	3.04E-06	0.6531967	21	1.05	LOC108348175	
DMR1:54988001	1	54988001	54989000	1000	1	3.51E-05	-0.7927798	10	1		
DMR1:59062001	1	59062001	59063000	1000	1	8.67E-05	-0.8227653	7	0.7		
DMR1:60461001	1	60461001	60462000	1000	1	2.65E-05	0.8913279	6	0.6		
DMR1:61634001	1	61634001	61636000	2000	1	7.40E-05	0.5984479	26	1.3	Vom1r22	
DMR1:62989001	1	62989001	62994000	5000	1	7.58E-05	0.7635615	31	0.62	AABR07001967.1	
DMR1:64058001	1	64058001	64059000	1000	1	2.04E-06	0.9312087	9	0.9	Rps9;Tmc4	Translation;Metabolism
DMR1:73625001	1	73625001	73626000	1000	1	1.70E-05	-0.6335903	2	0.2	Lilrb3	
DMR1:84573001	1	84573001	84575000	2000	1	8.33E-05	-1.3572632	50	2.5	AABR07071891.2;AABR07071891.1	
DMR1:90093001	1	90093001	90094000	1000	1	5.33E-05	-1.1142215	21	2.1	Gpi;RGD1308428	Metabolism
DMR1:100037001	1	100037001	100039000	2000	1	1.23E-06	0.7093672	13	0.65	LOC103690048;AABR07003247.1	
DMR1:102406001	1	102406001	102407000	1000	1	5.15E-06	-1.1810397	8	0.8	Myod1;Kcnc1	Transcription;Metabolism
DMR1:119541001	1	119541001	119542000	1000	1	3.66E-05	-0.8792013	6	0.6		
DMR1:121276001	1	121276001	121277000	1000	1	2.18E-05	-1.0123376	2	0.2		
DMR1:133343001	1	133343001	133344000	1000	1	4.11E-05	-0.6890897	5	0.5	Mctp2	
DMR1:136307001	1	136307001	136309000	2000	1	4.02E-05	0.5557544	14	0.7	AABR07004305.2	
DMR1:137933001	1	137933001	137934000	1000	1	5.63E-06	0.6520826	20	2	Agbl1	Signaling
DMR1:144624001	1	144624001	144626000	2000	1	3.74E-05	-1.2001754	22	1.1	Efl1;AABR07004481.2	
DMR1:166396001	1	166396001	166397000	1000	1	9.23E-05	-0.9590654	8	0.8	Fchsd2;Atg16l2	Cell Cycle;Unknown
DMR1:172060001	1	172060001	172061000	1000	1	5.39E-05	-1.1821306	4	0.4	Olr235;Olr237	
DMR1:176980001	1	176980001	176981000	1000	1	7.18E-05	-0.9894784	4	0.4	Dkk3	Signaling
DMR1:180692001	1	180692001	180695000	3000	1	4.28E-05	-0.4675775	62	2.067		
DMR1:180785001	1	180785001	180794000	9000	1	2.26E-05	-0.6763045	149	1.656		
DMR1:186189001	1	186189001	186191000	2000	1	1.22E-05	0.5796836	13	0.65	Sox6	Development
DMR1:193113001	1	193113001	193115000	2000	1	9.07E-05	-0.8699941	28	1.4	Tnrc6a	Unknown
DMR1:196233001	1	196233001	196235000	2000	1	9.45E-05	0.7734894	10	0.5		
DMR1:217492001	1	217492001	217494000	2000	1	3.89E-05	1.0377914	13	0.65	Shank2	Protein Binding
DMR1:228155001	1	228155001	228156000	1000	1	6.81E-05	-0.9491918	6	0.6	Mrpl16;Stx3	Transcription
DMR1:236357001	1	236357001	236358000	1000	1	2.52E-06	0.952331	9	0.9		
DMR1:236588001	1	236588001	236589000	1000	1	6.94E-05	0.6569094	17	1.7	AABR07006475.1;Gcnt1	Golgi
DMR1:245147001	1	245147001	245148000	1000	1	5.61E-05	-0.7475844	2	0.2		
DMR1:248225001	1	248225001	248226000	1000	1	2.48E-06	0.649355	10	1	Uhrf2	Transcription
DMR1:255321001	1	255321001	255322000	1000	1	9.12E-05	-0.72418	10	1		
DMR1:257536001	1	257536001	257537000	1000	1	6.72E-05	-1.3130463	8	0.8	LOC685933	Signaling
DMR1:257885001	1	257885001	257886000	1000	1	5.36E-06	0.8948901	7	0.7	LOC685933;LOC100911660	Signaling;Epigenetic
DMR1:267735001	1	267735001	267736000	1000	1	7.12E-05	0.5520989	22	2.2	Cfap58	
DMR1:281205001	1	281205001	281206000	1000	1	3.74E-05	-0.9075555	9	0.9		
DMR2:15554001	2	15554001	15555000	1000	1	7.49E-05	-0.9298355	4	0.4		
DMR2:29479001	2	29479001	29480000	1000	1	1.21E-05	-1.4504728	39	3.9	Zfp366	Transcription
DMR2:48679001	2	48679001	48680000	1000	1	6.94E-05	0.6278008	7	0.7		
DMR2:52076001	2	52076001	52077000	1000	1	6.82E-06	-0.893309	7	0.7		
DMR2:55578001	2	55578001	55579000	1000	1	3.13E-05	-1.2853593	4	0.4		
DMR2:67357001	2	67357001	67359000	2000	1	6.93E-06	-1.1419582	9	0.45		
DMR2:67886001	2	67886001	67887000	1000	1	7.53E-05	-0.6470419	9	0.9		
DMR2:114357001	2	114357001	114358000	1000	1	3.14E-06	-1.3964842	2	0.2	Tnik	Signaling
DMR2:128330001	2	128330001	128331000	1000	1	8.70E-05	-1.3358063	8	0.8		
DMR2:135417001	2	135417001	135418000	1000	1	1.36E-05	-0.8030921	7	0.7		
DMR2:135544001	2	135544001	135547000	3000	2	8.22E-07	-2.4345818	15	0.5		
DMR2:143703001	2	143703001	143704000	1000	1	5.77E-05	-1.0970776	12	1.2		
DMR2:160562001	2	160562001	160563000	1000	1	8.99E-05	-1.0858166	17	1.7		
DMR2:161527001	2	161527001	161530000	3000	1	1.28E-06	-0.7280003	78	2.6		
DMR2:182187001	2	182187001	182188000	1000	1	8.18E-05	-1.2082719	12	1.2		
DMR2:187780001	2	187780001	187781000	1000	1	3.27E-06	0.7383221	8	0.8	Pmf1;Slc25a44	Metabolism
DMR2:211690001	2	211690001	211692000	2000	1	2.00E-05	-1.6167339	21	1.05	Fndc7;Mir6314	
DMR2:229666001	2	229666001	229667000	1000	1	4.83E-05	-1.069674	5	0.5	Ugt8	Metabolism
DMR2:236286001	2	236286001	236287000	1000	1	6.97E-05	-1.1226707	12	1.2	Lef1	Transcription
DMR2:238069001	2	238069001	238070000	1000	1	3.75E-05	-0.9625217	26	2.6	Npnt	Extracellular Matrix
DMR2:253348001	2	253348001	253349000	1000	1	8.27E-08	-1.8831156	3	0.3		
DMR2:259748001	2	259748001	259749000	1000	1	6.90E-05	-1.330414	4	0.4	St6galnac3	Metabolism
DMR3:9094001	3	9094001	9095000	1000	1	7.63E-06	0.6316111	20	2	LOC100361008	

DMR3:22336001	3	22336001	22337000	1000	1	8.30E-05	-0.9218372	7	0.7	Dennd1a	Signaling
DMR3:28384001	3	28384001	28386000	2000	1	9.30E-05	-1.3794833	9	0.45		
DMR3:28913001	3	28913001	28914000	1000	1	2.63E-05	-0.9067119	16	1.6	Arhgap15	Signaling
DMR3:31909001	3	31909001	31910000	1000	1	6.67E-05	-1.0115509	2	0.2		
DMR3:35273001	3	35273001	35274000	1000	1	1.72E-05	0.6139203	14	1.4	Lypd6b	
DMR3:45131001	3	45131001	45132000	1000	1	4.13E-05	-0.8221292	12	1.2	Ccdc148	
DMR3:54437001	3	54437001	54438000	1000	1	9.98E-07	0.844713	16	1.6	Stk39	
DMR3:55662001	3	55662001	55664000	2000	1	7.92E-05	-0.72021	25	1.25	Lrp2	Receptor
DMR3:72807001	3	72807001	72809000	2000	1	1.33E-08	-1.0609646	17	0.85	Olr441;Olr442	
DMR3:80623001	3	80623001	80625000	2000	1	2.85E-05	-1.2304495	16	0.8	Harbi1;Ambra1	
DMR3:81644001	3	81644001	81645000	1000	1	9.47E-06	-0.5766329	6	0.6		
DMR3:83767001	3	83767001	83768000	1000	1	2.46E-06	0.7953334	7	0.7		
DMR3:83852001	3	83852001	83854000	2000	2	4.33E-05	0.839878	14	0.7		
DMR3:85010001	3	85010001	85011000	1000	1	9.63E-07	0.7192169	6	0.6		
DMR3:92254001	3	92254001	92255000	1000	1	7.91E-05	0.6212279	9	0.9		
DMR3:104483001	3	104483001	104485000	2000	1	3.89E-05	-1.1733107	13	0.65	Ryr3	Receptor
DMR3:122418001	3	122418001	122419000	1000	1	6.11E-06	-1.327026	10	1	AABR07053717.1	
DMR3:133610001	3	133610001	133611000	1000	1	9.28E-05	-1.083429	8	0.8		
DMR3:138385001	3	138385001	138386000	1000	1	2.93E-05	-1.1094787	12	1.2	Snx5;Snord17	Transport
DMR3:144531001	3	144531001	144533000	2000	1	3.65E-05	-1.1577189	4	0.2		
DMR3:145951001	3	145951001	145952000	1000	1	9.28E-05	-1.1799896	20	2		
DMR3:158279001	3	158279001	158280000	1000	1	1.04E-05	-1.6350769	10	1	Ptprt	Receptor
DMR4:22952001	4	22952001	22953000	1000	1	9.42E-05	-0.9415369	4	0.4	Adam22	Protease
DMR4:33948001	4	33948001	33949000	1000	1	8.60E-05	0.5491901	10	1	C1galt1;AABR07059757.1	Metabolism
DMR4:35101001	4	35101001	35102000	1000	1	2.08E-05	-0.9784684	24	2.4		
DMR4:35694001	4	35694001	35696000	2000	1	9.96E-05	0.624834	12	0.6		
DMR4:54492001	4	54492001	54493000	1000	1	8.53E-05	-0.8688826	2	0.2	Grm8	Receptor
DMR4:55945001	4	55945001	55946000	1000	1	4.93E-06	-1.5899731	9	0.9	Snd1	Transcription
DMR4:58045001	4	58045001	58046000	1000	1	2.76E-05	-1.0170658	30	3	Mest	Protease
DMR4:61022001	4	61022001	61023000	1000	1	7.63E-05	-1.1231757	14	1.4	Exoc4	Transport
DMR4:64433001	4	64433001	64435000	2000	1	2.46E-05	-1.0461751	25	1.25	Dgki	Signaling
DMR4:81237001	4	81237001	81238000	1000	1	4.59E-05	0.7549328	5	0.5	Nfe2l3;Hnrnpa2b1;Cbx3	Transcription;Translation
DMR4:85305001	4	85305001	85306000	1000	1	1.76E-05	-0.8731499	9	0.9	Crhr2	Receptor
DMR4:86860001	4	86860001	86861000	1000	1	5.78E-05	-0.6496695	11	1.1	AABR07060620.2	
DMR4:99183001	4	99183001	99184000	1000	1	5.42E-05	-1.0901763	10	1	Cd8b	Receptor
DMR4:134886001	4	134886001	134887000	1000	1	5.59E-05	-0.8213068	8	0.8	Cntn3	Cytoskeleton
DMR4:137264001	4	137264001	137265000	1000	1	3.39E-05	-1.400373	8	0.8		
DMR4:146553001	4	146553001	146555000	2000	1	4.51E-05	-1.0053376	51	2.55		
DMR4:156121001	4	156121001	156122000	1000	1	1.56E-05	-0.7972213	4	0.4	Clec4b2	Unknown
DMR4:170041001	4	170041001	170042000	1000	1	1.15E-05	-1.1092874	15	1.5		
DMR4:178753001	4	178753001	178754000	1000	1	7.07E-06	-1.339187	11	1.1		
DMR5:4077001	5	4077001	4080000	3000	1	1.22E-05	-1.1391649	34	1.133		
DMR5:14798001	5	14798001	14801000	3000	1	5.24E-05	-0.6922217	29	0.967		
DMR5:16844001	5	16844001	16846000	2000	1	3.75E-07	-1.6760922	21	1.05	Chchd7	
DMR5:27952001	5	27952001	27953000	1000	1	3.64E-05	-1.1767084	10	1		
DMR5:36774001	5	36774001	36775000	1000	1	2.65E-05	-0.9490611	4	0.4		
DMR5:42299001	5	42299001	42300000	1000	1	4.57E-05	-0.7690797	8	0.8		
DMR5:45966001	5	45966001	45968000	2000	1	1.27E-05	-0.9429073	4	0.2		
DMR5:57825001	5	57825001	57826000	1000	1	2.76E-05	0.7154461	8	0.8	Kif24	Cytoskeleton
DMR5:59628001	5	59628001	59629000	1000	1	4.32E-06	-0.9413961	5	0.5	Rnf38	
DMR5:61287001	5	61287001	61288000	1000	1	2.60E-05	0.6032875	9	0.9		
DMR5:96010001	5	96010001	96011000	1000	1	9.02E-05	0.6006966	3	0.3		
DMR5:107722001	5	107722001	107723000	1000	1	1.95E-05	-1.2038968	14	1.4	Mtap	
DMR5:126489001	5	126489001	126492000	3000	1	2.40E-06	-1.0480889	28	0.933		
DMR5:144201001	5	144201001	144203000	2000	1	5.39E-05	-1.0931817	14	0.7	Thrap3	Receptor
DMR5:148105001	5	148105001	148106000	1000	1	4.54E-05	0.5257974	18	1.8	U1	
DMR5:151703001	5	151703001	151704000	1000	1	2.48E-05	-0.6461152	25	2.5	Tent5b;Trnp1	
DMR5:155898001	5	155898001	155900000	2000	1	1.40E-05	0.8938407	22	1.1		
DMR5:156503001	5	156503001	156504000	1000	1	9.09E-05	-1.0530763	8	0.8	Eif4g3	Transcription
DMR5:172988001	5	172988001	172989000	1000	1	3.10E-06	1.0771823	6	0.6	Gnb1;Nadk	Signaling
DMR6:4238001	6	4238001	4239000	1000	1	4.00E-05	-0.8655096	9	0.9		
DMR6:4647001	6	4647001	4648000	1000	1	7.97E-06	-1.4071375	8	0.8		
DMR6:36731001	6	36731001	36732000	1000	1	2.17E-06	-1.1076164	8	0.8	AABR07063599.1	
DMR6:65128001	6	65128001	65130000	2000	1	2.08E-05	-1.4029828	23	1.15	Stxbp6	Receptor
DMR6:87150001	6	87150001	87151000	1000	1	1.65E-05	0.6609973	5	0.5		
DMR6:112575001	6	112575001	112576000	1000	1	7.50E-06	-1.0091646	6	0.6	Nrxn3	Receptor
DMR6:120975001	6	120975001	120976000	1000	1	9.48E-05	-1.0738573	7	0.7		
DMR6:134390001	6	134390001	134391000	1000	1	3.52E-05	-0.7153946	11	1.1		
DMR6:140003001	6	140003001	140005000	2000	1	7.98E-05	-0.7951035	6	0.3	AABR07065740.1	
DMR7:16238001	7	16238001	16241000	3000	1	2.19E-05	-1.5011709	134	4.467		

DMR7:20201001	7	20201001	20202000	1000	1	7.20E-05	-0.5294984	12	1.2	AABR07056103.1	
DMR7:21724001	7	21724001	21725000	1000	1	8.45E-05	-0.5506091	12	1.2		
DMR7:49057001	7	49057001	49058000	1000	1	9.56E-05	-1.3316113	15	1.5	Acss3	Metabolism
DMR7:75030001	7	75030001	75031000	1000	1	1.74E-05	-1.0096734	14	1.4	Spag1	
DMR7:129366001	7	129366001	129367000	1000	1	1.37E-06	0.7247297	5	0.5	LOC108351524	
DMR7:134309001	7	134309001	134310000	1000	1	6.34E-05	0.5170526	25	2.5		
DMR7:136265001	7	136265001	136266000	1000	1	1.78E-06	-0.8861094	11	1.1	Tmem117	Unknown
DMR7:136488001	7	136488001	136489000	1000	1	5.92E-05	-1.0784855	8	0.8	AABR07058758.1	
DMR7:140945001	7	140945001	140949000	4000	1	6.54E-05	-0.9324618	61	1.525	Fam186b	
DMR7:144045001	7	144045001	144046000	1000	1	8.02E-06	-0.9276276	3	0.3	Sp1;Amhr2	Transcription;Receptor
DMR7:145393001	7	145393001	145395000	2000	1	2.45E-07	-1.4650004	29	1.45		
DMR8:1559001	8	1559001	1560000	1000	1	2.45E-05	-1.0874875	4	0.4	Gria4	Signaling
DMR8:14587001	8	14587001	14588000	1000	1	4.04E-05	-1.3373711	3	0.3	Fat3	Transcription
DMR8:22992001	8	22992001	22993000	1000	1	1.25E-05	-0.937642	27	2.7	Rgl3;Ccdc151	Signaling
DMR8:38858001	8	38858001	38861000	3000	1	6.59E-05	0.7257845	9	0.3		
DMR8:42169001	8	42169001	42170000	1000	1	1.24E-05	-0.6905565	13	1.3	AABR07069913.1	
DMR8:45225001	8	45225001	45226000	1000	1	1.21E-06	-1.1426345	13	1.3	Crtam;Ubash3b	Receptor;Signaling
DMR8:45622001	8	45622001	45623000	1000	1	4.87E-05	-0.9210329	12	1.2		
DMR8:48107001	8	48107001	48108000	1000	1	2.75E-05	-1.0473603	13	1.3	Nectin1	
DMR8:76759001	8	76759001	76760000	1000	1	7.07E-05	0.5067759	16	1.6	Myo1e	Cytoskeleton
DMR8:77492001	8	77492001	77495000	3000	1	8.04E-05	0.6140048	14	0.467		
DMR8:103064001	8	103064001	103065000	1000	1	7.38E-05	0.6449066	9	0.9		
DMR8:104216001	8	104216001	104217000	1000	1	8.05E-05	-0.8025876	12	1.2	Atp1b3	Metabolism
DMR8:112479001	8	112479001	112480000	1000	1	7.21E-05	0.7093462	10	1	AABR07071374.1	
DMR8:116179001	8	116179001	116180000	1000	1	1.45E-05	-0.7814896	15	1.5	Cacna2d2	Transport
DMR8:126610001	8	126610001	126611000	1000	1	8.16E-07	0.8385739	10	1		
DMR9:4276001	9	4276001	4278000	2000	1	6.79E-05	-1.2488939	12	0.6	RGD1562392	Metabolism
DMR9:4455001	9	4455001	4456000	1000	1	8.97E-05	0.5330821	6	0.6	AABR07066180.1	
DMR9:4632001	9	4632001	4633000	1000	1	1.08E-05	-1.5578483	4	0.4	AABR07066188.1	
DMR9:11761001	9	11761001	11763000	2000	1	8.27E-05	-0.7520611	69	3.45		
DMR9:12672001	9	12672001	12673000	1000	1	9.16E-05	-1.1623316	8	0.8		
DMR9:21840001	9	21840001	21841000	1000	1	4.26E-05	-0.9792874	10	1		
DMR9:33491001	9	33491001	33492000	1000	1	8.49E-05	0.6530983	5	0.5		
DMR9:73193001	9	73193001	73194000	1000	1	9.17E-05	-1.3298769	17	1.7		
DMR9:82099001	9	82099001	82100000	1000	1	1.19E-05	-0.8831398	9	0.9		
DMR9:87704001	9	87704001	87705000	1000	1	3.36E-05	-1.2728764	10	1		
DMR9:92654001	9	92654001	92655000	1000	1	5.54E-05	0.5327646	5	0.5	Sp140	Transcription
DMR9:97042001	9	97042001	97044000	2000	1	1.59E-05	-0.8225465	19	0.95		
DMR9:110772001	9	110772001	110774000	2000	1	1.81E-05	-1.0459221	17	0.85	Fbxl17	Proteolysis
DMR9:111321001	9	111321001	111323000	2000	1	1.15E-05	-1.2731165	31	1.55	RGD1562136	Unknown
DMR9:111872001	9	111872001	111873000	1000	1	5.56E-05	-0.9137144	6	0.6	Fer	
DMR9:112978001	9	112978001	112980000	2000	1	1.62E-05	-0.8386865	37	1.85		
DMR9:118729001	9	118729001	118731000	2000	1	4.10E-05	-1.2728632	27	1.35	Dlgap1	Signaling
DMR10:6877001	10	6877001	6878000	1000	1	7.60E-05	0.5779581	15	1.5	RGD1309748	
DMR10:22034001	10	22034001	22035000	1000	1	8.34E-05	-0.8230772	7	0.7		
DMR10:34421001	10	34421001	34422000	1000	1	5.55E-05	-0.8860779	5	0.5	LOC684471;Btnl9	
DMR10:37502001	10	37502001	37503000	1000	1	4.67E-05	-1.2280332	9	0.9	Cdkl3	Cell Cycle
DMR10:42275001	10	42275001	42276000	1000	1	1.67E-05	-0.9886735	7	0.7	AABR07029636.1	
DMR10:50923001	10	50923001	50924000	1000	1	7.52E-05	-1.0279765	5	0.5	Hs3st3a1	Metabolism
DMR10:51079001	10	51079001	51080000	1000	1	9.10E-05	-0.9780121	18	1.8		
DMR10:51586001	10	51586001	51588000	2000	1	2.24E-07	-1.7435099	19	0.95	Arhgap44	
DMR10:52727001	10	52727001	52728000	1000	1	2.34E-05	-1.3782854	9	0.9		
DMR10:65156001	10	65156001	65157000	1000	1	7.54E-05	-0.8544568	5	0.5	Cryba1	Unknown
DMR10:67853001	10	67853001	67854000	1000	1	1.02E-05	-0.7774424	7	0.7	Psmd11;Cdk5r1	Unknown;Signaling
DMR10:72178001	10	72178001	72180000	2000	1	3.32E-05	-0.874667	12	0.6	Ggnbp2	
DMR10:83675001	10	83675001	83676000	1000	1	6.63E-05	0.8989509	9	0.9		
DMR10:84324001	10	84324001	84327000	3000	1	3.84E-07	-1.3372479	48	1.6	Skap1	
DMR10:95354001	10	95354001	95355000	1000	1	3.24E-05	-0.987754	6	0.6	Bptf;AABR07030599.2	Metabolism
DMR10:110055001	10	110055001	110056000	1000	1	7.76E-05	0.5178975	9	0.9	Ccdc57	Unknown
DMR11:7835001	11	7835001	7836000	1000	1	8.98E-05	-0.9775929	9	0.9		
DMR11:21835001	11	21835001	21836000	1000	1	8.32E-05	-1.1106651	8	0.8		
DMR11:25159001	11	25159001	25160000	1000	1	5.78E-06	-1.3820943	9	0.9		
DMR11:41804001	11	41804001	41805000	1000	1	2.85E-05	-0.7028727	1	0.1		
DMR11:54174001	11	54174001	54175000	1000	1	8.97E-06	-0.8782054	8	0.8	LOC100909977	
DMR11:81718001	11	81718001	81720000	2000	1	5.29E-05	-0.9384803	18	0.9	AABR07034632.1	
DMR12:14051001	12	14051001	14053000	2000	1	4.02E-05	0.8745884	37	1.85	Mmd2	Development
DMR12:22988001	12	22988001	22989000	1000	1	8.14E-05	-1.2033375	28	2.8	Col26a1	Extracellular Matrix
DMR13:10576001	13	10576001	10577000	1000	1	6.93E-05	-0.6521812	19	1.9		
DMR13:25383001	13	25383001	25384000	1000	1	1.20E-06	-1.381191	4	0.4		
DMR13:29632001	13	29632001	29633000	1000	1	3.01E-05	-1.3212516	4	0.4		

DMR13:33356001	13	33356001	33357000	1000	1	8.27E-05	-1.1366329	20	2		
DMR13:40898001	13	40898001	40899000	1000	1	4.46E-05	-0.6695408	7	0.7		
DMR13:41380001	13	41380001	41381000	1000	1	3.40E-06	-1.5801023	0	0	LOC102554127	
DMR13:50833001	13	50833001	50834000	1000	1	6.81E-05	0.4735309	11	1.1		
DMR13:55393001	13	55393001	55394000	1000	1	8.15E-05	-0.9918632	9	0.9		
DMR13:61792001	13	61792001	61794000	2000	1	7.82E-05	-1.3691315	11	0.55		
DMR13:80180001	13	80180001	80181000	1000	1	4.60E-05	-1.2104745	14	1.4	Dnm3	Cytoskeleton
DMR13:88516001	13	88516001	88517000	1000	1	2.31E-05	-1.1329653	7	0.7	Uhmk1	Transcription
DMR13:95030001	13	95030001	95031000	1000	1	5.79E-05	-0.7427282	13	1.3	Sdccag8	
DMR13:109034001	13	109034001	109035000	1000	1	2.19E-06	-1.1918238	27	2.7		
DMR13:112927001	13	112927001	112928000	1000	1	2.59E-05	-1.1886779	3	0.3		
DMR14:10051001	14	10051001	10052000	1000	1	6.33E-06	0.6934779	19	1.9		
DMR14:11157001	14	11157001	11158000	1000	1	6.30E-05	-1.1594133	8	0.8	Tmem150c	Unknown
DMR14:11306001	14	11306001	11307000	1000	1	1.96E-05	-0.9296349	8	0.8	AC131411.1;AC131411.2	
DMR14:15082001	14	15082001	15083000	1000	1	9.14E-05	-1.2044525	6	0.6	Cnot6l	Transcription
DMR14:24505001	14	24505001	24506000	1000	1	6.98E-05	-0.9787874	7	0.7		
DMR14:28394001	14	28394001	28395000	1000	1	4.31E-05	-0.959716	22	2.2	Adgrl3	
DMR14:41131001	14	41131001	41132000	1000	1	2.86E-06	-1.3215275	4	0.4		
DMR14:48496001	14	48496001	48497000	1000	1	7.60E-05	-1.3414693	6	0.6		
DMR14:61717001	14	61717001	61718000	1000	1	6.04E-05	-1.2461379	3	0.3		
DMR14:64170001	14	64170001	64171000	1000	1	3.05E-05	-1.0280359	10	1		
DMR14:66281001	14	66281001	66282000	1000	1	2.33E-05	-1.3362313	4	0.4		
DMR14:73871001	14	73871001	73873000	2000	1	1.76E-05	-0.7555674	16	0.8		
DMR14:74209001	14	74209001	74210000	1000	1	9.79E-05	-0.8368771	0	0		
DMR14:83052001	14	83052001	83053000	1000	1	3.55E-05	-0.910514	7	0.7	Ywhah	Signaling
DMR14:94372001	14	94372001	94373000	1000	1	3.75E-05	-0.6130377	11	1.1		
DMR14:103131001	14	103131001	103132000	1000	1	3.62E-06	0.6294533	8	0.8		
DMR15:5662001	15	5662001	5663000	1000	1	7.79E-05	-1.3113981	8	0.8	AABR07016976.1;AABR07016975.1;AABR07016975.2	
DMR15:12321001	15	12321001	12322000	1000	1	3.94E-05	-0.684535	5	0.5		
DMR15:19614001	15	19614001	19615000	1000	1	9.31E-05	-1.0463242	40	4	Gpr137c;LOC102552640;Ero1a	
DMR15:19735001	15	19735001	19736000	1000	1	3.97E-05	-0.9277614	20	2	Gnpnat1	Metabolism
DMR15:34348001	15	34348001	34349000	1000	1	4.31E-05	-0.9251778	14	1.4	Mdp1;Nedd8;Gmpr2	Development;Metabolism
DMR15:45682001	15	45682001	45683000	1000	1	9.19E-05	-1.0855668	11	1.1		
DMR15:46324001	15	46324001	46325000	1000	1	2.38E-05	-0.9833119	14	1.4	Ctsb	Protease
DMR15:54866001	15	54866001	54867000	1000	1	2.68E-06	0.7064705	18	1.8		
DMR15:58245001	15	58245001	58246000	1000	1	3.18E-06	-1.5973505	30	3		
DMR15:65236001	15	65236001	65237000	1000	1	3.39E-06	-1.283821	3	0.3		
DMR15:71081001	15	71081001	71082000	1000	1	2.48E-06	-1.4464039	5	0.5	U6	
DMR15:76685001	15	76685001	76686000	1000	1	8.13E-05	0.6125205	5	0.5		
DMR15:77264001	15	77264001	77266000	2000	1	5.15E-05	-0.8704296	15	0.75		
DMR15:79903001	15	79903001	79904000	1000	1	9.40E-05	0.5515135	4	0.4		
DMR15:86055001	15	86055001	86056000	1000	1	3.47E-05	-0.8237166	9	0.9	Tbc1d4	
DMR15:92083001	15	92083001	92086000	3000	1	4.61E-05	-0.903459	72	2.4	Mycbp2	Metabolism
DMR15:93602001	15	93602001	93603000	1000	1	5.56E-05	-1.2156462	9	0.9	Mycbp2;Acod1	Metabolism
DMR15:103353001	15	103353001	103355000	2000	1	2.95E-05	-0.7317328	21	1.05	Gpr180	
DMR16:2305001	16	2305001	2306000	1000	1	9.20E-05	-0.8426761	14	1.4	Dennd6a	
DMR16:40117001	16	40117001	40118000	1000	1	4.36E-06	0.7974991	7	0.7		
DMR16:64601001	16	64601001	64602000	1000	1	2.60E-05	-1.202847	23	2.3		
DMR16:79208001	16	79208001	79209000	1000	1	7.81E-05	-1.2422565	20	2	AABR07026499.2	
DMR17:2283001	17	2283001	2284000	1000	1	8.14E-05	-0.8585216	9	0.9	AABR07026805.1	
DMR17:4685001	17	4685001	4688000	3000	1	1.26E-06	-1.3819862	33	1.1	AABR07026912.1	
DMR17:17517001	17	17517001	17518000	1000	1	5.01E-05	0.6796817	9	0.9		
DMR17:21731001	17	21731001	21732000	1000	1	1.98E-05	-1.3067223	9	0.9	Gcnt2	Golgi
DMR17:44320001	17	44320001	44322000	2000	1	6.32E-06	-0.7095239	51	2.55		
DMR17:49774001	17	49774001	49775000	1000	1	3.41E-05	-0.9119001	6	0.6		
DMR17:50344001	17	50344001	50345000	1000	1	1.50E-05	0.5662602	8	0.8		
DMR17:56478001	17	56478001	56479000	1000	1	2.17E-05	-0.6900414	8	0.8		
DMR17:65166001	17	65166001	65167000	1000	1	6.41E-05	-0.8129795	4	0.4		
DMR17:66474001	17	66474001	66475000	1000	1	3.28E-05	-1.2410707	11	1.1	LOC108348069	
DMR17:74145001	17	74145001	74146000	1000	1	6.61E-05	0.5585463	7	0.7		
DMR17:78347001	17	78347001	78348000	1000	1	4.55E-05	0.5304743	13	1.3		
DMR17:79782001	17	79782001	79783000	1000	1	1.10E-05	-1.0764949	17	1.7	Mindy3	
DMR17:84788001	17	84788001	84789000	1000	1	4.72E-05	0.6034913	18	1.8		
DMR18:7522001	18	7522001	7523000	1000	1	1.50E-05	-0.6837076	3	0.3		
DMR18:9229001	18	9229001	9231000	2000	1	7.85E-05	0.5695869	8	0.4		
DMR18:15385001	18	15385001	15386000	1000	1	7.05E-05	0.474984	8	0.8	Trappc8	
DMR18:43503001	18	43503001	43504000	1000	1	5.58E-05	-1.1428906	2	0.2		
DMR18:52216001	18	52216001	52217000	1000	1	7.16E-05	-1.4935922	29	2.9	Megf10	Extracellular Matrix
DMR18:65445001	18	65445001	65446000	1000	1	1.89E-05	-1.1336444	16	1.6	Tcf4	Transcription
DMR18:69179001	18	69179001	69181000	2000	1	1.87E-05	-1.2072846	12	0.6		

DMR18:72859001	18	72859001	72860000	1000	1	1.39E-05	-0.8244131	14	1.4		
DMR19:209001	19	209001	210000	1000	1	7.54E-05	-0.9830732	8	0.8	LOC108348293	
DMR19:42053001	19	42053001	42054000	1000	1	6.10E-05	0.7609243	11	1.1	Pkd1l3	
DMR19:59087001	19	59087001	59088000	1000	1	4.27E-05	0.7978158	13	1.3		
DMR20:13324001	20	13324001	13325000	1000	1	8.91E-05	-1.1880511	11	1.1		
DMR20:16478001	20	16478001	16480000	2000	1	4.99E-05	-1.1645507	19	0.95		
DMR20:18237001	20	18237001	18238000	1000	1	2.42E-05	-1.2677377	30	3		
DMR20:19216001	20	19216001	19217000	1000	1	9.75E-05	-1.3000725	20	2		
DMR20:31107001	20	31107001	31109000	2000	1	2.11E-05	-0.9964676	26	1.3	Lrrc20	Development
DMR20:44016001	20	44016001	44017000	1000	1	8.55E-05	-1.1188359	1	0.1		
DMR20:45313001	20	45313001	45315000	2000	1	3.60E-07	-0.8789464	30	1.5	Rpf2	Transcription
DMR20:49653001	20	49653001	49654000	1000	1	1.97E-05	-1.262635	19	1.9		
DMR20:50616001	20	50616001	50617000	1000	1	5.25E-05	-1.1962739	23	2.3		
DMR20:56180001	20	56180001	56183000	3000	1	7.64E-05	-0.8543206	90	3	5_8S_rRNA;AABR07045678.1;Cd99	
DMRX:3938001	X	3938001	3939000	1000	1	3.40E-05	-1.1359343	8	0.8		
DMRX:7160001	X	7160001	7161000	1000	1	7.72E-05	0.7815561	3	0.3		
DMRX:11626001	X	11626001	11627000	1000	1	1.08E-05	0.7977537	15	1.5		
DMRX:11802001	X	11802001	11803000	1000	1	4.57E-05	-0.9249072	11	1.1		
DMRX:50872001	X	50872001	50873000	1000	1	9.42E-05	-0.8374812	5	0.5		
DMRX:79212001	X	79212001	79213000	1000	1	6.66E-05	-0.7351413	8	0.8		
DMRX:79615001	X	79615001	79616000	1000	1	5.15E-05	-1.1019625	3	0.3		
DMRX:88614001	X	88614001	88616000	2000	1	6.84E-05	0.5817764	7	0.35		
DMRX:124247001	X	124247001	124248000	1000	1	7.71E-06	0.7895461	6	0.6	Rhox12	
DMRX:127939001	X	127939001	127940000	1000	1	3.32E-05	0.742748	8	0.8		
DMRX:149565001	X	149565001	149566000	1000	1	7.21E-05	0.6489092	4	0.4		
DMRX:150187001	X	150187001	150188000	1000	1	8.94E-05	-0.7802851	6	0.6		
