# Supplementary material for: Epigenome-wide association study for atrazine induced transgenerational DNA methylation and histone retention sperm epigenetic biomarkers for disease
Source: PLoS One. 2020 Dec 16;15(12):e0239380. doi: 10.1371/journal.pone.0239380 (PMC7743986; doi:10.1371/journal.pone.0239380)
Supplement: S3 Table — DMR name, chromosome, start, stop, length, number signature windows, minimum p-value, max log-fold change, CpG number, CpG density, gene annotation, and gene category are presented. (PDF) [file pone.0239380.s010.pdf]

Supplemental Table S3
DMR Site List Kidney Disease p<1e-04

DMR Name	Chr	Start	Stop	Length	# Sig Win	minP	maxLFC	CpG #	CpG Density	Gene Annotation	Gene Category
DMR1:2794001	1	2794001	2796000	2000	1	3.29E-05	-0.7972089	32	1.6	Sash1	
DMR1:2798001	1	2798001	2799000	1000	1	2.67E-05	-1.5212843	27	2.7	Sash1	
DMR1:9332001	1	9332001	9333000	1000	1	2.69E-05	1.2137059	12	1.2		
DMR1:26464001	1	26464001	26465000	1000	1	8.62E-05	-0.6720654	9	0.9		
DMR1:28855001	1	28855001	28856000	1000	1	3.32E-06	-1.5460415	12	1.2		
DMR1:62630001	1	62630001	62631000	1000	1	8.59E-05	0.6426256	8	0.8		
DMR1:62944001	1	62944001	62945000	1000	1	8.66E-05	1.2189637	8	0.8	LOC682419	
DMR1:62984001	1	62984001	62987000	3000	1	8.11E-05	1.305553	11	0.367	AABR07001967.1	
DMR1:63053001	1	63053001	63054000	1000	1	9.40E-05	1.4987071	4	0.4		
DMR1:75126001	1	75126001	75129000	3000	1	5.15E-05	1.449691	30	1	Vom1r58	
DMR1:76331001	1	76331001	76339000	8000	1	4.35E-05	1.498325	71	0.887	Sult2a6;AABR07002523.1	Metabolism
DMR1:76584001	1	76584001	76586000	2000	1	2.54E-05	0.889275	6	0.3	Sult2a6	Metabolism
DMR1:76789001	1	76789001	76791000	2000	1	4.04E-05	1.0782679	9	0.45	Sult2a6	Metabolism
DMR1:76982001	1	76982001	76983000	1000	1	8.61E-06	0.9388829	4	0.4		
DMR1:77203001	1	77203001	77206000	3000	1	2.00E-07	1.0580387	8	0.267		
DMR1:77273001	1	77273001	77277000	4000	1	3.77E-05	1.2653056	29	0.725		
DMR1:93755001	1	93755001	93756000	1000	1	3.33E-06	1.0124224	30	3		
DMR1:100472001	1	100472001	100473000	1000	1	5.98E-05	-1.6703915	25	2.5	Lrrc4b;Aspdh;Josd2	Extracellular Matrix; Metabolism;Protease
DMR1:106755001	1	106755001	106756000	1000	1	1.51E-05	1.0421368	10	1		
DMR1:125140001	1	125140001	125141000	1000	1	2.28E-05	-0.9787437	4	0.4	Mtmr10	Cytoskeleton
DMR1:143957001	1	143957001	143958000	1000	1	2.39E-05	0.8345456	13	1.3		
DMR1:157667001	1	157667001	157668000	1000	1	5.65E-05	-0.9822366	3	0.3	Rab30;Ddias	Signaling
DMR1:160238001	1	160238001	160239000	1000	1	1.00E-06	0.7617582	12	1.2		
DMR1:162353001	1	162353001	162354000	1000	1	1.41E-07	1.235557	29	2.9	Alg8	Metabolism
DMR1:165110001	1	165110001	165111000	1000	1	2.46E-05	-0.7454669	25	2.5	Pold3	
DMR1:176740001	1	176740001	176742000	2000	1	3.33E-05	-1.8708424	11	0.55		
DMR1:181872001	1	181872001	181873000	1000	1	6.07E-05	-0.8661965	14	1.4		
DMR1:182871001	1	182871001	182872000	1000	1	3.49E-05	-0.8125988	14	1.4		
DMR1:192056001	1	192056001	192057000	1000	1	4.97E-05	0.8255612	10	1	Ndufab1;Palb2	Binding Protein
DMR1:211390001	1	211390001	211391000	1000	1	9.66E-05	-0.9319436	21	2.1	Jakmip3	
DMR1:239228001	1	239228001	239231000	3000	1	9.52E-05	1.1045493	33	1.1	RGD1359158	EST
DMR1:280961001	1	280961001	280962000	1000	1	9.46E-05	-1.1739744	6	0.6	AABR07007093.1	
DMR2:13576001	2	13576001	13577000	1000	1	3.60E-05	-1.8840782	7	0.7		
DMR2:21664001	2	21664001	21665000	1000	1	6.94E-06	-1.1510188	7	0.7	AABR07007642.1	
DMR2:23151001	2	23151001	23152000	1000	1	5.11E-05	-1.4526749	8	0.8		
DMR2:29652001	2	29652001	29653000	1000	1	5.97E-05	-1.0975518	8	0.8	Mrps27	Translation
DMR2:31151001	2	31151001	31152000	1000	1	1.77E-05	0.8857171	7	0.7		
DMR2:35847001	2	35847001	35848000	1000	1	3.56E-05	0.8473094	8	0.8	Rnf180	
DMR2:75962001	2	75962001	75964000	2000	1	2.75E-05	-1.3227656	10	0.5		
DMR2:110742001	2	110742001	110743000	1000	1	4.17E-05	-0.8543436	8	0.8		
DMR2:131758001	2	131758001	131759000	1000	1	7.33E-05	0.6192566	6	0.6		
DMR2:142020001	2	142020001	142022000	2000	1	7.94E-05	0.8984324	32	1.6		
DMR2:143409001	2	143409001	143410000	1000	1	3.67E-06	-1.1743313	3	0.3	AABR07010609.1	
DMR2:164109001	2	164109001	164110000	1000	1	3.59E-05	-0.6018404	5	0.5	Shox2	Transcription
DMR2:168951001	2	168951001	168952000	1000	1	7.61E-05	0.601907	6	0.6		
DMR2:178773001	2	178773001	178774000	1000	1	9.00E-05	-1.1057217	7	0.7		
DMR2:206024001	2	206024001	206026000	2000	1	7.89E-05	-0.6360451	12	0.6		
DMR2:211299001	2	211299001	211300000	1000	1	3.51E-06	-0.87063	12	1.2	5330417C22Rik;AC113756.3	
DMR2:220281001	2	220281001	220282000	1000	1	5.73E-05	0.9609198	6	0.6		
DMR2:221107001	2	221107001	221108000	1000	1	2.50E-05	0.8266039	10	1	Snx7	Signaling
DMR2:232406001	2	232406001	232407000	1000	1	9.22E-06	0.7191492	18	1.8		
DMR2:253213001	2	253213001	253215000	2000	1	6.53E-05	0.8665851	31	1.55		
DMR2:254222001	2	254222001	254223000	1000	1	9.28E-05	-0.7266525	13	1.3	AABR07013746.1	
DMR2:257895001	2	257895001	257896000	1000	1	5.06E-05	0.7643922	5	0.5		
DMR3:5434001	3	5434001	5435000	1000	1	5.14E-05	-1.0367478	15	1.5	Surf6	Development
DMR3:12433001	3	12433001	12434000	1000	1	2.88E-06	0.9378133	16	1.6		
DMR3:23869001	3	23869001	23870000	1000	1	4.74E-05	-1.2560924	9	0.9		
DMR3:29949001	3	29949001	29950000	1000	1	5.32E-05	0.6128633	14	1.4	Zeb2	Transcription
DMR3:63691001	3	63691001	63692000	1000	1	5.03E-05	1.0390139	10	1		
DMR3:72279001	3	72279001	72282000	3000	1	1.01E-05	-0.6821058	26	0.867	Rtn4rl2	Receptor

DMR3:93690001	3	93690001	93691000	1000	1	2.49E-05	-1.1555669	7	0.7	Nat10	Metabolism
DMR3:116697001	3	116697001	116698000	1000	1	4.68E-06	-1.9148556	13	1.3		
DMR3:121086001	3	121086001	121087000	1000	1	3.49E-05	-1.1674352	13	1.3		
DMR3:121353001	3	121353001	121354000	1000	1	7.44E-05	-0.9458851	10	1	Tmem87b	
DMR3:122059001	3	122059001	122060000	1000	1	4.44E-06	-1.2786071	8	0.8	Sirpb3	
DMR3:130336001	3	130336001	130338000	2000	1	6.64E-05	0.8856678	29	1.45		
DMR3:136718001	3	136718001	136719000	1000	1	1.00E-05	-1.0249815	12	1.2	Kif16b	Cytoskeleton
DMR3:147605001	3	147605001	147606000	1000	1	2.57E-05	-0.871565	10	1	Scrt2;AABR07054319.1	Transcription
DMR3:159809001	3	159809001	159810000	1000	1	8.26E-05	0.6740554	19	1.9	Oser1	
DMR3:164413001	3	164413001	164414000	1000	1	4.93E-05	-0.5889861	11	1.1		
DMR3:172071001	3	172071001	172072000	1000	1	6.11E-05	-1.5875891	12	1.2		
DMR3:173364001	3	173364001	173365000	1000	1	6.08E-05	-1.7881207	4	0.4	AABR07054909.1	
DMR4:18323001	4	18323001	18324000	1000	1	7.23E-05	-1.5028061	9	0.9	Sema3a	Growth Factors & Cytokines
DMR4:25638001	4	25638001	25640000	2000	1	3.54E-05	0.7836513	19	0.95	Gtpbp10	Signaling
DMR4:26266001	4	26266001	26268000	2000	1	6.42E-05	-0.8407566	6	0.3	Cdk14	Cell Cycle
DMR4:44361001	4	44361001	44364000	3000	1	4.38E-05	-0.8710819	29	0.967	Tes	Cytoskeleton
DMR4:49038001	4	49038001	49039000	1000	1	1.23E-06	0.8358168	11	1.1	Ing3	Transcription
DMR4:57325001	4	57325001	57326000	1000	1	7.01E-05	-0.8843102	12	1.2		
DMR4:58733001	4	58733001	58735000	2000	1	2.68E-05	0.7459138	16	0.8	Mkln1	Signaling
DMR4:73879001	4	73879001	73881000	2000	1	1.44E-05	-0.8473687	46	2.3		
DMR4:92541001	4	92541001	92542000	1000	1	3.77E-05	-1.3104003	7	0.7		
DMR4:120740001	4	120740001	120741000	1000	1	2.60E-05	-1.2392375	12	1.2	Mgll	Metabolism
DMR4:126092001	4	126092001	126093000	1000	1	5.43E-05	-1.6505674	6	0.6		
DMR4:139764001	4	139764001	139765000	1000	1	5.78E-05	1.0112862	12	1.2		
DMR4:148209001	4	148209001	148210000	1000	1	6.04E-05	0.9814071	9	0.9	Zfand4	
DMR4:153308001	4	153308001	153309000	1000	1	2.66E-05	0.9016817	11	1.1	Cecr2	Metabolism
DMR4:158230001	4	158230001	158231000	1000	1	8.56E-05	-1.2630273	6	0.6	Ano2	
DMR4:161378001	4	161378001	161379000	1000	1	7.70E-05	1.0580139	11	1.1	AABR07062111.1	
DMR5:1529001	5	1529001	1530000	1000	1	4.82E-05	-1.3279503	2	0.2		
DMR5:15532001	5	15532001	15534000	2000	1	3.30E-05	-1.0521501	14	0.7		
DMR5:29471001	5	29471001	29472000	1000	1	9.08E-05	1.0511141	9	0.9		
DMR5:31304001	5	31304001	31305000	1000	1	8.29E-05	-1.1208919	1	0.1	U6	
DMR5:38379001	5	38379001	38380000	1000	1	3.71E-05	0.7374326	6	0.6		
DMR5:59312001	5	59312001	59314000	2000	1	9.09E-05	-1.140107	28	1.4	Olr839	Receptor
DMR5:70133001	5	70133001	70134000	1000	1	7.39E-05	0.7471801	13	1.3		
DMR5:88155001	5	88155001	88156000	1000	1	1.93E-05	-1.5752595	20	2		
DMR5:123829001	5	123829001	123831000	2000	1	5.47E-05	0.6575117	20	1		
DMR5:124558001	5	124558001	124560000	2000	1	3.25E-05	0.9729254	9	0.45		
DMR5:133565001	5	133565001	133567000	2000	1	1.36E-06	-2.096761	28	1.4		
DMR5:156095001	5	156095001	156096000	1000	1	8.58E-05	-1.1539269	26	2.6	Alpl	Metabolism
DMR5:170341001	5	170341001	170342000	1000	1	8.73E-05	0.5081699	25	2.5		
DMR6:29342001	6	29342001	29343000	1000	1	8.98E-05	-1.1136225	32	3.2	Klhl29	Transcription
DMR6:38464001	6	38464001	38465000	1000	1	2.55E-05	1.0288354	5	0.5	Nbas	Unknown
DMR6:45697001	6	45697001	45698000	1000	1	5.84E-06	-1.9564851	5	0.5	Cmpk2	Metabolism
DMR6:53127001	6	53127001	53128000	1000	1	4.89E-05	-1.7845924	12	1.2		
DMR6:53251001	6	53251001	53252000	1000	1	9.56E-05	0.6481609	9	0.9		
DMR6:60906001	6	60906001	60907000	1000	1	7.57E-06	-1.1200326	3	0.3		
DMR6:72944001	6	72944001	72945000	1000	1	7.03E-05	0.7935659	15	1.5	Nubpl	Metabolism
DMR6:74423001	6	74423001	74424000	1000	1	2.19E-05	1.0780907	11	1.1		
DMR6:78051001	6	78051001	78052000	1000	1	7.94E-05	0.6411638	6	0.6		
DMR6:109250001	6	109250001	109251000	1000	1	2.97E-05	0.7318962	13	1.3		
DMR6:123909001	6	123909001	123910000	1000	1	2.24E-06	-1.2689023	20	2	Tdp1	DNA Repair
DMR6:132240001	6	132240001	132241000	1000	1	4.33E-05	-1.7418786	63	6.3	Hhipl1;Cyp46a1	Receptor;Metabolism
DMR6:145790001	6	145790001	145791000	1000	1	9.27E-05	-0.8429666	20	2	Cdca7l;Dnah11	Cytoskeleton
DMR7:2576001	7	2576001	2577000	1000	1	1.38E-05	-1.821193	5	0.5	Rbms2	Translation
DMR7:7691001	7	7691001	7692000	1000	1	1.21E-05	0.9036811	5	0.5		
DMR7:20208001	7	20208001	20209000	1000	1	2.93E-05	0.6403473	7	0.7	AABR07056103.1	
DMR7:24661001	7	24661001	24662000	1000	1	3.27E-05	1.0530116	11	1.1	Tmem263	
DMR7:27989001	7	27989001	27990000	1000	1	2.15E-06	-1.9315836	20	2		
DMR7:30139001	7	30139001	30140000	1000	1	3.48E-05	0.7456423	4	0.4	Nr1h4	Receptor
DMR7:37927001	7	37927001	37928000	1000	1	8.12E-06	-0.5990804	6	0.6		
DMR7:49314001	7	49314001	49315000	1000	1	6.12E-05	-1.5670915	5	0.5		
DMR7:51787001	7	51787001	51788000	1000	1	2.03E-05	1.1323373	7	0.7	Otogl	Unknown
DMR7:58324001	7	58324001	58325000	1000	1	4.06E-05	1.0933121	13	1.3	Tmem19	
DMR7:58835001	7	58835001	58839000	4000	1	6.44E-06	-1.4684352	40	1	Tspan8	Cytoskeleton

DMR7:59703001	7	59703001	59704000	1000	1	5.28E-06	-2.232255	8	0.8		
DMR7:76374001	7	76374001	76375000	1000	1	8.15E-06	-1.3065429	8	0.8	Ncald	Signaling
DMR7:81954001	7	81954001	81955000	1000	1	1.60E-05	-1.3116545	7	0.7	Rspo2	
DMR7:87890001	7	87890001	87891000	1000	1	8.42E-05	-1.4115	11	1.1	AABR07057765.1	
DMR7:87908001	7	87908001	87909000	1000	1	3.03E-05	0.7954608	19	1.9	AABR07057765.1	
DMR7:98338001	7	98338001	98339000	1000	1	4.48E-05	0.7279992	6	0.6	Fam91a1	Unknown
DMR7:115309001	7	115309001	115310000	1000	1	7.88E-05	-2.8147366	21	2.1		
DMR7:116061001	7	116061001	116062000	1000	1	3.70E-05	0.6477214	5	0.5	LOC100910068;Gml	
DMR7:125331001	7	125331001	125332000	1000	1	6.32E-05	0.7941471	45	4.5	Shisal1	
DMR7:127880001	7	127880001	127882000	2000	1	7.86E-05	0.7195392	15	0.75		
DMR7:130825001	7	130825001	130826000	1000	1	8.47E-05	-1.3911336	17	1.7	Syt10	Transport
DMR7:140127001	7	140127001	140128000	1000	1	8.42E-05	1.0671372	11	1.1	LOC103690317;AC103129.2	
DMR8:4344001	8	4344001	4346000	2000	1	5.00E-06	1.2394972	13	0.65	Vom2r18;Vom2r22	
DMR8:12424001	8	12424001	12425000	1000	1	1.05E-05	-1.3726712	7	0.7		
DMR8:12782001	8	12782001	12783000	1000	1	2.24E-05	-0.8766166	18	1.8		
DMR8:13128001	8	13128001	13129000	1000	1	5.60E-05	-0.5779305	11	1.1		
DMR8:22011001	8	22011001	22012000	1000	1	4.50E-05	-0.6247871	6	0.6	AC135310.1;Mrpl4	Transcription
DMR8:48629001	8	48629001	48630000	1000	1	8.74E-05	-0.847034	19	1.9	Abcg4;Hinfp	Transport;Transcription
DMR8:49839001	8	49839001	49840000	1000	1	9.00E-06	-1.3335761	11	1.1	Dscaml1	Development
DMR8:51656001	8	51656001	51660000	4000	1	5.88E-08	-1.4250927	44	1.1		
DMR8:58327001	8	58327001	58329000	2000	1	1.67E-05	0.6220956	37	1.85		
DMR8:60871001	8	60871001	60872000	1000	1	4.07E-06	0.6812535	16	1.6	Peak1	
DMR8:80988001	8	80988001	80989000	1000	1	4.32E-05	-1.0345893	16	1.6	Wdr72	
DMR8:87110001	8	87110001	87112000	2000	1	5.56E-05	0.9731728	18	0.9	Col12a1	Cytoskeleton
DMR8:116920001	8	116920001	116921000	1000	1	6.65E-05	-0.6434691	14	1.4	Bsn	Extracellular Matrix
DMR8:128377001	8	128377001	128378000	1000	1	9.18E-06	0.6527829	27	2.7	Scn10a	Transport
DMR8:131575001	8	131575001	131577000	2000	1	9.32E-05	-1.6202984	20	1		
DMR9:9933001	9	9933001	9934000	1000	1	8.72E-05	-1.6950265	27	2.7	AABR07066510.1	
DMR9:10220001	9	10220001	10222000	2000	1	9.86E-05	-1.1267321	25	1.25	Rfx2	Transcription
DMR9:53941001	9	53941001	53943000	2000	1	5.54E-06	-0.8632152	22	1.1	Nab1	Transcription
DMR9:64110001	9	64110001	64111000	1000	1	2.65E-05	-0.7678463	5	0.5	Gm22371;Maip1	
DMR9:65174001	9	65174001	65175000	1000	1	5.86E-06	-1.9830141	6	0.6	Aox2;AC128084.1;Aox4	
DMR9:70937001	9	70937001	70938000	1000	1	1.82E-05	0.7222847	9	0.9	AABR07067885.1	
DMR9:73430001	9	73430001	73431000	1000	1	9.13E-05	-0.8934301	13	1.3	Map2	Cytoskeleton
DMR9:88149001	9	88149001	88150000	1000	1	5.17E-05	-1.3652967	16	1.6	Rhbdd1	Protease
DMR9:93521001	9	93521001	93522000	1000	1	3.20E-05	-1.3506896	12	1.2		
DMR9:98386001	9	98386001	98387000	1000	1	2.01E-05	-0.7304221	5	0.5		
DMR9:100546001	9	100546001	100547000	1000	1	5.37E-05	-1.7713182	18	1.8	Hdlbp;Ano7	Metabolism
DMR9:116591001	9	116591001	116592000	1000	1	1.32E-05	0.7405799	20	2		
DMR10:2586001	10	2586001	2587000	1000	1	3.96E-05	-1.7374107	3	0.3		
DMR10:10901001	10	10901001	10903000	2000	1	8.14E-05	0.8158714	34	1.7	Ubald1;AABR07072078.2	
DMR10:28213001	10	28213001	28214000	1000	1	7.73E-05	-0.6868962	14	1.4		
DMR10:35897001	10	35897001	35898000	1000	1	8.29E-05	-1.5972991	10	1	Rufy1	Transcription
DMR10:56215001	10	56215001	56216000	1000	1	8.34E-05	-1.0565662	6	0.6	Atp1b2;Shbg	Metabolism;Binding Protein
DMR10:69494001	10	69494001	69495000	1000	1	5.56E-05	1.0675052	5	0.5		
DMR10:101860001	10	101860001	101861000	1000	1	5.48E-05	-0.6252105	12	1.2	Slc39a11	Metabolism
DMR10:110684001	10	110684001	110685000	1000	1	7.22E-05	-1.0629979	9	0.9	Tbcd	EST
DMR11:33787001	11	33787001	33789000	2000	1	9.19E-05	-1.1160369	38	1.9	Setd4;U4	
DMR11:68855001	11	68855001	68856000	1000	1	9.37E-05	-0.7476734	6	0.6		
DMR11:74178001	11	74178001	74179000	1000	1	5.15E-05	-0.7966847	23	2.3		
DMR11:78854001	11	78854001	78855000	1000	1	2.80E-05	-0.8981623	7	0.7	Tprg1	Unknown
DMR11:87454001	11	87454001	87455000	1000	1	9.86E-05	-0.9128929	12	1.2	P2rx6;Slc7a4	Receptor;Metabolism
DMR12:2545001	12	2545001	2546000	1000	1	2.39E-05	-0.8124154	10	1	Ctxn1;Snapc2;Tgfbr3l;Map2k7	Translation;Signaling
DMR12:13105001	12	13105001	13107000	2000	1	9.17E-05	-0.833368	27	1.35	Rac1;Daglb	Signaling
DMR12:23748001	12	23748001	23749000	1000	1	5.27E-05	-1.6262701	14	1.4	Zp3;Ssc4d	Receptor
DMR12:34737001	12	34737001	34738000	1000	1	6.78E-05	-0.4410242	8	0.8		
DMR12:38818001	12	38818001	38821000	3000	1	4.46E-07	-0.6911558	49	1.633	Hpd	Metabolism
DMR12:41475001	12	41475001	41476000	1000	1	6.91E-05	-1.4354037	12	1.2	Cfap73;Ddx54	Transcription
DMR12:50705001	12	50705001	50707000	2000	1	9.09E-05	-1.2303042	38	1.9		
DMR13:16354001	13	16354001	16355000	1000	1	1.58E-05	0.9331255	6	0.6		
DMR13:20176001	13	20176001	20177000	1000	1	5.30E-05	0.8093746	2	0.2		
DMR13:30848001	13	30848001	30849000	1000	1	5.06E-05	-0.9823742	2	0.2		
DMR13:36250001	13	36250001	36251000	1000	1	1.19E-06	0.8170114	13	1.3	Steap3	
DMR13:54407001	13	54407001	54410000	3000	1	9.18E-05	0.8433224	29	0.967		

DMR13:66502001	13	66502001	66503000	1000	1	8.97E-05	0.8732596	5	0.5		
DMR13:73057001	13	73057001	73058000	1000	1	4.89E-05	-1.3358523	6	0.6	Xpr1	Receptor
DMR13:73360001	13	73360001	73362000	2000	1	8.80E-05	-0.8944737	13	0.65	Lhx4	Transcription
DMR13:81668001	13	81668001	81669000	1000	1	8.50E-05	0.9808141	11	1.1		
DMR13:93930001	13	93930001	93931000	1000	1	8.37E-05	0.818125	9	0.9	AABR07021823.2;Exo1	Transcription
DMR13:94808001	13	94808001	94809000	1000	1	5.74E-05	0.67667	7	0.7	AABR07021840.1	
DMR13:98959001	13	98959001	98960000	1000	1	6.72E-05	-1.6256641	11	1.1	Lin9	Transcription
DMR13:105222001	13	105222001	105223000	1000	1	9.17E-05	0.7281162	26	2.6		
DMR13:105444001	13	105444001	105445000	1000	1	4.65E-06	-1.3993297	29	2.9		
DMR13:106108001	13	106108001	106109000	1000	1	7.84E-06	-1.566714	13	1.3		
DMR14:5209001	14	5209001	5210000	1000	1	1.17E-05	-1.64715	21	2.1		
DMR14:9523001	14	9523001	9524000	1000	1	1.44E-05	0.7147318	14	1.4	AABR07014323.1	
DMR14:29692001	14	29692001	29693000	1000	1	1.21E-05	1.1050523	9	0.9		
DMR14:33409001	14	33409001	33410000	1000	1	9.08E-05	-1.5035647	11	1.1	Thegl	
DMR14:33462001	14	33462001	33464000	2000	1	5.13E-05	0.8557112	9	0.45	LOC108348180	
DMR14:37137001	14	37137001	37138000	1000	1	1.05E-05	0.7248337	8	0.8	Sgcb;Lrrc66	Cytoskeleton;Receptor
DMR14:82454001	14	82454001	82455000	1000	1	6.31E-06	-1.0487829	6	0.6		
DMR14:104565001	14	104565001	104566000	1000	1	6.73E-05	-0.6654565	13	1.3		
DMR14:111477001	14	111477001	111478000	1000	1	7.13E-05	-0.9215365	4	0.4	AABR07016703.1	
DMR15:6903001	15	6903001	6904000	1000	1	5.06E-05	-1.0299065	0	0		
DMR15:22682001	15	22682001	22683000	1000	1	6.94E-05	-0.5739401	12	1.2		
DMR15:33157001	15	33157001	33158000	1000	1	3.91E-05	-1.5220316	16	1.6	Prmt5	Signaling
DMR15:33822001	15	33822001	33823000	1000	1	4.73E-05	-1.0310879	21	2.1	RGD1564324	
DMR15:42951001	15	42951001	42952000	1000	1	5.42E-05	1.0492301	4	0.4	Ptk2b;Mir6320;Trim35	Signaling;Metabolism
DMR15:59887001	15	59887001	59888000	1000	1	1.41E-06	1.3082857	4	0.4	Enox1	Transcription
DMR15:60666001	15	60666001	60667000	1000	1	5.75E-05	0.7479224	9	0.9		
DMR15:61794001	15	61794001	61795000	1000	1	9.65E-05	0.7353834	14	1.4		
DMR15:67824001	15	67824001	67825000	1000	1	8.70E-05	1.0423086	4	0.4		
DMR15:80263001	15	80263001	80264000	1000	1	6.98E-05	-1.0367334	13	1.3	Klhl1	Cytoskeleton
DMR15:81394001	15	81394001	81395000	1000	1	3.85E-05	0.9941129	3	0.3		
DMR15:82773001	15	82773001	82775000	2000	1	9.88E-05	0.6884848	30	1.5		
DMR15:93629001	15	93629001	93630000	1000	1	6.13E-05	0.7474931	25	2.5	Mycbp2;Acod1;U6;AABR07019209.2	Metabolism
DMR15:100817001	15	100817001	100818000	1000	1	2.76E-05	0.7641495	6	0.6		
DMR15:108660001	15	108660001	108664000	4000	1	9.23E-05	1.7653233	88	2.2	Clybl	Metabolism
DMR16:5078001	16	5078001	5079000	1000	1	3.63E-05	-0.7364474	9	0.9	Cacna2d3;5S_rRNA;LOC102547963	Transport;Receptor
DMR16:9711001	16	9711001	9712000	1000	1	8.21E-05	0.8812982	14	1.4	Mapk8	Signaling
DMR16:19213001	16	19213001	19214000	1000	1	9.37E-05	-1.1020897	20	2	Eps15l1;Klf2	Signaling
DMR16:19938001	16	19938001	19939000	1000	1	3.48E-05	-1.3123136	16	1.6	Bst2	Signaling
DMR16:26341001	16	26341001	26342000	1000	1	1.64E-05	0.8516476	15	1.5		
DMR16:30107001	16	30107001	30108000	1000	1	5.00E-06	-0.5307036	7	0.7		
DMR16:30578001	16	30578001	30579000	1000	1	2.10E-06	-0.638079	11	1.1		
DMR16:30812001	16	30812001	30813000	1000	1	5.01E-05	-0.6812971	5	0.5		
DMR16:67006001	16	67006001	67008000	2000	1	6.25E-05	0.7889991	21	1.05		
DMR17:27562001	17	27562001	27563000	1000	1	8.04E-05	-0.9389061	12	1.2	Rreb1	Signaling
DMR17:32956001	17	32956001	32957000	1000	1	6.80E-06	-2.0278854	15	1.5	Wrnip1;AABR07027478.1	
DMR17:36361001	17	36361001	36362000	1000	1	2.82E-05	-1.7416346	8	0.8	E2f3	Transcription
DMR17:37766001	17	37766001	37767000	1000	1	1.17E-05	0.7072538	14	1.4	AABR07027581.1	
DMR17:52762001	17	52762001	52763000	1000	1	1.16E-06	0.6903375	9	0.9	AABR07028009.1	
DMR17:72840001	17	72840001	72841000	1000	1	3.37E-05	-1.6527607	7	0.7		
DMR17:74196001	17	74196001	74197000	1000	1	4.49E-05	0.8103864	11	1.1		
DMR17:76606001	17	76606001	76608000	2000	1	2.94E-05	-1.1153023	21	1.05	Camk1d	Signaling
DMR17:77094001	17	77094001	77096000	2000	1	1.85E-05	-1.0227174	28	1.4	Ccdc3;AC105577.1	Transcription
DMR17:82447001	17	82447001	82448000	1000	1	9.40E-05	1.1284241	7	0.7	Malrd1	
DMR17:88760001	17	88760001	88761000	1000	1	1.33E-05	1.0135017	9	0.9		
DMR18:12751001	18	12751001	12754000	3000	1	2.69E-05	0.690109	33	1.1		
DMR18:81404001	18	81404001	81405000	1000	1	3.94E-05	0.7466615	9	0.9	Zfp407	Transcription
DMR19:10743001	19	10743001	10744000	1000	1	6.53E-05	-1.1948445	10	1	Pllp;Arl2bp	Unknown
DMR19:16269001	19	16269001	16270000	1000	1	8.07E-05	-1.327457	16	1.6		
DMR19:29976001	19	29976001	29977000	1000	1	6.93E-07	1.1425596	4	0.4	Inpp4b	Signaling
DMR19:32752001	19	32752001	32753000	1000	1	7.71E-06	-1.776709	12	1.2		
DMR19:58066001	19	58066001	58067000	1000	1	4.73E-05	-0.7708003	31	3.1	Disc1	
DMR19:58266001	19	58266001	58267000	1000	1	3.65E-05	-1.4504565	15	1.5	Sipa1l2	Signaling
DMR20:7390001	20	7390001	7391000	1000	1	1.20E-05	-0.873058	17	1.7	LOC294154	

DMR20:9867001	20	9867001	9868000	1000	1	1.60E-05	-0.7152167	5	0.5	Tff2	Signaling
DMR20:11561001	20	11561001	11562000	1000	1	7.94E-05	-1.4282447	17	1.7	RGD1561557;AABR07044583.1	EST
DMR20:24781001	20	24781001	24782000	1000	1	5.53E-05	-1.4672986	8	0.8		
DMR20:29868001	20	29868001	29870000	2000	1	3.16E-05	-1.0709268	34	1.7	Cdh23	Extracellular Matrix
DMRX:36262001	X	36262001	36263000	1000	1	5.23E-05	1.2067411	10	1		
DMRX:53984001	X	53984001	53985000	1000	1	2.53E-05	0.9674032	8	0.8		
DMRX:79269001	X	79269001	79270000	1000	1	8.94E-05	-1.2936469	25	2.5		
DMRX:83142001	X	83142001	83143000	1000	1	5.47E-05	1.1558337	5	0.5	Hdx	EST
DMRX:114990001	X	114990001	114991000	1000	1	6.70E-05	1.0134057	7	0.7	Pak3	Signaling
