# Supplementary material for: Epigenome-wide association study for atrazine induced transgenerational DNA methylation and histone retention sperm epigenetic biomarkers for disease
Source: PLoS One. 2020 Dec 16;15(12):e0239380. doi: 10.1371/journal.pone.0239380 (PMC7743986; doi:10.1371/journal.pone.0239380)
Supplement: S4 Table — DMR name, chromosome, start, stop, length, number signature windows, minimum p-value, max log-fold change, CpG number, CpG density, gene annotation, and gene category are presented. (PDF) [file pone.0239380.s011.pdf]

Supplemental Table S4
DMR Site List Testis Disease p<1e-04

DMR Name	Chr	Start	Stop	Length	# Sig Win	minP	maxLFC	CpG #	CpG Density	Gene Annotation	Gene Category
DMR1:2520001	1	2520001	2521000	1000	1	9.88E-06	-0.6026823	12	1.2	Ust	Metabolism
DMR1:4727001	1	4727001	4728000	1000	1	9.44E-05	-0.7568449	12	1.2	AABR07000207.1	
DMR1:10126001	1	10126001	10127000	1000	1	6.27E-05	0.8597653	5	0.5		
DMR1:14044001	1	14044001	14045000	1000	1	4.78E-05	-0.9487457	5	0.5		
DMR1:14466001	1	14466001	14468000	2000	1	2.60E-05	-0.6560972	27	1.35		
DMR1:24113001	1	24113001	24114000	1000	1	5.99E-05	-0.6851277	21	2.1		
DMR1:28177001	1	28177001	28178000	1000	1	2.61E-05	0.7694939	7	0.7		
DMR1:29209001	1	29209001	29210000	1000	1	6.58E-05	0.9198683	5	0.5	Hey2	Transcription
DMR1:31021001	1	31021001	31022000	1000	1	8.51E-05	0.9332828	6	0.6		
DMR1:32260001	1	32260001	32261000	1000	1	5.11E-05	-0.7538487	19	1.9	Tert	Transcription
DMR1:36932001	1	36932001	36934000	2000	1	6.60E-05	-0.6228924	18	0.9		
DMR1:39511001	1	39511001	39512000	1000	1	9.92E-05	0.6523471	4	0.4		
DMR1:40438001	1	40438001	40439000	1000	1	1.08E-05	-0.8305621	6	0.6	Plekhg1	Signaling
DMR1:40620001	1	40620001	40621000	1000	1	9.60E-05	-0.6831024	16	1.6	Mthfd1l	Metabolism
DMR1:53325001	1	53325001	53326000	1000	1	7.88E-06	0.9204895	11	1.1		
DMR1:61609001	1	61609001	61611000	2000	1	8.53E-05	1.1847916	7	0.35		
DMR1:65128001	1	65128001	65130000	2000	1	8.66E-08	0.8961119	8	0.4	Vom2r80	Receptor
DMR1:68985001	1	68985001	68986000	1000	1	2.54E-05	1.1650063	8	0.8	AABR07002209.1	
DMR1:75092001	1	75092001	75097000	5000	1	6.28E-05	0.8831217	64	1.28		
DMR1:75124001	1	75124001	75125000	1000	1	6.24E-05	0.7297828	6	0.6	Vom1r58	
DMR1:75126001	1	75126001	75129000	3000	1	6.66E-05	1.2653896	30	1	Vom1r58	
DMR1:75148001	1	75148001	75149000	1000	1	3.27E-05	1.4696728	7	0.7	RGD1564801	Signaling
DMR1:76331001	1	76331001	76339000	8000	1	2.72E-05	1.3841536	71	0.887	Sult2a6;AABR07002523.1	Metabolism
DMR1:76445001	1	76445001	76447000	2000	1	7.43E-05	0.9864633	19	0.95	Sult2a6	Metabolism
DMR1:76490001	1	76490001	76491000	1000	1	4.10E-06	1.0246145	9	0.9	Sult2a6	Metabolism
DMR1:76516001	1	76516001	76517000	1000	1	6.94E-05	1.0025237	16	1.6	Sult2a6	Metabolism
DMR1:76581001	1	76581001	76586000	5000	1	1.12E-06	0.9584254	21	0.42	Sult2a6	Metabolism
DMR1:76587001	1	76587001	76588000	1000	1	8.28E-05	1.046585	2	0.2	Sult2a6	Metabolism
DMR1:76591001	1	76591001	76595000	4000	1	4.58E-06	1.0553753	17	0.425	Sult2a6	Metabolism
DMR1:76605001	1	76605001	76610000	5000	1	2.36E-06	0.8570908	13	0.26	Sult2a6	Metabolism
DMR1:76613001	1	76613001	76614000	1000	1	2.21E-05	0.9193233	1	0.1	Sult2a6	Metabolism
DMR1:76679001	1	76679001	76681000	2000	1	2.92E-05	0.7860229	19	0.95	Sult2a6	Metabolism
DMR1:76720001	1	76720001	76721000	1000	1	4.28E-07	0.9252422	0	0	Sult2a6	Metabolism
DMR1:76761001	1	76761001	76764000	3000	2	3.51E-05	1.2023786	13	0.433	Sult2a6;U6	Metabolism
DMR1:76957001	1	76957001	76959000	2000	1	3.51E-05	1.130323	16	0.8		
DMR1:76982001	1	76982001	76984000	2000	1	1.27E-05	0.8259451	14	0.7		
DMR1:76986001	1	76986001	76988000	2000	1	1.28E-05	0.9898934	10	0.5		
DMR1:77087001	1	77087001	77090000	3000	1	7.76E-08	1.3435121	10	0.333		
DMR1:77197001	1	77197001	77199000	2000	1	6.58E-05	0.6093224	22	1.1		
DMR1:77268001	1	77268001	77270000	2000	1	7.01E-05	0.607004	18	0.9		
DMR1:77278001	1	77278001	77285000	7000	1	2.97E-05	1.0455965	53	0.757		
DMR1:77564001	1	77564001	77569000	5000	1	6.25E-08	0.9314344	48	0.96		
DMR1:81525001	1	81525001	81527000	2000	1	8.09E-05	-1.0159106	21	1.05	Tex101	Development
DMR1:82160001	1	82160001	82162000	2000	1	9.02E-05	-0.6295671	42	2.1	Cic;Pafah1b3;Prr19	Transcription;Metabolism
DMR1:82457001	1	82457001	82458000	1000	1	3.71E-05	-0.5985658	27	2.7	Bckdha;Exosc5;Tmem91	Metabolism
DMR1:82517001	1	82517001	82519000	2000	1	8.16E-06	0.618366	31	1.55	Ccdc97;Hnrnpul1	Transcription
DMR1:90868001	1	90868001	90869000	1000	1	1.12E-06	-1.3103989	12	1.2	AABR07002969.3;AABR07002969.2	
DMR1:90884001	1	90884001	90886000	2000	1	4.48E-05	-2.0610786	35	1.75		
DMR1:91580001	1	91580001	91582000	2000	1	1.09E-05	-1.0944479	13	0.65	Gpatch1;LOC687679	Transcription
DMR1:92333001	1	92333001	92334000	1000	1	3.12E-05	-2.059148	22	2.2	AABR07003001.2	
DMR1:92841001	1	92841001	92842000	1000	1	3.93E-05	-0.7642675	16	1.6		
DMR1:99370001	1	99370001	99372000	2000	1	6.90E-07	-0.9698888	23	1.15	AABR07003224.1	
DMR1:101410001	1	101410001	101411000	1000	1	8.03E-05	-1.2714258	19	1.9	Kcna7;Ntf4;Lhb;Ruvbl2	Transport;Signaling;Transcription
DMR1:104533001	1	104533001	104534000	1000	1	7.99E-05	-0.6672064	18	1.8		
DMR1:104693001	1	104693001	104694000	1000	1	3.44E-08	-1.2201046	17	1.7	Nav2	Development
DMR1:109801001	1	109801001	109802000	1000	1	2.68E-05	1.0604066	8	0.8		
DMR1:111561001	1	111561001	111563000	2000	1	4.89E-05	-0.6750784	15	0.75		
DMR1:129489001	1	129489001	129490000	1000	1	4.73E-07	0.7436184	13	1.3		
DMR1:133239001	1	133239001	133240000	1000	1	3.30E-05	0.6800866	4	0.4		
DMR1:138949001	1	138949001	138950000	1000	1	1.35E-05	1.046456	9	0.9		
DMR1:139470001	1	139470001	139471000	1000	1	1.92E-06	1.5956116	4	0.4		
DMR1:142911001	1	142911001	142912000	1000	1	6.17E-07	-0.8975449	16	1.6	Alpk3	Cytoskeleton
DMR1:145971001	1	145971001	145972000	1000	1	8.63E-05	-0.913418	11	1.1		
DMR1:150626001	1	150626001	150627000	1000	1	8.77E-06	-1.2179342	6	0.6		
DMR1:154233001	1	154233001	154234000	1000	1	6.73E-05	-0.6688146	15	1.5		
DMR1:159123001	1	159123001	159124000	1000	1	2.92E-05	0.6487402	3	0.3		
DMR1:159824001	1	159824001	159825000	1000	1	6.29E-05	-1.6163086	2	0.2		
DMR1:160919001	1	160919001	160921000	2000	1	5.90E-05	-1.5888765	15	0.75		
DMR1:165552001	1	165552001	165553000	1000	1	2.78E-05	-0.9178595	12	1.2	Coa4;AC110837.1	

DMR1:173250001	1	173250001	173251000	1000	1	1.51E-05	0.7794329	3	0.3	Gvin1	
DMR1:193233001	1	193233001	193234000	1000	1	6.95E-05	0.9099619	3	0.3	Slc5a11;Arhgap17	Transport;Signaling
DMR1:193581001	1	193581001	193582000	1000	1	7.72E-05	0.6676082	5	0.5		
DMR1:195362001	1	195362001	195363000	1000	1	6.36E-05	0.7807509	11	1.1		
DMR1:203230001	1	203230001	203232000	2000	1	5.47E-06	-0.8542935	34	1.7	AABR07005844.1	
DMR1:203994001	1	203994001	203995000	1000	1	8.70E-05	-0.8924607	17	1.7	Gpr26	Receptor
DMR1:204864001	1	204864001	204865000	1000	1	9.99E-05	-1.077554	6	0.6	Eef1akmt2;Abraxas2	
DMR1:207548001	1	207548001	207551000	3000	1	8.53E-05	0.8405838	11	0.367		
DMR1:209595001	1	209595001	209597000	2000	1	5.66E-05	-1.1019564	36	1.8	Ebf3	
DMR1:214667001	1	214667001	214668000	1000	1	3.14E-05	-1.6521622	22	2.2	AABR07006030.1	
DMR1:218426001	1	218426001	218427000	1000	1	4.90E-05	-0.6151727	11	1.1	Tpcn2	Transport
DMR1:218515001	1	218515001	218516000	1000	1	4.35E-05	-0.8811969	15	1.5	Ighmbp2	Immune
DMR1:222828001	1	222828001	222830000	2000	1	4.83E-05	0.8858828	13	0.65		
DMR1:230821001	1	230821001	230822000	1000	1	1.24E-05	1.2074588	4	0.4	Olr384	Receptor
DMR1:241331001	1	241331001	241332000	1000	1	4.33E-05	0.8000964	7	0.7	RGD1560242	
DMR1:243983001	1	243983001	243984000	1000	1	5.58E-05	0.8418495	6	0.6		
DMR1:255379001	1	255379001	255380000	1000	1	9.22E-06	-0.7375562	12	1.2	Ppp1r3c	Signaling
DMR1:256159001	1	256159001	256161000	2000	1	2.49E-05	-1.0434109	24	1.2		
DMR1:267022001	1	267022001	267024000	2000	1	1.04E-05	-0.9132665	50	2.5	Neurl1	Protease
DMR1:267222001	1	267222001	267224000	2000	1	8.66E-05	-0.6713782	43	2.15	Sh3pxd2a	Development
DMR1:273494001	1	273494001	273495000	1000	1	8.66E-06	0.8910877	11	1.1		
DMR1:274644001	1	274644001	274645000	1000	1	1.41E-06	1.2296068	10	1	Pdcd4;Bbip1	Transcription
DMR1:275074001	1	275074001	275075000	1000	1	8.91E-05	0.8837504	5	0.5		
DMR1:277294001	1	277294001	277295000	1000	1	2.58E-05	-0.8535881	31	3.1	Plekhs1	
DMR1:278041001	1	278041001	278043000	2000	1	6.13E-05	-0.5416343	36	1.8	AABR07007032.1	
DMR1:278777001	1	278777001	278778000	1000	1	9.02E-06	0.8289133	8	0.8	Atrnl1	Signaling
DMR1:281670001	1	281670001	281671000	1000	1	3.00E-05	-0.896765	10	1		
DMR2:6793001	2	6793001	6794000	1000	1	6.64E-05	0.8129894	5	0.5		
DMR2:11726001	2	11726001	11730000	4000	1	5.74E-05	-0.6048496	52	1.3	Mef2c	Transcription
DMR2:20629001	2	20629001	20630000	1000	1	1.05E-05	0.7892498	7	0.7	Ssbp2	Transcription
DMR2:22392001	2	22392001	22393000	1000	1	9.46E-05	-0.7011542	22	2.2	Thbs4	Signaling
DMR2:22743001	2	22743001	22744000	1000	1	1.18E-06	-1.6425321	20	2	Cmya5;Tent2	
DMR2:23605001	2	23605001	23606000	1000	1	9.64E-05	0.7212354	16	1.6		
DMR2:26622001	2	26622001	26623000	1000	1	6.58E-05	-0.7122935	11	1.1	Sv2c	Development
DMR2:32812001	2	32812001	32813000	1000	1	1.50E-05	-1.4710019	11	1.1	Cd180	Immune
DMR2:41378001	2	41378001	41379000	1000	1	2.69E-05	-0.6588956	9	0.9	Pde4d	Metabolism
DMR2:44855001	2	44855001	44856000	1000	1	5.61E-05	-0.8824834	18	1.8	Ccno;Mcidas	Cell Cycle
DMR2:44961001	2	44961001	44962000	1000	1	7.43E-07	0.8214718	10	1	Gzma	Proteolysis
DMR2:53782001	2	53782001	53783000	1000	1	7.80E-06	-0.9626618	6	0.6		
DMR2:54791001	2	54791001	54793000	2000	1	2.33E-05	1.0177305	8	0.4		
DMR2:55320001	2	55320001	55321000	1000	1	2.39E-05	0.8630639	12	1.2	AABR07008386.1	
DMR2:66973001	2	66973001	66974000	1000	1	7.32E-05	-0.9257174	2	0.2	Cdh9	Extracellular Matrix
DMR2:79449001	2	79449001	79451000	2000	1	6.55E-05	1.0471424	11	0.55		
DMR2:83335001	2	83335001	83336000	1000	1	6.87E-05	1.0667193	7	0.7		
DMR2:87330001	2	87330001	87331000	1000	1	5.37E-05	0.954407	5	0.5		
DMR2:97695001	2	97695001	97696000	1000	1	6.00E-05	-1.0042443	17	1.7		
DMR2:100866001	2	100866001	100867000	1000	1	9.74E-05	1.0103548	2	0.2		
DMR2:102007001	2	102007001	102009000	2000	1	1.62E-05	0.6662852	17	0.85	AABR07009533.1	
DMR2:107547001	2	107547001	107548000	1000	1	2.94E-05	1.0182857	7	0.7		
DMR2:110080001	2	110080001	110081000	1000	1	6.84E-05	0.7413246	6	0.6		
DMR2:113646001	2	113646001	113647000	1000	1	3.35E-05	1.0913392	3	0.3	Pld1	Metabolism
DMR2:116439001	2	116439001	116440000	1000	1	3.27E-05	0.6368504	9	0.9	Terc	
DMR2:127971001	2	127971001	127972000	1000	1	6.03E-05	-0.875822	8	0.8	AABR07010180.1	
DMR2:130167001	2	130167001	130168000	1000	1	2.34E-05	0.8359785	6	0.6		
DMR2:144575001	2	144575001	144576000	1000	1	3.50E-07	0.99557	7	0.7	Ccdc169	
DMR2:151384001	2	151384001	151388000	4000	1	8.46E-05	0.5643852	30	0.75		
DMR2:161475001	2	161475001	161476000	1000	1	2.19E-05	-1.545121	2	0.2		
DMR2:163547001	2	163547001	163550000	3000	1	9.57E-05	0.5453919	19	0.633		
DMR2:165892001	2	165892001	165893000	1000	1	3.57E-05	0.76097	14	1.4		
DMR2:176464001	2	176464001	176465000	1000	1	3.86E-05	0.5993734	3	0.3		
DMR2:178424001	2	178424001	178426000	2000	1	7.60E-06	0.840232	15	0.75	Rxfp1	Receptor
DMR2:178848001	2	178848001	178850000	2000	1	3.66E-05	1.1384021	15	0.75		
DMR2:179160001	2	179160001	179161000	1000	1	1.23E-06	0.9508831	5	0.5		
DMR2:185680001	2	185680001	185681000	1000	1	8.41E-05	1.0416722	4	0.4	Lrba	Signaling
DMR2:188444001	2	188444001	188445000	1000	1	3.61E-05	-0.6695802	22	2.2	Pklr;Hcn3	Signaling;Receptor
DMR2:193601001	2	193601001	193602000	1000	1	8.24E-05	0.6888793	2	0.2	U6	
DMR2:193869001	2	193869001	193870000	1000	1	2.47E-05	1.0738796	5	0.5	S100a11	Receptor
DMR2:199207001	2	199207001	199210000	3000	1	9.94E-05	-0.7287068	17	0.567	LOC100909441	Cytoskeleton
DMR2:199395001	2	199395001	199401000	6000	1	6.04E-05	-0.5819166	114	1.9		
DMR2:201465001	2	201465001	201466000	1000	1	2.13E-05	-1.2002204	6	0.6		
DMR2:203982001	2	203982001	203983000	1000	1	7.35E-05	-0.5037781	23	2.3		
DMR2:212190001	2	212190001	212191000	1000	1	2.29E-05	-0.7615907	4	0.4		
DMR2:215357001	2	215357001	215359000	2000	1	7.36E-05	0.9245748	19	0.95		
DMR2:220677001	2	220677001	220679000	2000	1	7.71E-05	0.7977846	24	1.2		

DMR2:222756001	2	222756001	222757000	1000	1	1.10E-05	0.8529839	3	0.3	AABR07013085.1	
DMR2:227104001	2	227104001	227105000	1000	1	8.24E-05	1.0696483	7	0.7	LOC691807;Usp53	Proteolysis
DMR2:228086001	2	228086001	228087000	1000	1	7.69E-05	0.7472078	4	0.4		
DMR2:230266001	2	230266001	230268000	2000	1	3.53E-05	-0.8533909	19	0.95	Mcub	
DMR2:231916001	2	231916001	231917000	1000	1	5.97E-05	1.0831208	3	0.3	Zgrf1	
DMR2:241962001	2	241962001	241963000	1000	1	3.81E-05	0.6980526	6	0.6	Ppp3ca	Signaling
DMR2:246622001	2	246622001	246623000	1000	1	8.77E-05	0.6940376	14	1.4		
DMR2:247675001	2	247675001	247676000	1000	1	5.60E-05	0.7455516	12	1.2		
DMR2:250741001	2	250741001	250742000	1000	1	1.74E-05	0.614149	10	1	Sh3glb1	Signaling
DMR2:252967001	2	252967001	252969000	2000	1	5.62E-05	0.8433922	33	1.65		
DMR3:788001	3	788001	789000	1000	1	4.36E-06	-1.2428969	11	1.1		
DMR3:6983001	3	6983001	6984000	1000	1	8.33E-05	-0.5583691	8	0.8	AABR07051374.1	
DMR3:10428001	3	10428001	10430000	2000	1	1.18E-05	-0.9071391	38	1.9	AABR07072853.5	
DMR3:10901001	3	10901001	10903000	2000	1	4.35E-05	-1.2198022	44	2.2	AABR07051426.1	
DMR3:13317001	3	13317001	13318000	1000	1	8.63E-05	0.7706266	4	0.4	Pbx3	Transcription
DMR3:17336001	3	17336001	17337000	1000	1	6.68E-06	0.9004873	3	0.3	AABR07051578.1	
DMR3:19619001	3	19619001	19620000	1000	1	7.03E-05	1.0243707	6	0.6		
DMR3:20904001	3	20904001	20906000	2000	1	3.97E-05	1.0047564	10	0.5	Olr415;Olr416	
DMR3:29063001	3	29063001	29064000	1000	1	8.31E-06	1.0507705	6	0.6	Arhgap15	Signaling
DMR3:29799001	3	29799001	29800000	1000	1	4.45E-05	0.9645102	13	1.3		
DMR3:33936001	3	33936001	33937000	1000	1	4.15E-05	0.6820344	5	0.5	AABR07052048.2	
DMR3:36816001	3	36816001	36820000	4000	1	7.52E-05	0.7237822	43	1.075		
DMR3:37233001	3	37233001	37234000	1000	1	6.03E-05	1.0577775	8	0.8		
DMR3:43051001	3	43051001	43052000	1000	1	7.40E-05	0.8581358	13	1.3		
DMR3:43141001	3	43141001	43143000	2000	1	2.77E-05	-1.9746164	6	0.3		
DMR3:53567001	3	53567001	53570000	3000	1	5.88E-05	0.7471762	26	0.867	Xirp2	
DMR3:59637001	3	59637001	59638000	1000	1	5.00E-06	-0.974276	10	1	Sp3	Transcription
DMR3:72962001	3	72962001	72963000	1000	1	9.96E-06	1.0020873	2	0.2		
DMR3:73358001	3	73358001	73359000	1000	1	3.04E-05	-1.2097596	2	0.2	Olr472;Olr473	Receptor
DMR3:78692001	3	78692001	78693000	1000	1	6.51E-06	-1.1036248	1	0.1	AC105628.1	
DMR3:80526001	3	80526001	80527000	1000	1	9.75E-05	-1.2194893	4	0.4	Ckap5;F2	Cytoskeleton;Protease
DMR3:85119001	3	85119001	85120000	1000	1	9.75E-05	0.5959021	12	1.2		
DMR3:85595001	3	85595001	85596000	1000	1	8.62E-06	0.8626368	9	0.9	Lrrc4c	Extracellular Matrix
DMR3:87046001	3	87046001	87048000	2000	1	9.84E-05	0.6913641	17	0.85		
DMR3:99477001	3	99477001	99478000	1000	1	8.13E-05	1.0354736	5	0.5		
DMR3:103798001	3	103798001	103800000	2000	1	1.84E-05	0.9398569	13	0.65	Slc12a6	Transport
DMR3:111866001	3	111866001	111867000	1000	1	3.27E-05	-0.7627015	20	2	AABR07053509.2	
DMR3:113486001	3	113486001	113487000	1000	1	6.58E-06	-0.9382052	26	2.6	Wdr76;Frmd5	Signaling
DMR3:123526001	3	123526001	123527000	1000	1	6.87E-05	-0.6123731	7	0.7	Atrn	Signaling
DMR3:128461001	3	128461001	128463000	2000	1	7.21E-05	1.0875296	16	0.8		
DMR3:133047001	3	133047001	133048000	1000	1	2.89E-06	0.7669082	7	0.7	Tasp1	Metabolism
DMR3:133630001	3	133630001	133631000	1000	1	7.00E-05	-0.6128439	19	1.9		
DMR3:133797001	3	133797001	133798000	1000	1	1.30E-05	-0.8474543	7	0.7		
DMR3:136082001	3	136082001	136083000	1000	1	6.83E-06	1.0691405	6	0.6		
DMR3:149795001	3	149795001	149796000	1000	1	8.26E-05	-0.8663462	5	0.5	Cdk5rap1;AABR07054358.1;LOC690507	Signaling
DMR3:153134001	3	153134001	153135000	1000	1	3.42E-05	1.2059079	15	1.5	Soga1	
DMR3:153175001	3	153175001	153176000	1000	1	3.52E-05	-0.8345799	16	1.6	Soga1	
DMR3:153594001	3	153594001	153595000	1000	1	1.16E-05	-0.7846111	17	1.7	Src	Transcription
DMR3:157977001	3	157977001	157978000	1000	1	2.84E-05	0.7262981	13	1.3	Ptprt	Receptor
DMR3:159495001	3	159495001	159496000	1000	1	2.57E-05	0.9836427	11	1.1		
DMR3:162836001	3	162836001	162838000	2000	1	7.45E-06	-0.7996903	33	1.65	Sulf2	Metabolism
DMR3:163329001	3	163329001	163330000	1000	1	1.18E-05	-1.2061414	20	2	Prex1	
DMR3:167752001	3	167752001	167753000	1000	1	7.85E-06	0.7156055	6	0.6	AABR07054721.1	
DMR3:175054001	3	175054001	175055000	1000	1	7.96E-05	-0.9052164	11	1.1		
DMR4:533001	4	533001	534000	1000	1	1.20E-05	-0.8806113	13	1.3		
DMR4:1423001	4	1423001	1424000	1000	1	9.73E-05	-0.866043	15	1.5	Olr1230;Olr1231	
DMR4:3949001	4	3949001	3950000	1000	1	8.19E-06	1.0645269	15	1.5	Paxip1	
DMR4:7138001	4	7138001	7139000	1000	1	7.12E-05	-0.8142733	7	0.7	Abcf2;Iqca1l	Transport
DMR4:9641001	4	9641001	9642000	1000	1	1.75E-05	-0.7463347	19	1.9	Reln	Protease
DMR4:12404001	4	12404001	12405000	1000	1	9.84E-05	0.5844728	14	1.4	AABR07059258.1	
DMR4:16197001	4	16197001	16200000	3000	1	8.42E-05	-1.0085727	54	1.8		
DMR4:24278001	4	24278001	24281000	3000	1	5.24E-05	0.6571396	21	0.7		
DMR4:28815001	4	28815001	28816000	1000	1	2.24E-05	0.8879792	6	0.6		
DMR4:29896001	4	29896001	29897000	1000	1	6.85E-05	0.6783015	7	0.7		
DMR4:31758001	4	31758001	31759000	1000	1	4.12E-06	0.99773	13	1.3		
DMR4:34312001	4	34312001	34316000	4000	1	2.41E-05	0.765073	20	0.5	Glcci1	
DMR4:34328001	4	34328001	34329000	1000	1	1.97E-05	1.1757882	5	0.5	Glcci1	
DMR4:45820001	4	45820001	45821000	1000	1	2.27E-06	0.8493528	6	0.6		
DMR4:49541001	4	49541001	49542000	1000	1	4.00E-05	-0.7341962	47	4.7		
DMR4:49916001	4	49916001	49917000	1000	1	7.21E-05	0.6774497	6	0.6		
DMR4:52399001	4	52399001	52400000	1000	1	7.96E-05	0.8991609	3	0.3		
DMR4:52693001	4	52693001	52694000	1000	1	1.91E-06	1.2641277	7	0.7		
DMR4:56717001	4	56717001	56719000	2000	1	1.87E-05	-0.6586862	45	2.25	Flnc	Cytoskeleton
DMR4:61623001	4	61623001	61624000	1000	1	3.63E-05	0.6188526	12	1.2		

DMR4:65126001	4	65126001	65127000	1000	1	4.86E-06	0.9246725	6	0.6	Akr1d1	Metabolism
DMR4:69079001	4	69079001	69080000	1000	1	3.81E-05	-0.9606972	2	0.2		
DMR4:74293001	4	74293001	74294000	1000	1	6.29E-06	1.0457315	6	0.6		
DMR4:79146001	4	79146001	79147000	1000	1	8.42E-05	-0.8062455	7	0.7	Stk31	Transcription
DMR4:94763001	4	94763001	94765000	2000	1	1.43E-05	0.8925978	10	0.5	Grid2	Signaling
DMR4:99801001	4	99801001	99802000	1000	1	1.85E-05	0.8994012	3	0.3	Immt;Ptcd3;Gm22486	Unknown
DMR4:102980001	4	102980001	102981000	1000	1	4.02E-05	0.8054052	11	1.1	AABR07061030.1	
DMR4:108146001	4	108146001	108147000	1000	1	5.35E-05	0.8253675	7	0.7		
DMR4:109502001	4	109502001	109503000	1000	1	9.23E-06	0.7028798	6	0.6	Reg1a	Signaling
DMR4:116679001	4	116679001	116680000	1000	1	4.05E-05	-0.9655799	4	0.4	Exoc6b	Transport
DMR4:130142001	4	130142001	130144000	2000	1	6.90E-05	0.6428073	35	1.75		
DMR4:133363001	4	133363001	133365000	2000	1	1.98E-06	-0.9736044	27	1.35		
DMR4:135619001	4	135619001	135620000	1000	1	1.57E-05	-0.7087541	21	2.1		
DMR4:135771001	4	135771001	135772000	1000	1	4.57E-07	0.9741505	6	0.6		
DMR4:137401001	4	137401001	137403000	2000	1	6.94E-06	0.6542071	13	0.65		
DMR4:147288001	4	147288001	147289000	1000	1	1.80E-05	0.8886701	11	1.1	Pparg	Receptor
DMR4:157979001	4	157979001	157980000	1000	1	4.38E-05	-0.52056	26	2.6	Cd9	Extracellular Matrix
DMR4:161765001	4	161765001	161767000	2000	1	3.26E-05	0.9207393	22	1.1	Fkbp4	Protein Binding
DMR4:165431001	4	165431001	165433000	2000	1	7.62E-05	0.7403906	16	0.8	Klra2	Immune
DMR4:166288001	4	166288001	166290000	2000	1	3.12E-05	0.7888533	13	0.65		
DMR4:167451001	4	167451001	167453000	2000	1	7.14E-05	0.8295651	8	0.4	Prr4	
DMR4:172225001	4	172225001	172226000	1000	1	2.36E-05	1.1193627	2	0.2		
DMR4:174872001	4	174872001	174873000	1000	1	4.49E-06	-0.7317549	17	1.7	Aebp2	Transcription
DMR4:176418001	4	176418001	176420000	2000	1	5.39E-05	0.6608135	13	0.65	7SK	
DMR4:180928001	4	180928001	180930000	2000	1	3.51E-05	-0.5789976	37	1.85	Tm7sf3	Unknown
DMR5:17052001	5	17052001	17053000	1000	1	4.49E-05	0.8475632	7	0.7	Penk;AABR07047044.1;LOC100912510	Signaling
DMR5:18268001	5	18268001	18269000	1000	1	5.69E-05	0.7285496	4	0.4		
DMR5:29739001	5	29739001	29740000	1000	1	7.32E-05	0.7980463	12	1.2		
DMR5:30414001	5	30414001	30415000	1000	1	8.80E-05	1.0463077	7	0.7		
DMR5:32310001	5	32310001	32312000	2000	1	1.26E-05	0.7777557	7	0.35		
DMR5:32518001	5	32518001	32519000	1000	1	8.18E-05	0.7989657	4	0.4		
DMR5:33461001	5	33461001	33466000	5000	1	3.93E-05	1.1486412	27	0.54	Cngb3	Receptor
DMR5:36723001	5	36723001	36724000	1000	1	6.49E-05	0.9939761	5	0.5		
DMR5:44269001	5	44269001	44270000	1000	1	1.29E-05	1.0253648	2	0.2	AABR07047714.1	
DMR5:46402001	5	46402001	46403000	1000	1	2.01E-05	0.8251671	7	0.7		
DMR5:46804001	5	46804001	46805000	1000	1	8.17E-05	0.6700538	12	1.2		
DMR5:48403001	5	48403001	48404000	1000	1	1.45E-05	-1.5301763	5	0.5	Gabrr1;U6;AABR07047799.1	Receptor
DMR5:54500001	5	54500001	54501000	1000	1	5.93E-05	0.8909154	3	0.3		
DMR5:55498001	5	55498001	55500000	2000	1	8.13E-05	0.7332563	9	0.45		
DMR5:57910001	5	57910001	57912000	2000	1	5.72E-05	-0.7752147	22	1.1	Fam219a	
DMR5:64191001	5	64191001	64193000	2000	1	6.37E-05	0.559394	9	0.45	Tex10	Development
DMR5:65884001	5	65884001	65885000	1000	1	9.81E-05	0.6856531	7	0.7		
DMR5:66535001	5	66535001	66537000	2000	1	2.52E-06	1.201018	8	0.4		
DMR5:68250001	5	68250001	68251000	1000	1	2.19E-06	0.9355956	16	1.6		
DMR5:70405001	5	70405001	70406000	1000	1	6.00E-05	0.9250395	8	0.8	Slc44a1	Metabolism
DMR5:78493001	5	78493001	78494000	1000	1	5.91E-05	-0.8260848	20	2	Rgs3;U4	
DMR5:79842001	5	79842001	79843000	1000	1	5.17E-05	0.6364725	11	1.1	Tnc	Extracellular Matrix
DMR5:80098001	5	80098001	80099000	1000	1	6.11E-07	0.9583837	3	0.3		
DMR5:87243001	5	87243001	87244000	1000	1	8.85E-05	-0.8087653	4	0.4		
DMR5:88976001	5	88976001	88977000	1000	1	4.88E-05	0.898281	4	0.4		
DMR5:98286001	5	98286001	98287000	1000	1	8.22E-05	0.7950606	6	0.6		
DMR5:103523001	5	103523001	103525000	2000	1	5.86E-05	-0.8242963	13	0.65	Sh3gl2	Signaling
DMR5:105076001	5	105076001	105078000	2000	1	3.50E-05	-0.6893558	18	0.9		
DMR5:107967001	5	107967001	107968000	1000	1	3.19E-05	0.8961837	8	0.8	AABR07049156.1	
DMR5:113691001	5	113691001	113692000	1000	1	4.44E-05	0.8015114	6	0.6	Ift74	Unknown
DMR5:115264001	5	115264001	115265000	1000	1	2.73E-05	0.7594028	2	0.2		
DMR5:118326001	5	118326001	118328000	2000	1	3.62E-05	1.0335554	12	0.6		
DMR5:124851001	5	124851001	124855000	4000	1	6.36E-05	-1.0573028	43	1.075		
DMR5:126428001	5	126428001	126430000	2000	1	1.61E-05	-0.7975831	14	0.7		
DMR5:127454001	5	127454001	127456000	2000	1	7.99E-05	-0.5151108	43	2.15	Lrp8	Receptor
DMR5:129264001	5	129264001	129265000	1000	1	3.10E-05	-0.7846257	9	0.9	Ttc39a	Unknown
DMR5:130142001	5	130142001	130143000	1000	1	8.32E-05	0.7612596	10	1		
DMR5:138061001	5	138061001	138062000	1000	1	4.45E-05	-0.7081638	12	1.2		
DMR5:141183001	5	141183001	141184000	1000	1	8.45E-05	-1.1679231	18	1.8	Macf1	Cytoskeleton
DMR5:151503001	5	151503001	151504000	1000	1	2.17E-06	-0.8946291	24	2.4	Wdtc1	Development
DMR5:153224001	5	153224001	153226000	2000	1	3.00E-05	-0.7055083	33	1.65	Rhd;Tmem50a	Transport
DMR5:156552001	5	156552001	156554000	2000	1	7.92E-05	-1.1179317	36	1.8	Eif4g3	Transcription
DMR5:156619001	5	156619001	156621000	2000	1	5.45E-05	-0.7198553	47	2.35	Hp1bp3;Sh2d5;Kif17	Cytoskeleton
DMR5:162082001	5	162082001	162083000	1000	1	4.25E-05	-0.8901271	31	3.1		
DMR5:162102001	5	162102001	162104000	2000	1	9.78E-05	-0.7534457	74	3.7		
DMR5:162254001	5	162254001	162255000	1000	1	5.04E-06	-1.1348173	13	1.3	Oog1	
DMR5:165132001	5	165132001	165135000	3000	1	2.67E-06	-0.8922847	66	2.2		
DMR5:166596001	5	166596001	166599000	3000	1	9.36E-05	-0.9350159	85	2.833	Clstn1;Pik3cd	Metabolism;Signaling
DMR5:169693001	5	169693001	169694000	1000	1	7.21E-05	-0.7889387	33	3.3	Nphp4	Development

DMR5:171556001	5	171556001	171558000	2000	1	1.44E-05	-0.8389422	37	1.85	Megf6	Growth Factors & Cytokines
DMR6:1645001	6	1645001	1646000	1000	1	7.60E-05	-0.7134954	16	1.6		
DMR6:3732001	6	3732001	3735000	3000	1	1.24E-05	-1.070249	54	1.8	Thumpd2	Metabolism
DMR6:8380001	6	8380001	8381000	1000	1	4.02E-05	1.0260905	5	0.5		
DMR6:8785001	6	8785001	8786000	1000	1	4.80E-05	-1.0133922	14	1.4		
DMR6:9131001	6	9131001	9132000	1000	1	4.17E-05	-0.8759886	20	2		
DMR6:10144001	6	10144001	10145000	1000	1	5.51E-05	-1.1223576	20	2	Eif3h	Translation
DMR6:12627001	6	12627001	12628000	1000	1	2.54E-05	0.8611642	5	0.5		
DMR6:13950001	6	13950001	13951000	1000	1	7.14E-05	-0.8730591	32	3.2	Nrxn1	Receptor
DMR6:26307001	6	26307001	26308000	1000	1	8.86E-05	0.8242846	10	1	Zfp512;AABR07063276.1	Transcription
DMR6:26567001	6	26567001	26568000	1000	1	7.30E-05	-0.6799271	18	1.8	Gtf3c2	Transcription
DMR6:35272001	6	35272001	35273000	1000	1	6.34E-05	0.8684145	5	0.5		
DMR6:44367001	6	44367001	44368000	1000	1	3.84E-06	-0.9041623	36	3.6	Id2	Epigenetic
DMR6:45069001	6	45069001	45070000	1000	1	8.12E-05	0.9847939	8	0.8		
DMR6:47115001	6	47115001	47116000	1000	1	7.65E-05	0.9626066	11	1.1		
DMR6:47122001	6	47122001	47123000	1000	1	2.00E-05	-0.7904029	7	0.7		
DMR6:51219001	6	51219001	51220000	1000	1	4.71E-05	-0.8055154	9	0.9	Cog5	Golgi
DMR6:51468001	6	51468001	51469000	1000	1	4.57E-05	-0.8088128	13	1.3	Pik3cg	Signaling
DMR6:58869001	6	58869001	58870000	1000	1	1.72E-05	1.0805315	7	0.7		
DMR6:65372001	6	65372001	65373000	1000	1	6.73E-05	-0.6399044	21	2.1		
DMR6:71822001	6	71822001	71823000	1000	1	8.31E-06	1.0714239	15	1.5		
DMR6:74656001	6	74656001	74657000	1000	1	9.56E-05	1.0164468	16	1.6		
DMR6:79181001	6	79181001	79182000	1000	1	2.65E-05	-0.5893554	20	2	AC098459.3	
DMR6:92510001	6	92510001	92511000	1000	1	5.39E-06	-1.256957	19	1.9	Nin	Transcription
DMR6:97524001	6	97524001	97525000	1000	1	9.31E-05	0.7659497	9	0.9		
DMR6:108359001	6	108359001	108360000	1000	1	4.54E-05	-0.9415268	14	1.4	Vrtn	
DMR6:121396001	6	121396001	121397000	1000	1	7.90E-05	0.6762693	6	0.6		
DMR6:121477001	6	121477001	121478000	1000	1	2.07E-05	0.8793907	3	0.3		
DMR6:124111001	6	124111001	124112000	1000	1	4.85E-05	-1.4864592	22	2.2		
DMR6:131285001	6	131285001	131286000	1000	1	6.23E-05	0.9855779	12	1.2		
DMR6:133612001	6	133612001	133613000	1000	1	7.65E-06	-0.5727323	24	2.4		
DMR6:135181001	6	135181001	135182000	1000	1	1.35E-05	-0.8004866	22	2.2	Wdr20	
DMR6:136246001	6	136246001	136248000	2000	1	7.09E-05	-1.1334283	41	2.05	LOC103692719	
DMR6:144540001	6	144540001	144541000	1000	1	9.41E-05	-1.2280499	5	0.5	Ptprn2;7SK	Signaling
DMR7:3231001	7	3231001	3232000	1000	1	2.53E-08	-1.027197	26	2.6	Mmp19;Tmem198b;Dnajc14	Protease;Protein Binding
DMR7:12302001	7	12302001	12304000	2000	1	4.11E-05	-0.632097	47	2.35	Dazap1	
DMR7:14552001	7	14552001	14553000	1000	1	4.17E-05	-1.633511	1	0.1	Cyp4f17;Cyp4f5	
DMR7:17221001	7	17221001	17224000	3000	1	8.44E-05	0.4900442	18	0.6		
DMR7:17385001	7	17385001	17386000	1000	1	9.58E-05	0.827571	3	0.3		
DMR7:18485001	7	18485001	18487000	2000	1	2.92E-05	-0.6284285	38	1.9	Myo1f	Cytoskeleton
DMR7:19650001	7	19650001	19652000	2000	1	5.49E-05	0.7569265	6	0.3		
DMR7:38001001	7	38001001	38002000	1000	1	5.87E-05	0.8941696	3	0.3		
DMR7:38869001	7	38869001	38870000	1000	1	1.69E-05	1.4606296	4	0.4	Kera	Receptor
DMR7:40760001	7	40760001	40762000	2000	1	5.90E-05	1.053334	8	0.4		
DMR7:42315001	7	42315001	42316000	1000	1	5.50E-05	0.8730151	9	0.9	Kitlg	Growth Factors & Cytokines
DMR7:47833001	7	47833001	47834000	1000	1	7.46E-05	0.5707091	11	1.1		
DMR7:50432001	7	50432001	50433000	1000	1	7.37E-05	-0.8518798	6	0.6	Syt1	Transport
DMR7:51221001	7	51221001	51222000	1000	1	9.60E-05	0.7710502	6	0.6		
DMR7:53927001	7	53927001	53928000	1000	1	6.06E-06	-0.924434	7	0.7	Osbpl8	Receptor
DMR7:54320001	7	54320001	54321000	1000	1	5.65E-05	0.8114108	2	0.2		
DMR7:56358001	7	56358001	56361000	3000	1	1.86E-05	0.9113818	29	0.967		
DMR7:57694001	7	57694001	57695000	1000	1	3.24E-05	-1.5798707	5	0.5		
DMR7:59137001	7	59137001	59138000	1000	1	9.93E-05	-0.7524192	3	0.3	Ptprr;Taf7l2	Signaling
DMR7:62264001	7	62264001	62265000	1000	1	2.67E-05	0.6933919	13	1.3		
DMR7:76233001	7	76233001	76234000	1000	1	6.60E-05	-0.744768	13	1.3	Ncald	Signaling
DMR7:78759001	7	78759001	78760000	1000	1	8.46E-05	0.77215	10	1	Dpys	Metabolism
DMR7:87157001	7	87157001	87158000	1000	1	1.00E-05	0.7284798	10	1	AABR07057765.1	
DMR7:97415001	7	97415001	97416000	1000	1	9.54E-05	0.7862625	8	0.8	AABR07057997.1	
DMR7:102791001	7	102791001	102792000	1000	1	9.80E-05	-1.187355	7	0.7	AABR07058158.1	
DMR7:109181001	7	109181001	109184000	3000	1	5.05E-06	1.1910555	28	0.933	Zfat	Transcription
DMR7:121343001	7	121343001	121344000	1000	1	4.26E-05	-0.7077624	15	1.5	Syngr1;AC127784.5;Tab1	Development;Signaling
DMR7:124847001	7	124847001	124849000	2000	1	9.62E-05	-0.7076106	36	1.8	Efcab6	Signaling
DMR7:125677001	7	125677001	125678000	1000	1	3.06E-05	-0.7524572	21	2.1	Phf21b	Metabolism
DMR7:129306001	7	129306001	129307000	1000	1	2.87E-05	0.7773291	14	1.4		
DMR7:133131001	7	133131001	133135000	4000	1	8.85E-05	-0.7355674	64	1.6	AC096792.1	
DMR7:139802001	7	139802001	139804000	2000	1	7.32E-05	-0.8482078	25	1.25	Olr1102	
DMR7:143340001	7	143340001	143341000	1000	1	2.56E-05	1.0813776	14	1.4	Krt5;Krt71	Cytoskeleton
DMR7:143427001	7	143427001	143428000	1000	1	4.89E-05	0.8052602	10	1	Krt5;Krt2	Cytoskeleton
DMR7:143907001	7	143907001	143909000	2000	1	9.37E-05	-0.8048736	34	1.7	Espl1	Transcription
DMR7:145655001	7	145655001	145656000	1000	1	2.31E-06	0.9427234	4	0.4		
DMR8:8905001	8	8905001	8906000	1000	1	2.82E-05	1.1116845	8	0.8		
DMR8:10322001	8	10322001	10323000	1000	1	5.38E-06	-1.2078549	15	1.5		
DMR8:11583001	8	11583001	11584000	1000	1	4.49E-05	0.646919	9	0.9		
DMR8:16161001	8	16161001	16162000	1000	1	7.37E-05	0.6890992	3	0.3		

DMR8:20304001	8	20304001	20306000	2000	1	9.08E-05	1.0106501	5	0.25		
DMR8:25692001	8	25692001	25694000	2000	1	5.75E-05	-0.9989103	20	1		
DMR8:25851001	8	25851001	25852000	1000	1	2.90E-05	-0.7988251	11	1.1	Tbx20	Transcription
DMR8:27149001	8	27149001	27150000	1000	1	1.27E-05	1.1026987	8	0.8		
DMR8:40866001	8	40866001	40867000	1000	1	3.76E-06	0.9050909	9	0.9		
DMR8:46966001	8	46966001	46967000	1000	1	2.18E-05	0.9789598	9	0.9	Grik4	Signaling
DMR8:51963001	8	51963001	51964000	1000	1	6.13E-05	-0.7574592	13	1.3		
DMR8:64449001	8	64449001	64450000	1000	1	3.26E-07	0.9692375	2	0.2	Parp6	
DMR8:65410001	8	65410001	65411000	1000	1	4.23E-05	-0.7081126	15	1.5	AABR07070312.1	
DMR8:73523001	8	73523001	73525000	2000	1	4.35E-05	0.8020484	14	0.7	AABR07070518.1	
DMR8:75694001	8	75694001	75696000	2000	1	4.95E-05	-0.5512555	36	1.8	Anxa2;AABR07070580.1	Signaling
DMR8:77515001	8	77515001	77516000	1000	1	5.47E-05	0.7397835	10	1		
DMR8:86351001	8	86351001	86354000	3000	1	4.82E-05	1.071058	5	0.167		
DMR8:89418001	8	89418001	89419000	1000	1	8.42E-05	-0.7163495	11	1.1	Mei4	
DMR8:90142001	8	90142001	90145000	3000	1	3.11E-05	0.7687677	10	0.333		
DMR8:90471001	8	90471001	90472000	1000	1	3.81E-07	1.269922	5	0.5	Phip	
DMR8:91378001	8	91378001	91380000	2000	1	7.08E-05	-0.7002118	21	1.05	Ttk	Signaling
DMR8:92043001	8	92043001	92044000	1000	1	7.04E-05	-0.835649	15	1.5		
DMR8:95437001	8	95437001	95439000	2000	1	9.54E-05	1.0006787	27	1.35		
DMR8:97802001	8	97802001	97803000	1000	1	1.55E-05	-0.5955998	13	1.3		
DMR8:98170001	8	98170001	98171000	1000	1	6.00E-05	0.722875	3	0.3		
DMR8:102019001	8	102019001	102020000	1000	1	4.57E-05	-0.8148261	8	0.8		
DMR8:103539001	8	103539001	103540000	1000	1	4.18E-06	0.8016732	3	0.3	Trpc1	Transport
DMR8:108935001	8	108935001	108936000	1000	1	9.83E-05	-1.5169771	8	0.8		
DMR8:111478001	8	111478001	111481000	3000	1	9.57E-05	0.8390559	55	1.833		
DMR8:112600001	8	112600001	112601000	1000	1	8.44E-05	0.8401871	7	0.7	Uba5;Acad11	Proteolysis;Cytoskeleton
DMR8:114170001	8	114170001	114171000	1000	1	6.71E-05	1.2127989	10	1	LOC102552009	
DMR8:116251001	8	116251001	116252000	1000	1	6.54E-05	-1.2418642	5	0.5	Cacna2d2	Transport
DMR8:123596001	8	123596001	123597000	1000	1	5.42E-06	1.0861915	11	1.1		
DMR8:123636001	8	123636001	123638000	2000	1	9.50E-05	0.7939236	30	1.5		
DMR8:123852001	8	123852001	123853000	1000	1	2.05E-05	-1.0740822	15	1.5		
DMR8:124346001	8	124346001	124347000	1000	1	3.65E-05	-0.8981842	14	1.4	Tgfbr2	Growth Factors & Cytokines
DMR8:124620001	8	124620001	124621000	1000	1	8.15E-05	1.0439397	16	1.6		
DMR8:130912001	8	130912001	130913000	1000	1	1.34E-06	-1.134454	9	0.9	Ano10	Signaling
DMR9:5264001	9	5264001	5265000	1000	1	1.04E-06	1.4208134	10	1		
DMR9:6318001	9	6318001	6319000	1000	1	1.83E-06	0.726102	5	0.5		
DMR9:7368001	9	7368001	7371000	3000	1	4.46E-05	0.9395498	14	0.467		
DMR9:10446001	9	10446001	10447000	1000	1	7.55E-05	1.028575	49	4.9	Lonp1;Rpl36;Micos13;Safb	Protease;Translation;Signaling
DMR9:11352001	9	11352001	11354000	2000	1	8.18E-05	0.630289	9	0.45		
DMR9:16174001	9	16174001	16175000	1000	1	1.39E-05	-0.9769489	10	1		
DMR9:16629001	9	16629001	16632000	3000	1	4.91E-05	-0.5959644	58	1.933	Rrp36;Cul7	
DMR9:24772001	9	24772001	24773000	1000	1	6.16E-07	1.3602788	10	1		
DMR9:28135001	9	28135001	28136000	1000	1	1.51E-05	0.7386049	14	1.4		
DMR9:29283001	9	29283001	29284000	1000	1	4.52E-06	0.7570387	4	0.4		
DMR9:32532001	9	32532001	32533000	1000	1	1.77E-05	0.7771734	5	0.5	U6	
DMR9:36445001	9	36445001	36446000	1000	1	1.07E-05	0.8229692	8	0.8		
DMR9:38509001	9	38509001	38510000	1000	1	1.51E-05	-0.8050467	13	1.3		
DMR9:38868001	9	38868001	38869000	1000	1	4.47E-05	1.1148711	7	0.7		
DMR9:41523001	9	41523001	41524000	1000	1	3.50E-05	0.846954	6	0.6		
DMR9:48749001	9	48749001	48750000	1000	1	5.06E-05	0.8035122	7	0.7		
DMR9:50302001	9	50302001	50303000	1000	1	2.13E-05	0.8911286	10	1	Nck2	Cytoskeleton
DMR9:51708001	9	51708001	51711000	3000	1	5.53E-05	0.8763723	20	0.667		
DMR9:53983001	9	53983001	53984000	1000	1	3.76E-05	0.926027	7	0.7		
DMR9:57407001	9	57407001	57408000	1000	1	5.92E-05	0.5188728	10	1		
DMR9:60729001	9	60729001	60730000	1000	1	9.81E-05	0.7398796	9	0.9		
DMR9:62806001	9	62806001	62807000	1000	1	4.70E-06	1.2176223	7	0.7		
DMR9:63755001	9	63755001	63756000	1000	1	9.23E-05	-0.7595488	19	1.9		
DMR9:71438001	9	71438001	71440000	2000	1	5.25E-05	1.1370924	18	0.9	Ccnyl1;Fzd5	Cell Cycle;Receptor
DMR9:85027001	9	85027001	85028000	1000	1	1.99E-05	0.8016284	11	1.1		
DMR9:85196001	9	85196001	85197000	1000	1	2.03E-05	1.152133	5	0.5		
DMR9:96642001	9	96642001	96645000	3000	1	1.34E-05	-0.7402965	28	0.933	Agap1	Signaling
DMR9:103241001	9	103241001	103242000	1000	1	3.48E-05	1.0376194	14	1.4	AABR07068400.1	
DMR9:112816001	9	112816001	112817000	1000	1	3.28E-05	0.8068502	9	0.9		
DMR9:113558001	9	113558001	113559000	1000	1	1.75E-05	-1.005489	8	0.8	Ppp4r1	Signaling
DMR9:118750001	9	118750001	118751000	1000	1	7.27E-05	0.9531691	16	1.6	Dlgap1	Signaling
DMR10:13069001	10	13069001	13070000	1000	1	8.74E-06	-1.1358389	26	2.6	Pkmyt1;Paqr4;Kremen2	Signaling;Receptor
DMR10:25748001	10	25748001	25749000	1000	1	8.57E-05	-0.8558676	17	1.7		
DMR10:25815001	10	25815001	25816000	1000	1	5.65E-05	1.1397317	5	0.5		
DMR10:32782001	10	32782001	32783000	1000	1	6.79E-05	0.6279211	5	0.5		
DMR10:47260001	10	47260001	47261000	1000	1	7.52E-07	-0.8278173	17	1.7		
DMR10:54175001	10	54175001	54176000	1000	1	9.16E-05	0.9075148	14	1.4	Gas7	Transcription
DMR10:75932001	10	75932001	75933000	1000	1	7.45E-05	0.6137536	14	1.4	Msi2	Translation
DMR10:84418001	10	84418001	84419000	1000	1	1.21E-05	-0.7115957	22	2.2	Skap1	
DMR10:85766001	10	85766001	85768000	2000	1	4.16E-05	-0.6124898	23	1.15	Lasp1	Cytoskeleton

DMR10:88699001	10	88699001	88702000	3000	1	1.23E-05	-0.8644144	81	2.7	Stat5b	Transcription
DMR10:89011001	10	89011001	89012000	1000	1	3.35E-05	-0.9700298	14	1.4	Mlx;Psmc3ip;Retreg3	Transcription;Signaling
DMR10:90248001	10	90248001	90250000	2000	1	3.47E-05	-0.7436606	30	1.5	Atxn7l3;Ubtf	Transcription
DMR10:92938001	10	92938001	92939000	1000	1	6.46E-05	0.9722698	11	1.1		
DMR10:93243001	10	93243001	93245000	2000	1	4.40E-05	-0.6884987	10	0.5	AABR07030568.2;AABR07030568.4;AABR07030568.1	
DMR10:93633001	10	93633001	93635000	2000	1	8.66E-05	-0.6792174	28	1.4	10-Mar	
DMR10:93654001	10	93654001	93655000	1000	1	3.02E-05	-0.6709411	31	3.1	10-Mar	
DMR10:97778001	10	97778001	97779000	1000	1	4.89E-05	-0.6146777	32	3.2	Arsg	Metabolism
DMR10:102544001	10	102544001	102545000	1000	1	4.30E-05	-0.514127	16	1.6	Sdk2	Development
DMR10:103817001	10	103817001	103819000	2000	1	3.90E-06	-1.0279636	49	2.45	Grin2c;Fdxr	Signaling;Metabolism
DMR10:105113001	10	105113001	105115000	2000	1	5.70E-05	-0.7474227	43	2.15	Evpl;Srp68	Cytoskeleton;Transcription
DMR10:108024001	10	108024001	108025000	1000	1	5.43E-05	-1.1044296	25	2.5		
DMR10:108271001	10	108271001	108274000	3000	1	8.94E-05	-1.1652316	55	1.833	Tbc1d16	Signaling
DMR10:109197001	10	109197001	109201000	4000	1	3.11E-05	-0.8082385	81	2.025	Baiap2;Aatk;Mir3065	Receptor
DMR10:110411001	10	110411001	110415000	4000	1	1.45E-06	-1.757837	50	1.25	Ogfod3;Hexd;AC110474.2;Cybc1	
DMR11:3782001	11	3782001	3783000	1000	1	5.57E-05	0.9123192	5	0.5		
DMR11:7053001	11	7053001	7054000	1000	1	1.76E-05	-1.0809132	3	0.3		
DMR11:8250001	11	8250001	8251000	1000	1	7.38E-05	0.9141468	7	0.7		
DMR11:9012001	11	9012001	9014000	2000	1	6.69E-05	0.5788394	8	0.4		
DMR11:9026001	11	9026001	9027000	1000	1	1.79E-05	0.9771959	10	1		
DMR11:12910001	11	12910001	12911000	1000	1	5.77E-07	0.8806331	10	1		
DMR11:18202001	11	18202001	18203000	1000	1	6.07E-05	-0.8862437	4	0.4		
DMR11:31579001	11	31579001	31581000	2000	1	4.72E-06	-0.8796479	43	2.15	Il10rb	Receptor
DMR11:32013001	11	32013001	32014000	1000	1	4.29E-05	-0.832516	18	1.8	Itsn1	Signaling
DMR11:32075001	11	32075001	32076000	1000	1	5.96E-05	-0.8728406	7	0.7	Itsn1;Atp5po	Signaling
DMR11:32820001	11	32820001	32823000	3000	1	8.59E-05	-0.7390866	41	1.367	Runx1	Transcription
DMR11:33408001	11	33408001	33409000	1000	1	5.39E-05	1.1552598	5	0.5		
DMR11:36244001	11	36244001	36245000	1000	1	8.93E-05	-0.8870654	23	2.3		
DMR11:39770001	11	39770001	39771000	1000	1	5.90E-05	0.8284514	6	0.6	RGD1565472;U6	
DMR11:58850001	11	58850001	58851000	1000	1	7.56E-05	0.9921722	11	1.1	Lsamp	Extracellular Matrix
DMR11:67509001	11	67509001	67510000	1000	1	8.72E-06	0.6979145	11	1.1	AABR07034362.2	
DMR11:74627001	11	74627001	74628000	1000	1	6.64E-05	-1.4914711	15	1.5		
DMR11:74912001	11	74912001	74913000	1000	1	7.69E-09	1.3433333	8	0.8	Atp13a4	Transport
DMR11:77623001	11	77623001	77624000	1000	1	1.99E-05	-0.9086771	13	1.3		
DMR11:80134001	11	80134001	80135000	1000	1	8.36E-05	-1.1731411	5	0.5		
DMR11:83007001	11	83007001	83008000	1000	1	6.16E-05	-0.5227935	31	3.1		
DMR11:83629001	11	83629001	83630000	1000	1	2.38E-05	1.0867282	3	0.3		
DMR11:86670001	11	86670001	86671000	1000	1	3.99E-05	-0.8722501	9	0.9	Txnrd2	Metabolism
DMR12:5687001	12	5687001	5688000	1000	1	7.89E-05	-1.0298973	25	2.5	Fry	Development
DMR12:17254001	12	17254001	17255000	1000	1	3.40E-05	-1.0219972	21	2.1	Zfand2a	Transcription
DMR12:19659001	12	19659001	19660000	1000	1	3.56E-07	0.88046	9	0.9		
DMR12:23729001	12	23729001	23730000	1000	1	1.62E-05	-0.7015619	25	2.5	Dtx2;Zp3	Transcription;Receptor
DMR12:25365001	12	25365001	25366000	1000	1	2.57E-07	-0.8143	23	2.3	Gtf2ird1	Transcription
DMR12:26103001	12	26103001	26104000	1000	1	1.25E-05	1.2647991	9	0.9		
DMR12:28107001	12	28107001	28109000	2000	1	4.02E-05	-0.6443295	30	1.5	5S_rRNA	
DMR12:38834001	12	38834001	38837000	3000	1	2.76E-05	-0.7812983	47	1.567	Hpd;Setd1b	Metabolism
DMR12:39616001	12	39616001	39618000	2000	1	4.59E-05	-0.7479951	29	1.45	Anapc7	Cell Cycle
DMR12:40905001	12	40905001	40907000	2000	1	9.43E-05	-1.2326708	25	1.25	Ptpn11	
DMR12:40972001	12	40972001	40974000	2000	1	6.49E-05	-0.6306028	44	2.2	AABR07036376.1	
DMR12:42414001	12	42414001	42415000	1000	1	7.61E-05	-0.9094161	10	1		
DMR12:46448001	12	46448001	46449000	1000	1	5.88E-05	-0.7259432	23	2.3	Cit	Signaling
DMR12:46473001	12	46473001	46475000	2000	1	6.19E-05	-0.8262375	40	2	Cit	Signaling
DMR12:47781001	12	47781001	47783000	2000	1	8.58E-05	-0.8169114	37	1.85	Fam222a	Unknown
DMR12:49104001	12	49104001	49105000	1000	1	2.05E-05	-0.6343626	3	0.3		
DMR12:50598001	12	50598001	50599000	1000	1	8.45E-05	1.0838854	10	1		
DMR13:12355001	13	12355001	12356000	1000	1	7.01E-05	0.745148	10	1		
DMR13:26053001	13	26053001	26054000	1000	1	7.38E-06	1.0528758	8	0.8		
DMR13:29800001	13	29800001	29801000	1000	1	7.50E-05	1.1506238	9	0.9		
DMR13:34840001	13	34840001	34842000	2000	1	2.85E-05	-0.7232317	38	1.9	Gli2	Transcription
DMR13:40036001	13	40036001	40037000	1000	1	1.42E-05	1.3100357	8	0.8		
DMR13:41434001	13	41434001	41436000	2000	1	3.75E-05	1.2019778	20	1		
DMR13:45229001	13	45229001	45230000	1000	1	2.54E-05	-1.7495143	4	0.4	SNORA70	
DMR13:46876001	13	46876001	46878000	2000	1	7.38E-05	0.8214522	11	0.55	Thsd7b	Extracellular Matrix
DMR13:51167001	13	51167001	51169000	2000	1	4.80E-06	-0.8175611	32	1.6	Ppfia4	Signaling
DMR13:53817001	13	53817001	53818000	1000	1	9.62E-05	-0.6875915	10	1	Nr5a2	Receptor
DMR13:56113001	13	56113001	56114000	1000	1	9.46E-05	0.7389138	5	0.5	Dennd1b	
DMR13:59503001	13	59503001	59505000	2000	1	1.72E-06	1.0703597	14	0.7		
DMR13:60862001	13	60862001	60863000	1000	1	8.54E-05	1.0016383	8	0.8		
DMR13:66521001	13	66521001	66522000	1000	1	8.94E-06	1.0355769	4	0.4		
DMR13:66594001	13	66594001	66595000	1000	1	3.35E-05	-1.0473228	10	1	AABR07021337.1	
DMR13:66907001	13	66907001	66909000	2000	1	8.88E-05	0.748562	18	0.9		
DMR13:67533001	13	67533001	67534000	1000	1	2.31E-05	0.9259518	10	1		
DMR13:91983001	13	91983001	91984000	1000	1	5.52E-05	0.9655428	16	1.6	AABR07021804.1	
DMR13:94264001	13	94264001	94265000	1000	1	7.37E-05	0.7502435	9	0.9	Pld5	Signaling

DMR13:96931001	13	96931001	96932000	1000	1	5.71E-05	0.8648266	2	0.2	Kif26b	Cytoskeleton
DMR13:97855001	13	97855001	97856000	1000	1	4.58E-05	1.1652864	6	0.6	Cnst	
DMR13:107324001	13	107324001	107326000	2000	1	2.32E-05	-0.7946816	28	1.4	Ush2a	Extracellular Matrix
DMR13:108505001	13	108505001	108507000	2000	1	7.45E-05	-0.9227265	28	1.4		
DMR13:108872001	13	108872001	108873000	1000	1	3.53E-06	-1.1521018	18	1.8		
DMR13:109576001	13	109576001	109577000	1000	1	8.59E-07	0.9891437	11	1.1	Flvcr1	
DMR14:9149001	14	9149001	9151000	2000	1	4.40E-05	0.6801743	27	1.35	Wdfy3	Apoptosis
DMR14:10236001	14	10236001	10238000	2000	1	8.94E-05	0.6270901	27	1.35		
DMR14:13949001	14	13949001	13950000	1000	1	5.67E-05	0.9014813	10	1		
DMR14:13994001	14	13994001	13995000	1000	1	6.91E-05	0.7447619	12	1.2		
DMR14:29692001	14	29692001	29693000	1000	1	4.18E-05	1.0522105	9	0.9		
DMR14:32573001	14	32573001	32574000	1000	1	1.14E-06	1.218572	1	0.1		
DMR14:45014001	14	45014001	45016000	2000	1	3.79E-05	0.9536388	20	1	Tlr6	Receptor
DMR14:49482001	14	49482001	49483000	1000	1	9.64E-05	-0.8192244	7	0.7		
DMR14:58317001	14	58317001	58319000	2000	1	9.01E-05	-0.6506149	26	1.3		
DMR14:59932001	14	59932001	59934000	2000	1	1.72E-05	-0.8274055	15	0.75		
DMR14:61437001	14	61437001	61438000	1000	1	6.55E-05	-1.2140745	7	0.7		
DMR14:61568001	14	61568001	61569000	1000	1	8.92E-05	1.1574743	9	0.9		
DMR14:67131001	14	67131001	67132000	1000	1	7.67E-05	0.9951842	8	0.8	Slit2	Development
DMR14:69701001	14	69701001	69702000	1000	1	2.17E-06	0.8783657	4	0.4		
DMR14:70149001	14	70149001	70150000	1000	1	8.61E-05	0.8435981	11	1.1	Clrn2	
DMR14:78153001	14	78153001	78154000	1000	1	1.38E-05	0.8625038	2	0.2	Evc2	Development
DMR14:86250001	14	86250001	86252000	2000	1	3.66E-05	-0.5878002	34	1.7	Camk2b	Signaling
DMR14:88821001	14	88821001	88822000	1000	1	6.97E-05	-0.7696299	16	1.6	Tns3	Signaling
DMR14:90150001	14	90150001	90151000	1000	1	4.46E-05	0.6365212	8	0.8		
DMR14:91001001	14	91001001	91002000	1000	1	7.08E-05	0.5307444	13	1.3		
DMR14:95959001	14	95959001	95960000	1000	1	2.17E-05	-0.6023331	23	2.3		
DMR14:102792001	14	102792001	102794000	2000	1	5.75E-06	0.8608286	7	0.35		
DMR14:103131001	14	103131001	103132000	1000	1	2.03E-05	0.6379048	8	0.8		
DMR14:103366001	14	103366001	103367000	1000	1	9.25E-05	0.8081739	10	1	AABR07016556.1	
DMR14:103911001	14	103911001	103912000	1000	1	6.64E-06	-1.2237705	7	0.7		
DMR14:105617001	14	105617001	105619000	2000	1	4.36E-05	0.9394201	21	1.05		
DMR14:107023001	14	107023001	107024000	1000	1	7.82E-05	0.8751411	12	1.2	Ehbp1	Unknown
DMR14:110189001	14	110189001	110190000	1000	1	5.26E-05	-1.6782307	9	0.9	AABR07016674.1	
DMR15:613001	15	613001	614000	1000	1	4.66E-05	0.697115	3	0.3	Kcnma1	Metabolism
DMR15:5985001	15	5985001	5986000	1000	1	5.32E-06	1.2311195	11	1.1		
DMR15:7528001	15	7528001	7529000	1000	1	5.13E-05	0.7454711	3	0.3		
DMR15:12615001	15	12615001	12616000	1000	1	2.54E-05	0.7826712	8	0.8		
DMR15:17228001	15	17228001	17229000	1000	1	8.66E-05	1.2191241	9	0.9		
DMR15:27755001	15	27755001	27756000	1000	1	8.12E-05	-0.9450599	12	1.2	Parp2;Tep1	Metabolism;Transcription
DMR15:42074001	15	42074001	42076000	2000	1	1.14E-05	1.0511213	13	0.65		
DMR15:43188001	15	43188001	43189000	1000	1	6.16E-05	0.8075006	2	0.2		
DMR15:48198001	15	48198001	48199000	1000	1	3.06E-05	-1.4771543	7	0.7	Kif13b;Hmbox1	Cytoskeleton
DMR15:61492001	15	61492001	61493000	1000	1	5.58E-05	-0.9912544	24	2.4		
DMR15:62524001	15	62524001	62525000	1000	1	8.30E-05	-1.4670297	11	1.1	LOC306079	
DMR15:63687001	15	63687001	63688000	1000	1	2.74E-05	1.0696024	7	0.7		
DMR15:69574001	15	69574001	69575000	1000	1	7.83E-05	0.8336432	7	0.7	U6	
DMR15:70109001	15	70109001	70110000	1000	1	5.64E-05	0.6676237	12	1.2	Diaph3	Cytoskeleton
DMR15:73519001	15	73519001	73520000	1000	1	3.10E-05	0.8765804	3	0.3		
DMR15:74062001	15	74062001	74063000	1000	1	5.20E-05	-1.0886883	31	3.1		
DMR15:76320001	15	76320001	76321000	1000	1	3.90E-05	0.9720426	7	0.7		
DMR15:85964001	15	85964001	85965000	1000	1	2.56E-05	0.5924548	16	1.6	Tbc1d4	
DMR15:88065001	15	88065001	88066000	1000	1	4.22E-05	-0.5037123	17	1.7	Mycbp2	Metabolism
DMR15:90300001	15	90300001	90301000	1000	1	9.00E-05	0.9361332	8	0.8	Mycbp2	Metabolism
DMR15:92507001	15	92507001	92508000	1000	1	3.00E-05	0.78616	7	0.7	Mycbp2	Metabolism
DMR15:102413001	15	102413001	102414000	1000	1	4.57E-07	0.9240847	7	0.7	Gpc6	Extracellular Matrix
DMR15:102932001	15	102932001	102933000	1000	1	7.61E-05	1.0240304	4	0.4	Gpc6	Extracellular Matrix
DMR15:106279001	15	106279001	106280000	1000	1	3.46E-05	-0.9151989	10	1	Ipo5	Transport
DMR15:108845001	15	108845001	108846000	1000	1	4.28E-05	0.7543816	8	0.8	Clybl	Metabolism
DMR16:57001	16	57001	58000	1000	1	4.93E-05	0.7321017	8	0.8		
DMR16:627001	16	627001	628000	1000	1	8.08E-05	0.6710844	2	0.2	AABR07024473.2;AABR07024473.1	
DMR16:1942001	16	1942001	1943000	1000	1	2.63E-05	-1.0813899	17	1.7	Zmiz1	Metabolism
DMR16:6598001	16	6598001	6601000	3000	1	2.49E-05	-0.66911	66	2.2	U6;Tkt	Metabolism
DMR16:7165001	16	7165001	7166000	1000	1	9.35E-10	0.8544289	5	0.5	Pbrm1	Unknown
DMR16:8181001	16	8181001	8182000	1000	1	8.65E-05	-0.6451272	13	1.3	Galnt15	Metabolism
DMR16:13457001	16	13457001	13458000	1000	1	4.86E-05	0.5806038	9	0.9		
DMR16:23604001	16	23604001	23605000	1000	1	4.81E-06	0.9811923	9	0.9	Psd3	Signaling
DMR16:34729001	16	34729001	34730000	1000	1	8.80E-05	0.9134534	8	0.8		
DMR16:37565001	16	37565001	37566000	1000	1	9.50E-05	0.6413062	6	0.6		
DMR16:50118001	16	50118001	50121000	3000	1	2.07E-05	0.7502504	28	0.933	Cyp4v3	Metabolism
DMR16:56045001	16	56045001	56046000	1000	1	3.28E-05	-1.1072386	9	0.9	AABR07025953.1	
DMR16:70274001	16	70274001	70276000	2000	1	7.02E-05	-0.6511438	41	2.05		
DMR16:71674001	16	71674001	71675000	1000	1	6.48E-05	-0.537736	27	2.7	Tacc1	Unknown
DMR16:75470001	16	75470001	75471000	1000	1	5.54E-05	0.6213523	7	0.7	Np4;Defa9	

DMR16:82500001	16	82500001	82501000	1000	1	1.88E-06	-0.8886505	22	2.2		
DMR16:84706001	16	84706001	84707000	1000	1	3.47E-06	1.0515143	4	0.4	Myo16	Cytoskeleton
DMR17:940001	17	940001	941000	1000	1	7.86E-05	-0.9488977	5	0.5	Fancc	
DMR17:5614001	17	5614001	5615000	1000	1	6.56E-05	0.9170118	15	1.5	Agtpbp1	Signaling
DMR17:8200001	17	8200001	8202000	2000	1	9.39E-05	0.9182056	20	1	Trpc7	Transport
DMR17:9856001	17	9856001	9858000	2000	1	1.11E-05	0.8089679	25	1.25	Nsd1	Transcription
DMR17:10789001	17	10789001	10790000	1000	1	2.84E-05	-1.0965923	17	1.7	Cplx2	Unknown
DMR17:13278001	17	13278001	13279000	1000	1	6.69E-06	-0.8792707	22	2.2		
DMR17:13733001	17	13733001	13735000	2000	1	6.60E-05	0.8708097	8	0.4	Shc3	Signaling
DMR17:15883001	17	15883001	15884000	1000	1	3.57E-06	-0.8819787	12	1.2	Ninj1	Cytoskeleton
DMR17:20534001	17	20534001	20535000	1000	1	1.03E-05	-1.1659395	13	1.3		
DMR17:25943001	17	25943001	25944000	1000	1	4.76E-05	1.0016513	6	0.6		
DMR17:27000001	17	27000001	27001000	1000	1	7.78E-05	-1.1445285	21	2.1	Bmp6	Growth Factors & Cytokines
DMR17:29455001	17	29455001	29457000	2000	1	1.09E-05	0.8795631	20	1	Lyrm4	
DMR17:41908001	17	41908001	41909000	1000	1	1.77E-05	0.9468765	4	0.4	Dcdc2	Development
DMR17:42043001	17	42043001	42044000	1000	1	1.24E-05	-1.54302	10	1		
DMR17:44778001	17	44778001	44779000	1000	1	7.60E-05	-0.624403	19	1.9	Hist1h2bo	
DMR17:48344001	17	48344001	48345000	1000	1	1.14E-06	-1.0263377	12	1.2	Amph	Receptor
DMR17:48356001	17	48356001	48357000	1000	1	3.07E-06	-0.7536133	5	0.5	Amph	Receptor
DMR17:53580001	17	53580001	53581000	1000	1	9.17E-06	-1.8076763	3	0.3	Hecw1	Protease
DMR17:56798001	17	56798001	56799000	1000	1	5.80E-05	-0.8462048	8	0.8		
DMR17:57936001	17	57936001	57938000	2000	1	5.76E-05	1.1054366	31	1.55		
DMR17:81553001	17	81553001	81554000	1000	1	7.73E-05	0.8521692	5	0.5	AABR07028691.1	
DMR17:83015001	17	83015001	83017000	2000	1	2.27E-05	0.8166561	12	0.6		
DMR18:811001	18	811001	812000	1000	1	2.45E-05	-0.6955817	8	0.8		
DMR18:7516001	18	7516001	7517000	1000	1	3.32E-05	-0.6179308	11	1.1		
DMR18:16228001	18	16228001	16229000	1000	1	6.89E-05	0.9645348	9	0.9		
DMR18:18693001	18	18693001	18694000	1000	1	9.25E-05	1.0049637	7	0.7		
DMR18:19086001	18	19086001	19088000	2000	1	1.56E-06	0.986444	15	0.75		
DMR18:19089001	18	19089001	19090000	1000	1	4.87E-05	1.0849652	11	1.1		
DMR18:22613001	18	22613001	22614000	1000	1	3.37E-05	1.0027319	10	1		
DMR18:23960001	18	23960001	23961000	1000	1	5.47E-06	0.7052839	10	1	Rit2	Signaling
DMR18:25597001	18	25597001	25598000	1000	1	1.88E-05	1.0622141	4	0.4		
DMR18:26192001	18	26192001	26193000	1000	1	7.11E-05	0.6402148	11	1.1	Nrep	Development
DMR18:30248001	18	30248001	30250000	2000	1	4.73E-06	0.7730744	22	1.1		
DMR18:49378001	18	49378001	49379000	1000	1	8.48E-05	-1.1760157	5	0.5		
DMR18:53572001	18	53572001	53573000	1000	1	7.71E-05	0.7308958	8	0.8		
DMR18:56233001	18	56233001	56234000	1000	1	2.97E-05	-0.925689	15	1.5	Camk2a;AABR07032237.1	Signaling
DMR18:56801001	18	56801001	56802000	1000	1	1.32E-06	0.7420768	17	1.7	U6	
DMR18:72601001	18	72601001	72602000	1000	1	2.46E-05	1.0084949	9	0.9	Smad2	Transcription
DMR18:80181001	18	80181001	80183000	2000	1	1.62E-06	1.005768	27	1.35		
DMR18:80957001	18	80957001	80958000	1000	1	8.66E-05	-1.0242987	12	1.2	Zadh2	Metabolism
DMR18:83644001	18	83644001	83645000	1000	1	3.07E-05	0.7281222	11	1.1		
DMR18:87351001	18	87351001	87353000	2000	1	1.57E-05	0.6939752	6	0.3		
DMR19:1212001	19	1212001	1213000	1000	1	9.63E-05	0.6507686	4	0.4		
DMR19:13210001	19	13210001	13211000	1000	1	8.85E-05	0.9212584	5	0.5		
DMR19:16135001	19	16135001	16136000	1000	1	1.65E-05	-0.6427114	11	1.1		
DMR19:16283001	19	16283001	16284000	1000	1	4.73E-05	0.9655729	7	0.7		
DMR19:17843001	19	17843001	17844000	1000	1	2.62E-05	0.9944065	9	0.9		
DMR19:20252001	19	20252001	20254000	2000	1	6.73E-05	-0.8404615	36	1.8	Zfp423	Transcription
DMR19:26348001	19	26348001	26350000	2000	1	4.54E-05	-0.8120244	53	2.65	AABR07043190.1	
DMR19:31307001	19	31307001	31308000	1000	1	9.06E-05	0.9609904	7	0.7		
DMR19:31412001	19	31412001	31413000	1000	1	2.36E-05	1.0536696	10	1		
DMR19:36684001	19	36684001	36686000	2000	1	3.53E-08	1.2434939	12	0.6		
DMR19:39122001	19	39122001	39123000	1000	1	6.48E-06	0.8161355	11	1.1	Utp4;Sntb2	Development
DMR19:39668001	19	39668001	39669000	1000	1	5.90E-05	-0.9411066	18	1.8	AABR07043776.1	
DMR19:42402001	19	42402001	42403000	1000	1	1.84E-05	0.8794471	10	1		
DMR19:49242001	19	49242001	49243000	1000	1	3.94E-05	1.0272866	9	0.9		
DMR19:53347001	19	53347001	53349000	2000	1	5.00E-05	-0.7062073	60	3		
DMR19:55320001	19	55320001	55323000	3000	1	2.61E-06	-0.9563342	110	3.667	Piezo1	
DMR19:56161001	19	56161001	56163000	2000	1	1.14E-05	-0.9223423	47	2.35	Spire2	Cytoskeleton
DMR19:57988001	19	57988001	57989000	1000	1	2.85E-05	-0.9468674	10	1	Disc1	
DMR20:1212001	20	1212001	1213000	1000	1	2.38E-06	0.7930351	2	0.2	AC094221.3	
DMR20:3451001	20	3451001	3452000	1000	1	6.21E-05	-0.8510616	20	2		
DMR20:4314001	20	4314001	4315000	1000	1	2.98E-05	-0.9002536	18	1.8	C4b;LOC103689965;AABR07044388.1	Immune
DMR20:4460001	20	4460001	4463000	3000	1	5.54E-05	-0.7830771	69	2.3	LOC103689965;AABR07044388.2	
DMR20:4829001	20	4829001	4830000	1000	1	3.58E-05	-0.7401859	19	1.9	RT1-CE1;RT1-CE4;RT1-CE7;Atp6v1g2;Nfk	Immune;Transport;Signaling
DMR20:4949001	20	4949001	4952000	3000	1	5.72E-05	-1.1457808	42	1.4	RT1-CE1;AABR07044408.1;AABR070444	Immune;Protein Binding
DMR20:6512001	20	6512001	6516000	4000	1	1.45E-05	-0.7205251	117	2.925	Ppil1	Immune
DMR20:6680001	20	6680001	6682000	2000	1	1.34E-05	-0.7312552	37	1.85	Ppil1	Immune
DMR20:6940001	20	6940001	6943000	3000	1	7.34E-05	-0.9343952	64	2.133	Pi16;Mtch1;AABR07044473.1	Binding Protein
DMR20:8407001	20	8407001	8408000	1000	1	3.62E-05	-1.379709	19	1.9		
DMR20:9424001	20	9424001	9425000	1000	1	5.48E-05	-0.5320401	32	3.2	Dnah8	Cytoskeleton
DMR20:11539001	20	11539001	11541000	2000	1	4.11E-05	-0.9593214	45	2.25	AABR07044583.2;LOC690386	

DMR20:11920001	20	11920001	11921000	1000	1	3.46E-05	-1.2217402	21	2.1	AABR07044593.1	
DMR20:12001001	20	12001001	12003000	2000	1	9.36E-05	-0.6602865	37	1.85	Adarb1	Epigenetic
DMR20:16464001	20	16464001	16465000	1000	1	6.53E-05	-0.8902909	6	0.6		
DMR20:26529001	20	26529001	26530000	1000	1	6.25E-05	0.7733646	20	2	AABR07044914.1;Tmem167b	Unknown
DMR20:28613001	20	28613001	28615000	2000	1	3.72E-05	0.9111417	17	0.85	Sh3rf3	
DMR20:32009001	20	32009001	32011000	2000	1	3.07E-05	-0.6103511	32	1.6	AABR07045032.1	
DMR20:38960001	20	38960001	38961000	1000	1	6.05E-05	1.1222019	10	1	Hsf2;Serinc1	Signaling;Unknown
DMR20:45992001	20	45992001	45993000	1000	1	3.77E-05	-0.6452822	13	1.3	Fig4	Signaling
DMR20:53947001	20	53947001	53948000	1000	1	2.79E-05	0.7107224	11	1.1	Grik2	Signaling
DMR20:55081001	20	55081001	55084000	3000	2	5.34E-06	1.1526943	18	0.6		
DMR20:55869001	20	55869001	55870000	1000	1	5.62E-06	-0.858182	5	0.5		
DMRX:269001	X	269001	270000	1000	1	6.08E-06	-1.0546504	6	0.6	AABR07036679.1	
DMRX:7403001	X	7403001	7404000	1000	1	9.81E-05	0.6137294	12	1.2		
DMRX:11615001	X	11615001	11616000	1000	1	2.01E-05	-0.9535361	11	1.1		
DMRX:17596001	X	17596001	17597000	1000	1	2.37E-05	0.8264476	7	0.7		
DMRX:18032001	X	18032001	18033000	1000	1	2.70E-05	1.0456964	7	0.7		
DMRX:19300001	X	19300001	19301000	1000	1	8.57E-05	1.0864357	4	0.4		
DMRX:21169001	X	21169001	21170000	1000	1	5.76E-05	-1.0428166	10	1	Phf8	Cell Cycle
DMRX:41586001	X	41586001	41587000	1000	1	6.45E-05	0.9095375	8	0.8		
DMRX:59498001	X	59498001	59499000	1000	1	1.94E-05	1.046875	6	0.6		
DMRX:70501001	X	70501001	70503000	2000	1	5.10E-05	0.6918105	11	0.55	Kif4a	Cytoskeleton
DMRX:79738001	X	79738001	79739000	1000	1	9.27E-05	-1.3639741	2	0.2	LOC103690878	
DMRX:88448001	X	88448001	88449000	1000	1	3.47E-05	0.9603048	3	0.3		
DMRX:110626001	X	110626001	110627000	1000	1	1.01E-05	0.9399365	5	0.5		
DMRX:111704001	X	111704001	111705000	1000	1	7.50E-05	0.8247611	9	0.9		
DMRX:111779001	X	111779001	111780000	1000	1	6.32E-05	-0.6754169	17	1.7		
DMRX:128851001	X	128851001	128852000	1000	1	8.45E-05	0.6218717	14	1.4		
DMRX:130617001	X	130617001	130618000	1000	1	8.31E-05	0.5635119	14	1.4	AABR07041451.1	
DMRX:138218001	X	138218001	138219000	1000	1	7.67E-05	0.9875738	4	0.4	Rap2c	Signaling
DMRX:140697001	X	140697001	140698000	1000	1	8.97E-05	0.7971748	5	0.5		
DMRX:142903001	X	142903001	142904000	1000	1	1.29E-05	1.1333234	7	0.7		
DMRX:156030001	X	156030001	156032000	2000	1	9.78E-05	-1.1445418	16	0.8		
