# Supplementary material for: Epigenome-wide association study for atrazine induced transgenerational DNA methylation and histone retention sperm epigenetic biomarkers for disease
Source: PLoS One. 2020 Dec 16;15(12):e0239380. doi: 10.1371/journal.pone.0239380 (PMC7743986; doi:10.1371/journal.pone.0239380)
Supplement: S5 Table — DMR name, chromosome, start, stop, length, number signature windows, minimum p-value, max log-fold change, CpG number, CpG density, gene annotation, and gene category are presented. (PDF) [file pone.0239380.s012.pdf]

Supplemental Table S5
DMR Site List Puberty p<1e-04

DMR Name	Chr	Start	Stop	Length	# Sig Win	minP	maxLFC	CpG #	CpG Density	Gene Annotation	Gene Category
DMR1:435001	1	435001	436000	1000	1	1.22E-06	-1.1462844	2	0.2		
DMR1:3969001	1	3969001	3970000	1000	1	1.96E-05	-1.4081103	7	0.7	Stxbp5	Receptor
DMR1:16497001	1	16497001	16498000	1000	1	6.62E-05	-1.311799	12	1.2	Ahi1	Development
DMR1:20015001	1	20015001	20016000	1000	1	1.11E-05	0.6393151	15	1.5	L3mbtl3	Epigenetic
DMR1:38352001	1	38352001	38354000	2000	1	8.90E-05	-1.0139721	17	0.85		
DMR1:54960001	1	54960001	54961000	1000	1	5.49E-06	-1.6499381	3	0.3		
DMR1:59901001	1	59901001	59902000	1000	1	8.26E-05	-1.3037831	3	0.3	Fpr-rs3	
DMR1:83303001	1	83303001	83304000	1000	1	5.58E-06	-1.0391266	8	0.8	LOC103691092	
DMR1:93067001	1	93067001	93068000	1000	1	5.58E-07	0.8118998	7	0.7		
DMR1:95528001	1	95528001	95529000	1000	1	2.45E-05	-1.3849444	8	0.8		
DMR1:97801001	1	97801001	97802000	1000	1	8.04E-05	-0.9417029	11	1.1		
DMR1:101794001	1	101794001	101795000	1000	1	3.75E-05	0.7046803	11	1.1	AC095693.1;Cyth2	Signaling
DMR1:131099001	1	131099001	131101000	2000	1	2.54E-05	-0.9085532	10	0.5		
DMR1:154285001	1	154285001	154286000	1000	1	3.06E-05	-1.1617996	6	0.6		
DMR1:156887001	1	156887001	156888000	1000	1	1.25E-05	-1.5061864	6	0.6	Dlg2	
DMR1:167942001	1	167942001	167944000	2000	1	3.70E-05	-1.2117764	6	0.3	Olr57;Olr56	Receptor
DMR1:170903001	1	170903001	170904000	1000	1	8.67E-05	0.8527215	4	0.4	AABR07071959.1	
DMR1:171551001	1	171551001	171553000	2000	1	4.56E-05	-0.637721	11	0.55		
DMR1:179370001	1	179370001	179371000	1000	1	6.27E-06	-0.743028	2	0.2		
DMR1:179563001	1	179563001	179566000	3000	1	2.54E-05	-0.8880772	17	0.567		
DMR1:179858001	1	179858001	179861000	3000	1	1.69E-05	-1.5163121	13	0.433		
DMR1:179944001	1	179944001	179947000	3000	1	4.95E-05	-0.6359109	22	0.733		
DMR1:182993001	1	182993001	182996000	3000	1	5.38E-05	-1.3448014	19	0.633		
DMR1:188236001	1	188236001	188237000	1000	1	3.31E-05	-0.9989495	15	1.5	Tmc7	Transport
DMR1:226007001	1	226007001	226008000	1000	1	5.85E-05	-1.0169825	12	1.2		
DMR1:233973001	1	233973001	233975000	2000	1	1.41E-05	-0.7911151	7	0.35		
DMR1:241988001	1	241988001	241989000	1000	1	3.49E-05	-0.8101507	8	0.8	Tjp2	Extracellular Matrix
DMR1:267472001	1	267472001	267473000	1000	1	2.96E-05	-1.2312384	20	2	Col17a1;AC096311.1	Extracellular Matrix
DMR2:2150001	2	2150001	2151000	1000	1	6.71E-05	-0.8415615	5	0.5		
DMR2:11065001	2	11065001	11066000	1000	1	1.60E-06	-1.4472269	2	0.2		
DMR2:13712001	2	13712001	13713000	1000	1	7.17E-05	-1.0645252	3	0.3		
DMR2:20959001	2	20959001	20960000	1000	1	4.20E-05	-1.191915	27	2.7		
DMR2:27674001	2	27674001	27675000	1000	1	8.63E-06	-1.3415761	9	0.9	AABR07007769.1	
DMR2:27721001	2	27721001	27723000	2000	1	6.24E-05	-1.0213828	10	0.5	AABR07007769.1;Gcnt4;U6	Metabolism
DMR2:28835001	2	28835001	28836000	1000	1	8.14E-05	-1.1397334	2	0.2	AABR07007798.1;AABR07007799.1	
DMR2:40973001	2	40973001	40974000	1000	1	5.95E-05	-1.2280384	10	1	Pde4d	Metabolism
DMR2:52273001	2	52273001	52274000	1000	1	4.89E-06	-1.574294	6	0.6	Nnt	Metabolism
DMR2:54550001	2	54550001	54551000	1000	1	2.46E-05	-0.9728382	5	0.5		
DMR2:62710001	2	62710001	62711000	1000	1	7.73E-05	-0.907719	7	0.7		
DMR2:73641001	2	73641001	73642000	1000	1	5.73E-05	-1.0959463	5	0.5		
DMR2:83834001	2	83834001	83835000	1000	1	9.52E-05	-0.9690998	6	0.6	Ctnnd2	Extracellular Matrix
DMR2:84647001	2	84647001	84648000	1000	1	4.82E-06	-1.263803	11	1.1	Cmbl	Metabolism
DMR2:86253001	2	86253001	86254000	1000	1	4.14E-05	-0.7414549	0	0		
DMR2:90326001	2	90326001	90327000	1000	1	5.40E-05	-1.462393	1	0.1		
DMR2:90662001	2	90662001	90663000	1000	1	3.29E-05	-1.2403823	1	0.1		
DMR2:95004001	2	95004001	95005000	1000	1	5.01E-06	-1.3223055	10	1	Tpd52	
DMR2:145541001	2	145541001	145542000	1000	1	3.03E-05	-0.8369893	8	0.8		
DMR2:159010001	2	159010001	159012000	2000	1	7.36E-05	-1.3925502	28	1.4		
DMR2:160664001	2	160664001	160666000	2000	1	8.58E-05	-1.4516087	6	0.3		
DMR2:168186001	2	168186001	168187000	1000	1	9.40E-05	-0.8282088	3	0.3		
DMR2:173384001	2	173384001	173385000	1000	1	1.27E-05	-1.2252666	2	0.2		
DMR2:180012001	2	180012001	180014000	2000	1	1.49E-05	-1.6920602	18	0.9	Pdgfc	Growth Factors & Cytokines
DMR2:183208001	2	183208001	183210000	2000	1	2.03E-06	0.6277707	26	1.3	Trim2	Unknown
DMR2:196922001	2	196922001	196924000	2000	1	6.98E-05	-1.0493412	18	0.9		
DMR2:200090001	2	200090001	200091000	1000	1	9.83E-06	-0.8603395	13	1.3	Sec22b	Transport
DMR2:204308001	2	204308001	204309000	1000	1	8.98E-05	0.6087219	11	1.1	Slc22a15	Transport
DMR2:218333001	2	218333001	218334000	1000	1	6.26E-05	-0.7239514	5	0.5		
DMR2:246377001	2	246377001	246378000	1000	1	6.99E-05	0.7324466	8	0.8		
DMR2:249271001	2	249271001	249272000	1000	1	4.22E-05	-1.2474919	3	0.3		
DMR2:257414001	2	257414001	257415000	1000	1	4.83E-05	-0.9343216	8	0.8	Dnajb4	Protein Binding
DMR3:683001	3	683001	684000	1000	1	4.71E-05	-0.9930672	1	0.1		
DMR3:1867001	3	1867001	1868000	1000	1	9.46E-05	0.7571476	18	1.8	Cacna1b	Transport
DMR3:11018001	3	11018001	11019000	1000	1	2.68E-05	-0.8953186	8	0.8	Nup214	Transcription
DMR3:23620001	3	23620001	23621000	1000	1	7.19E-05	-0.9441565	3	0.3		
DMR3:25190001	3	25190001	25192000	2000	1	2.86E-05	0.779564	21	1.05	AABR07051878.1	
DMR3:34960001	3	34960001	34961000	1000	1	9.73E-05	-0.7298448	3	0.3		

DMR3:36908001	3	36908001	36911000	3000	1	6.81E-05	0.9319519	42	1.4		
DMR3:38719001	3	38719001	38720000	1000	1	2.42E-05	-1.4041677	4	0.4	Prpf40a	
DMR3:42407001	3	42407001	42408000	1000	1	2.56E-05	-1.3263675	2	0.2		
DMR3:52508001	3	52508001	52509000	1000	1	8.15E-05	-1.0031067	13	1.3	Scn1a	Metabolism
DMR3:57938001	3	57938001	57940000	2000	1	2.17E-05	0.7032637	25	1.25	Slc25a12	Binding Protein
DMR3:59609001	3	59609001	59611000	2000	1	3.04E-05	-1.5731187	11	0.55		
DMR3:64721001	3	64721001	64722000	1000	1	9.52E-05	0.5688342	7	0.7	Cwc22	Translation
DMR3:70548001	3	70548001	70549000	1000	1	4.64E-05	0.9046255	6	0.6		
DMR3:80549001	3	80549001	80550000	1000	1	3.92E-05	0.5960607	15	1.5	F2;Arhgap1	Protease;Signaling
DMR3:84108001	3	84108001	84109000	1000	1	9.21E-05	-0.7199395	1	0.1		
DMR3:89575001	3	89575001	89576000	1000	1	3.71E-05	-1.287247	6	0.6		
DMR3:90047001	3	90047001	90048000	1000	1	1.67E-05	-1.5739578	13	1.3		
DMR3:93887001	3	93887001	93888000	1000	1	5.42E-05	-1.4005	6	0.6		
DMR3:95764001	3	95764001	95765000	1000	1	1.52E-05	-1.0294168	10	1		
DMR3:137662001	3	137662001	137663000	1000	1	1.93E-05	-1.3225906	9	0.9	Pcsk2	Proteolysis
DMR3:139408001	3	139408001	139409000	1000	1	4.75E-06	-1.1035431	6	0.6		
DMR3:146479001	3	146479001	146480000	1000	1	4.89E-05	0.6151724	11	1.1	Acss1;Vsx1	Metabolism
DMR3:149268001	3	149268001	149269000	1000	1	3.48E-05	-0.8941647	13	1.3	AABR07054352.3;AABR07054352.1	
DMR3:149482001	3	149482001	149483000	1000	1	6.44E-05	0.7572435	9	0.9	Bpifa6	
DMR3:150350001	3	150350001	150351000	1000	1	8.10E-05	-0.7541286	9	0.9	Raly	Transcription
DMR3:152264001	3	152264001	152265000	1000	1	3.39E-05	-0.801264	12	1.2	Rbm39	Translation
DMR3:162832001	3	162832001	162833000	1000	1	2.31E-05	-0.8244745	10	1	Sulf2	Metabolism
DMR3:177563001	3	177563001	177564000	1000	1	1.16E-05	0.699017	6	0.6		
DMR4:743001	4	743001	744000	1000	1	9.09E-05	-1.166318	14	1.4	AABR07059002.1	
DMR4:20764001	4	20764001	20765000	1000	1	7.96E-06	-1.2341364	1	0.1		
DMR4:36538001	4	36538001	36539000	1000	1	9.84E-05	-0.9737943	3	0.3		
DMR4:36764001	4	36764001	36765000	1000	1	4.08E-05	-1.0502416	0	0	AABR07059807.1	
DMR4:65746001	4	65746001	65747000	1000	1	3.72E-05	-0.7270659	13	1.3	Atp6v0a4	Metabolism
DMR4:71020001	4	71020001	71021000	1000	1	6.72E-05	-0.8044122	2	0.2		
DMR4:72887001	4	72887001	72888000	1000	1	7.34E-05	0.7733921	9	0.9	Tpk1	Signaling
DMR4:81780001	4	81780001	81781000	1000	1	2.75E-05	0.959244	12	1.2	AC096072.1	
DMR4:106707001	4	106707001	106708000	1000	1	8.39E-05	-0.9152563	10	1		
DMR4:116516001	4	116516001	116517000	1000	1	3.13E-05	-1.0181712	2	0.2	Exoc6b	Transport
DMR4:118482001	4	118482001	118483000	1000	1	7.94E-06	-0.9588982	11	1.1	Mxd1;Gmcl1	Transcription
DMR4:118680001	4	118680001	118682000	2000	1	5.87E-05	-2.675921	26	1.3	Aak1	Signaling
DMR4:124451001	4	124451001	124452000	1000	1	4.13E-05	-1.1760666	1	0.1		
DMR4:132212001	4	132212001	132213000	1000	1	8.54E-06	0.4910494	24	2.4		
DMR4:133418001	4	133418001	133420000	2000	1	1.56E-05	0.5675649	30	1.5		
DMR4:135153001	4	135153001	135154000	1000	1	6.57E-05	-1.0340491	7	0.7		
DMR4:138689001	4	138689001	138691000	2000	1	1.58E-05	0.8439342	20	1	Cntn4	Cytoskeleton
DMR4:161335001	4	161335001	161336000	1000	1	5.79E-06	0.880879	15	1.5	AABR07062111.1	
DMR5:2231001	5	2231001	2232000	1000	1	5.80E-05	0.7127916	7	0.7		
DMR5:18587001	5	18587001	18588000	1000	1	5.85E-06	-1.3747396	3	0.3		
DMR5:31543001	5	31543001	31544000	1000	1	1.46E-06	-0.751427	6	0.6		
DMR5:35411001	5	35411001	35412000	1000	1	7.67E-06	-1.0519778	0	0		
DMR5:39851001	5	39851001	39852000	1000	1	4.74E-05	-1.1123388	5	0.5		
DMR5:40721001	5	40721001	40722000	1000	1	1.29E-06	-1.647365	7	0.7		
DMR5:60413001	5	60413001	60415000	2000	2	4.71E-05	0.7815211	21	1.05	Zcchc7	Transcription
DMR5:62472001	5	62472001	62474000	2000	1	3.23E-05	0.6173124	28	1.4	Gabbr2	Receptor
DMR5:63220001	5	63220001	63221000	1000	1	5.59E-05	-1.6685847	14	1.4		
DMR5:70565001	5	70565001	70566000	1000	1	4.80E-05	-0.9826168	11	1.1	Fktn	
DMR5:84797001	5	84797001	84798000	1000	1	6.79E-05	0.5181533	6	0.6		
DMR5:90237001	5	90237001	90238000	1000	1	7.58E-05	-1.200461	7	0.7	Frmd3	Signaling
DMR5:121406001	5	121406001	121407000	1000	1	8.85E-05	-0.5668437	11	1.1		
DMR5:123603001	5	123603001	123604000	1000	1	1.54E-05	-1.0229661	10	1		
DMR5:128329001	5	128329001	128330000	1000	1	5.41E-05	0.7160282	11	1.1	Zfyve9	Transcription
DMR5:132128001	5	132128001	132129000	1000	1	2.07E-05	-1.1164891	1	0.1		
DMR5:136513001	5	136513001	136515000	2000	1	3.51E-05	-1.0517683	20	1	Eri3	Transcription
DMR5:141139001	5	141139001	141140000	1000	1	5.18E-05	-1.0720199	5	0.5	Macf1	Cytoskeleton
DMR5:153972001	5	153972001	153973000	1000	1	6.88E-05	-1.0997845	13	1.3	LOC500567	
DMR5:154776001	5	154776001	154777000	1000	1	9.79E-06	0.5123249	12	1.2		
DMR6:3090001	6	3090001	3091000	1000	1	3.08E-05	-1.0140612	13	1.3	Arhgef33	
DMR6:4945001	6	4945001	4947000	2000	1	2.84E-05	-1.1383617	15	0.75		
DMR6:22804001	6	22804001	22806000	2000	1	9.18E-05	-0.7461745	26	1.3	Alk	Receptor
DMR6:28609001	6	28609001	28610000	1000	1	7.33E-05	-0.9294956	12	1.2	Adcy3	Metabolism
DMR6:29025001	6	29025001	29027000	2000	1	7.10E-05	-1.4045185	11	0.55	Atad2b	Metabolism
DMR6:50313001	6	50313001	50315000	2000	1	6.80E-05	-0.6557676	30	1.5	AABR07063811.1	
DMR6:50911001	6	50911001	50912000	1000	1	7.72E-05	-1.3215046	6	0.6	Bcap29	Transport
DMR6:63899001	6	63899001	63901000	2000	1	7.35E-05	0.7210983	19	0.95		
DMR6:70249001	6	70249001	70250000	1000	1	8.07E-05	-0.8806379	2	0.2		
DMR6:87210001	6	87210001	87211000	1000	1	3.71E-05	0.5185988	3	0.3	AABR07064618.1	

DMR6:104888001	6	104888001	104890000	2000	1	1.14E-05	-1.0143492	17	0.85	Slc8a3	Transport
DMR6:109572001	6	109572001	109573000	1000	1	7.18E-05	0.6652949	14	1.4	Batf	Transcription
DMR6:115123001	6	115123001	115124000	1000	1	1.04E-05	-0.6885389	4	0.4	Cep128	
DMR6:115456001	6	115456001	115459000	3000	1	5.76E-05	-1.3115196	37	1.233	Ston2	Metabolism
DMR6:116771001	6	116771001	116772000	1000	1	1.45E-05	0.5440843	9	0.9		
DMR6:127978001	6	127978001	127979000	1000	1	2.44E-05	-1.6253348	1	0.1	LOC500712	
DMR6:128143001	6	128143001	128144000	1000	1	8.00E-05	0.512357	27	2.7	Gsc;AABR07065424.1	Transcription
DMR7:12544001	7	12544001	12545000	1000	1	7.98E-06	1.0207165	14	1.4	Polr2e;Arhgap45;Med16	Transcription
DMR7:21517001	7	21517001	21520000	3000	1	7.79E-05	-0.9696776	15	0.5		
DMR7:33173001	7	33173001	33174000	1000	1	5.55E-06	-1.1749653	4	0.4		
DMR7:34902001	7	34902001	34903000	1000	1	4.42E-05	0.7257171	8	0.8	Vezt	
DMR7:44513001	7	44513001	44515000	2000	1	1.57E-05	0.8801237	18	0.9		
DMR7:46967001	7	46967001	46968000	1000	1	1.86E-05	-1.573756	7	0.7		
DMR7:56904001	7	56904001	56905000	1000	1	2.40E-05	-1.2369234	3	0.3		
DMR7:56990001	7	56990001	56991000	1000	1	1.37E-05	-1.0578927	7	0.7		
DMR7:65117001	7	65117001	65118000	1000	1	3.82E-05	-0.7920895	22	2.2		
DMR7:82810001	7	82810001	82811000	1000	1	7.49E-05	-1.7810449	2	0.2		
DMR7:95492001	7	95492001	95493000	1000	1	2.22E-05	-1.2124497	9	0.9	Sntb1	Development
DMR7:100336001	7	100336001	100338000	2000	1	3.35E-06	-1.3222847	10	0.5	AABR07058102.1	
DMR7:117745001	7	117745001	117747000	2000	1	3.10E-05	0.5010036	23	1.15	Ppp1r16a	Signaling
DMR7:133483001	7	133483001	133484000	1000	1	5.19E-05	0.6093221	7	0.7	Cntn1	Extracellular Matrix
DMR7:133724001	7	133724001	133726000	2000	1	7.85E-05	-0.925269	29	1.45		
DMR7:133741001	7	133741001	133742000	1000	1	6.06E-05	0.5743464	29	2.9		
DMR7:133818001	7	133818001	133819000	1000	1	7.00E-06	0.7345987	8	0.8		
DMR7:134405001	7	134405001	134406000	1000	1	4.26E-05	-1.1342276	7	0.7		
DMR7:135258001	7	135258001	135259000	1000	1	5.78E-05	0.6671087	13	1.3		
DMR7:141462001	7	141462001	141464000	2000	1	4.85E-06	0.5455552	16	0.8	Lima1	Cytoskeleton
DMR7:141628001	7	141628001	141629000	1000	1	1.60E-05	0.6163305	15	1.5	AABR07058884.1	
DMR7:144417001	7	144417001	144418000	1000	1	4.83E-05	0.7143011	7	0.7		
DMR8:11332001	8	11332001	11334000	2000	1	2.09E-05	0.7020515	32	1.6		
DMR8:13074001	8	13074001	13076000	2000	1	7.03E-05	-1.2441486	16	0.8	Amotl1	Unknown
DMR8:15718001	8	15718001	15719000	1000	1	4.21E-05	-0.9896102	6	0.6		
DMR8:19837001	8	19837001	19838000	1000	1	9.54E-05	-1.2619918	2	0.2		
DMR8:25016001	8	25016001	25017000	1000	1	1.88E-05	-0.9205614	7	0.7		
DMR8:32793001	8	32793001	32794000	1000	1	8.12E-05	-1.0666987	17	1.7		
DMR8:45951001	8	45951001	45952000	1000	1	9.44E-05	-1.1152386	8	0.8		
DMR8:58421001	8	58421001	58423000	2000	1	9.96E-06	-1.0515845	39	1.95	Sln	
DMR8:72300001	8	72300001	72301000	1000	1	3.91E-05	0.6055227	9	0.9	AABR07070486.1	
DMR8:73745001	8	73745001	73746000	1000	1	1.12E-05	-1.3016169	16	1.6	Vps13c	
DMR8:74124001	8	74124001	74125000	1000	1	1.30E-05	-1.3553095	5	0.5		
DMR8:74891001	8	74891001	74893000	2000	1	3.76E-05	-0.795244	22	1.1		
DMR8:77341001	8	77341001	77343000	2000	1	8.10E-05	0.5290338	31	1.55	Lipc	
DMR8:87872001	8	87872001	87873000	1000	1	4.67E-05	-0.9362102	3	0.3	Impg1	Receptor
DMR8:94130001	8	94130001	94131000	1000	1	8.57E-05	-1.068719	9	0.9	Ube3d;Dop1a	Proteolysis
DMR8:96921001	8	96921001	96922000	1000	1	6.89E-05	-1.2615387	1	0.1		
DMR8:106587001	8	106587001	106588000	1000	1	8.82E-05	0.6621953	11	1.1	AABR07071243.1;AABR07071244.1	
DMR8:108727001	8	108727001	108728000	1000	1	8.04E-05	-1.2565335	12	1.2		
DMR8:113445001	8	113445001	113447000	2000	1	8.53E-05	-0.8899048	13	0.65	Cpne4	Development
DMR8:120116001	8	120116001	120117000	1000	1	6.67E-05	-1.112227	12	1.2		
DMR9:4170001	9	4170001	4171000	1000	1	5.19E-05	-0.8910663	4	0.4	RGD1562392;Sult1c2a	Metabolism
DMR9:8965001	9	8965001	8966000	1000	1	5.82E-05	-0.6505026	6	0.6		
DMR9:12877001	9	12877001	12880000	3000	1	1.70E-05	0.5253982	61	2.033	Rftn1	Unknown
DMR9:31860001	9	31860001	31862000	2000	1	7.39E-05	0.6392246	21	1.05	Adgrb3	
DMR9:32388001	9	32388001	32389000	1000	1	6.50E-05	-1.1477828	6	0.6		
DMR9:38148001	9	38148001	38149000	1000	1	3.61E-05	-0.7255721	21	2.1	Dst	Cell Junction
DMR9:44298001	9	44298001	44300000	2000	1	8.93E-05	-0.5958476	26	1.3		
DMR9:53805001	9	53805001	53806000	1000	1	2.34E-05	-1.1078608	1	0.1		
DMR9:56395001	9	56395001	56396000	1000	1	3.65E-05	-1.2424213	6	0.6		
DMR9:60646001	9	60646001	60647000	1000	1	4.32E-05	0.528162	15	1.5	Hecw2	Proteolysis
DMR9:65252001	9	65252001	65254000	2000	1	4.20E-05	0.5875978	27	1.35	Aox2	
DMR10:6876001	10	6876001	6878000	2000	1	1.77E-05	0.6133358	19	0.95	RGD1309748	
DMR10:13788001	10	13788001	13789000	1000	1	3.17E-07	-1.4022943	8	0.8	Abca3;AC103090.1;Rnps1;Eci1	Receptor;Translation
DMR10:37226001	10	37226001	37228000	2000	1	9.01E-05	-1.1520515	7	0.35	Sar1b	Development
DMR10:45720001	10	45720001	45721000	1000	1	3.85E-06	-1.344867	13	1.3	Prss38	Protease
DMR10:48171001	10	48171001	48172000	1000	1	8.64E-05	0.679859	7	0.7	Akap10;U6	Signaling
DMR10:58424001	10	58424001	58425000	1000	1	1.07E-05	-1.1299848	7	0.7	Gm22927	
DMR10:100293001	10	100293001	100294000	1000	1	2.55E-06	-1.6087288	12	1.2		
DMR10:101809001	10	101809001	101810000	1000	1	1.51E-05	0.9224978	11	1.1	Slc39a11	Metabolism
DMR10:102123001	10	102123001	102124000	1000	1	3.08E-05	-1.1371111	7	0.7		
DMR10:104413001	10	104413001	104414000	1000	1	9.36E-05	-0.8676525	22	2.2	Llgl2	Development
DMR10:109925001	10	109925001	109926000	1000	1	3.67E-05	-0.8455131	16	1.6		

DMR11:12502001	11	12502001	12504000	2000	1	6.79E-05	-1.2140538	14	0.7		
DMR11:12571001	11	12571001	12572000	1000	1	2.21E-05	0.5790611	13	1.3		
DMR11:27521001	11	27521001	27522000	1000	1	3.06E-06	-1.0946268	16	1.6		
DMR11:35622001	11	35622001	35624000	2000	1	9.95E-05	-1.2553765	16	0.8	AABR07033697.2	
DMR11:54735001	11	54735001	54736000	1000	1	1.46E-05	-0.8983856	0	0		
DMR11:75224001	11	75224001	75225000	1000	1	1.41E-05	-1.4224092	11	1.1		
DMR11:78732001	11	78732001	78733000	1000	1	1.54E-05	0.8447141	8	0.8	Tprg1	Unknown
DMR12:1491001	12	1491001	1492000	1000	1	7.05E-06	-1.2949655	4	0.4		
DMR12:1722001	12	1722001	1723000	1000	1	7.22E-05	-1.2299221	13	1.3	Insr	Receptor
DMR12:2044001	12	2044001	2045000	1000	1	4.37E-05	-0.9981239	17	1.7	Zfp358;Mcoln1	Transcription;Cytoskeleton
DMR12:2116001	12	2116001	2118000	2000	1	1.41E-05	-1.8305003	14	0.7		
DMR12:15557001	12	15557001	15558000	1000	1	2.79E-05	-0.7825887	6	0.6		
DMR12:17514001	12	17514001	17515000	1000	1	2.47E-05	0.8024589	14	1.4	Sun1	Cytoskeleton
DMR12:17996001	12	17996001	17997000	1000	1	7.28E-05	0.6559075	8	0.8		
DMR12:27286001	12	27286001	27287000	1000	1	9.93E-05	0.5380009	21	2.1	AABR07035916.1	
DMR12:36106001	12	36106001	36107000	1000	1	6.89E-05	-1.2933727	19	1.9	Tmem132b	Unknown
DMR12:36556001	12	36556001	36558000	2000	1	5.88E-05	-1.1285836	19	0.95	Aacs	Metabolism
DMR13:19631001	13	19631001	19634000	3000	1	9.22E-05	-0.5619679	22	0.733		
DMR13:24469001	13	24469001	24470000	1000	1	1.22E-05	-1.4649854	4	0.4		
DMR13:36630001	13	36630001	36631000	1000	1	5.13E-07	1.0480467	16	1.6		
DMR13:47241001	13	47241001	47242000	1000	1	8.36E-05	0.5429536	10	1		
DMR13:49003001	13	49003001	49004000	1000	1	3.91E-05	-0.7090897	17	1.7	AABR07020966.1	
DMR13:65436001	13	65436001	65437000	1000	1	5.07E-05	-1.0516502	7	0.7		
DMR13:73361001	13	73361001	73362000	1000	1	5.36E-07	-0.9666148	10	1	Lhx4	Transcription
DMR13:95887001	13	95887001	95888000	1000	1	6.90E-05	0.7151393	10	1	LOC689766	
DMR13:101498001	13	101498001	101499000	1000	1	6.46E-05	0.5295476	11	1.1	Disp1	
DMR13:105768001	13	105768001	105769000	1000	1	4.13E-05	0.8314303	9	0.9	Gpatch2	Transcription
DMR13:107704001	13	107704001	107705000	1000	1	8.03E-06	-1.4825674	3	0.3	Kcnk2	Transport
DMR13:112927001	13	112927001	112928000	1000	1	2.69E-06	-1.2514641	3	0.3		
DMR13:113490001	13	113490001	113491000	1000	1	8.75E-05	-1.1027395	6	0.6	Plxna2	Receptor
DMR14:7716001	14	7716001	7717000	1000	1	8.01E-05	0.6563868	18	1.8	Ptpn13	Signaling
DMR14:18858001	14	18858001	18859000	1000	1	6.19E-05	-1.1809564	8	0.8	Pf4;Ppbp;AABR07014512.2;Cxcl6	Growth Factors & Cytokines
DMR14:23349001	14	23349001	23350000	1000	1	7.74E-06	0.9923343	6	0.6	Tmprss11d	Protease
DMR14:38106001	14	38106001	38107000	1000	1	5.39E-07	-1.2753713	13	1.3	Nipal1	
DMR14:40523001	14	40523001	40524000	1000	1	4.51E-05	-1.2440247	4	0.4		
DMR14:71596001	14	71596001	71597000	1000	1	6.51E-05	0.5200386	5	0.5	Prom1	Transport
DMR14:72078001	14	72078001	72079000	1000	1	6.00E-05	-0.8096496	5	0.5	C1qtnf7	Growth Factors & Cytokines
DMR14:89868001	14	89868001	89869000	1000	1	3.27E-06	0.6669236	5	0.5		
DMR14:91229001	14	91229001	91230000	1000	1	5.77E-05	-0.9902468	1	0.1	Vwc2;5S_rRNA	
DMR14:95532001	14	95532001	95535000	3000	1	1.29E-06	-1.6023243	25	0.833		
DMR14:100586001	14	100586001	100587000	1000	1	2.25E-05	0.6279084	3	0.3		
DMR14:105999001	14	105999001	1.06E+08	1000	1	9.37E-05	0.6221604	20	2	Peli1	Signaling
DMR14:107703001	14	107703001	107704000	1000	1	4.67E-05	-1.0500256	8	0.8	Commd1	
DMR14:109245001	14	109245001	109246000	1000	1	6.04E-05	-0.8331377	22	2.2		
DMR15:5822001	15	5822001	5823000	1000	1	1.00E-05	-1.0787395	7	0.7	Cd99l2	Extracellular Matrix
DMR15:9819001	15	9819001	9820000	1000	1	5.70E-05	-1.6271292	5	0.5		
DMR15:15760001	15	15760001	15761000	1000	1	3.28E-06	0.5800377	19	1.9		
DMR15:16225001	15	16225001	16226000	1000	1	6.74E-05	1.0944276	7	0.7		
DMR15:20426001	15	20426001	20428000	2000	1	6.08E-05	-1.4356213	18	0.9	AABR07017269.1	
DMR15:21184001	15	21184001	21186000	2000	1	5.88E-05	-1.2399142	12	0.6	AABR07017308.1	
DMR15:23130001	15	23130001	23133000	3000	1	1.01E-05	-1.7395231	21	0.7		
DMR15:23192001	15	23192001	23194000	2000	1	7.59E-05	-0.889956	7	0.35		
DMR15:26001001	15	26001001	26002000	1000	1	2.89E-05	-0.9787275	5	0.5		
DMR15:26038001	15	26038001	26039000	1000	1	1.44E-05	-1.1297024	2	0.2	Naa30	Metabolism
DMR15:47176001	15	47176001	47177000	1000	1	5.57E-05	-1.1627238	5	0.5	Xkr6	Immune
DMR15:49138001	15	49138001	49139000	1000	1	5.36E-05	-0.7923326	9	0.9	Scara5	Unknown
DMR15:65236001	15	65236001	65237000	1000	1	3.11E-05	-1.1734616	3	0.3		
DMR15:86176001	15	86176001	86177000	1000	1	3.89E-05	0.8810072	20	2	Uchl3	Protease
DMR15:90951001	15	90951001	90952000	1000	1	5.46E-05	0.6097344	7	0.7	Mycbp2	Metabolism
DMR15:103748001	15	103748001	103749000	1000	1	9.82E-05	0.5432777	24	2.4	Abcc4	Receptor
DMR15:108660001	15	108660001	108664000	4000	1	4.00E-06	1.8507672	88	2.2	Clybl	Metabolism
DMR15:108671001	15	108671001	108672000	1000	1	4.57E-05	1.0080987	7	0.7	Clybl	Metabolism
DMR15:108692001	15	108692001	108693000	1000	1	1.29E-05	1.6671952	21	2.1	Clybl	Metabolism
DMR16:9431001	16	9431001	9432000	1000	1	2.39E-05	-1.1940834	13	1.3	Wdfy4	
DMR16:15106001	16	15106001	15108000	2000	1	1.41E-05	-0.7667964	27	1.35	LOC683469	Translation
DMR16:39058001	16	39058001	39059000	1000	1	5.81E-05	-1.0308247	8	0.8		
DMR16:39324001	16	39324001	39326000	2000	1	9.92E-06	-1.4432043	9	0.45		
DMR16:42242001	16	42242001	42243000	1000	1	5.63E-05	-1.5901394	3	0.3		
DMR16:64485001	16	64485001	64486000	1000	1	2.79E-05	-1.1520814	4	0.4		
DMR16:85539001	16	85539001	85540000	1000	1	5.84E-05	0.4469145	5	0.5		
DMR16:86646001	16	86646001	86647000	1000	1	1.03E-05	0.5504418	22	2.2	Efnb2	Signaling

DMR16:88003001	16	88003001	88004000	1000	1	4.62E-06	0.6981062	8	0.8		
DMR16:88127001	16	88127001	88128000	1000	1	5.68E-05	-0.8139281	8	0.8		
DMR17:16041001	17	16041001	16044000	3000	1	7.58E-05	-0.8525884	36	1.2	Wnk2	Signaling
DMR17:16594001	17	16594001	16595000	1000	1	3.35E-06	-1.660094	9	0.9		
DMR17:17747001	17	17747001	17748000	1000	1	2.10E-05	0.6225104	27	2.7		
DMR17:53399001	17	53399001	53400000	1000	1	3.33E-06	-1.2824609	1	0.1	Hecw1;AABR07028027.1	Protease
DMR17:55798001	17	55798001	55799000	1000	1	2.28E-06	-1.6014162	7	0.7		
DMR17:73795001	17	73795001	73796000	1000	1	8.98E-05	-1.112587	6	0.6		
DMR17:76868001	17	76868001	76869000	1000	1	3.44E-05	-1.1220655	11	1.1	Camk1d	Signaling
DMR17:80822001	17	80822001	80823000	1000	1	2.55E-05	-0.7066377	15	1.5	Trdmt1	Transcription
DMR17:89385001	17	89385001	89388000	3000	1	2.48E-05	-0.7755132	23	0.767		
DMR17:89389001	17	89389001	89397000	8000	2	1.18E-06	-0.6710242	64	0.8		
DMR17:89430001	17	89430001	89445000	15000	3	1.12E-06	-0.7520237	96	0.64	AABR07028872.1	
DMR17:89446001	17	89446001	89464000	18000	4	6.00E-08	-0.7730347	115	0.639	AABR07028872.1	
DMR17:89466001	17	89466001	89469000	3000	1	4.37E-07	-0.7250559	23	0.767		
DMR18:2976001	18	2976001	2977000	1000	1	4.69E-05	-1.3215282	12	1.2	AABR07031168.1	
DMR18:13711001	18	13711001	13712000	1000	1	1.54E-05	-1.4480359	14	1.4	Nol4	Translation
DMR18:16554001	18	16554001	16555000	1000	1	2.22E-06	-1.0302868	16	1.6	Elp2	Transcription
DMR18:18453001	18	18453001	18454000	1000	1	4.22E-05	-1.2578587	12	1.2		
DMR18:23034001	18	23034001	23035000	1000	1	2.08E-05	-1.3733509	17	1.7	Pik3c3	Signaling
DMR18:27555001	18	27555001	27557000	2000	1	1.93E-05	-0.9568318	18	0.9	Cdc25c;U6;LOC100363294;Fam53c	Cell Cycle
DMR18:30279001	18	30279001	30280000	1000	1	1.10E-05	-1.4962182	4	0.4	Pcdhb1	Extracellular Matrix
DMR18:53598001	18	53598001	53599000	1000	1	2.24E-05	-0.8816391	1	0.1		
DMR18:54496001	18	54496001	54497000	1000	1	6.94E-05	-0.9411587	12	1.2	Chsy3	Metabolism
DMR18:56159001	18	56159001	56160000	1000	1	6.83E-05	-0.9631697	12	1.2		
DMR18:76192001	18	76192001	76193000	1000	1	5.54E-05	-1.0510845	12	1.2		
DMR18:77197001	18	77197001	77198000	1000	1	4.41E-05	0.4908132	21	2.1	Nfatc1	Transcription
DMR18:79886001	18	79886001	79887000	1000	1	5.21E-05	-0.9121166	24	2.4		
DMR18:83884001	18	83884001	83885000	1000	1	1.43E-05	-1.3414639	2	0.2		
DMR18:85028001	18	85028001	85029000	1000	1	9.20E-07	-1.4918199	19	1.9	AABR07032834.1	
DMR18:88104001	18	88104001	88106000	2000	1	1.04E-05	-1.1212648	33	1.65		
DMR19:22099001	19	22099001	22100000	1000	1	2.20E-05	-1.3313688	12	1.2	Phkb	Signaling
DMR19:57534001	19	57534001	57535000	1000	1	2.13E-05	-1.1145928	10	1		
DMR20:23705001	20	23705001	23706000	1000	1	4.20E-06	-1.4501808	9	0.9		
DMR20:30875001	20	30875001	30876000	1000	1	1.61E-05	-1.2259581	16	1.6	Adamts14	Protease
DMR20:42092001	20	42092001	42093000	1000	1	8.96E-05	-1.0100606	3	0.3		
DMR20:48860001	20	48860001	48861000	1000	1	2.30E-06	0.6891799	29	2.9	Qrsl1	Translation
DMRX:27789001	X	27789001	27790000	1000	1	2.59E-05	-1.4122122	3	0.3		
DMRX:32396001	X	32396001	32397000	1000	1	7.36E-05	0.9238835	17	1.7		
DMRX:49554001	X	49554001	49555000	1000	1	9.85E-05	-0.6735922	28	2.8		
DMRX:101420001	X	101420001	101421000	1000	1	2.02E-05	-1.1107346	8	0.8		
DMRX:133088001	X	133088001	133089000	1000	1	5.11E-05	1.3419176	4	0.4		
DMRX:151209001	X	151209001	151210000	1000	1	2.71E-05	-1.1625839	2	0.2		
