# Supplementary material for: Epigenome-wide association study for atrazine induced transgenerational DNA methylation and histone retention sperm epigenetic biomarkers for disease
Source: PLoS One. 2020 Dec 16;15(12):e0239380. doi: 10.1371/journal.pone.0239380 (PMC7743986; doi:10.1371/journal.pone.0239380)
Supplement: S6 Table — DMR name, chromosome, start, stop, length, number signature windows, minimum p-value, max log-fold change, CpG number, CpG density, gene annotation, and gene category are presented. (PDF) [file pone.0239380.s013.pdf]

Supplemental Table S6
DMR Site List Multiple Disease p<1e-04

DMR Name	Chr	Start	Stop	Length	# Sig Win	minP	maxLFC	CpG #	CpG Density	Gene Annotation	Gene Category
DMR1:3688001	1	3688001	3689000	1000	1	9.79E-05	0.4550467	12	1.2		
DMR1:8648001	1	8648001	8649000	1000	1	1.58E-05	0.4124684	10	1	Adgrg6	
DMR1:8743001	1	8743001	8744000	1000	1	7.87E-08	0.6323743	8	0.8	Adgrg6	
DMR1:22650001	1	22650001	22652000	2000	1	6.39E-05	-0.5591665	19	0.95	Vnn3	Metabolism
DMR1:28333001	1	28333001	28335000	2000	1	8.51E-06	0.4880196	16	0.8	Rnf217	
DMR1:29063001	1	29063001	29065000	2000	1	9.84E-05	-0.4192423	25	1.25		
DMR1:38352001	1	38352001	38355000	3000	1	3.12E-06	-0.7536336	18	0.6		
DMR1:46193001	1	46193001	46194000	1000	1	9.02E-06	-0.7735747	11	1.1	Arid1b	Transcription
DMR1:48679001	1	48679001	48680000	1000	1	9.45E-05	-0.761413	10	1	Map3k4;AABR07001516.1	Signaling
DMR1:56910001	1	56910001	56911000	1000	1	9.83E-05	0.4104039	7	0.7	Wdr27	
DMR1:74044001	1	74044001	74045000	1000	1	5.51E-05	-0.6455263	4	0.4		
DMR1:76385001	1	76385001	76386000	1000	1	5.78E-05	-0.7321682	2	0.2	Sult2a6	Metabolism
DMR1:83672001	1	83672001	83673000	1000	1	6.42E-06	-0.6435801	4	0.4	Cyp2a1	
DMR1:85106001	1	85106001	85107000	1000	1	5.52E-05	-0.660996	5	0.5	Fcgbpl1;Fbl;Dyrk1b	Transcription;Signaling
DMR1:105624001	1	105624001	105625000	1000	1	5.88E-06	0.6081659	13	1.3	Nell1	Development
DMR1:112151001	1	112151001	112152000	1000	1	7.52E-05	-0.6567794	1	0.1	Luzp2;Gabrg3	Receptor
DMR1:115213001	1	115213001	115214000	1000	1	4.15E-05	-0.5405133	10	1		
DMR1:137431001	1	137431001	137432000	1000	1	6.54E-05	0.5092987	7	0.7		
DMR1:139862001	1	139862001	139863000	1000	1	6.11E-05	-0.6440615	6	0.6		
DMR1:142568001	1	142568001	142569000	1000	1	1.67E-05	-0.5883048	7	0.7	Iqgap1	Signaling
DMR1:151794001	1	151794001	151795000	1000	1	5.68E-08	-0.6204871	4	0.4		
DMR1:165426001	1	165426001	165428000	2000	1	2.11E-05	-0.8288968	21	1.05	C2cd3	
DMR1:167821001	1	167821001	167822000	1000	1	1.75E-05	-0.8308516	3	0.3	LOC684171;LOC684179	Receptor
DMR1:171298001	1	171298001	171300000	2000	1	9.18E-05	0.6303828	16	0.8	Nlrp14	
DMR1:178315001	1	178315001	178316000	1000	1	3.67E-05	-0.5339629	10	1		
DMR1:190875001	1	190875001	190876000	1000	1	7.23E-05	-0.546436	19	1.9	Polr3e	Transcription
DMR1:197254001	1	197254001	197255000	1000	1	6.96E-05	-0.6035983	2	0.2	LOC361646	
DMR1:203689001	1	203689001	203690000	1000	1	5.69E-05	0.4978465	22	2.2		
DMR1:205572001	1	205572001	205573000	1000	1	8.22E-07	-0.8124901	5	0.5		
DMR1:208919001	1	208919001	208920000	1000	1	7.94E-05	0.6109308	6	0.6		
DMR1:214530001	1	214530001	214531000	1000	1	1.50E-05	0.5376348	10	1	U6;Ap2a2	Receptor
DMR1:217492001	1	217492001	217493000	1000	1	9.51E-06	0.5879407	10	1	Shank2	Protein Binding
DMR1:231035001	1	231035001	231036000	1000	1	8.56E-06	-0.5962656	3	0.3		
DMR1:233561001	1	233561001	233562000	1000	1	3.88E-06	0.425618	12	1.2	Gnaq	Signaling
DMR1:239342001	1	239342001	239343000	1000	1	5.77E-05	-0.7589876	8	0.8		
DMR1:245742001	1	245742001	245743000	1000	1	6.30E-05	0.2980767	4	0.4		
DMR1:247484001	1	247484001	247485000	1000	1	8.70E-05	0.4402441	15	1.5	Insl6;Rln1	Growth Factors & Cytokines;Signaling
DMR1:248734001	1	248734001	248735000	1000	1	3.03E-05	0.6509126	5	0.5	Mbl2	Immune
DMR1:253513001	1	253513001	253514000	1000	1	1.03E-05	-0.896252	8	0.8		
DMR1:270103001	1	270103001	270104000	1000	1	7.05E-05	0.5367418	5	0.5	Sorcs1	Receptor
DMR2:929001	2	929001	930000	1000	1	2.02E-05	-0.8724551	4	0.4		
DMR2:9067001	2	9067001	9068000	1000	1	1.04E-05	-0.8696684	3	0.3	Adgrv1	
DMR2:21829001	2	21829001	21830000	1000	1	4.80E-05	0.7217762	12	1.2	Msh3	DNA Repair
DMR2:40130001	2	40130001	40131000	1000	1	5.97E-05	-0.4223996	1	0.1		
DMR2:57476001	2	57476001	57478000	2000	1	9.83E-05	-0.4890715	13	0.65		
DMR2:59199001	2	59199001	59200000	1000	1	4.04E-06	-0.7290757	10	1		
DMR2:62618001	2	62618001	62619000	1000	1	1.20E-07	-0.8365268	14	1.4	Pdzd2	
DMR2:63990001	2	63990001	63993000	3000	1	5.55E-05	-0.5574715	18	0.6		
DMR2:65998001	2	65998001	65999000	1000	1	4.65E-05	-0.6296242	3	0.3		
DMR2:69519001	2	69519001	69520000	1000	1	6.98E-05	-0.629543	9	0.9	Cdh10	Extracellular Matrix
DMR2:76111001	2	76111001	76113000	2000	1	9.74E-05	-0.6536796	11	0.55		
DMR2:76495001	2	76495001	76496000	1000	1	7.43E-05	-0.7598099	10	1		
DMR2:79573001	2	79573001	79574000	1000	1	1.53E-05	-0.7409051	1	0.1		
DMR2:81387001	2	81387001	81388000	1000	1	2.33E-05	-0.6150769	13	1.3		
DMR2:92934001	2	92934001	92935000	1000	1	7.65E-06	-0.8497992	2	0.2		
DMR2:113217001	2	113217001	113218000	1000	1	3.79E-05	0.4366136	14	1.4	Fndc3b	Cytoskeleton
DMR2:117835001	2	117835001	117836000	1000	1	9.10E-05	0.4018914	18	1.8		
DMR2:118458001	2	118458001	118459000	1000	1	1.68E-05	0.4740721	15	1.5		

DMR2:124270001	2	124270001	124272000	2000	1	4.97E-05	0.4264787	8	0.4	Spata5	Development
DMR2:141269001	2	141269001	141270000	1000	1	5.76E-06	0.4111902	18	1.8		
DMR2:166228001	2	166228001	166229000	1000	1	5.86E-06	-0.8086626	17	1.7	Ppm1l;B3galnt1	Signaling;Golgi
DMR2:168645001	2	168645001	168646000	1000	1	2.60E-05	-0.6727538	5	0.5		
DMR2:172865001	2	172865001	172866000	1000	1	5.84E-05	-0.7985352	6	0.6		
DMR2:182318001	2	182318001	182319000	1000	1	7.04E-05	-0.406129	16	1.6		
DMR2:195007001	2	195007001	195008000	1000	1	4.90E-05	-0.9404002	0	0		
DMR2:199170001	2	199170001	199171000	1000	1	6.03E-05	-0.5759626	13	1.3	Gja5	
DMR2:200685001	2	200685001	200687000	2000	1	1.22E-05	-0.8937544	24	1.2	Hsd3b5;Hsd3b3	Metabolism
DMR2:202832001	2	202832001	202833000	1000	1	4.44E-06	0.8037611	12	1.2		
DMR2:207526001	2	207526001	207527000	1000	1	2.51E-05	0.449327	9	0.9	Cttnbp2nl	
DMR2:207693001	2	207693001	207696000	3000	1	5.38E-05	0.5660154	22	0.733		
DMR2:209381001	2	209381001	209382000	1000	1	2.07E-05	-0.5536613	2	0.2	Lrif1	
DMR2:211938001	2	211938001	211939000	1000	1	1.81E-05	-0.5656549	7	0.7	Slc25a24	Binding Protein
DMR2:235434001	2	235434001	235435000	1000	1	2.28E-05	-0.8622835	3	0.3		
DMR2:235987001	2	235987001	235988000	1000	1	2.31E-06	0.3358431	9	0.9		
DMR2:245020001	2	245020001	245021000	1000	1	4.00E-05	-0.6612118	13	1.3	Stpg2	Development
DMR2:256611001	2	256611001	256613000	2000	1	5.01E-05	-0.8848376	18	0.9	Adgrl4	
DMR2:258690001	2	258690001	258691000	1000	1	1.68E-05	0.6661629	14	1.4		
DMR3:11753001	3	11753001	11754000	1000	1	1.73E-06	-0.5983461	16	1.6	Cdk9;AC142126.1;Mir2861;Sh2d3c	Signaling
DMR3:27382001	3	27382001	27383000	1000	1	7.89E-05	-0.7485327	8	0.8		
DMR3:28342001	3	28342001	28343000	1000	1	2.10E-05	-0.7702982	3	0.3		
DMR3:29129001	3	29129001	29130000	1000	1	7.19E-06	0.7050267	14	1.4	Arhgap15	Signaling
DMR3:31751001	3	31751001	31752000	1000	1	9.56E-05	-0.5290039	9	0.9		
DMR3:37393001	3	37393001	37394000	1000	1	6.24E-05	0.6116809	17	1.7		
DMR3:45322001	3	45322001	45323000	1000	1	5.96E-05	0.5712629	14	1.4	Pkp4	Cytoskeleton
DMR3:54381001	3	54381001	54383000	2000	1	3.24E-06	0.8283497	33	1.65	Stk39	
DMR3:56423001	3	56423001	56424000	1000	1	3.42E-06	-0.4844636	20	2	Myo3b	Cytoskeleton
DMR3:58087001	3	58087001	58088000	1000	1	9.98E-05	-0.8023152	5	0.5	Metap1d	
DMR3:59536001	3	59536001	59538000	2000	1	7.38E-05	0.5716842	23	1.15		
DMR3:61930001	3	61930001	61931000	1000	1	3.76E-05	-0.4666057	23	2.3		
DMR3:73287001	3	73287001	73288000	1000	1	2.29E-05	-0.6298196	3	0.3	AABR07052755.1	
DMR3:77643001	3	77643001	77644000	1000	1	5.46E-06	-0.7716567	4	0.4		
DMR3:80826001	3	80826001	80827000	1000	1	4.27E-05	-0.5212522	7	0.7	Ambra1;Chrm4	Receptor
DMR3:83164001	3	83164001	83165000	1000	1	3.79E-05	-0.7462968	6	0.6	AABR07052897.1	
DMR3:97124001	3	97124001	97126000	2000	1	3.47E-05	-0.5955599	29	1.45	AABR07053283.1	
DMR3:99774001	3	99774001	99775000	1000	1	4.36E-05	-0.6839778	5	0.5		
DMR3:111469001	3	111469001	111470000	1000	1	3.99E-05	-0.6856884	27	2.7	Ndufaf1;Rtf1	
DMR3:111957001	3	111957001	111958000	1000	1	6.81E-05	-0.6626665	15	1.5	Pla2g4e	Metabolism
DMR3:117593001	3	117593001	117594000	1000	1	4.10E-05	-0.5996439	13	1.3	Fbn1	Development
DMR3:128337001	3	128337001	128338000	1000	1	8.26E-05	-0.5987417	7	0.7	Plcb1	Metabolism
DMR3:136748001	3	136748001	136749000	1000	1	2.87E-06	-0.7546292	9	0.9	Kif16b	Cytoskeleton
DMR3:149124001	3	149124001	149126000	2000	1	4.72E-06	0.6374473	22	1.1	Commd7;Dnmt3b	Transcription
DMR3:155726001	3	155726001	155727000	1000	1	8.53E-05	-0.6020075	17	1.7		
DMR3:165619001	3	165619001	165620000	1000	1	5.78E-05	-0.9935127	40	4		
DMR3:169905001	3	169905001	169907000	2000	1	7.49E-05	0.3531993	35	1.75		
DMR4:211001	4	211001	212000	1000	1	6.15E-05	-0.3827307	17	1.7	Htr5a	Receptor
DMR4:31359001	4	31359001	31360000	1000	1	7.68E-05	-0.5852652	9	0.9	LOC100911994	
DMR4:62848001	4	62848001	62849000	1000	1	9.53E-05	-0.4283399	17	1.7	Slc13a4;Fam180a	Transport
DMR4:80102001	4	80102001	80104000	2000	1	6.10E-05	-0.6625502	26	1.3	Osbpl3	Receptor
DMR4:111000001	4	111000001	111001000	1000	1	3.81E-06	-0.8441598	2	0.2	Lrrtm4	Receptor
DMR4:119148001	4	119148001	119150000	2000	1	1.81E-05	0.440834	37	1.85	Gkn1	
DMR4:137308001	4	137308001	137309000	1000	1	5.61E-05	0.3750276	22	2.2		
DMR4:138948001	4	138948001	138949000	1000	1	4.52E-06	-0.6202578	5	0.5		
DMR4:150782001	4	150782001	150784000	2000	1	8.07E-06	-0.7966452	23	1.15	Cacna1c	Metabolism
DMR4:161378001	4	161378001	161380000	2000	1	6.37E-07	0.777093	32	1.6	AABR07062111.1	
DMR4:166001001	4	166001001	166002000	1000	1	4.29E-05	-0.5207088	3	0.3		
DMR4:172567001	4	172567001	172568000	1000	1	3.57E-05	-0.52337	3	0.3		
DMR4:179380001	4	179380001	179381000	1000	1	2.60E-05	0.2937561	17	1.7		
DMR5:698001	5	698001	699000	1000	1	6.84E-05	-0.6822382	4	0.4	Crispld1	Development
DMR5:10840001	5	10840001	10841000	1000	1	8.87E-05	-0.7932938	7	0.7		
DMR5:11912001	5	11912001	11913000	1000	1	4.40E-05	0.6730052	9	0.9		
DMR5:28953001	5	28953001	28954000	1000	1	1.18E-06	-0.7143507	7	0.7		

DMR5:32577001	5	32577001	32578000	1000	1	2.28E-06	-0.8847239	6	0.6		
DMR5:52514001	5	52514001	52515000	1000	1	4.50E-05	-0.4743328	4	0.4		
DMR5:52803001	5	52803001	52804000	1000	1	8.41E-05	-0.4756065	6	0.6		
DMR5:54330001	5	54330001	54331000	1000	1	1.36E-05	-0.6098552	13	1.3	U6	
DMR5:68748001	5	68748001	68749000	1000	1	2.92E-05	-0.6512244	7	0.7	Smc2	Transcription
DMR5:69877001	5	69877001	69878000	1000	1	9.21E-06	0.4527614	23	2.3	Abca1	Receptor
DMR5:72072001	5	72072001	72075000	3000	1	4.11E-05	-0.3735287	29	0.967		
DMR5:76835001	5	76835001	76836000	1000	1	2.75E-05	0.5346322	7	0.7	Hsdl2	Metabolism
DMR5:83286001	5	83286001	83287000	1000	1	5.47E-05	-0.6304658	3	0.3		
DMR5:88712001	5	88712001	88713000	1000	1	2.67E-05	-0.502081	3	0.3		
DMR5:90398001	5	90398001	90399000	1000	1	8.11E-05	-0.5179866	1	0.1		
DMR5:101112001	5	101112001	101113000	1000	1	9.76E-07	0.7017451	9	0.9	Frem1;AABR07049037.1	
DMR5:105712001	5	105712001	105714000	2000	1	6.37E-05	0.4686825	43	2.15		
DMR5:114634001	5	114634001	114635000	1000	1	5.46E-05	0.5670134	12	1.2	Fggy	Signaling
DMR5:115560001	5	115560001	115561000	1000	1	5.80E-05	-0.3674092	8	0.8		
DMR5:121190001	5	121190001	121191000	1000	1	2.62E-05	-0.3891947	8	0.8		
DMR5:123502001	5	123502001	123504000	2000	1	2.21E-05	-0.4974394	30	1.5		
DMR5:123882001	5	123882001	123883000	1000	1	9.15E-05	-0.7989845	7	0.7		
DMR5:153735001	5	153735001	153736000	1000	1	8.48E-05	0.4411397	14	1.4	Ncmap	
DMR5:167044001	5	167044001	167045000	1000	1	5.25E-05	0.7836825	8	0.8		
DMR6:13496001	6	13496001	13497000	1000	1	3.33E-06	-0.8494919	1	0.1		
DMR6:25930001	6	25930001	25931000	1000	1	7.81E-05	-0.5241253	5	0.5	Babam2	
DMR6:29368001	6	29368001	29369000	1000	1	5.05E-06	-0.7573502	10	1	Klhl29	Transcription
DMR6:37407001	6	37407001	37408000	1000	1	9.41E-05	-0.5299635	3	0.3	5S_rRNA	
DMR6:37941001	6	37941001	37942000	1000	1	8.27E-05	-0.7383765	6	0.6		
DMR6:53745001	6	53745001	53746000	1000	1	5.60E-05	-0.7888937	8	0.8	Hdac9	
DMR6:56706001	6	56706001	56707000	1000	1	2.87E-05	-0.6423695	3	0.3		
DMR6:64947001	6	64947001	64948000	1000	1	3.82E-05	-0.8332263	3	0.3		
DMR6:66851001	6	66851001	66852000	1000	1	5.23E-05	-0.799275	7	0.7		
DMR6:69530001	6	69530001	69531000	1000	1	5.37E-05	0.2810358	23	2.3		
DMR6:70149001	6	70149001	70151000	2000	1	3.53E-05	0.4849832	22	1.1		
DMR6:71130001	6	71130001	71131000	1000	1	8.96E-05	-0.756329	4	0.4	Prkd1	Signaling
DMR6:71773001	6	71773001	71775000	2000	1	3.95E-05	0.612768	27	1.35		
DMR6:75111001	6	75111001	75112000	1000	1	6.52E-06	0.5144163	6	0.6	U1	
DMR6:76315001	6	76315001	76317000	2000	1	9.68E-05	0.3601941	29	1.45		
DMR6:79888001	6	79888001	79889000	1000	1	9.03E-05	0.431672	19	1.9		
DMR6:81726001	6	81726001	81727000	1000	1	1.77E-05	0.465683	14	1.4		
DMR6:82276001	6	82276001	82277000	1000	1	3.49E-05	0.5013638	5	0.5		
DMR6:103041001	6	103041001	103042000	1000	1	9.04E-05	-0.4584246	20	2		
DMR6:109572001	6	109572001	109573000	1000	1	2.44E-05	0.5292655	14	1.4	Batf	Transcription
DMR6:109634001	6	109634001	109637000	3000	1	8.92E-05	0.4857958	41	1.367	Flvcr2	Transport
DMR6:112283001	6	112283001	112284000	1000	1	4.59E-05	-0.5170634	6	0.6	Nrxn3;AABR07065161.1	Receptor
DMR6:114824001	6	114824001	114825000	1000	1	9.63E-05	0.7221871	6	0.6	Cep128	
DMR6:121180001	6	121180001	121181000	1000	1	5.78E-05	-0.5046606	4	0.4		
DMR6:134518001	6	134518001	134519000	1000	1	2.21E-06	0.7257987	12	1.2		
DMR7:14452001	7	14452001	14453000	1000	1	8.12E-05	-0.5607848	18	1.8	Cyp4f39	Metabolism
DMR7:14657001	7	14657001	14658000	1000	1	3.31E-06	-0.6213052	14	1.4		
DMR7:14926001	7	14926001	14927000	1000	1	2.21E-05	0.4119475	7	0.7		
DMR7:18437001	7	18437001	18438000	1000	1	3.88E-05	0.5611618	12	1.2	Adamts10;Myo1f	Protease;Cytoskeleton
DMR7:21782001	7	21782001	21784000	2000	1	1.52E-06	-0.6079553	6	0.3		
DMR7:30822001	7	30822001	30823000	1000	1	6.14E-05	0.4645876	8	0.8	Anks1b	Receptor
DMR7:38040001	7	38040001	38041000	1000	1	3.18E-05	-0.6783348	8	0.8		
DMR7:53434001	7	53434001	53435000	1000	1	6.95E-05	-0.4139594	5	0.5		
DMR7:54216001	7	54216001	54217000	1000	1	5.33E-05	0.3998642	11	1.1	Nap1l1;Gm25117	Signaling
DMR7:56203001	7	56203001	56204000	1000	1	3.51E-05	-0.8335988	8	0.8		
DMR7:56277001	7	56277001	56278000	1000	1	3.80E-05	-0.7632473	5	0.5	AABR07057116.1	
DMR7:60206001	7	60206001	60207000	1000	1	5.49E-05	0.4831389	9	0.9	Frs2	Unknown
DMR7:60460001	7	60460001	60461000	1000	1	1.81E-05	-0.9444202	17	1.7		
DMR7:61317001	7	61317001	61319000	2000	1	3.80E-05	-0.7676277	6	0.3		
DMR7:65780001	7	65780001	65781000	1000	1	4.96E-05	-0.8119167	5	0.5		
DMR7:83560001	7	83560001	83561000	1000	1	2.78E-05	-0.6167302	6	0.6	Ebag9	Receptor
DMR7:93410001	7	93410001	93411000	1000	1	2.03E-05	0.731046	5	0.5	Samd12	Unknown
DMR7:105181001	7	105181001	105183000	2000	1	2.89E-05	-0.9102652	11	0.55		
DMR7:121126001	7	121126001	121127000	1000	1	3.00E-05	-0.8624248	17	1.7	Apobec3;AC128476.2;Cbx7	Metabolism;Transcription
DMR7:128948001	7	128948001	128949000	1000	1	9.19E-05	0.3424388	9	0.9		

DMR7:132729001	7	132729001	132730000	1000	1	5.69E-06	-0.9122642	8	0.8	Slc2a13	Transport
DMR8:978001	8	978001	979000	1000	1	8.66E-06	-0.7714431	4	0.4		
DMR8:3552001	8	3552001	3553000	1000	1	1.70E-05	-0.6750696	1	0.1		
DMR8:22999001	8	22999001	2.30E+07	1000	1	3.74E-05	-0.8075279	52	5.2	Rgl3;Ccdc151	Signaling
DMR8:24436001	8	24436001	24437000	1000	1	9.03E-05	-0.6041955	13	1.3	Bmper	Development
DMR8:32082001	8	32082001	32083000	1000	1	1.72E-05	-0.6417817	6	0.6		
DMR8:40443001	8	40443001	40445000	2000	1	2.08E-05	0.455309	11	0.55	Olr1203	Receptor
DMR8:64429001	8	64429001	64430000	1000	1	8.48E-05	-0.7101341	9	0.9	Celf6	Transcription
DMR8:73374001	8	73374001	73375000	1000	1	6.34E-05	-0.6527849	8	0.8		
DMR8:91378001	8	91378001	91380000	2000	1	2.82E-06	-0.4698935	21	1.05	Ttk	Signaling
DMR8:107231001	8	107231001	107232000	1000	1	3.87E-06	0.5714468	24	2.4	Faim	
DMR8:107996001	8	107996001	107998000	2000	1	5.17E-05	0.532465	27	1.35		
DMR8:112712001	8	112712001	112713000	1000	1	2.91E-05	0.6443391	12	1.2	Dnajc13	Protein Binding
DMR8:128648001	8	128648001	128649000	1000	1	5.11E-05	-0.4273143	21	2.1	Ttc21a	
DMR9:2026001	9	2026001	2027000	1000	1	1.36E-05	-0.7123366	6	0.6		
DMR9:3799001	9	3799001	3800000	1000	1	5.48E-05	0.4981515	9	0.9		
DMR9:6223001	9	6223001	6224000	1000	1	4.44E-06	-0.8773064	3	0.3		
DMR9:11644001	9	11644001	11645000	1000	1	2.66E-05	-0.5166887	6	0.6		
DMR9:46390001	9	46390001	46391000	1000	1	4.48E-05	0.3885813	18	1.8	Creg2	
DMR9:61885001	9	61885001	61886000	1000	1	4.70E-05	0.5154001	7	0.7	Boll	Development
DMR9:86474001	9	86474001	86475000	1000	1	6.18E-05	0.5584573	9	0.9	AABR07068161.1	
DMR9:101394001	9	101394001	101395000	1000	1	2.24E-05	-0.5333877	19	1.9	Chchd2	Transcription
DMR9:115288001	9	115288001	115291000	3000	1	7.96E-05	0.3079521	44	1.467	Ptprm	Receptor
DMR10:710001	10	710001	712000	2000	1	1.43E-05	-0.7984021	11	0.55		
DMR10:10323001	10	10323001	10324000	1000	1	3.59E-05	0.3314876	15	1.5		
DMR10:17278001	10	17278001	17280000	2000	1	6.67E-05	-0.618654	28	1.4	Sh3pxd2b	
DMR10:17850001	10	17850001	17852000	2000	1	3.16E-05	-0.7511466	35	1.75		
DMR10:27598001	10	27598001	27599000	1000	1	3.71E-07	-0.5086068	6	0.6		
DMR10:28262001	10	28262001	28263000	1000	1	8.10E-05	-0.683295	8	0.8		
DMR10:30804001	10	30804001	30805000	1000	1	4.18E-05	-0.8908961	14	1.4		
DMR10:62659001	10	62659001	62660000	1000	1	3.87E-05	-0.6435826	17	1.7	Tp53i13;Git1	Transcription
DMR10:64496001	10	64496001	64497000	1000	1	6.41E-05	0.4173983	10	1	Nxn	Signaling
DMR10:74079001	10	74079001	74080000	1000	1	2.68E-05	-0.7971757	6	0.6	Cltc;Dhx40	Cytoskeleton;Transcription
DMR10:79403001	10	79403001	79404000	1000	1	2.72E-05	-0.748428	7	0.7		
DMR10:82258001	10	82258001	82260000	2000	1	6.29E-05	0.298283	27	1.35	Mycbpap	
DMR10:88111001	10	88111001	88112000	1000	1	8.73E-05	-0.6437305	8	0.8	Krt14;Krt9;Krt17	Cytoskeleton
DMR10:95245001	10	95245001	95246000	1000	1	3.54E-05	-0.7710907	6	0.6	AABR07030598.1;RGD1565033;Bptf	Unknown;Metabolism
DMR10:100504001	10	100504001	100505000	1000	1	3.31E-05	-0.5364573	13	1.3		
DMR10:109015001	10	109015001	109016000	1000	1	2.91E-05	0.6376324	7	0.7	Rptor	
DMR11:3020001	11	3020001	3021000	1000	1	7.60E-05	0.6196269	11	1.1	U6	
DMR11:12740001	11	12740001	12741000	1000	1	2.15E-05	-0.5245868	10	1		
DMR11:19274001	11	19274001	19275000	1000	1	7.31E-05	-0.7999423	6	0.6		
DMR11:30218001	11	30218001	30219000	1000	1	4.71E-05	-0.691115	17	1.7		
DMR11:36347001	11	36347001	36348000	1000	1	1.94E-05	0.6302801	9	0.9	Psmg1;Brwd1	
DMR11:36660001	11	36660001	36661000	1000	1	7.27E-05	-0.7703329	1	0.1	B3galt5	Metabolism
DMR11:37144001	11	37144001	37145000	1000	1	2.71E-05	0.6241987	7	0.7	Dscam	Extracellular Matrix
DMR11:41708001	11	41708001	41710000	2000	1	8.91E-05	-0.6402817	21	1.05		
DMR11:50572001	11	50572001	50573000	1000	1	1.90E-05	0.5702615	9	0.9		
DMR11:61804001	11	61804001	61805000	1000	1	8.23E-06	-0.883568	8	0.8	Gramd1c;AC114526.2	
DMR11:73268001	11	73268001	73269000	1000	1	5.65E-05	-0.3886398	20	2	Xxylt1	
DMR12:4835001	12	4835001	4837000	2000	1	3.33E-05	-0.6691649	6	0.3		
DMR12:7349001	12	7349001	7350000	1000	1	9.79E-05	-0.669871	9	0.9	Metazoa_SRP;AABR07035224.1	
DMR12:9372001	12	9372001	9373000	1000	1	2.59E-05	-0.423091	18	1.8	Flt3	Receptor
DMR12:25873001	12	25873001	25874000	1000	1	3.86E-05	0.4949726	16	1.6		
DMR12:28240001	12	28240001	28241000	1000	1	5.77E-05	0.6089925	7	0.7	Auts2	Development
DMR12:36044001	12	36044001	36046000	2000	1	4.95E-05	-0.4329375	17	0.85	Tmem132b	Unknown
DMR12:37701001	12	37701001	37703000	2000	1	5.67E-05	-0.4144552	23	1.15	RGD1563482;Mphosph9	Transcription;Cell Cycle
DMR12:40906001	12	40906001	40908000	2000	1	6.72E-05	-0.8047945	25	1.25	Ptpn11	
DMR13:2407001	13	2407001	2410000	3000	1	4.91E-05	-0.5587864	36	1.2		
DMR13:14103001	13	14103001	14104000	1000	1	2.63E-07	-0.6664538	5	0.5		
DMR13:52118001	13	52118001	52120000	2000	1	3.66E-05	0.5406726	23	1.15	Rnpep;Timm17a	Metabolism

DMR13:61101001	13	61101001	61103000	2000	1	6.04E-06	-0.8550245	5	0.25		
DMR13:61539001	13	61539001	61540000	1000	1	1.63E-05	-0.530645	7	0.7		
DMR13:92173001	13	92173001	92174000	1000	1	6.03E-05	-0.6646624	5	0.5	Olr1589;Olr1590	Receptor
DMR13:95226001	13	95226001	95228000	2000	1	8.81E-05	0.3246712	12	0.6	Akt3	Signaling
DMR13:96607001	13	96607001	96610000	3000	1	5.20E-05	0.5682877	33	1.1		
DMR13:96951001	13	96951001	96953000	2000	1	2.96E-05	0.5911688	29	1.45	Kif26b	Cytoskeleton
DMR13:97714001	13	97714001	97715000	1000	1	8.76E-05	0.7337883	11	1.1	LOC103689999	
DMR13:105844001	13	105844001	105845000	1000	1	3.29E-05	0.4754354	7	0.7	AABR07022072.2	
DMR14:10915001	14	10915001	10917000	2000	1	5.96E-05	-0.5290805	20	1	Sec31a	Transport
DMR14:36524001	14	36524001	36525000	1000	1	4.92E-06	-0.649176	10	1	Scfd2	Unknown
DMR14:50209001	14	50209001	50210000	1000	1	1.20E-05	-0.8910592	4	0.4		
DMR14:63253001	14	63253001	63254000	1000	1	6.33E-05	-0.3823569	15	1.5		
DMR14:63321001	14	63321001	63322000	1000	1	4.40E-05	-0.5617492	3	0.3		
DMR14:94643001	14	94643001	94645000	2000	1	5.49E-05	-0.475864	8	0.4		
DMR14:101335001	14	101335001	101336000	1000	1	1.15E-05	-0.6455051	10	1		
DMR14:105441001	14	105441001	105442000	1000	1	2.71E-05	-0.4481655	9	0.9	AABR07016586.1	
DMR14:105664001	14	105664001	105666000	2000	1	2.95E-05	-0.84987	15	0.75		
DMR14:107124001	14	107124001	107125000	1000	1	3.16E-05	-0.8382782	20	2	Ehbp1	Unknown
DMR15:826001	15	826001	827000	1000	1	9.20E-05	-0.3711648	8	0.8	Kcnma1	Metabolism
DMR15:9025001	15	9025001	9026000	1000	1	9.59E-06	0.7389738	13	1.3	Thrb	
DMR15:10522001	15	10522001	10523000	1000	1	6.93E-05	-0.4303526	13	1.3		
DMR15:13734001	15	13734001	13736000	2000	1	2.71E-05	0.5252944	19	0.95		
DMR15:40222001	15	40222001	40223000	1000	1	9.70E-05	0.3686662	8	0.8	Atp8a2	Transport
DMR15:73633001	15	73633001	73634000	1000	1	6.37E-07	-0.9402633	2	0.2		
DMR15:90530001	15	90530001	90531000	1000	1	6.43E-05	-0.80461	9	0.9	Mycbp2	Metabolism
DMR15:99520001	15	99520001	99522000	2000	1	7.80E-05	-0.7914149	13	0.65		
DMR15:103354001	15	103354001	103355000	1000	1	2.18E-05	-0.5211018	10	1	Gpr180	
DMR15:103365001	15	103365001	103366000	1000	1	8.20E-05	0.5175108	20	2	Gpr180	
DMR15:106161001	15	106161001	106164000	3000	1	1.79E-05	0.9163179	21	0.7	AABR07019449.1	
DMR16:4071001	16	4071001	4072000	1000	1	8.11E-05	0.5840157	9	0.9		
DMR16:5661001	16	5661001	5663000	2000	1	7.97E-05	-0.4847627	35	1.75	Cacna2d3	Transport
DMR16:15658001	16	15658001	15659000	1000	1	6.25E-05	-0.7348594	4	0.4	5S_rRNA	
DMR16:31255001	16	31255001	31256000	1000	1	9.34E-05	-0.5557199	12	1.2	AABR07025272.1	
DMR16:31863001	16	31863001	31864000	1000	1	4.10E-05	0.4380709	12	1.2	AABR07025295.1	
DMR16:48458001	16	48458001	48459000	1000	1	4.50E-05	0.3907019	11	1.1		
DMR16:54080001	16	54080001	54081000	1000	1	1.74E-05	-0.6135934	8	0.8	Pcm1	Unknown
DMR16:71775001	16	71775001	71777000	2000	1	1.33E-05	-0.7587918	25	1.25	Plekha2	
DMR16:77694001	16	77694001	77695000	1000	1	4.71E-05	-0.9453709	3	0.3		
DMR16:80950001	16	80950001	80951000	1000	1	1.48E-07	0.6814103	15	1.5	LOC103693999	
DMR17:17504001	17	17504001	17505000	1000	1	7.94E-06	-0.7363107	4	0.4		
DMR17:20302001	17	20302001	20303000	1000	1	2.28E-05	-0.8221487	5	0.5	Jarid2	Epigenetic
DMR17:20383001	17	20383001	20384000	1000	1	7.21E-05	-0.8062024	20	2		
DMR17:20724001	17	20724001	20725000	1000	1	2.27E-05	-0.5470864	7	0.7		
DMR17:29734001	17	29734001	29735000	1000	1	4.71E-05	-0.6497593	12	1.2	Cdyl	Metabolism
DMR17:48803001	17	48803001	48804000	1000	1	1.71E-05	-0.7101976	7	0.7	Vps41	Transport
DMR17:54725001	17	54725001	54727000	2000	1	8.76E-05	0.4722282	16	0.8		
DMR17:65166001	17	65166001	65167000	1000	1	9.25E-05	-0.5592668	4	0.4		
DMR17:69779001	17	69779001	69780000	1000	1	4.18E-05	0.7625334	11	1.1	Akr1c3	Metabolism
DMR17:76230001	17	76230001	76231000	1000	1	1.26E-05	0.4044602	10	1	Upf2	Transcription
DMR17:87124001	17	87124001	87125000	1000	1	8.84E-05	-0.695955	4	0.4		
DMR17:87184001	17	87184001	87185000	1000	1	5.76E-05	0.553443	7	0.7		
DMR17:88235001	17	88235001	88237000	2000	1	1.24E-05	0.5052064	20	1	Gpr158	Receptor
DMR18:12079001	18	12079001	12080000	1000	1	3.20E-05	0.4029352	7	0.7	Dsg4	Extracellular Matrix
DMR18:34546001	18	34546001	34547000	1000	1	8.44E-05	-0.6749159	14	1.4		
DMR18:52326001	18	52326001	52327000	1000	1	1.64E-05	-0.7911563	12	1.2	Megf10	Extracellular Matrix
DMR18:55432001	18	55432001	55433000	1000	1	3.57E-05	0.3284183	42	4.2		
DMR18:55969001	18	55969001	55970000	1000	1	2.17E-06	-0.8649885	12	1.2	Ndst1	Metabolism
DMR18:61966001	18	61966001	61967000	1000	1	6.97E-05	0.3970444	22	2.2		
DMR18:64641001	18	64641001	64643000	2000	1	1.40E-05	-0.6364171	13	0.65		
DMR18:68616001	18	68616001	68617000	1000	1	8.97E-05	0.3603521	10	1	AABR07032496.1	
DMR18:76192001	18	76192001	76193000	1000	1	7.64E-05	-0.652097	12	1.2		
DMR18:80742001	18	80742001	80743000	1000	1	5.04E-05	-0.6061234	18	1.8		
DMR19:5962001	19	5962001	5963000	1000	1	4.39E-05	0.4237041	10	1		
DMR19:6461001	19	6461001	6463000	2000	1	4.09E-05	-0.5261174	26	1.3		

DMR19:11295001	19	11295001	11296000	1000	1	9.48E-05	0.498321	19	1.9	AC128848.1	
DMR19:27558001	19	27558001	27559000	1000	1	2.58E-05	0.3854115	7	0.7		
DMR19:30950001	19	30950001	30951000	1000	1	9.82E-05	0.4358055	17	1.7	Smarca5	Epigenetic
DMR19:38149001	19	38149001	38150000	1000	1	4.20E-05	0.5597008	5	0.5	Pla2g15;AC128800.1	Metabolism
DMR19:40075001	19	40075001	40076000	1000	1	8.18E-05	0.4339826	12	1.2		
DMR19:41013001	19	41013001	41014000	1000	1	6.96E-05	0.6444346	10	1	Vac14	Receptor
DMR19:46333001	19	46333001	46334000	1000	1	5.52E-05	-0.5939199	3	0.3		
DMR19:50526001	19	50526001	50528000	2000	1	7.52E-05	0.4773708	41	2.05		
DMR19:56798001	19	56798001	56800000	2000	1	7.97E-06	-0.6592802	24	1.2	Taf5l;Urb2	Translation;Development
DMR20:1985001	20	1985001	1986000	1000	1	5.39E-05	-0.5872565	20	2	Gabbr1;AC108572.3	Receptor
DMR20:4231001	20	4231001	4232000	1000	1	9.40E-05	0.6801145	13	1.3	Tsbp1;RGD1624210;AABR07044388.5	Immune
DMR20:14758001	20	14758001	14759000	1000	1	1.02E-05	-0.7240155	9	0.9	AC141961.1	
DMR20:25644001	20	25644001	25646000	2000	1	4.39E-06	-0.7302607	9	0.45	AABR07044900.1	
DMR20:39936001	20	39936001	39937000	1000	1	5.73E-05	-0.4511694	4	0.4		
DMR20:42516001	20	42516001	42517000	1000	1	5.79E-05	-0.5248516	10	1	U6	
DMRX:13076001	X	13076001	13077000	1000	1	5.06E-05	0.7275453	8	0.8		
DMRX:18871001	X	18871001	18872000	1000	1	3.00E-05	0.4516599	2	0.2	Klf8	Transcription
DMRX:20468001	X	20468001	20469000	1000	1	1.58E-05	-0.7617333	5	0.5		
DMRX:44626001	X	44626001	44627000	1000	1	4.11E-06	-0.7027323	9	0.9		
DMRX:54322001	X	54322001	54323000	1000	1	4.89E-05	-0.5314672	5	0.5		
DMRX:122437001	X	122437001	122439000	2000	1	6.49E-05	0.361654	31	1.55		
DMRX:127801001	X	127801001	127802000	1000	1	4.92E-05	0.6944038	7	0.7	Gria3;AABR07041374.1	Signaling
DMRX:135879001	X	135879001	135880000	1000	1	8.15E-05	0.4072373	20	2		
DMRX:149987001	X	149987001	149989000	2000	1	3.62E-05	-0.6008401	11	0.55		
DMRX:150468001	X	150468001	150470000	2000	1	9.11E-05	-0.7957886	11	0.55		
