# Supplementary material for: Epigenome-wide association study for atrazine induced transgenerational DNA methylation and histone retention sperm epigenetic biomarkers for disease
Source: PLoS One. 2020 Dec 16;15(12):e0239380. doi: 10.1371/journal.pone.0239380 (PMC7743986; doi:10.1371/journal.pone.0239380)
Supplement: S7 Table — DHR name, chromosome, start, stop, length, number signature windows, minimum p-value, max log-fold change, CpG number, CpG density, gene annotation, and gene category are presented. (PDF) [file pone.0239380.s014.pdf]

Supplemental Table S7
DHR Site List Lean p<1e-05

DHR Name	Chr	Start	Stop	Length	# Sig Win	minP	maxLFC	CpG #	CpG Density	Gene Annotation	Gene Category
DHR1:175001	1	175001	176000	1000	1	8.97E-07	0.6904805	9	0.9		
DHR1:14997001	1	14997001	14998000	1000	1	3.25E-06	0.9138199	16	1.6		
DHR1:24432001	1	24432001	24433000	1000	1	5.01E-08	0.8983316	4	0.4		
DHR1:24686001	1	24686001	24687000	1000	1	9.89E-06	0.9158629	9	0.9		
DHR1:25938001	1	25938001	25940000	2000	1	9.89E-07	1.3368908	67	3.35		
DHR1:27676001	1	27676001	27677000	1000	1	1.23E-06	-1.4474879	2	0.2	Nkain2	Transport
DHR1:29254001	1	29254001	29255000	1000	1	9.65E-06	0.910554	13	1.3	Ncoa7	
DHR1:43666001	1	43666001	43667000	1000	1	9.66E-06	-1.3168746	2	0.2	Oprm1;AABR07001433.1	Receptor
DHR1:43852001	1	43852001	43854000	2000	1	6.47E-06	0.9428896	14	0.7	Cnksr3	Signaling
DHR1:53901001	1	53901001	53904000	3000	1	4.41E-08	0.9841509	37	1.233	AABR07001592.2	
DHR1:54499001	1	54499001	54501000	2000	1	1.47E-06	-2.1587265	5	0.25	AABR07001640.1	
DHR1:60582001	1	60582001	60584000	2000	1	9.45E-06	-1.5319049	6	0.3		
DHR1:62113001	1	62113001	62114000	1000	1	5.17E-06	-1.3386302	16	1.6	AABR07001923.1	
DHR1:67337001	1	67337001	67338000	1000	1	1.00E-06	1.1143191	31	3.1	LOC100362054	
DHR1:75369001	1	75369001	75371000	2000	1	1.48E-07	-1.9369636	11	0.55	Lig1	Transcription
DHR1:78823001	1	78823001	78825000	2000	1	8.46E-06	1.0513482	17	0.85	Gng8;Ptgir	Signaling;Receptor
DHR1:80850001	1	80850001	80852000	2000	1	4.25E-06	1.117747	44	2.2	Igsf23	
DHR1:83491001	1	83491001	83493000	2000	1	2.95E-06	0.9750265	12	0.6		
DHR1:85929001	1	85929001	85931000	2000	1	4.66E-06	-1.5463337	7	0.35		
DHR1:92137001	1	92137001	92142000	5000	1	3.71E-09	1.1987578	81	1.62		
DHR1:98287001	1	98287001	98288000	1000	1	2.70E-06	-1.353102	6	0.6		
DHR1:101485001	1	101485001	101486000	1000	1	9.02E-06	1.0138652	8	0.8	Dhdh;Tulp2;Nucb1	Metabolism;Transcription
DHR1:102844001	1	102844001	102846000	2000	1	5.34E-09	1.20797	40	2	Saa4;Gtf2h1	Transport;Transcription
DHR1:113743001	1	113743001	113746000	3000	1	3.84E-07	-1.5993622	11	0.367	AABR07003510.1	
DHR1:116595001	1	116595001	116598000	3000	1	3.40E-06	-1.3312776	5	0.167	Ube3a	Metabolism
DHR1:122543001	1	122543001	122544000	1000	1	3.08E-06	-1.3787446	3	0.3		
DHR1:128279001	1	128279001	128280000	1000	1	1.18E-06	-1.2528844	7	0.7	Mef2a	Transcription
DHR1:140778001	1	140778001	140780000	2000	1	6.18E-06	0.9869065	19	0.95	Acan	Extracellular Matrix
DHR1:150720001	1	150720001	150721000	1000	1	4.72E-06	-1.0839969	1	0.1		
DHR1:155585001	1	155585001	155586000	1000	1	6.87E-07	-1.4794752	7	0.7		
DHR1:159666001	1	159666001	159668000	2000	1	9.35E-07	-1.3413719	8	0.4		
DHR1:165843001	1	165843001	165846000	3000	1	3.44E-06	-0.7889515	26	0.867	Fam168a	
DHR1:168397001	1	168397001	168399000	2000	1	7.11E-06	-1.1163191	7	0.35	Olr87;Olr88	Receptor
DHR1:189379001	1	189379001	189381000	2000	1	7.77E-06	-1.1863235	16	0.8	Acsm2	Metabolism
DHR1:198641001	1	198641001	198645000	4000	1	4.80E-06	1.060546	59	1.475	Cd2bp2;Tbc1d10b	Transcription;Signaling
DHR1:200296001	1	200296001	200297000	1000	1	2.85E-07	1.4137926	8	0.8		
DHR1:202591001	1	202591001	202592000	1000	1	6.98E-06	-1.5939303	1	0.1		
DHR1:205758001	1	205758001	205761000	3000	1	2.41E-06	1.0909489	37	1.233	Mmp21;Uros	Protease;Metabolism
DHR1:209776001	1	209776001	209777000	1000	1	6.49E-06	0.9177143	10	1	Glrx3	Electron Transport
DHR1:217617001	1	217617001	217618000	1000	1	3.14E-06	1.3013129	24	2.4	Cttn	Cytoskeleton
DHR1:217941001	1	217941001	217943000	2000	1	7.02E-06	1.0681172	23	1.15	AC095937.1	
DHR1:224091001	1	224091001	224093000	2000	1	1.00E-07	-1.2915663	7	0.35		
DHR1:229864001	1	229864001	229866000	2000	1	1.41E-06	-1.2653695	3	0.15		
DHR1:234073001	1	234073001	234076000	3000	1	5.28E-06	-1.3876922	22	0.733		
DHR1:237332001	1	237332001	237334000	2000	1	2.30E-06	-1.1947816	11	0.55		
DHR1:238442001	1	238442001	238443000	1000	1	4.06E-08	-1.6169347	9	0.9	Tmc1	
DHR1:243636001	1	243636001	243639000	3000	1	4.97E-07	1.0494862	59	1.967		
DHR1:262213001	1	262213001	262214000	1000	1	7.22E-06	-0.9534454	3	0.3		
DHR1:263399001	1	263399001	263400000	1000	1	4.62E-06	0.8980702	13	1.3		
DHR1:272156001	1	272156001	272158000	2000	1	5.49E-06	-1.5019023	9	0.45		
DHR1:275288001	1	275288001	275289000	1000	1	5.90E-06	-1.1523391	3	0.3		
DHR1:280835001	1	280835001	280837000	2000	1	2.40E-06	0.8413799	37	1.85		
DHR2:5226001	2	5226001	5228000	2000	1	8.39E-06	-1.9980382	5	0.25	Fam172a	Unknown
DHR2:15504001	2	15504001	15505000	1000	1	5.20E-06	-1.4378341	5	0.5		
DHR2:34326001	2	34326001	34327000	1000	1	4.91E-06	0.9632708	5	0.5	Cenpk	Cell Cycle
DHR2:36988001	2	36988001	36989000	1000	1	8.94E-06	-1.3008798	2	0.2		
DHR2:42038001	2	42038001	42040000	2000	1	8.41E-06	-1.248826	8	0.4		
DHR2:48766001	2	48766001	48767000	1000	1	7.42E-06	-1.0771915	3	0.3		
DHR2:54301001	2	54301001	54303000	2000	1	7.87E-07	-1.0491576	10	0.5	Plcxd3	
DHR2:55799001	2	55799001	55800000	1000	1	2.79E-06	-1.4941357	4	0.4	C9	Immune
DHR2:61918001	2	61918001	61919000	1000	1	4.52E-06	-1.4192618	2	0.2	Npr3	Receptor
DHR2:66656001	2	66656001	66658000	2000	1	5.61E-08	-1.7467087	6	0.3		
DHR2:67446001	2	67446001	67452000	6000	1	4.15E-06	-1.5325485	37	0.617		
DHR2:70350001	2	70350001	70353000	3000	1	7.00E-06	-1.0456407	19	0.633		

DHR2:75291001	2	75291001	75293000	2000	1	6.68E-06	-1.5541176	4	0.2		
DHR2:76067001	2	76067001	76077000	10000	1	7.66E-06	-1.9922643	44	0.44	U1	
DHR2:90007001	2	90007001	90008000	1000	1	1.48E-06	-1.2578619	2	0.2		
DHR2:90165001	2	90165001	90167000	2000	1	1.39E-06	-1.301819	6	0.3		
DHR2:96943001	2	96943001	96945000	2000	1	7.18E-06	-1.4154469	6	0.3		
DHR2:127049001	2	127049001	127050000	1000	1	2.47E-06	-1.1348125	6	0.6		
DHR2:132813001	2	132813001	132814000	1000	1	2.52E-06	-1.2998916	4	0.4		
DHR2:138351001	2	138351001	138354000	3000	1	6.48E-06	-1.5326711	30	1		
DHR2:139356001	2	139356001	139358000	2000	1	3.19E-06	-1.3053107	11	0.55		
DHR2:139717001	2	139717001	139720000	3000	1	6.63E-06	0.9584164	23	0.767		
DHR2:146805001	2	146805001	146808000	3000	1	6.39E-06	-1.0687496	13	0.433		
DHR2:151115001	2	151115001	151116000	1000	1	4.86E-06	-1.4256608	2	0.2		
DHR2:152205001	2	152205001	152206000	1000	1	2.98E-06	-1.464597	2	0.2		
DHR2:152947001	2	152947001	152948000	1000	1	3.74E-07	-1.4264093	3	0.3		
DHR2:153032001	2	153032001	153033000	1000	1	4.33E-06	0.9298896	8	0.8		
DHR2:153101001	2	153101001	153103000	2000	1	1.77E-06	-1.2544595	12	0.6		
DHR2:162124001	2	162124001	162125000	1000	1	8.59E-06	0.9646362	8	0.8		
DHR2:162362001	2	162362001	162363000	1000	1	6.00E-06	-1.222288	2	0.2		
DHR2:168096001	2	168096001	168101000	5000	1	5.01E-06	-1.5360041	37	0.74		
DHR2:171787001	2	171787001	171790000	3000	1	7.88E-06	-1.1219829	32	1.067		
DHR2:175835001	2	175835001	175837000	2000	1	7.51E-06	-1.5492739	8	0.4		
DHR2:185199001	2	185199001	185200000	1000	1	1.23E-06	0.9844025	12	1.2	AABR07012092.1	
DHR2:187761001	2	187761001	187764000	3000	1	4.59E-07	0.9153662	36	1.2	Pmf1	
DHR2:189839001	2	189839001	189841000	2000	1	9.57E-06	1.1841249	22	1.1	Npr1	Receptor
DHR2:190852001	2	190852001	190854000	2000	1	1.01E-06	-2.122228	7	0.35		
DHR2:200747001	2	200747001	200748000	1000	1	5.37E-06	-1.3200323	2	0.2	Hsd3b3	Metabolism
DHR2:204713001	2	204713001	204714000	1000	1	5.48E-07	-0.7965941	10	1		
DHR2:214605001	2	214605001	214606000	1000	1	1.26E-06	-1.981346	4	0.4		
DHR2:215412001	2	215412001	215416000	4000	1	3.99E-06	-1.5679011	19	0.475		
DHR2:216218001	2	216218001	216220000	2000	1	4.30E-06	-1.1157494	5	0.25		
DHR2:224695001	2	224695001	224696000	1000	1	6.99E-06	-1.0087293	2	0.2		
DHR2:225941001	2	225941001	225944000	3000	1	2.03E-06	0.9580321	41	1.367	AABR07013167.1	
DHR2:226965001	2	226965001	226966000	1000	1	6.43E-07	-1.4421797	8	0.8	Pde5a	Metabolism
DHR2:228140001	2	228140001	228142000	2000	1	2.31E-06	-1.1462279	11	0.55		
DHR2:244705001	2	244705001	244708000	3000	1	4.21E-06	-1.3446314	22	0.733	Stpg2	Development
DHR2:245964001	2	245964001	245965000	1000	1	7.64E-06	-1.069649	10	1		
DHR2:247005001	2	247005001	247006000	1000	1	2.42E-06	-1.1819925	1	0.1		
DHR2:253349001	2	253349001	253350000	1000	1	4.16E-06	-1.3921133	4	0.4		
DHR2:257845001	2	257845001	257846000	1000	1	7.36E-06	0.914084	8	0.8	Ak5	Signaling
DHR2:259581001	2	259581001	259583000	2000	1	5.46E-07	0.8488049	30	1.5	St6galnac3	Metabolism
DHR2:265097001	2	265097001	265098000	1000	1	9.39E-06	-1.7996534	11	1.1	Lrrc7	Unknown
DHR2:266324001	2	266324001	266326000	2000	1	1.58E-08	-1.4991428	7	0.35	Wls	
DHR3:15648001	3	15648001	15650000	2000	1	6.58E-06	-0.7200518	11	0.55		
DHR3:18256001	3	18256001	18259000	3000	1	3.30E-06	-1.3884059	11	0.367		
DHR3:23695001	3	23695001	23697000	2000	1	4.12E-06	-2.2518778	4	0.2		
DHR3:23836001	3	23836001	23837000	1000	1	8.88E-08	-1.6033225	2	0.2		
DHR3:26239001	3	26239001	26240000	1000	1	7.94E-06	-1.4773409	6	0.6		
DHR3:31784001	3	31784001	31786000	2000	1	6.28E-06	-1.1642903	10	0.5		
DHR3:35374001	3	35374001	35375000	1000	1	3.22E-06	1.1494254	2	0.2	Lypd6b	
DHR3:39444001	3	39444001	39445000	1000	1	4.44E-06	-1.0963453	3	0.3		
DHR3:41168001	3	41168001	41169000	1000	1	3.58E-06	-1.0694453	11	1.1	Kcnj3	Transport
DHR3:41578001	3	41578001	41579000	1000	1	9.68E-06	-1.0025208	7	0.7		
DHR3:45465001	3	45465001	45468000	3000	1	9.89E-07	0.8201837	38	1.267		
DHR3:47897001	3	47897001	47898000	1000	1	8.28E-06	-1.1603891	2	0.2		
DHR3:52398001	3	52398001	52399000	1000	1	3.14E-06	-1.4291608	0	0	Scn1a	Metabolism
DHR3:61689001	3	61689001	61691000	2000	1	5.42E-06	-1.4445172	29	1.45	Hoxd1	Transcription
DHR3:69674001	3	69674001	69677000	3000	1	5.58E-06	-1.6254346	7	0.233		
DHR3:74575001	3	74575001	74577000	2000	1	1.86E-06	-1.5573916	8	0.4	Olr532;Olr533;AABR07052768.1	Receptor
DHR3:76341001	3	76341001	76342000	1000	1	6.37E-06	-1.3779149	4	0.4	Olr613	
DHR3:87481001	3	87481001	87482000	1000	1	1.93E-06	-1.5415199	5	0.5		
DHR3:87932001	3	87932001	87935000	3000	1	1.72E-06	-1.5125882	6	0.2		
DHR3:100372001	3	100372001	100373000	1000	1	6.42E-06	-1.0019998	5	0.5	Mettl15;Kif18a	Cytoskeleton
DHR3:101170001	3	101170001	101173000	3000	1	8.41E-06	-1.1106948	10	0.333	Ccdc34	Transcription
DHR3:104702001	3	104702001	104703000	1000	1	1.76E-06	-1.4630831	3	0.3		
DHR3:109240001	3	109240001	109242000	2000	1	3.60E-06	-1.3747067	7	0.35		
DHR3:110495001	3	110495001	110497000	2000	1	9.64E-06	1.0180991	15	0.75	Pak6;Ankrd63;Plcb2	Signaling;Metabolism
DHR3:115109001	3	115109001	115110000	1000	1	4.06E-07	-1.3358886	3	0.3		
DHR3:120740001	3	120740001	120741000	1000	1	5.87E-06	1.1016143	29	2.9	Bcl2l11	Apoptosis
DHR3:124639001	3	124639001	124640000	1000	1	3.62E-06	0.873528	10	1	Slc23a2	Transport

DHR3:132186001	3	132186001	132190000	4000	1	3.03E-06	-1.2878155	26	0.65		
DHR3:133827001	3	133827001	133828000	1000	1	4.91E-06	-1.161826	1	0.1		
DHR3:135088001	3	135088001	135090000	2000	1	3.31E-07	-1.3320411	6	0.3		
DHR3:135529001	3	135529001	135531000	2000	1	1.62E-06	-1.3459493	11	0.55		
DHR3:144751001	3	144751001	144753000	2000	1	1.61E-07	-1.294795	8	0.4		
DHR3:146015001	3	146015001	146017000	2000	1	2.25E-06	-1.086493	6	0.3		
DHR3:147268001	3	147268001	147271000	3000	1	7.71E-06	0.9080874	35	1.167	Psmf1	
DHR3:152524001	3	152524001	152525000	1000	1	9.86E-06	0.8972203	7	0.7		
DHR3:153111001	3	153111001	153112000	1000	1	5.55E-06	0.9443833	15	1.5	Dsn1	
DHR3:156572001	3	156572001	156575000	3000	1	9.02E-07	0.9147701	52	1.733	AABR07054490.1	
DHR3:156773001	3	156773001	156779000	6000	1	4.33E-06	0.9881411	93	1.55	Zhx3	Transcription
DHR3:159577001	3	159577001	159581000	4000	1	4.57E-06	1.1702284	79	1.975	Tox2	
DHR3:163075001	3	163075001	163076000	1000	1	1.24E-06	0.9424812	14	1.4		
DHR3:173919001	3	173919001	173920000	1000	1	2.21E-07	-1.418358	1	0.1	Sycp2	
DHR4:14606001	4	14606001	14607000	1000	1	1.41E-06	-1.4942703	7	0.7		
DHR4:15183001	4	15183001	15184000	1000	1	5.70E-06	-1.2446375	4	0.4		
DHR4:15259001	4	15259001	15260000	1000	1	4.41E-06	-1.5187926	5	0.5		
DHR4:15355001	4	15355001	15356000	1000	1	9.01E-06	-1.2627758	4	0.4		
DHR4:17292001	4	17292001	17293000	1000	1	6.30E-06	-1.2718209	1	0.1	Sema3e	Development
DHR4:18086001	4	18086001	18087000	1000	1	7.64E-06	-1.5717795	3	0.3		
DHR4:24676001	4	24676001	24678000	2000	1	2.20E-08	-1.6095039	5	0.25		
DHR4:32596001	4	32596001	32597000	1000	1	6.39E-06	-1.2397285	4	0.4		
DHR4:34115001	4	34115001	34116000	1000	1	2.63E-06	-1.4700461	6	0.6	Col28a1	
DHR4:37136001	4	37136001	37137000	1000	1	2.13E-06	-1.4245131	1	0.1		
DHR4:48445001	4	48445001	48446000	1000	1	3.25E-06	-1.2096672	10	1		
DHR4:53712001	4	53712001	53715000	3000	1	8.46E-06	-1.1586998	18	0.6		
DHR4:57968001	4	57968001	57971000	3000	1	1.57E-07	0.9797417	53	1.767	Cpa1;Cep41	Protease
DHR4:63714001	4	63714001	63716000	2000	1	5.47E-06	-1.4186829	5	0.25		
DHR4:66091001	4	66091001	66092000	1000	1	3.76E-06	0.8174661	8	0.8	Ttc26;AABR07060287.1	
DHR4:71477001	4	71477001	71478000	1000	1	2.56E-06	-0.9973202	7	0.7		
DHR4:73880001	4	73880001	73882000	2000	1	1.78E-06	0.9932371	58	2.9		
DHR4:86483001	4	86483001	86485000	2000	1	1.18E-06	-1.3799428	8	0.4	Pde1c	Metabolism
DHR4:88026001	4	88026001	88028000	2000	1	5.40E-06	-1.3649268	5	0.25	Vom1r80	
DHR4:88321001	4	88321001	88326000	5000	1	4.43E-07	-1.7320479	20	0.4	Vom1r87	Receptor
DHR4:95977001	4	95977001	95978000	1000	1	3.57E-06	-1.0945255	2	0.2	Hpgds	Metabolism
DHR4:105364001	4	105364001	105366000	2000	1	3.65E-07	-1.4210526	10	0.5		
DHR4:106642001	4	106642001	106644000	2000	1	8.47E-06	-1.2347334	11	0.55		
DHR4:121224001	4	121224001	121226000	2000	1	7.98E-06	1.0938707	34	1.7	Plxna1	Receptor
DHR4:124453001	4	124453001	124454000	1000	1	6.01E-06	0.9516077	8	0.8		
DHR4:127574001	4	127574001	127575000	1000	1	7.41E-06	0.7907236	14	1.4	Suclg2	Metabolism
DHR4:137204001	4	137204001	137206000	2000	1	2.31E-06	-1.5850353	6	0.3		
DHR4:146344001	4	146344001	146345000	1000	1	7.88E-07	0.8799713	11	1.1		
DHR4:153885001	4	153885001	153886000	1000	1	5.55E-06	0.9599572	18	1.8	Slc6a13	Transport
DHR4:156050001	4	156050001	156052000	2000	1	3.40E-06	-0.8635978	17	0.85	Clec4a	Receptor
DHR4:156289001	4	156289001	156291000	2000	1	2.39E-07	-1.334785	8	0.4		
DHR4:159138001	4	159138001	159140000	2000	1	5.33E-06	0.8743747	35	1.75		
DHR4:161257001	4	161257001	161258000	1000	1	3.23E-07	-1.3030481	4	0.4		
DHR4:167126001	4	167126001	167127000	1000	1	2.08E-06	-1.2007739	7	0.7	Tas2r110	
DHR4:170485001	4	170485001	170486000	1000	1	8.93E-06	-1.174559	0	0	AABR07062350.1	
DHR4:170526001	4	170526001	170528000	2000	1	1.10E-06	-1.3280065	9	0.45	Atf7ip	
DHR4:173388001	4	173388001	173391000	3000	1	9.82E-06	0.9183811	34	1.133		
DHR5:15861001	5	15861001	15864000	3000	1	7.06E-07	-1.207469	8	0.267	Xkr4	Unknown
DHR5:16391001	5	16391001	16393000	2000	1	6.81E-06	-1.2127757	19	0.95	Tmem68	Unknown
DHR5:20462001	5	20462001	20463000	1000	1	6.21E-06	-1.5043242	4	0.4		
DHR5:31636001	5	31636001	31638000	2000	1	4.42E-06	-1.2515139	14	0.7	Mmp16	Proteolysis
DHR5:37032001	5	37032001	37034000	2000	1	3.30E-06	-1.168237	4	0.2		
DHR5:38445001	5	38445001	38447000	2000	1	6.10E-07	-1.6678498	3	0.15		
DHR5:41868001	5	41868001	41869000	1000	1	2.65E-06	-1.4138449	10	1		
DHR5:53532001	5	53532001	53534000	2000	1	4.31E-06	-1.3336246	7	0.35		
DHR5:62113001	5	62113001	62114000	1000	1	4.48E-06	0.9092883	26	2.6	Nans	Metabolism
DHR5:67155001	5	67155001	67156000	1000	1	7.01E-06	-1.6695476	4	0.4		
DHR5:79925001	5	79925001	79926000	1000	1	9.96E-06	-0.8725637	2	0.2	AC229945.2	
DHR5:82887001	5	82887001	82888000	1000	1	2.52E-06	-2.0099793	2	0.2		
DHR5:85441001	5	85441001	85443000	2000	1	2.15E-06	-1.2228764	16	0.8		
DHR5:93516001	5	93516001	93517000	1000	1	6.00E-07	-1.3655353	2	0.2		
DHR5:94152001	5	94152001	94154000	2000	1	6.93E-06	-1.2387906	13	0.65		
DHR5:97342001	5	97342001	97344000	2000	1	5.07E-06	-1.285579	7	0.35		
DHR5:97480001	5	97480001	97481000	1000	1	7.06E-06	-1.3198459	3	0.3		
DHR5:98415001	5	98415001	98416000	1000	1	9.33E-06	-1.2495885	6	0.6	Tyrp1	Metabolism

DHR5:107363001	5	107363001	107365000	2000	1	3.76E-06	-0.8081941	14	0.7	AABR07049134.3;LOC100912314	Immune
DHR5:113816001	5	113816001	113817000	1000	1	8.77E-06	-0.9503538	10	1	Tek;AABR07049286.1	Signaling
DHR5:135977001	5	135977001	135978000	1000	1	1.11E-06	1.2393865	33	3.3	Ptch2;Btbd19	
DHR5:139616001	5	139616001	139617000	1000	1	1.25E-06	1.1664641	28	2.8		
DHR5:142000001	5	142000001	142002000	2000	1	4.93E-06	0.9373544	32	1.6		
DHR5:155231001	5	155231001	155235000	4000	1	4.27E-06	0.888901	89	2.225		
DHR5:158808001	5	158808001	158811000	3000	1	4.13E-06	0.9686698	80	2.667		
DHR5:159900001	5	159900001	159901000	1000	1	8.92E-06	1.0710416	23	2.3		
DHR5:162739001	5	162739001	162740000	1000	1	1.88E-06	-1.0645756	2	0.2	RGD1559644	
DHR5:170125001	5	170125001	170126000	1000	1	1.10E-08	1.0961473	25	2.5		
DHR5:172026001	5	172026001	172028000	2000	1	8.46E-06	1.1137274	32	1.6		
DHR6:2767001	6	2767001	2768000	1000	1	1.91E-06	1.1029902	20	2		
DHR6:4597001	6	4597001	4599000	2000	1	3.28E-06	-1.1425368	10	0.5		
DHR6:4979001	6	4979001	4981000	2000	1	9.20E-09	-1.4809202	7	0.35		
DHR6:7993001	6	7993001	7994000	1000	1	3.05E-06	0.926207	22	2.2	Lrpprc	Development
DHR6:9755001	6	9755001	9756000	1000	1	7.00E-06	0.9201645	17	1.7		
DHR6:11392001	6	11392001	11395000	3000	1	6.94E-07	1.1039657	57	1.9	Kcnk12	Transport
DHR6:20816001	6	20816001	20817000	1000	1	4.63E-06	-1.1871034	2	0.2		
DHR6:29216001	6	29216001	29218000	2000	1	1.02E-06	0.9500702	25	1.25	Klhl29	Transcription
DHR6:38447001	6	38447001	38449000	2000	1	7.35E-06	-1.2766377	9	0.45	Ddx1	Transcription
DHR6:40005001	6	40005001	40008000	3000	1	1.91E-06	-1.5202297	14	0.467		
DHR6:48714001	6	48714001	48715000	1000	1	2.13E-06	0.8891124	21	2.1	Myt1l	Transcription
DHR6:56874001	6	56874001	56875000	1000	1	1.50E-06	-1.2450602	6	0.6	Agmo	Metabolism
DHR6:59484001	6	59484001	59487000	3000	1	3.88E-07	-1.2446313	9	0.3		
DHR6:63364001	6	63364001	63366000	2000	1	3.45E-06	-1.5071319	6	0.3		
DHR6:65071001	6	65071001	65072000	1000	1	5.52E-06	-1.2499216	2	0.2		
DHR6:68065001	6	68065001	68068000	3000	1	2.01E-06	-1.3510918	6	0.2		
DHR6:71468001	6	71468001	71470000	2000	1	1.61E-06	-1.0795293	4	0.2		
DHR6:90162001	6	90162001	90165000	3000	1	5.94E-07	-1.3866614	20	0.667		
DHR6:100345001	6	100345001	100346000	1000	1	7.23E-08	-1.4049174	6	0.6	Fut8	Metabolism
DHR6:104538001	6	104538001	104539000	1000	1	6.62E-06	0.9225193	11	1.1	Susd6	
DHR6:118285001	6	118285001	118286000	1000	1	1.01E-06	-1.645431	4	0.4		
DHR6:120978001	6	120978001	120980000	2000	1	3.38E-08	-1.676635	19	0.95		
DHR6:124347001	6	124347001	124349000	2000	1	8.70E-06	1.0558961	23	1.15	Ttc7b	Metabolism
DHR6:130724001	6	130724001	130726000	2000	1	4.08E-06	0.9754726	25	1.25		
DHR7:12270001	7	12270001	12272000	2000	1	2.35E-06	1.0623791	62	3.1	Apc2	Cytoskeleton
DHR7:12397001	7	12397001	12401000	4000	1	4.83E-06	0.873983	123	3.075	Efna2;RGD1562114;Cirbp	Signaling;Transcription
DHR7:13080001	7	13080001	13082000	2000	1	7.49E-06	1.0573686	30	1.5	Plpp2	
DHR7:17778001	7	17778001	17780000	2000	1	3.16E-07	-1.4536079	8	0.4		
DHR7:18113001	7	18113001	18114000	1000	1	9.03E-07	-0.9674317	6	0.6	Vom1r108	
DHR7:19687001	7	19687001	19688000	1000	1	1.39E-06	-1.6283413	1	0.1		
DHR7:21470001	7	21470001	21472000	2000	1	1.97E-06	-1.5232866	10	0.5		
DHR7:27856001	7	27856001	27857000	1000	1	8.09E-06	-1.1388568	5	0.5		
DHR7:36226001	7	36226001	36227000	1000	1	8.69E-06	0.8919028	20	2		
DHR7:43459001	7	43459001	43460000	1000	1	2.78E-07	-2.1734312	1	0.1		
DHR7:52971001	7	52971001	52972000	1000	1	3.18E-06	0.9862529	14	1.4		
DHR7:53085001	7	53085001	53086000	1000	1	6.26E-06	-1.0910849	8	0.8		
DHR7:55960001	7	55960001	55963000	3000	1	8.53E-08	-1.8084681	16	0.533		
DHR7:62342001	7	62342001	62343000	1000	1	7.30E-06	-1.0893968	4	0.4	AABR07057237.2	
DHR7:67847001	7	67847001	67848000	1000	1	2.88E-06	-1.0153564	1	0.1		
DHR7:71180001	7	71180001	71181000	1000	1	3.23E-06	0.8892696	10	1	AABR07057436.1;Sdr9c7	Metabolism
DHR7:76260001	7	76260001	76261000	1000	1	2.76E-06	0.9121376	15	1.5	Ncald	Signaling
DHR7:87780001	7	87780001	87781000	1000	1	8.86E-07	-1.1650355	7	0.7	AABR07057765.1	
DHR7:97046001	7	97046001	97047000	1000	1	5.64E-06	-1.1728706	5	0.5	Slc22a22	
DHR7:107716001	7	107716001	107717000	1000	1	9.21E-06	0.9971403	16	1.6	Ccn4	
DHR7:111676001	7	111676001	111679000	3000	1	1.83E-07	-1.4470555	8	0.267	AABR07058360.3	
DHR7:112628001	7	112628001	112629000	1000	1	5.54E-06	-1.2908773	6	0.6		
DHR7:115530001	7	115530001	115532000	2000	1	4.85E-06	0.8956183	18	0.9		
DHR7:118424001	7	118424001	118425000	1000	1	2.78E-06	1.0136008	20	2		
DHR7:119475001	7	119475001	119477000	2000	1	6.15E-06	0.8515441	30	1.5	Ncf4	Development
DHR7:122811001	7	122811001	122813000	2000	1	8.53E-06	1.0733081	29	1.45	Gm23880;AABR07058539.1	
DHR7:135477001	7	135477001	135480000	3000	1	9.13E-07	1.072002	51	1.7	Adamts20	Protease
DHR7:137802001	7	137802001	137803000	1000	1	2.09E-06	0.8827294	16	1.6	Arid2;AABR07058795.1;Scaf11	
DHR7:142472001	7	142472001	142476000	4000	1	9.05E-07	1.1053447	73	1.825	Slc4a8	Transport
DHR7:145381001	7	145381001	145382000	1000	1	1.51E-06	1.2141797	18	1.8	AABR07058955.1;AABR07058955.2	
DHR8:1981001	8	1981001	1983000	2000	1	1.98E-06	-1.3486421	4	0.2	Gria4	Signaling
DHR8:2264001	8	2264001	2265000	1000	1	7.69E-06	-1.3279399	0	0		
DHR8:2753001	8	2753001	2755000	2000	1	9.35E-06	-1.4171475	3	0.15		
DHR8:3625001	8	3625001	3628000	3000	1	6.47E-06	-1.2266618	8	0.267		

DHR8:3641001	8	3641001	3642000	1000	1	4.49E-07	-1.4286283	1	0.1	Vom1r24	
DHR8:4193001	8	4193001	4194000	1000	1	3.09E-06	-1.5222035	4	0.4		
DHR8:5058001	8	5058001	5060000	2000	1	2.93E-06	-1.0610318	11	0.55		
DHR8:11104001	8	11104001	11107000	3000	1	1.18E-07	-1.8200042	23	0.767		
DHR8:15902001	8	15902001	15904000	2000	1	4.31E-06	-1.3676315	8	0.4		
DHR8:16937001	8	16937001	16939000	2000	1	4.56E-06	-1.2195571	7	0.35		
DHR8:19434001	8	19434001	19435000	1000	1	4.06E-07	-1.3157988	7	0.7		
DHR8:25576001	8	25576001	25577000	1000	1	3.21E-06	-1.1714675	6	0.6	Dpy19l1	Unknown
DHR8:28559001	8	28559001	28561000	2000	1	4.27E-06	-1.2569489	16	0.8		
DHR8:34409001	8	34409001	34410000	1000	1	4.10E-07	0.9505369	20	2		
DHR8:37818001	8	37818001	37819000	1000	1	3.02E-06	-1.1135347	1	0.1		
DHR8:39706001	8	39706001	39709000	3000	1	9.67E-06	0.931665	46	1.533	Tmem218;Slc37a2	Metabolism
DHR8:44441001	8	44441001	44442000	1000	1	7.44E-06	1.0217067	11	1.1		
DHR8:44956001	8	44956001	44958000	2000	1	6.25E-07	0.9778571	43	2.15	Clmp	Receptor
DHR8:58540001	8	58540001	58542000	2000	1	8.35E-08	0.9211207	31	1.55	Elmod1	Signaling
DHR8:61464001	8	61464001	61466000	2000	1	3.73E-08	1.107157	27	1.35		
DHR8:61528001	8	61528001	61529000	1000	1	1.26E-06	1.1758572	12	1.2	Odf3l1;Cspg4	Cytoskeleton
DHR8:61682001	8	61682001	61684000	2000	1	2.59E-06	-1.3232783	16	0.8	Ptpn9	Signaling
DHR8:73282001	8	73282001	73283000	1000	1	7.17E-06	1.0884246	16	1.6		
DHR8:76429001	8	76429001	76432000	3000	1	6.48E-06	-1.2065422	24	0.8	Bnip2;LOC103695118	
DHR8:77978001	8	77978001	77979000	1000	1	3.46E-06	-0.9343226	4	0.4	AC132740.1;Polr2m	
DHR8:83942001	8	83942001	83944000	2000	1	6.34E-06	-1.2453229	11	0.55		
DHR8:97913001	8	97913001	97914000	1000	1	1.93E-07	-1.3797465	5	0.5		
DHR8:98307001	8	98307001	98308000	1000	1	9.79E-07	-1.5810798	0	0		
DHR8:116459001	8	116459001	116464000	5000	1	6.98E-08	1.141495	69	1.38	Sema3f	Growth Factors & Cytokines
DHR8:116760001	8	116760001	116763000	3000	1	7.43E-06	1.1110377	49	1.633	Traip;Inka1;AC128059.3;Uba7;AC128059.5	Signaling;Metabolism
DHR8:127552001	8	127552001	127556000	4000	1	5.19E-06	1.0540764	51	1.275	Itga9	Extracellular Matrix
DHR8:128739001	8	128739001	128743000	4000	1	8.82E-06	1.0809658	101	2.525	Cx3cr1;AABR07071742.3	Growth Factors & Cytokines
DHR8:129049001	8	129049001	129051000	2000	1	8.38E-06	1.0536607	40	2	Myrip	Metabolism
DHR9:34227001	9	34227001	34229000	2000	1	2.01E-06	-1.2111541	19	0.95		
DHR9:35553001	9	35553001	35556000	3000	1	5.31E-06	-1.1280068	20	0.667		
DHR9:40592001	9	40592001	40594000	2000	1	2.60E-06	-1.1681839	4	0.2		
DHR9:51385001	9	51385001	51386000	1000	1	4.49E-06	-1.1979433	8	0.8	Gulp1	Development
DHR9:52285001	9	52285001	52286000	1000	1	2.64E-06	-1.1773243	0	0		
DHR9:68487001	9	68487001	68490000	3000	1	3.32E-06	-1.5447784	12	0.4	Pard3b	Cell Junction
DHR9:76303001	9	76303001	76305000	2000	1	7.48E-06	-1.1697284	11	0.55		
DHR9:79096001	9	79096001	79097000	1000	1	1.85E-07	-1.4686089	4	0.4		
DHR9:85957001	9	85957001	85958000	1000	1	4.15E-06	1.0306726	4	0.4		
DHR9:102526001	9	102526001	102528000	2000	1	9.76E-06	-1.2706717	3	0.15		
DHR9:104069001	9	104069001	104072000	3000	1	8.49E-06	-1.4239741	16	0.533		
DHR9:114699001	9	114699001	114700000	1000	1	1.46E-06	0.9261265	16	1.6	Rab12	Signaling
DHR9:117148001	9	117148001	117149000	1000	1	8.13E-06	0.8530135	13	1.3		
DHR9:120955001	9	120955001	120957000	2000	1	6.00E-06	-1.1404585	12	0.6		
DHR10:3774001	10	3774001	3778000	4000	1	2.67E-06	0.8363746	55	1.375	Cpped1	Signaling
DHR10:13747001	10	13747001	13750000	3000	1	9.74E-06	1.0520673	52	1.733	Abca3	Receptor
DHR10:16119001	10	16119001	16120000	1000	1	2.78E-08	1.0493789	10	1		
DHR10:18903001	10	18903001	18905000	2000	1	4.61E-07	1.320565	24	1.2	Kcnip1;Kcnmb1	Signaling;Metabolism
DHR10:23813001	10	23813001	23815000	2000	1	6.13E-07	0.9743229	32	1.6	Ebf1	Transcription
DHR10:38994001	10	38994001	38995000	1000	1	3.33E-06	0.9526139	15	1.5	Il13;Rad50	Signaling;Transcription
DHR10:44145001	10	44145001	44147000	2000	1	6.51E-06	-1.0739713	10	0.5	Olr1424;AC097901.1;Olr1425	Receptor
DHR10:70613001	10	70613001	70614000	1000	1	4.79E-06	0.8817755	12	1.2	Ap2b1	Transport
DHR10:74245001	10	74245001	74250000	5000	1	6.57E-06	1.06488	85	1.7	Ypel2	
DHR10:95098001	10	95098001	95099000	1000	1	5.21E-06	-1.0655352	7	0.7		
DHR10:96277001	10	96277001	96279000	2000	1	1.48E-06	0.9399712	49	2.45	Prkca	Binding Protein
DHR10:96750001	10	96750001	96752000	2000	1	3.70E-06	-0.7093502	15	0.75	Cep112	
DHR10:97728001	10	97728001	97729000	1000	1	5.28E-06	1.1024783	20	2	Amz2;Slc16a6	
DHR10:103221001	10	103221001	103223000	2000	1	6.41E-06	0.8737851	36	1.8	Ttyh2	Transport
DHR10:103248001	10	103248001	103249000	1000	1	6.92E-06	1.1009296	22	2.2	Ttyh2	Transport
DHR10:105591001	10	105591001	105597000	6000	1	5.84E-06	0.9834115	132	2.2	Rhbdf2	Protease
DHR11:1859001	11	1859001	1862000	3000	1	3.44E-06	-1.2698788	15	0.5		
DHR11:3165001	11	3165001	3167000	2000	1	2.41E-06	-1.0602401	16	0.8	Vgll3	Unknown
DHR11:4768001	11	4768001	4771000	3000	1	5.30E-07	-1.3594324	10	0.333		
DHR11:7834001	11	7834001	7835000	1000	1	4.32E-06	-1.4921393	5	0.5		
DHR11:8586001	11	8586001	8587000	1000	1	3.42E-06	-1.3950885	4	0.4		
DHR11:11376001	11	11376001	11377000	1000	1	6.61E-06	-1.1774264	7	0.7	Robo2	Receptor
DHR11:12131001	11	12131001	12132000	1000	1	9.05E-06	-1.0990746	2	0.2		
DHR11:13130001	11	13130001	13132000	2000	1	1.45E-06	-1.1535201	25	1.25		

DHR11:21851001	11	21851001	21853000	2000	1	9.26E-06	-1.3657341	11	0.55		
DHR11:21926001	11	21926001	21927000	1000	1	7.28E-06	-1.1817822	2	0.2		
DHR11:23349001	11	23349001	23353000	4000	1	2.54E-06	-1.6608749	11	0.275		
DHR11:25953001	11	25953001	25954000	1000	1	9.53E-06	-1.298161	1	0.1		
DHR11:27518001	11	27518001	27519000	1000	1	3.76E-06	1.0698225	12	1.2		
DHR11:35103001	11	35103001	35108000	5000	1	6.97E-06	0.946887	58	1.16	Kcnj6	Metabolism
DHR11:36281001	11	36281001	36282000	1000	1	8.93E-06	0.8529387	16	1.6		
DHR11:39881001	11	39881001	39882000	1000	1	5.74E-06	-1.2866405	4	0.4		
DHR11:43182001	11	43182001	43185000	3000	1	9.74E-06	-1.3016165	11	0.367	Olr1531;Olr1532	
DHR11:48266001	11	48266001	48267000	1000	1	5.06E-06	-1.2612983	3	0.3		
DHR11:51991001	11	51991001	51994000	3000	1	3.43E-07	-1.3555853	8	0.267		
DHR11:57614001	11	57614001	57620000	6000	1	9.53E-06	-1.2765388	30	0.5		
DHR11:63856001	11	63856001	63859000	3000	1	7.59E-06	-1.0671245	19	0.633		
DHR11:65408001	11	65408001	65409000	1000	1	8.46E-06	-1.2197159	6	0.6		
DHR11:69453001	11	69453001	69454000	1000	1	1.21E-06	0.8411612	12	1.2		
DHR11:70085001	11	70085001	70088000	3000	1	8.21E-07	1.0067433	71	2.367	Itgb5	Receptor
DHR11:73342001	11	73342001	73345000	3000	1	2.41E-06	0.9930107	43	1.433		
DHR11:73795001	11	73795001	73796000	1000	1	9.34E-06	1.3840446	30	3		
DHR11:75769001	11	75769001	75770000	1000	1	4.28E-06	-1.3308983	2	0.2		
DHR11:78666001	11	78666001	78667000	1000	1	8.32E-07	1.0927562	3	0.3		
DHR11:85166001	11	85166001	85168000	2000	1	7.39E-07	-1.26914	5	0.25	Olr1565	
DHR11:86940001	11	86940001	86944000	4000	1	9.78E-06	1.0216835	64	1.6	AABR07072264.4	
DHR12:1478001	12	1478001	1480000	2000	1	3.77E-06	-1.3781175	11	0.55	Rfc3	Transcription
DHR12:2282001	12	2282001	2283000	1000	1	7.54E-07	0.9485395	11	1.1	Clec4g	Receptor
DHR12:9754001	12	9754001	9756000	2000	1	1.60E-06	0.9587395	31	1.55	Lnx2	Cytoskeleton
DHR12:13573001	12	13573001	13574000	1000	1	2.90E-06	0.9127797	8	0.8	Rnf216	Metabolism
DHR12:18767001	12	18767001	18768000	1000	1	4.96E-06	-1.2056476	2	0.2		
DHR12:19371001	12	19371001	19373000	2000	1	3.25E-06	-1.3843127	5	0.25		
DHR12:22457001	12	22457001	22459000	2000	1	9.60E-07	0.9827786	95	4.75	Slc12a9;Trip6;AABR07035787.1	Transport;Cytoskeleton
DHR12:29500001	12	29500001	29502000	2000	1	6.69E-06	0.9110661	23	1.15	Caln1	Signaling
DHR12:40992001	12	40992001	40994000	2000	1	4.11E-06	0.8717805	15	0.75		
DHR12:42501001	12	42501001	42504000	3000	1	4.69E-06	0.8855786	95	3.167	Tbx3	Epigenetic
DHR13:2520001	13	2520001	2521000	1000	1	2.02E-06	-1.3797211	1	0.1		
DHR13:7906001	13	7906001	7911000	5000	1	2.27E-06	-1.7511888	19	0.38		
DHR13:10093001	13	10093001	10094000	1000	1	5.15E-06	-1.2951725	2	0.2		
DHR13:12223001	13	12223001	12226000	3000	1	1.95E-06	-1.331543	21	0.7		
DHR13:12612001	13	12612001	12613000	1000	1	7.87E-06	-0.9102025	7	0.7		
DHR13:19812001	13	19812001	19815000	3000	1	5.96E-06	-1.6686157	9	0.3		
DHR13:20850001	13	20850001	20853000	3000	1	2.88E-07	-1.6772633	6	0.2		
DHR13:21001001	13	21001001	21003000	2000	1	7.81E-06	-1.0797438	4	0.2		
DHR13:22730001	13	22730001	22731000	1000	1	2.23E-08	-1.8714531	3	0.3		
DHR13:29950001	13	29950001	29951000	1000	1	8.34E-07	-1.1938719	2	0.2		
DHR13:31217001	13	31217001	31218000	1000	1	6.99E-06	-1.4923663	3	0.3	Cdh7	Extracellular Matrix
DHR13:32306001	13	32306001	32307000	1000	1	7.98E-06	-1.0710778	3	0.3		
DHR13:34914001	13	34914001	34918000	4000	1	7.64E-06	1.1093931	44	1.1	Gli2	Transcription
DHR13:35017001	13	35017001	35021000	4000	1	2.12E-06	0.9350764	57	1.425	Gli2	Transcription
DHR13:43143001	13	43143001	43145000	2000	1	9.90E-06	-1.4818426	13	0.65		
DHR13:44873001	13	44873001	44876000	3000	1	6.84E-07	-1.4186253	12	0.4	R3hdm1	Unknown
DHR13:47686001	13	47686001	47687000	1000	1	5.49E-06	-1.2089384	5	0.5		
DHR13:56256001	13	56256001	56258000	2000	1	9.57E-06	-1.0824012	13	0.65	AABR07021086.1	
DHR13:57358001	13	57358001	57359000	1000	1	6.18E-06	-1.2551028	8	0.8	Kcnt2	Transport
DHR13:58288001	13	58288001	58289000	1000	1	9.55E-06	-1.4240193	2	0.2		
DHR13:59249001	13	59249001	59251000	2000	1	7.08E-06	-1.2867996	3	0.15		
DHR13:63648001	13	63648001	63650000	2000	1	8.77E-06	-1.4769727	9	0.45	Brinp3	
DHR13:65108001	13	65108001	65110000	2000	1	5.12E-08	-1.5210534	14	0.7		
DHR13:66005001	13	66005001	66007000	2000	1	1.05E-06	-1.5845514	11	0.55		
DHR13:73285001	13	73285001	73286000	1000	1	1.24E-06	0.9241502	7	0.7	Acbd6	Metabolism
DHR13:79755001	13	79755001	79757000	2000	1	2.83E-06	-1.4054128	10	0.5	Suco	
DHR13:100061001	13	100061001	100063000	2000	1	5.30E-06	1.1165036	25	1.25	AABR07021946.1;AABR07021946.2	
DHR13:111314001	13	111314001	111316000	2000	1	6.53E-06	0.9723461	31	1.55	Hhat	Metabolism
DHR13:111933001	13	111933001	111935000	2000	1	8.63E-06	1.0121967	17	0.85	Hsd11b1	Metabolism
DHR13:113779001	13	113779001	113781000	2000	1	9.81E-07	1.1527244	8	0.4	Mir29b2;Mir3556b;Cd46	Immune
DHR14:4424001	14	4424001	4425000	1000	1	2.90E-07	0.958347	17	1.7		
DHR14:22591001	14	22591001	22592000	1000	1	1.71E-06	-1.4736995	2	0.2	Ugt2a3;Ugt2b35	Metabolism
DHR14:24574001	14	24574001	24576000	2000	1	5.51E-07	-1.0986763	8	0.4		
DHR14:25472001	14	25472001	25473000	1000	1	7.88E-06	-1.5045223	2	0.2		
DHR14:26691001	14	26691001	26693000	2000	1	1.36E-06	-1.3513566	11	0.55	Tecrl	
DHR14:26851001	14	26851001	26852000	1000	1	9.52E-06	-1.4436006	6	0.6		
DHR14:27644001	14	27644001	27646000	2000	1	8.77E-06	-1.8126304	7	0.35		

DHR14:29348001	14	29348001	29350000	2000	1	7.28E-06	-1.2694122	8	0.4		
DHR14:31023001	14	31023001	31024000	1000	1	2.39E-07	-1.3872805	3	0.3	AABR07014769.1	
DHR14:32964001	14	32964001	32966000	2000	1	8.03E-06	0.8081918	25	1.25		
DHR14:39392001	14	39392001	39394000	2000	1	1.61E-06	0.8765383	61	3.05		
DHR14:47178001	14	47178001	47180000	2000	1	4.79E-06	-1.291933	15	0.75		
DHR14:48619001	14	48619001	48620000	1000	1	4.77E-06	-0.6450885	14	1.4	Dthd1	
DHR14:49049001	14	49049001	49053000	4000	1	6.56E-06	-1.2304093	8	0.2		
DHR14:52045001	14	52045001	52049000	4000	1	5.71E-06	-1.3792903	11	0.275		
DHR14:52375001	14	52375001	52376000	1000	1	1.97E-06	-1.7502305	2	0.2		
DHR14:54599001	14	54599001	54605000	6000	1	7.20E-07	-1.5670502	38	0.633		
DHR14:56584001	14	56584001	56586000	2000	1	6.28E-06	-1.2028879	8	0.4		
DHR14:57463001	14	57463001	57466000	3000	1	6.17E-06	-1.3248159	8	0.267		
DHR14:57637001	14	57637001	57638000	1000	1	1.90E-06	-1.1496588	2	0.2		
DHR14:63669001	14	63669001	63672000	3000	1	3.30E-07	-1.2972214	8	0.267		
DHR14:64578001	14	64578001	64579000	1000	1	2.51E-06	0.8466982	6	0.6		
DHR14:75447001	14	75447001	75450000	3000	1	1.25E-06	-1.545532	18	0.6		
DHR14:80862001	14	80862001	80864000	2000	1	9.32E-06	0.8842388	27	1.35		
DHR14:82915001	14	82915001	82917000	2000	1	4.08E-07	1.0091332	43	2.15	Slc5a1	Transport
DHR14:85392001	14	85392001	85394000	2000	1	1.54E-06	1.1746355	62	3.1	Emid1	Extracellular Matrix
DHR14:88949001	14	88949001	88952000	3000	1	9.04E-06	0.9337025	54	1.8		
DHR14:90249001	14	90249001	90250000	1000	1	3.39E-06	-1.3797414	2	0.2		
DHR14:94036001	14	94036001	94038000	2000	1	5.25E-06	-1.5711101	8	0.4		
DHR14:96938001	14	96938001	96939000	1000	1	1.99E-06	-0.8540288	2	0.2		
DHR14:112286001	14	112286001	112289000	3000	1	2.05E-07	-1.47181	14	0.467	RNaseP_nuc	
DHR15:7193001	15	7193001	7194000	1000	1	2.90E-07	-1.5539227	6	0.6		
DHR15:7373001	15	7373001	7375000	2000	1	6.46E-06	-1.1059301	7	0.35		
DHR15:24955001	15	24955001	24957000	2000	1	9.00E-06	0.8506991	44	2.2	Peli2	Signaling
DHR15:30204001	15	30204001	30209000	5000	1	3.84E-06	-1.6711343	21	0.42	AABR07017701.1	
DHR15:38415001	15	38415001	38416000	1000	1	3.71E-06	0.9527257	13	1.3		
DHR15:39838001	15	39838001	39839000	1000	1	1.66E-07	0.9781521	13	1.3	Cab39l	Signaling
DHR15:46243001	15	46243001	46246000	3000	1	2.74E-06	0.9841786	27	0.9	Defb44	
DHR15:64065001	15	64065001	64066000	1000	1	3.50E-06	-1.3559519	2	0.2		
DHR15:65111001	15	65111001	65113000	2000	1	2.17E-06	-1.2305104	9	0.45		
DHR15:65552001	15	65552001	65556000	4000	1	9.80E-07	-1.3903227	20	0.5		
DHR15:65911001	15	65911001	65913000	2000	1	1.90E-06	-1.403175	12	0.6		
DHR15:66212001	15	66212001	66214000	2000	1	1.54E-06	-1.5512106	6	0.3		
DHR15:78297001	15	78297001	78298000	1000	1	4.06E-08	-1.7186372	4	0.4		
DHR15:79362001	15	79362001	79363000	1000	1	2.01E-06	-1.5307378	7	0.7		
DHR15:84287001	15	84287001	84288000	1000	1	9.53E-06	-1.0644938	2	0.2		
DHR15:91474001	15	91474001	91475000	1000	1	1.90E-06	-1.3982919	3	0.3	Mycbp2;AABR07019155.1	Metabolism
DHR15:94310001	15	94310001	94312000	2000	1	6.41E-06	-1.4070592	13	0.65		
DHR15:94984001	15	94984001	94986000	2000	1	9.89E-06	-1.372557	6	0.3		
DHR15:96237001	15	96237001	96239000	2000	1	2.19E-09	-1.6712154	11	0.55		
DHR15:98231001	15	98231001	98233000	2000	1	3.36E-07	-1.6757572	7	0.35		
DHR15:107067001	15	107067001	107069000	2000	1	8.26E-06	-1.2807314	13	0.65		
DHR16:131001	16	131001	133000	2000	1	2.54E-09	-1.2544404	17	0.85	AABR07024442.1	
DHR16:1508001	16	1508001	1511000	3000	1	5.80E-06	0.8266503	49	1.633		
DHR16:7130001	16	7130001	7131000	1000	1	8.77E-07	-1.2203231	10	1	Pbrm1	Unknown
DHR16:12967001	16	12967001	12971000	4000	1	1.78E-06	-1.0816536	24	0.6	AABR07024732.1;AABR07024733.1	
DHR16:17132001	16	17132001	17133000	1000	1	5.56E-06	-1.2146039	3	0.3	AABR07024799.1	
DHR16:27299001	16	27299001	27300000	1000	1	2.40E-07	-1.3403277	2	0.2		
DHR16:41030001	16	41030001	41031000	1000	1	7.96E-07	-1.6532517	10	1	AABR07025594.1	
DHR16:43766001	16	43766001	43767000	1000	1	7.83E-07	-1.3633368	5	0.5		
DHR16:44605001	16	44605001	44607000	2000	1	6.52E-06	-1.3529863	5	0.25		
DHR16:52546001	16	52546001	52550000	4000	1	3.62E-08	-1.658517	24	0.6	U6	
DHR16:53415001	16	53415001	53417000	2000	1	4.35E-07	-1.5835313	7	0.35		
DHR16:57180001	16	57180001	57182000	2000	1	6.84E-06	-1.8719072	25	1.25		
DHR16:61298001	16	61298001	61299000	1000	1	1.80E-06	1.0854358	7	0.7		
DHR16:72053001	16	72053001	72055000	2000	1	1.41E-07	-0.8018306	22	1.1	Adam5	
DHR16:74280001	16	74280001	74281000	1000	1	2.79E-06	0.8338042	27	2.7		
DHR16:74871001	16	74871001	74873000	2000	1	9.08E-06	0.9061835	19	0.95	Alg11;Atp7b	Metabolism;Transport
DHR16:81268001	16	81268001	81269000	1000	1	9.57E-06	-1.2896122	4	0.4	AABR07026534.3	
DHR16:83414001	16	83414001	83415000	1000	1	9.13E-06	1.3607709	31	3.1	Col4a2	Cytoskeleton
DHR17:28911001	17	28911001	28912000	1000	1	6.43E-06	-0.8596984	7	0.7		
DHR17:29273001	17	29273001	29274000	1000	1	3.04E-06	-1.1358825	6	0.6	Fars2	
DHR17:34716001	17	34716001	34717000	1000	1	4.88E-06	1.2128401	6	0.6	Exoc2;AABR07027502.1	
DHR17:35823001	17	35823001	35824000	1000	1	9.88E-06	0.9444305	13	1.3		
DHR17:36613001	17	36613001	36614000	1000	1	4.30E-06	0.9901922	12	1.2	AABR07027567.1	
DHR17:40369001	17	40369001	40370000	1000	1	7.31E-06	-0.993551	5	0.5		

DHR17:44881001	17	44881001	44884000	3000	1	9.63E-06	-1.3681324	3	0.1		
DHR17:48814001	17	48814001	48815000	1000	1	6.19E-07	-1.3693727	7	0.7	Vps41	Transport
DHR17:49124001	17	49124001	49125000	1000	1	2.74E-06	-1.1054878	4	0.4		
DHR17:50888001	17	50888001	50890000	2000	1	9.61E-06	-1.1863904	8	0.4		
DHR17:64730001	17	64730001	64732000	2000	1	3.41E-06	-1.4303614	10	0.5		
DHR17:69672001	17	69672001	69674000	2000	1	2.67E-06	-1.3707456	9	0.45	Akr1c19	Metabolism
DHR17:70431001	17	70431001	70432000	1000	1	1.42E-06	0.9975988	13	1.3	Fbh1	
DHR17:71730001	17	71730001	71731000	1000	1	5.28E-06	1.0894335	21	2.1	Sfmbt2	Transcription
DHR18:1286001	18	1286001	1290000	4000	1	3.39E-06	-1.4337112	13	0.325	Rock1	Signaling
DHR18:2710001	18	2710001	2712000	2000	1	4.13E-07	1.1115527	32	1.6		
DHR18:34447001	18	34447001	34449000	2000	1	6.93E-06	-1.3169565	4	0.2		
DHR18:39097001	18	39097001	39098000	1000	1	4.47E-06	-1.2548324	1	0.1		
DHR18:53079001	18	53079001	53081000	2000	1	9.03E-06	-1.0545386	38	1.9	Fbn2;AC104053.2	Extracellular Matrix
DHR18:70342001	18	70342001	70345000	3000	1	1.12E-06	1.0342285	50	1.667		
DHR18:79735001	18	79735001	79738000	3000	1	2.20E-06	1.0033043	60	2		
DHR18:84897001	18	84897001	84898000	1000	1	4.72E-06	-1.4729389	1	0.1		
DHR19:7079001	19	7079001	7081000	2000	1	1.73E-07	-1.5043608	8	0.4		
DHR19:9587001	19	9587001	9589000	2000	1	9.75E-06	0.9967526	99	4.95	Got2	Metabolism
DHR19:11568001	19	11568001	11569000	1000	1	3.81E-06	0.973311	10	1	Gnao1	Signaling
DHR19:27537001	19	27537001	27540000	3000	1	6.99E-06	-1.072661	15	0.5	Olr1666	Receptor
DHR19:37109001	19	37109001	37111000	2000	1	1.36E-06	0.9437954	31	1.55	Ces4a;U6	
DHR19:50122001	19	50122001	50126000	4000	1	7.54E-06	0.8122062	57	1.425	Plcg2	Signaling
DHR19:53081001	19	53081001	53084000	3000	1	4.47E-06	0.8885451	33	1.1		
DHR19:54196001	19	54196001	54197000	1000	1	8.63E-06	1.1943494	18	1.8	RGD1309651	
DHR19:55354001	19	55354001	55355000	1000	1	6.75E-06	0.8562963	16	1.6	Piezo1	
DHR19:57403001	19	57403001	57404000	1000	1	4.62E-06	0.9609608	16	1.6	RGD1559896	EST
DHR19:58214001	19	58214001	58216000	2000	1	4.06E-06	1.0545314	34	1.7		
DHR19:58524001	19	58524001	58525000	1000	1	2.67E-07	1.0326376	17	1.7		
DHR19:60645001	19	60645001	60647000	2000	1	3.28E-06	0.8242639	27	1.35		
DHR20:3288001	20	3288001	3291000	3000	1	8.12E-06	0.9292302	41	1.367	RT1-T24-1;Gnl1;Prr3	Immune;Transcription
DHR20:4986001	20	4986001	4987000	1000	1	9.29E-06	0.9453185	22	2.2	AABR07044408.1;AC094348.4;Vars	Metabolism
DHR20:6185001	20	6185001	6188000	3000	1	9.88E-06	0.9949027	60	2	AABR07044444.2;Pxt1	
DHR20:24894001	20	24894001	24896000	2000	1	8.20E-07	-1.3772935	13	0.65		
DHR20:26056001	20	26056001	26057000	1000	1	4.32E-06	-1.4434983	9	0.9	Lrrtm3	Receptor
DHR20:29728001	20	29728001	29731000	3000	1	1.88E-06	1.0959688	38	1.267	Chst3	Metabolism
DHR20:30157001	20	30157001	30160000	3000	1	7.94E-06	1.098396	54	1.8	AABR07044980.1	
DHR20:36885001	20	36885001	36887000	2000	1	8.72E-06	-1.1624751	12	0.6		
DHR20:37257001	20	37257001	37259000	2000	1	5.75E-06	-1.4031875	2	0.1		
DHR20:38832001	20	38832001	38837000	5000	1	5.49E-07	-1.3738942	31	0.62		
DHR20:42182001	20	42182001	42183000	1000	1	1.53E-06	-1.4339413	5	0.5		
DHR20:42943001	20	42943001	42944000	1000	1	3.53E-06	-1.0960825	5	0.5		
DHR20:53813001	20	53813001	53815000	2000	1	6.04E-06	-1.4613786	10	0.5	Grik2	Signaling
DHR20:53994001	20	53994001	53995000	1000	1	2.78E-06	-1.5229499	3	0.3	Grik2	Signaling
DHR20:55909001	20	55909001	55911000	2000	1	3.91E-06	-1.6302831	8	0.4		
DHRX:6852001	X	6852001	6855000	3000	1	6.07E-08	-0.8809166	29	0.967	AABR07073516.1	
DHRX:15190001	X	15190001	15191000	1000	1	8.86E-07	1.3273143	6	0.6	Suv39h1l1	
DHRX:16604001	X	16604001	16605000	1000	1	6.37E-06	-1.5706523	8	0.8		
DHRX:59070001	X	59070001	59071000	1000	1	5.28E-08	-1.8791795	6	0.6		
DHRX:63816001	X	63816001	63817000	1000	1	2.74E-06	-1.8626656	11	1.1	Maged1;AABR07038902.1;AABR07038902.2	Extracellular Matrix
DHRX:92709001	X	92709001	92710000	1000	1	7.61E-06	-1.5913318	8	0.8		
DHRX:103131001	X	103131001	103132000	1000	1	3.46E-07	-2.167639	3	0.3		
DHRX:106356001	X	106356001	106358000	2000	1	2.60E-06	1.5867972	47	2.35	Gprasp2	
DHRX:125863001	X	125863001	125864000	1000	1	4.65E-06	-1.9180939	5	0.5		
DHRX:127279001	X	127279001	127282000	3000	1	9.40E-06	-1.5807267	14	0.467		
DHRX:132474001	X	132474001	132475000	1000	1	2.46E-06	-1.9711229	2	0.2		
DHRX:152609001	X	152609001	152610000	1000	1	4.42E-06	-1.7690737	3	0.3	Gabra3	Receptor
