# Supplementary material for: Epigenome-wide association study for atrazine induced transgenerational DNA methylation and histone retention sperm epigenetic biomarkers for disease
Source: PLoS One. 2020 Dec 16;15(12):e0239380. doi: 10.1371/journal.pone.0239380 (PMC7743986; doi:10.1371/journal.pone.0239380)
Supplement: S8 Table — DHR name, chromosome, start, stop, length, number signature windows, minimum p-value, max log-fold change, CpG number, CpG density, gene annotation, and gene category are presented. (PDF) [file pone.0239380.s015.pdf]

Supplemental Table S8
DHR Site List Kidney Disease p<1e-04

DHR Name	Chr	Start	Stop	Length	# Sig Win	minP	maxLFC	CpG #	CpG Density	Gene Annotation	Gene Category
DHR1:15223001	1	15223001	15224000	1000	1	3.97E-05	-0.9694469	18	1.8	Il20ra	
DHR1:34264001	1	34264001	34265000	1000	1	8.74E-06	-1.1601736	10	1	AABR07001025.1	
DHR1:65050001	1	65050001	65051000	1000	1	1.44E-05	1.2735624	0	0	Vom2r80;AABR07002025.1	Receptor
DHR1:76584001	1	76584001	76595000	11000	1	6.00E-05	0.8502241	37	0.336	Sult2a6	Metabolism
DHR1:76698001	1	76698001	76707000	9000	1	1.74E-05	1.1612308	79	0.878	Sult2a6	Metabolism
DHR1:76953001	1	76953001	76961000	8000	1	3.52E-05	1.4550449	48	0.6		
DHR1:76999001	1	76999001	77001000	2000	1	4.27E-05	1.1005052	6	0.3		
DHR1:77048001	1	77048001	77049000	1000	1	2.79E-05	1.4796164	4	0.4		
DHR1:77205001	1	77205001	77210000	5000	1	9.35E-06	0.9225666	16	0.32		
DHR1:77326001	1	77326001	77329000	3000	1	3.71E-05	0.9240578	18	0.6		
DHR1:79604001	1	79604001	79605000	1000	1	9.38E-08	0.8590981	5	0.5		
DHR1:93880001	1	93880001	93881000	1000	1	5.03E-05	-0.9024491	21	2.1	Zfp536	Transcription
DHR1:94496001	1	94496001	94497000	1000	1	1.00E-04	0.8922243	12	1.2	Ccne1	Cell Cycle
DHR1:94749001	1	94749001	94750000	1000	1	6.37E-05	0.6871612	33	3.3	AC120712.1	
DHR1:113350001	1	113350001	113352000	2000	1	3.45E-05	-6.5181459	11	0.55	Luzp2;AABR07003504.1	
DHR1:124499001	1	124499001	124500000	1000	1	3.99E-05	-0.9028138	5	0.5		
DHR1:125670001	1	125670001	125671000	1000	1	3.63E-06	-1.2435304	17	1.7	Fam189a1	Unknown
DHR1:129118001	1	129118001	129119000	1000	1	6.81E-05	-1.1186355	14	1.4	Igf1r	Growth Factors & Cytokines
DHR1:138121001	1	138121001	138122000	1000	1	1.87E-07	-1.38049	4	0.4	Agbl1	Signaling
DHR1:153001001	1	153001001	153002000	1000	1	6.35E-05	0.8828783	7	0.7	Tmem135	Unknown
DHR1:161151001	1	161151001	161152000	1000	1	7.43E-05	-1.1222656	6	0.6		
DHR1:162360001	1	162360001	162361000	1000	1	2.61E-05	-1.5376435	17	1.7	Alg8;Ndufc2	Metabolism
DHR1:183627001	1	183627001	183628000	1000	1	4.60E-05	0.8103758	9	0.9	AABR07005506.1	
DHR1:200201001	1	200201001	200202000	1000	1	9.84E-05	-0.9662524	13	1.3	Sec23ip	Golgi
DHR1:211361001	1	211361001	211362000	1000	1	7.70E-05	-1.1456095	12	1.2	Jakmip3	
DHR1:212151001	1	212151001	212152000	1000	1	5.74E-05	0.9329483	7	0.7	AABR07005985.3	
DHR1:248645001	1	248645001	248646000	1000	1	3.12E-05	-1.4541656	13	1.3	LOC103690131	
DHR1:253252001	1	253252001	253253000	1000	1	5.86E-05	0.964272	6	0.6	Kif20b	Cytoskeleton
DHR2:51613001	2	51613001	51614000	1000	1	4.53E-05	0.8316954	9	0.9	AABR07008287.1	
DHR2:53573001	2	53573001	53574000	1000	1	5.17E-05	0.9099667	12	1.2		
DHR2:58356001	2	58356001	58357000	1000	1	6.56E-05	-0.5309087	12	1.2		
DHR2:81405001	2	81405001	81406000	1000	1	9.44E-05	-1.108241	8	0.8		
DHR2:81745001	2	81745001	81746000	1000	1	7.01E-05	0.7996121	9	0.9		
DHR2:97639001	2	97639001	97640000	1000	1	2.97E-05	0.8316949	4	0.4		
DHR2:105833001	2	105833001	105834000	1000	1	9.81E-05	0.8150319	3	0.3		
DHR2:163292001	2	163292001	163293000	1000	1	3.67E-06	-1.0368352	6	0.6		
DHR2:188182001	2	188182001	188183000	1000	1	2.83E-05	0.8488544	19	1.9	Gon4l	Transcription
DHR2:196416001	2	196416001	196419000	3000	1	7.55E-05	-1.0929574	45	1.5	Cdc42se1;RGD1359334;Bnipl;Prune1	
DHR2:198778001	2	198778001	198779000	1000	1	7.31E-05	-1.1017397	36	3.6	Pex11b;U2;Itga10	Extracellular Matrix
DHR2:202717001	2	202717001	202718000	1000	1	7.05E-05	-0.9544574	15	1.5		
DHR2:231465001	2	231465001	231466000	1000	1	1.42E-05	1.0233969	10	1	Ank2	Cytoskeleton
DHR2:253095001	2	253095001	253096000	1000	1	2.98E-05	-0.978899	6	0.6		
DHR2:254920001	2	254920001	254921000	1000	1	7.16E-06	0.828603	5	0.5		
DHR2:256738001	2	256738001	256739000	1000	1	8.03E-05	-1.1350917	14	1.4		
DHR2:262944001	2	262944001	262945000	1000	1	9.04E-05	-0.8407525	6	0.6	Negr1	Growth Factors & Cytokines
DHR3:27928001	3	27928001	27930000	2000	1	8.50E-05	-2.2237159	9	0.45		
DHR3:36264001	3	36264001	36265000	1000	1	6.50E-05	-1.0919194	10	1		
DHR3:43947001	3	43947001	43949000	2000	1	6.86E-05	-1.1452973	19	0.95		
DHR3:52221001	3	52221001	52222000	1000	1	4.96E-05	-1.0689051	13	1.3	Galnt3	Metabolism
DHR3:59577001	3	59577001	59580000	3000	1	6.15E-05	0.942383	29	0.967	AC120066.1	
DHR3:91538001	3	91538001	91539000	1000	1	9.19E-05	-1.7443327	10	1	Ldlrad3	Receptor
DHR3:139219001	3	139219001	139220000	1000	1	7.41E-05	-0.9853297	3	0.3		
DHR3:151016001	3	151016001	151017000	1000	1	6.81E-06	-1.2260167	19	1.9	Ggt7	Metabolism
DHR3:156623001	3	156623001	156624000	1000	1	3.75E-05	-1.3470753	7	0.7	AABR07054490.1;AABR07072945.1	
DHR3:172060001	3	172060001	172061000	1000	1	7.04E-05	0.7626667	17	1.7		
DHR4:7370001	4	7370001	7371000	1000	1	8.89E-05	-1.1677262	17	1.7	Kcnh2	
DHR4:22025001	4	22025001	22027000	2000	1	9.89E-05	-1.044303	10	0.5		
DHR4:28549001	4	28549001	28551000	2000	1	7.56E-06	1.0266018	15	0.75	Vps50	
DHR4:42706001	4	42706001	42707000	1000	1	8.36E-05	-1.1194185	5	0.5	Cftr	Transport
DHR4:50853001	4	50853001	50854000	1000	1	8.43E-05	0.7526469	16	1.6	Cadps2	Metabolism
DHR4:81453001	4	81453001	81454000	1000	1	5.34E-05	-0.9776987	8	0.8		
DHR4:85438001	4	85438001	85440000	2000	1	7.33E-05	-1.3974882	31	1.55	Mindy4	
DHR4:139917001	4	139917001	139918000	1000	1	8.35E-05	-1.5545446	9	0.9		

DHR4:167282001	4	167282001	167283000	1000	1	1.06E-05	0.9637367	2	0.2		
DHR4:169310001	4	169310001	169312000	2000	1	4.18E-05	-1.0677402	11	0.55		
DHR4:173722001	4	173722001	173723000	1000	1	7.46E-05	0.8328339	12	1.2	Pik3c2g	Signaling
DHR4:175766001	4	175766001	175767000	1000	1	3.20E-05	1.0117341	5	0.5	Slco1c1	Transport
DHR4:178495001	4	178495001	178496000	1000	1	1.96E-05	-1.0559599	12	1.2		
DHR5:2431001	5	2431001	2432000	1000	1	8.94E-05	-0.9533457	9	0.9		
DHR5:6831001	5	6831001	6832000	1000	1	5.78E-05	-1.0371918	4	0.4		
DHR5:16598001	5	16598001	16599000	1000	1	7.75E-05	-1.2805373	15	1.5	Lyn	Transcription
DHR5:23989001	5	23989001	23991000	2000	1	2.68E-05	0.7736813	17	0.85		
DHR5:27254001	5	27254001	27255000	1000	1	2.84E-05	0.934357	10	1		
DHR5:43961001	5	43961001	43962000	1000	1	9.76E-05	-1.0090983	31	3.1		
DHR5:45555001	5	45555001	45557000	2000	1	2.48E-05	1.2055848	10	0.5	AABR07047744.2;AABR07047744.1	
DHR5:58556001	5	58556001	58557000	1000	1	2.15E-06	-1.2563492	5	0.5	Unc13b	Receptor
DHR5:73863001	5	73863001	73864000	1000	1	1.16E-05	-1.334223	2	0.2		
DHR5:75657001	5	75657001	75658000	1000	1	1.03E-05	-1.4749988	24	2.4	Lpar1	Receptor
DHR5:113160001	5	113160001	113162000	2000	1	6.02E-05	0.715639	8	0.4		
DHR5:116127001	5	116127001	116129000	2000	1	1.19E-05	0.8501151	6	0.3		
DHR5:121819001	5	121819001	121820000	1000	1	5.88E-05	-0.8113004	6	0.6		
DHR5:126271001	5	126271001	126272000	1000	1	5.26E-05	-1.0380005	22	2.2	Ttc4	
DHR5:152507001	5	152507001	152509000	2000	1	2.85E-05	-1.5867324	68	3.4	Pdik1l	Signaling
DHR5:155539001	5	155539001	155540000	1000	1	7.24E-05	-1.046391	24	2.4		
DHR5:161157001	5	161157001	161158000	1000	1	6.80E-05	-0.9229866	17	1.7		
DHR6:36840001	6	36840001	36841000	1000	1	7.53E-05	-0.9236753	13	1.3	AABR07063601.2	
DHR6:63724001	6	63724001	63726000	2000	1	8.28E-05	-0.94474	7	0.35		
DHR6:75530001	6	75530001	75531000	1000	1	8.63E-05	-0.8704732	15	1.5	Sptssa	
DHR6:88214001	6	88214001	88216000	2000	1	5.81E-05	0.6780697	8	0.4		
DHR6:94050001	6	94050001	94051000	1000	1	5.73E-05	-1.4047013	7	0.7		
DHR6:98968001	6	98968001	98970000	2000	1	4.76E-05	-1.012212	16	0.8	AABR07064873.1	
DHR6:102156001	6	102156001	102157000	1000	1	8.96E-05	-0.9915222	17	1.7	Tmem229b	Unknown
DHR6:108246001	6	108246001	108247000	1000	1	7.32E-05	-1.0285568	16	1.6		
DHR6:139299001	6	139299001	139300000	1000	1	8.69E-05	1.0898648	3	0.3	AABR07065684.1	
DHR6:145045001	6	145045001	145046000	1000	1	8.96E-05	-0.9286688	13	1.3	Ptprn2	Signaling
DHR7:5417001	7	5417001	5418000	1000	1	7.47E-05	0.9613414	2	0.2	Olr920	
DHR7:12951001	7	12951001	12952000	1000	1	6.27E-05	-1.0348778	8	0.8	Odf3l2;Shc2	Unknown;Signaling
DHR7:14921001	7	14921001	14922000	1000	1	4.90E-05	-1.1836841	13	1.3		
DHR7:34880001	7	34880001	34881000	1000	1	7.00E-05	-0.8609342	11	1.1	Mir331;Vezt	
DHR7:38916001	7	38916001	38917000	1000	1	9.70E-05	-1.27288	11	1.1	Epyc	Receptor
DHR7:74019001	7	74019001	74020000	1000	1	4.07E-05	0.6990207	14	1.4		
DHR7:83768001	7	83768001	83769000	1000	1	6.81E-05	-0.9505179	6	0.6		
DHR7:94290001	7	94290001	94291000	1000	1	2.38E-05	-0.9761852	1	0.1		
DHR7:110099001	7	110099001	110100000	1000	1	7.78E-05	0.7602525	8	0.8	Khdrbs3	Transcription
DHR7:114779001	7	114779001	114780000	1000	1	4.86E-05	-1.388892	13	1.3	Dennd3	Signaling
DHR7:121791001	7	121791001	121792000	1000	1	7.46E-05	-1.1375772	30	3	Enthd1	Transport
DHR7:141792001	7	141792001	141793000	1000	1	9.17E-11	-1.6618994	15	1.5	Dip2b	Development
DHR7:142951001	7	142951001	142952000	1000	1	5.07E-05	-0.8860301	23	2.3	AC119007.1;AC119007.4	
DHR8:25423001	8	25423001	25425000	2000	1	3.20E-05	0.9505794	6	0.3	Npsr1	Receptor
DHR8:39397001	8	39397001	39399000	2000	1	1.28E-05	0.9609867	24	1.2	Pknox2	Transcription
DHR8:55089001	8	55089001	55092000	3000	1	1.24E-05	0.9712763	29	0.967	Dlat;Dixdc1	Metabolism;Signaling
DHR8:55565001	8	55565001	55566000	1000	1	1.12E-05	0.8133414	9	0.9		
DHR8:75060001	8	75060001	75061000	1000	1	5.83E-05	1.2382627	15	1.5		
DHR8:83188001	8	83188001	83189000	1000	1	4.72E-05	-1.2990559	13	1.3	Hmgcll1	Metabolism
DHR8:94288001	8	94288001	94289000	1000	1	9.38E-05	-1.1396339	13	1.3	Me1	Metabolism
DHR8:118747001	8	118747001	118748000	1000	1	8.20E-05	0.7142699	6	0.6	Kif9	Cytoskeleton
DHR8:119373001	8	119373001	119374000	1000	1	5.58E-05	-1.1066062	19	1.9	AABR07073453.1;Lrrfip2	Transcription
DHR8:120051001	8	120051001	120052000	1000	1	9.81E-05	-0.8150037	8	0.8		
DHR8:128367001	8	128367001	128368000	1000	1	5.35E-05	-0.8559377	31	3.1	Scn10a	Transport
DHR8:130947001	8	130947001	130948000	1000	1	1.96E-05	-1.0965034	11	1.1	AABR07071779.2	
DHR9:4514001	9	4514001	4515000	1000	1	1.20E-05	-1.6356088	7	0.7		
DHR9:13101001	9	13101001	13102000	1000	1	1.90E-05	-1.0847028	24	2.4	Kif6	Cytoskeleton
DHR9:37624001	9	37624001	37627000	3000	1	9.86E-05	-1.4919287	16	0.533		
DHR9:38858001	9	38858001	38859000	1000	1	7.73E-05	-0.9276263	8	0.8		
DHR9:51309001	9	51309001	51310000	1000	1	6.94E-05	-1.3066386	13	1.3	Gulp1	Development
DHR9:58681001	9	58681001	58682000	1000	1	5.31E-05	-0.9262081	5	0.5		
DHR9:61927001	9	61927001	61928000	1000	1	2.86E-05	-1.5387531	2	0.2	Boll	Development
DHR9:85039001	9	85039001	85040000	1000	1	6.47E-05	0.9757443	12	1.2		
DHR9:90254001	9	90254001	90255000	1000	1	3.54E-05	-1.0849721	12	1.2		
DHR9:105206001	9	105206001	105207000	1000	1	4.92E-05	0.7801044	17	1.7		
DHR10:343001	10	343001	344000	1000	1	1.95E-07	0.3829771	10	1		

DHR10:18429001	10	18429001	18430000	1000	1	2.09E-06	1.0990421	10	1	Ranbp17	Binding Protein
DHR10:19536001	10	19536001	19537000	1000	1	8.15E-05	-0.9828027	15	1.5	AABR07029272.1	
DHR10:49721001	10	49721001	49722000	1000	1	5.71E-05	0.9768791	5	0.5		
DHR10:63446001	10	63446001	63447000	1000	1	4.20E-05	-1.1492337	11	1.1	Gosr1	Transport
DHR10:85582001	10	85582001	85583000	1000	1	9.90E-05	-1.2844539	20	2	Epop	
DHR10:88792001	10	88792001	88793000	1000	1	7.64E-05	-1.3803399	22	2.2	Stat5a;Stat3	Transcription
DHR11:8549001	11	8549001	8551000	2000	1	5.61E-05	0.9200934	2	0.1		
DHR11:16590001	11	16590001	16591000	1000	1	4.60E-05	-1.0422999	15	1.5		
DHR11:32125001	11	32125001	32126000	1000	1	2.56E-05	-0.9765434	9	0.9		
DHR11:44730001	11	44730001	44731000	1000	1	4.38E-05	0.9475482	7	0.7	AABR07033925.1	
DHR11:49458001	11	49458001	49459000	1000	1	6.23E-05	0.8486265	8	0.8		
DHR11:76194001	11	76194001	76195000	1000	1	6.55E-05	-1.0310076	8	0.8		
DHR11:81818001	11	81818001	81819000	1000	1	8.48E-05	-0.9526061	20	2		
DHR12:14052001	12	14052001	14053000	1000	1	4.63E-05	-1.1273722	19	1.9	Mmd2	Development
DHR12:45565001	12	45565001	45566000	1000	1	3.69E-05	-1.1112514	12	1.2		
DHR12:45758001	12	45758001	45759000	1000	1	1.80E-05	-1.1851684	7	0.7	Srrm4	Translation
DHR13:7680001	13	7680001	7681000	1000	1	2.55E-05	0.5572764	11	1.1		
DHR13:9041001	13	9041001	9043000	2000	1	9.32E-05	-1.1399511	8	0.4		
DHR13:37214001	13	37214001	37215000	1000	1	2.99E-05	-1.2399957	2	0.2		
DHR13:39779001	13	39779001	39780000	1000	1	2.42E-06	0.8875321	2	0.2		
DHR13:41028001	13	41028001	41029000	1000	1	3.67E-05	-0.955187	6	0.6		
DHR13:49936001	13	49936001	49937000	1000	1	2.11E-05	-1.1304301	9	0.9	Ppp1r15b	Signaling
DHR13:72960001	13	72960001	72961000	1000	1	2.44E-05	-1.1057383	8	0.8	Xpr1	Receptor
DHR13:79937001	13	79937001	79938000	1000	1	4.20E-06	0.8784284	15	1.5	Dnm3;AC144674.2	Cytoskeleton
DHR13:80458001	13	80458001	80459000	1000	1	3.13E-05	-1.0757115	9	0.9	AABR07021596.3;Vamp4	Transport
DHR13:83362001	13	83362001	83363000	1000	1	4.27E-05	-1.300356	13	1.3		
DHR13:85239001	13	85239001	85240000	1000	1	5.23E-05	-1.3032375	9	0.9	Fam78b	
DHR13:101343001	13	101343001	101345000	2000	1	6.78E-05	-1.0881854	26	1.3		
DHR13:106893001	13	106893001	106894000	1000	1	8.42E-06	-1.3657073	19	1.9	Ush2a	Extracellular Matrix
DHR14:741001	14	741001	742000	1000	1	2.25E-05	1.1247994	2	0.2		
DHR14:55016001	14	55016001	55017000	1000	1	3.52E-05	-1.1874986	17	1.7		
DHR14:64636001	14	64636001	64638000	2000	1	6.78E-05	0.9618548	19	0.95		
DHR14:69142001	14	69142001	69143000	1000	1	7.11E-05	0.8530223	3	0.3		
DHR14:93200001	14	93200001	93201000	1000	1	8.79E-06	-0.9959549	8	0.8		
DHR14:108732001	14	108732001	108733000	1000	1	1.22E-05	-1.5063798	21	2.1		
DHR15:5046001	15	5046001	5048000	2000	1	3.03E-05	-1.0532098	13	0.65	AABR07016919.1	
DHR15:26912001	15	26912001	26913000	1000	1	1.02E-05	-0.9309377	8	0.8		
DHR15:26936001	15	26936001	26938000	2000	1	6.18E-05	-1.2290865	6	0.3	Olr1630	
DHR15:53029001	15	53029001	53030000	1000	1	7.41E-05	-1.022485	8	0.8		
DHR15:56578001	15	56578001	56579000	1000	1	4.70E-05	0.9499761	3	0.3		
DHR15:59707001	15	59707001	59708000	1000	1	9.52E-05	-0.9380675	18	1.8	Enox1	Transcription
DHR15:77725001	15	77725001	77726000	1000	1	5.30E-05	-1.1371831	9	0.9	Pcdh9	Extracellular Matrix
DHR15:84671001	15	84671001	84672000	1000	1	6.06E-06	-1.2766525	12	1.2	Klf12	Transcription
DHR15:90976001	15	90976001	90977000	1000	1	8.90E-05	-1.0244222	13	1.3	Mycbp2	Metabolism
DHR15:93810001	15	93810001	93811000	1000	1	5.93E-05	-1.235751	17	1.7	Mycbp2	Metabolism
DHR15:109547001	15	109547001	109548000	1000	1	9.63E-05	0.8479137	3	0.3		
DHR16:199001	16	199001	201000	2000	1	2.53E-05	-1.1058459	11	0.55		
DHR16:25101001	16	25101001	25102000	1000	1	2.04E-08	-1.3779033	1	0.1	1-Mar	Metabolism
DHR16:26261001	16	26261001	26263000	2000	1	8.12E-06	-1.3508939	10	0.5		
DHR16:31858001	16	31858001	31859000	1000	1	3.58E-05	-1.0274823	8	0.8	AABR07025295.1	
DHR16:38183001	16	38183001	38184000	1000	1	1.19E-07	-2.2523299	6	0.6		
DHR16:62754001	16	62754001	62755000	1000	1	4.06E-05	-1.3362861	4	0.4		
DHR16:78715001	16	78715001	78716000	1000	1	4.27E-06	-1.1362284	16	1.6	Csmd1	Signaling
DHR16:83235001	16	83235001	83236000	1000	1	9.31E-05	-1.0281187	15	1.5	Ankrd10	Transcription
DHR17:26425001	17	26425001	26426000	1000	1	8.31E-05	-1.0542235	9	0.9		
DHR17:27804001	17	27804001	27805000	1000	1	4.53E-05	-1.0461723	15	1.5		
DHR17:37576001	17	37576001	37577000	1000	1	6.07E-05	-1.0405946	6	0.6		
DHR17:58100001	17	58100001	58101000	1000	1	5.43E-05	0.8220433	13	1.3	Adarb2	Metabolism
DHR18:60383001	18	60383001	60384000	1000	1	2.92E-05	-1.2206528	20	2	Nedd4l	Protease
DHR18:62378001	18	62378001	62381000	3000	1	3.73E-05	-2.2865111	28	0.933		
DHR18:84641001	18	84641001	84642000	1000	1	2.81E-05	0.769187	3	0.3		
DHR19:3010001	19	3010001	3012000	2000	1	2.24E-05	-0.9432514	16	0.8		
DHR19:27721001	19	27721001	27723000	2000	1	8.27E-05	-1.0024956	9	0.45		
DHR19:28180001	19	28180001	28181000	1000	1	5.60E-05	0.7487744	6	0.6		
DHR20:7213001	20	7213001	7215000	2000	1	2.40E-06	-1.2650194	28	1.4	Nudt3;Rps10	Transcription;Translation
DHR20:7469001	20	7469001	7470000	1000	1	5.00E-05	-1.4214207	33	3.3	Uhrf1bp1;Taf11	Translation
DHR20:11188001	20	11188001	11189000	1000	1	6.67E-05	-0.951284	12	1.2	Trappc10	Golgi
DHR20:16010001	20	16010001	16011000	1000	1	9.62E-07	0.9300227	12	1.2		
DHR20:27952001	20	27952001	27955000	3000	1	5.62E-05	-0.9510282	33	1.1	Lims1	Immune

DHR20:28759001	20	28759001	28760000	1000	1	2.75E-05	0.895926	12	1.2	10-Sep	Cytoskeleton
DHR20:31440001	20	31440001	31442000	2000	1	4.93E-05	0.9852599	38	1.9		
DHR20:34917001	20	34917001	34918000	1000	1	7.01E-05	-1.0466689	10	1	Mcm9;Asf1a	Transcription
DHR20:38673001	20	38673001	38675000	2000	1	3.02E-05	-1.1601936	12	0.6		
DHR20:45650001	20	45650001	45652000	2000	1	7.57E-05	-1.3488495	41	2.05		
DHRX:53101001	X	53101001	53102000	1000	1	8.61E-05	1.1075008	6	0.6	Dmd	Development
DHRX:57899001	X	57899001	57900000	1000	1	1.34E-06	-1.8194543	25	2.5		
DHRX:89956001	X	89956001	89957000	1000	1	4.39E-05	0.883436	7	0.7		
DHRX:105071001	X	105071001	105072000	1000	1	2.27E-05	-1.836248	12	1.2		
DHRX:116864001	X	116864001	116865000	1000	1	2.75E-05	1.2661213	10	1		
DHRX:119833001	X	119833001	119834000	1000	1	6.31E-05	1.2292699	2	0.2		
DHRX:126416001	X	126416001	126417000	1000	1	4.74E-05	1.1270243	3	0.3		
DHRX:134631001	X	134631001	134632000	1000	1	6.19E-05	1.1593753	9	0.9		
DHRX:136828001	X	136828001	136829000	1000	1	8.00E-05	1.0762025	6	0.6		
DHRX:149483001	X	149483001	149484000	1000	1	2.65E-05	1.082224	8	0.8		
DHRX:152441001	X	152441001	152442000	1000	1	9.51E-05	1.1499996	9	0.9	Gabra3	Receptor
