# Supplementary material for: Epigenome-wide association study for atrazine induced transgenerational DNA methylation and histone retention sperm epigenetic biomarkers for disease
Source: PLoS One. 2020 Dec 16;15(12):e0239380. doi: 10.1371/journal.pone.0239380 (PMC7743986; doi:10.1371/journal.pone.0239380)
Supplement: S9 Table — DHR name, chromosome, start, stop, length, number signature windows, minimum p-value, max log-fold change, CpG number, CpG density, gene annotation, and gene category are presented. (PDF) [file pone.0239380.s016.pdf]

Supplemental Table S9
DHR Site List Testis Disease p<1e-04

DHR Name	Chr	Start	Stop	Length	# Sig Win	minP	maxLFC	CpG #	CpG Density	Gene Annotation	Gene Category
DHR1:25025001	1	25025001	25029000	4000	1	4.65E-05	0.4659154	14	0.35		
DHR1:28219001	1	28219001	28220000	1000	1	7.62E-05	0.8081254	0	0		
DHR1:33594001	1	33594001	33595000	1000	1	1.79E-05	-1.0178117	10	1		
DHR1:48155001	1	48155001	48156000	1000	1	4.51E-05	0.8163254	7	0.7		
DHR1:50638001	1	50638001	50639000	1000	1	9.09E-06	-1.5626368	6	0.6		
DHR1:51443001	1	51443001	51444000	1000	1	8.98E-06	0.8272049	12	1.2		
DHR1:57104001	1	57104001	57105000	1000	1	4.42E-05	-0.9499754	14	1.4		
DHR1:72600001	1	72600001	72601000	1000	1	2.52E-05	-1.39676	5	0.5		
DHR1:72629001	1	72629001	72630000	1000	1	7.88E-05	0.758901	3	0.3	Rpl28;AABR07071876.1;Tmem190;Il11	Transcription
DHR1:75160001	1	75160001	75162000	2000	1	9.16E-05	1.3938343	20	1	RGD1564801;Vom1r59	Signaling;Receptor
DHR1:76561001	1	76561001	76563000	2000	1	4.77E-05	0.8220296	19	0.95	Sult2a6	Metabolism
DHR1:76582001	1	76582001	76590000	8000	1	8.12E-05	1.0246113	27	0.338	Sult2a6	Metabolism
DHR1:76591001	1	76591001	76596000	5000	1	9.55E-05	0.7569775	20	0.4	Sult2a6	Metabolism
DHR1:76652001	1	76652001	76655000	3000	1	2.24E-05	0.9498529	42	1.4	Sult2a6;AABR07002546.1	Metabolism
DHR1:76952001	1	76952001	76960000	8000	1	8.46E-05	0.9120606	45	0.562		
DHR1:77059001	1	77059001	77062000	3000	1	4.07E-05	1.0659756	11	0.367		
DHR1:77088001	1	77088001	77092000	4000	1	2.36E-05	1.0069066	17	0.425		
DHR1:86953001	1	86953001	86954000	1000	1	1.10E-05	-0.9612898	9	0.9	Nfkbib;Sirt2	Signaling;Transcription
DHR1:88417001	1	88417001	88418000	1000	1	7.44E-05	1.0614638	13	1.3	AABR07002888.2;Zfp566;AABR07002888.1	Transcription
DHR1:90952001	1	90952001	90953000	1000	1	9.79E-05	-1.5792203	18	1.8	AABR07002973.1;Sdhaf1;Syne4;Alkbh6;7SK;Clip3	Metabolism;Cytoskeleton
DHR1:92251001	1	92251001	92254000	3000	2	3.70E-09	-6.6820392	34	1.133		
DHR1:99861001	1	99861001	99862000	1000	1	5.67E-05	-1.3717399	9	0.9	AABR07003241.1;AABR07003241.2	
DHR1:106862001	1	106862001	106863000	1000	1	9.61E-05	0.7397922	6	0.6	AABR07003349.1	
DHR1:123485001	1	123485001	123486000	1000	1	1.63E-05	0.877794	4	0.4	AABR07004061.1	
DHR1:124281001	1	124281001	124282000	1000	1	8.48E-05	-0.8660391	6	0.6	AABR07004090.1	
DHR1:130843001	1	130843001	130845000	2000	1	6.93E-05	-0.9224261	7	0.35		
DHR1:141966001	1	141966001	141967000	1000	1	3.49E-05	-1.0228156	13	1.3	LOC102548889	
DHR1:156378001	1	156378001	156379000	1000	1	9.85E-06	-2.0494193	11	1.1		
DHR1:157960001	1	157960001	157961000	1000	1	6.98E-05	0.6850703	3	0.3		
DHR1:161087001	1	161087001	161088000	1000	1	6.55E-05	-0.8805806	8	0.8		
DHR1:161943001	1	161943001	161945000	2000	1	2.16E-05	1.0538332	41	2.05	Nars2	Transcription
DHR1:171949001	1	171949001	171950000	1000	1	4.22E-05	-0.8418715	4	0.4	Ppfibp2	Receptor
DHR1:176506001	1	176506001	176507000	1000	1	5.23E-05	-1.2579113	21	2.1	Galnt18	Unknown
DHR1:185598001	1	185598001	185599000	1000	1	2.36E-05	0.8281052	6	0.6	AC128610.1	
DHR1:197788001	1	197788001	197789000	1000	1	6.02E-05	0.7175318	9	0.9	Spns1;Nfatc2ip	Transport;Unknown
DHR1:201886001	1	201886001	201887000	1000	1	5.01E-05	0.8616152	9	0.9	AC123083.1;Cuzd1	
DHR1:216302001	1	216302001	216303000	1000	1	6.76E-05	-1.1796989	7	0.7	Kcnq1	Transport
DHR1:217341001	1	217341001	217342000	1000	1	5.34E-05	-0.9829494	8	0.8	Shank2	Protein Binding
DHR1:231793001	1	231793001	231794000	1000	1	5.46E-06	-1.1428613	3	0.3		
DHR1:233754001	1	233754001	233755000	1000	1	5.29E-05	0.8879584	2	0.2		
DHR1:235445001	1	235445001	235446000	1000	1	1.79E-05	0.5214253	11	1.1		
DHR1:243936001	1	243936001	243938000	2000	1	8.13E-08	-1.3422308	13	0.65		
DHR1:248349001	1	248349001	248350000	1000	1	3.82E-05	0.9704893	15	1.5	Gldc	Metabolism
DHR1:249224001	1	249224001	249225000	1000	1	4.28E-05	-0.8667342	8	0.8		
DHR1:249596001	1	249596001	249597000	1000	1	5.70E-05	0.8912301	13	1.3		
DHR1:253895001	1	253895001	253896000	1000	1	2.61E-05	1.1475927	8	0.8		
DHR1:255390001	1	255390001	255392000	2000	1	2.04E-05	-0.9334903	26	1.3		
DHR1:265476001	1	265476001	265477000	1000	1	2.80E-05	-1.5683585	7	0.7		
DHR1:270777001	1	270777001	270778000	1000	1	4.17E-05	0.6779589	5	0.5		
DHR1:271713001	1	271713001	271714000	1000	1	3.49E-05	-1.4073188	9	0.9		
DHR1:275550001	1	275550001	275552000	2000	1	7.79E-05	0.8003211	16	0.8		
DHR2:379001	2	379001	380000	1000	1	9.11E-05	0.6316247	8	0.8		
DHR2:12446001	2	12446001	12447000	1000	1	2.80E-05	-1.2807957	14	1.4		
DHR2:20025001	2	20025001	20026000	1000	1	2.98E-05	-1.0468371	11	1.1	Atg10	
DHR2:27896001	2	27896001	27897000	1000	1	8.26E-05	0.6697529	11	1.1	Fam169a	Unknown
DHR2:28938001	2	28938001	28940000	2000	1	8.90E-05	0.7907607	21	1.05	Fcho2	Cell Cycle
DHR2:36574001	2	36574001	36575000	1000	1	3.24E-05	0.7282819	4	0.4		
DHR2:53773001	2	53773001	53774000	1000	1	6.40E-05	-0.9756262	17	1.7		
DHR2:60338001	2	60338001	60339000	1000	1	9.63E-05	0.8171521	5	0.5	Agxt2	Metabolism
DHR2:78264001	2	78264001	78265000	1000	1	6.94E-05	-1.636027	10	1	Zfp622	
DHR2:90051001	2	90051001	90052000	1000	1	8.17E-05	0.6477013	5	0.5		
DHR2:103788001	2	103788001	103789000	1000	1	6.67E-05	-1.0658878	2	0.2		
DHR2:107590001	2	107590001	107591000	1000	1	5.90E-05	0.7110088	7	0.7		
DHR2:113418001	2	113418001	113419000	1000	1	1.86E-05	-1.0427186	6	0.6		
DHR2:123211001	2	123211001	123212000	1000	1	2.01E-05	-1.0738851	19	1.9		
DHR2:147704001	2	147704001	147706000	2000	1	2.65E-06	-1.0397463	11	0.55		
DHR2:175975001	2	175975001	175976000	1000	1	6.13E-06	-1.0783636	2	0.2		
DHR2:196881001	2	196881001	196882000	1000	1	4.77E-05	-0.9630622	11	1.1		
DHR2:197939001	2	197939001	197941000	2000	1	3.11E-05	-0.9125175	27	1.35	Rprd2;Prpf3	Signaling;Translation
DHR2:203436001	2	203436001	203437000	1000	1	3.27E-05	0.6798696	13	1.3	Ptgfrn	
DHR2:211725001	2	211725001	211726000	1000	1	7.14E-05	-0.8361887	13	1.3	Prpf38b	
DHR2:226584001	2	226584001	226585000	1000	1	5.29E-05	-0.9159756	12	1.2	Bcar3	Signaling

DHR2:226967001	2	226967001	226968000	1000	1	1.80E-05	-1.1055945	17	1.7	Pde5a	Metabolism
DHR2:235415001	2	235415001	235416000	1000	1	6.27E-05	0.9484636	3	0.3		
DHR2:241930001	2	241930001	241931000	1000	1	7.27E-05	-0.7853713	10	1	Ppp3ca	Signaling
DHR2:253261001	2	253261001	253262000	1000	1	9.94E-05	0.7745969	7	0.7		
DHR2:256134001	2	256134001	256136000	2000	1	6.49E-05	-0.8816972	21	1.05		
DHR3:8889001	3	8889001	8890000	1000	1	7.86E-05	-1.0034981	18	1.8	Nup188	
DHR3:16512001	3	16512001	16513000	1000	1	9.63E-05	-1.2108901	7	0.7		
DHR3:16755001	3	16755001	16758000	3000	1	4.06E-05	-1.0570758	20	0.667	AABR07051551.1;AABR07051548.1;AABR07051548.2	
DHR3:19022001	3	19022001	19023000	1000	1	6.93E-05	-0.984172	8	0.8		
DHR3:29496001	3	29496001	29497000	1000	1	5.75E-05	-0.791405	11	1.1	Gtdc1	Metabolism
DHR3:60045001	3	60045001	60046000	1000	1	9.72E-05	-1.1209597	6	0.6	Scrn3;Gpr155	Unknown;Receptor
DHR3:63739001	3	63739001	63740000	1000	1	5.78E-07	-1.717991	16	1.6		
DHR3:74776001	3	74776001	74777000	1000	1	7.14E-05	0.9931325	1	0.1	Olr493;LOC100909940	
DHR3:82644001	3	82644001	82646000	2000	1	1.20E-05	-1.0890316	27	1.35	Ext2	Metabolism
DHR3:99085001	3	99085001	99087000	2000	1	3.76E-06	0.8458865	11	0.55		
DHR3:100509001	3	100509001	100512000	3000	1	4.19E-05	-0.83738	14	0.467		
DHR3:113425001	3	113425001	113426000	1000	1	3.00E-06	0.7609154	6	0.6	Ppip5k1;Serf2;Gm22953;Serinc4;Hypk	Signaling;Development
DHR3:116411001	3	116411001	116412000	1000	1	3.78E-05	-0.8511431	2	0.2		
DHR3:118476001	3	118476001	118477000	1000	1	1.41E-05	0.7129406	9	0.9		
DHR3:119330001	3	119330001	119331000	1000	1	4.33E-05	0.6654662	5	0.5	Trpm7	Development
DHR3:128042001	3	128042001	128043000	1000	1	9.26E-05	0.8032803	5	0.5		
DHR3:129493001	3	129493001	129494000	1000	1	8.76E-05	0.7551686	9	0.9	Ankef1	
DHR3:131242001	3	131242001	131243000	1000	1	6.46E-05	0.713007	9	0.9		
DHR3:134357001	3	134357001	134358000	1000	1	2.85E-05	-0.9979075	10	1	Sel1l2	Signaling
DHR3:135782001	3	135782001	135784000	2000	1	3.52E-05	0.8216436	13	0.65		
DHR3:137188001	3	137188001	137189000	1000	1	3.42E-05	-1.0289316	1	0.1		
DHR3:150202001	3	150202001	150203000	1000	1	7.11E-05	-1.1175325	7	0.7	AABR07054370.1	
DHR3:154249001	3	154249001	154250000	1000	1	8.87E-05	-1.0512363	12	1.2	Ctnnbl1	
DHR4:1273001	4	1273001	1274000	1000	1	4.20E-05	0.823012	5	0.5		
DHR4:13458001	4	13458001	13460000	2000	1	4.91E-05	-0.908667	38	1.9	Gnai1	Signaling
DHR4:32446001	4	32446001	32447000	1000	1	5.15E-07	-1.2180019	5	0.5		
DHR4:32542001	4	32542001	32543000	1000	1	4.90E-05	-0.8593466	19	1.9	Sdhaf3	
DHR4:35172001	4	35172001	35173000	1000	1	9.87E-05	0.7535196	3	0.3		
DHR4:39572001	4	39572001	39573000	1000	1	7.11E-05	-0.8559072	9	0.9		
DHR4:43110001	4	43110001	43111000	1000	1	8.35E-05	-0.552056	23	2.3		
DHR4:45443001	4	45443001	45444000	1000	1	5.13E-05	-1.2054199	9	0.9		
DHR4:52353001	4	52353001	52355000	2000	1	7.08E-05	-0.8413073	24	1.2	Tmem229a	
DHR4:56027001	4	56027001	56028000	1000	1	4.41E-05	-1.0070407	15	1.5	Snd1	Transcription
DHR4:60010001	4	60010001	60011000	1000	1	5.10E-05	-1.2440371	5	0.5		
DHR4:66288001	4	66288001	66289000	1000	1	6.69E-05	-0.8171584	5	0.5	Fmc1;U6;Luc7l2	Translation
DHR4:67163001	4	67163001	67165000	2000	1	1.93E-05	-1.235712	24	1.2	Slc37a3;Rab19	Transport;Signaling
DHR4:77884001	4	77884001	77885000	1000	1	4.15E-07	-1.0737226	13	1.3		
DHR4:78731001	4	78731001	78733000	2000	1	1.18E-05	-0.9870178	27	1.35	Malsu1	
DHR4:85880001	4	85880001	85881000	1000	1	6.17E-05	-0.9540889	4	0.4		
DHR4:108373001	4	108373001	108374000	1000	1	9.24E-06	-1.1895672	1	0.1		
DHR4:114887001	4	114887001	114888000	1000	1	7.04E-05	-1.1052411	33	3.3	Dctn1	Cytoskeleton
DHR4:150274001	4	150274001	150276000	2000	1	6.33E-05	-1.2155127	28	1.4		
DHR4:155112001	4	155112001	155113000	1000	1	7.49E-05	-1.044946	9	0.9	Phc1	Transcription
DHR4:158574001	4	158574001	158576000	2000	1	1.06E-05	-0.9453049	27	1.35	Ano2	
DHR4:159471001	4	159471001	159473000	2000	1	6.60E-07	-1.2152526	18	0.9	Dyrk4;Rad51ap1	Signaling
DHR4:160583001	4	160583001	160585000	2000	1	1.65E-05	-0.9621234	31	1.55	Tspan9	Cytoskeleton
DHR4:161954001	4	161954001	161955000	1000	1	3.00E-05	-0.8682761	7	0.7	Klrb1a	Immune
DHR5:1599001	5	1599001	1600000	1000	1	4.47E-05	0.8505375	1	0.1		
DHR5:6439001	5	6439001	6440000	1000	1	3.13E-05	0.8295412	4	0.4		
DHR5:7135001	5	7135001	7136000	1000	1	1.12E-06	-1.2219083	5	0.5	RGD1564053	Unknown
DHR5:14985001	5	14985001	14986000	1000	1	5.48E-05	0.6572401	16	1.6	Sumo4	
DHR5:19727001	5	19727001	19728000	1000	1	5.35E-05	1.1432794	16	1.6	AABR07047103.1	
DHR5:52873001	5	52873001	52874000	1000	1	8.17E-05	0.9040623	3	0.3		
DHR5:68861001	5	68861001	68862000	1000	1	9.37E-05	-0.8438889	7	0.7		
DHR5:79785001	5	79785001	79786000	1000	1	9.74E-05	-0.9777135	7	0.7	Tnc	Extracellular Matrix
DHR5:79884001	5	79884001	79885000	1000	1	2.64E-05	0.8148363	2	0.2	Tnc;AC229945.1	Extracellular Matrix
DHR5:90965001	5	90965001	90966000	1000	1	6.47E-05	-1.2415567	3	0.3	Kdm4c	Epigenetic
DHR5:97251001	5	97251001	97252000	1000	1	3.99E-05	0.6306608	7	0.7		
DHR5:117184001	5	117184001	117185000	1000	1	8.48E-05	-0.820993	20	2	Patj	Development
DHR5:123422001	5	123422001	123424000	2000	1	1.40E-06	-1.1274963	24	1.2		
DHR5:148108001	5	148108001	148109000	1000	1	3.02E-05	-1.6906671	11	1.1	U1	
DHR5:154126001	5	154126001	154127000	1000	1	9.27E-05	-0.816663	16	1.6	Myom3	Cytoskeleton
DHR5:156837001	5	156837001	156838000	1000	1	9.10E-05	-0.9944316	8	0.8		
DHR6:3256001	6	3256001	3257000	1000	1	6.63E-05	-1.1244324	10	1	Cdkl4	Cell Cycle
DHR6:4269001	6	4269001	4270000	1000	1	5.13E-06	0.7695982	5	0.5	Slc8a1	Transport
DHR6:6748001	6	6748001	6749000	1000	1	7.33E-05	-1.0128288	16	1.6		
DHR6:12629001	6	12629001	12631000	2000	1	7.07E-05	-1.0039061	13	0.65		
DHR6:18170001	6	18170001	18171000	1000	1	7.65E-05	0.8081564	2	0.2		
DHR6:31684001	6	31684001	31685000	1000	1	6.87E-05	-0.8181156	5	0.5		
DHR6:33381001	6	33381001	33382000	1000	1	8.23E-05	-0.9551569	7	0.7		
DHR6:36621001	6	36621001	36622000	1000	1	5.11E-05	0.7265301	8	0.8		
DHR6:44038001	6	44038001	44040000	2000	1	6.89E-05	-0.7655507	27	1.35	Mboat2	Metabolism

DHR6:75650001	6	75650001	75651000	1000	1	2.63E-06	-1.0578628	8	0.8	Snx6	Signaling
DHR6:80190001	6	80190001	80192000	2000	1	3.46E-05	0.8045049	20	1	AABR07064415.1	
DHR6:85724001	6	85724001	85725000	1000	1	3.96E-05	-0.9537558	6	0.6		
DHR6:93355001	6	93355001	93356000	1000	1	1.16E-05	-1.0651235	12	1.2	Frmd6;AABR07064747.2;AABR07064747.1	Signaling
DHR6:98630001	6	98630001	98631000	1000	1	6.49E-05	-0.9176177	8	0.8		
DHR6:118512001	6	118512001	118513000	1000	1	2.92E-05	1.0812277	2	0.2	AABR07065274.1	
DHR6:130045001	6	130045001	130046000	1000	1	7.46E-05	-0.8818698	14	1.4	AABR07065466.1	
DHR6:131113001	6	131113001	131114000	1000	1	2.19E-05	-1.1535003	6	0.6		
DHR7:1292001	7	1292001	1294000	2000	1	1.53E-05	-0.8877057	14	0.7	Pros1	Apoptosis
DHR7:2626001	7	2626001	2627000	1000	1	7.22E-05	-1.0358986	6	0.6	Gls2;Spryd4;Mip	Metabolism;Unknown;Transport
DHR7:8471001	7	8471001	8472000	1000	1	3.00E-05	-1.4603658	7	0.7		
DHR7:12018001	7	12018001	12019000	1000	1	3.64E-05	-1.0046708	26	2.6	Klf16;Rexo1	Transcription
DHR7:14051001	7	14051001	14052000	1000	1	4.63E-05	-1.2966109	23	2.3	Syde1;Ilvbl	Signaling;Metabolism
DHR7:23319001	7	23319001	23320000	1000	1	8.75E-05	-1.2560698	10	1		
DHR7:27884001	7	27884001	27886000	2000	1	1.70E-05	-1.2530933	57	2.85		
DHR7:29058001	7	29058001	29059000	1000	1	6.87E-05	0.7576284	14	1.4	Chpt1	Metabolism
DHR7:49418001	7	49418001	49419000	1000	1	8.59E-05	-1.0026752	9	0.9	Lin7a	Cell Junction
DHR7:59548001	7	59548001	59549000	1000	1	4.79E-05	-0.8203889	16	1.6	Cnot2	Transcription
DHR7:62440001	7	62440001	62441000	1000	1	5.01E-06	-0.8583683	11	1.1		
DHR7:67817001	7	67817001	67818000	1000	1	3.60E-05	0.6214101	2	0.2		
DHR7:68778001	7	68778001	68779000	1000	1	8.94E-06	-1.1207329	9	0.9		
DHR7:69396001	7	69396001	69397000	1000	1	9.87E-05	-0.9937711	12	1.2		
DHR7:91031001	7	91031001	91032000	1000	1	4.27E-05	-0.9378098	6	0.6		
DHR7:122660001	7	122660001	122661000	1000	1	3.90E-05	-1.0061646	8	0.8	Xpnpep3	Protease
DHR7:127415001	7	127415001	127416000	1000	1	8.51E-05	0.6417072	3	0.3	U7	
DHR7:133716001	7	133716001	133717000	1000	1	5.40E-05	-0.898477	24	2.4		
DHR8:3127001	8	3127001	3128000	1000	1	3.24E-05	0.8144041	4	0.4		
DHR8:8289001	8	8289001	8290000	1000	1	3.89E-06	0.9159748	8	0.8	Cntn5	Extracellular Matrix
DHR8:8456001	8	8456001	8457000	1000	1	5.59E-05	0.6336524	7	0.7	Cntn5	Extracellular Matrix
DHR8:16012001	8	16012001	16013000	1000	1	7.99E-05	-0.9458893	3	0.3		
DHR8:27364001	8	27364001	27366000	2000	1	9.43E-05	1.0360166	13	0.65		
DHR8:46870001	8	46870001	46871000	1000	1	4.95E-05	-1.1178574	11	1.1	Grik4	Signaling
DHR8:46980001	8	46980001	46981000	1000	1	9.06E-05	-0.92213	12	1.2	Grik4	Signaling
DHR8:47829001	8	47829001	47831000	2000	1	2.32E-06	-1.0289287	35	1.75		
DHR8:50143001	8	50143001	50144000	1000	1	4.47E-05	-1.0469205	15	1.5	Bace1	Protease
DHR8:51728001	8	51728001	51729000	1000	1	7.74E-05	-0.9585947	23	2.3		
DHR8:56266001	8	56266001	56267000	1000	1	1.38E-05	-1.0293583	4	0.4	Arhgap20	Signaling
DHR8:63718001	8	63718001	63719000	1000	1	4.42E-05	-1.1363514	10	1	Neo1	Receptor
DHR8:68351001	8	68351001	68352000	1000	1	1.07E-05	-1.0661717	11	1.1	Iqch;U4	
DHR8:100928001	8	100928001	100929000	1000	1	7.99E-05	0.852381	6	0.6		
DHR8:104665001	8	104665001	104666000	1000	1	2.86E-05	0.6827308	19	1.9	AABR07071208.2	
DHR8:105197001	8	105197001	105199000	2000	1	4.10E-05	-1.3129556	14	0.7	Trim42	Metabolism
DHR8:111301001	8	111301001	111302000	1000	1	2.42E-05	-1.0111264	10	1		
DHR8:124006001	8	124006001	124007000	1000	1	1.75E-05	-1.2564115	17	1.7		
DHR8:124502001	8	124502001	124503000	1000	1	4.30E-05	0.8491584	10	1	AABR07071620.1	
DHR9:4454001	9	4454001	4455000	1000	1	1.88E-05	-1.0715961	8	0.8	AABR07066180.1	
DHR9:20540001	9	20540001	20541000	1000	1	3.75E-05	0.9403876	7	0.7	Tnfrsf21	Apoptosis
DHR9:27655001	9	27655001	27656000	1000	1	2.85E-06	0.8474202	5	0.5	Kcnq5	Transport
DHR9:31686001	9	31686001	31687000	1000	1	8.91E-05	0.8162354	5	0.5	Adgrb3	
DHR9:38004001	9	38004001	38005000	1000	1	9.88E-05	-0.9720139	11	1.1	Dst	Cell Junction
DHR9:46232001	9	46232001	46233000	1000	1	5.61E-05	-0.8662051	8	0.8		
DHR9:50244001	9	50244001	50245000	1000	1	6.82E-05	0.7959233	12	1.2	Nck2	Cytoskeleton
DHR9:54818001	9	54818001	54819000	1000	1	1.11E-05	-1.1854993	11	1.1		
DHR9:60563001	9	60563001	60564000	1000	1	5.69E-05	-0.9244746	15	1.5	Hecw2	Proteolysis
DHR9:69411001	9	69411001	69412000	1000	1	5.32E-05	-1.118069	7	0.7	Pard3b	Cell Junction
DHR9:75126001	9	75126001	75128000	2000	1	8.11E-06	0.7920194	17	0.85	Erbb4	Signaling
DHR9:81369001	9	81369001	81370000	1000	1	5.71E-05	-0.8973605	3	0.3	Tns1	Signaling
DHR9:112796001	9	112796001	112797000	1000	1	5.96E-05	0.7673581	20	2		
DHR9:118452001	9	118452001	118453000	1000	1	5.23E-05	-1.0595153	13	1.3		
DHR9:121441001	9	121441001	121443000	2000	1	8.90E-05	-1.0982693	6	0.3		
DHR10:18726001	10	18726001	18728000	2000	1	4.73E-06	-1.1150161	22	1.1	Kcnip1	Signaling
DHR10:49106001	10	49106001	49107000	1000	1	1.70E-05	-0.9319299	29	2.9		
DHR10:51838001	10	51838001	51839000	1000	1	7.62E-05	0.6703069	13	1.3		
DHR10:97894001	10	97894001	97895000	1000	1	6.29E-05	-0.7557888	14	1.4	Wipi1	
DHR10:102605001	10	102605001	102606000	1000	1	2.97E-05	-1.0644822	20	2	AABR07030739.1	
DHR10:110548001	10	110548001	110549000	1000	1	5.43E-05	0.7326438	7	0.7	Wdr45b;Rab40b	Signaling
DHR11:27605001	11	27605001	27606000	1000	1	9.96E-08	-2.0705784	19	1.9	AABR07033564.2	
DHR11:38638001	11	38638001	38639000	1000	1	3.20E-05	-1.4217908	55	5.5		
DHR11:41933001	11	41933001	41935000	2000	1	9.57E-05	-1.0899743	16	0.8		
DHR11:54020001	11	54020001	54021000	1000	1	4.11E-05	0.7544521	4	0.4		
DHR11:63880001	11	63880001	63881000	1000	1	4.05E-05	-0.7825429	2	0.2		
DHR11:66837001	11	66837001	66838000	1000	1	3.32E-05	-1.0584527	5	0.5	Iqcb1	
DHR11:69558001	11	69558001	69559000	1000	1	2.43E-05	-0.9690355	8	0.8	Kalrn	Signaling
DHR11:70719001	11	70719001	70720000	1000	1	9.17E-05	-1.1324078	13	1.3	Snx4	Transport
DHR11:71981001	11	71981001	71982000	1000	1	1.72E-07	-1.9038537	13	1.3	Pak2;AABR07034444.1	Signaling
DHR11:81350001	11	81350001	81351000	1000	1	9.45E-05	-1.0825691	14	1.4	Adipoq;Rfc4	Hormone;Transcription
DHR11:87250001	11	87250001	87251000	1000	1	1.02E-06	-1.5132671	3	0.3	Tssk1b;LOC100910275	Signaling;Development

DHR11:88727001	11	88727001	88732000	5000	1	2.26E-05	-1.1275285	30	0.6	Fgd4	Signaling
DHR12:11340001	12	11340001	11341000	1000	1	7.96E-05	-1.4379343	9	0.9	AABR07035368.1;Kpna7	Unknown
DHR12:15269001	12	15269001	15272000	3000	1	3.48E-05	-0.8571638	39	1.3		
DHR12:27355001	12	27355001	27356000	1000	1	3.77E-05	-1.2195747	21	2.1	AABR07035916.1	
DHR12:28285001	12	28285001	28286000	1000	1	6.05E-05	-1.0117236	14	1.4		
DHR13:2675001	13	2675001	2676000	1000	1	5.74E-06	0.8996432	2	0.2		
DHR13:3680001	13	3680001	3682000	2000	1	8.73E-07	-1.1113007	6	0.3		
DHR13:23147001	13	23147001	23148000	1000	1	5.52E-05	0.8520438	0	0		
DHR13:31447001	13	31447001	31448000	1000	1	9.43E-05	0.6171175	5	0.5		
DHR13:33835001	13	33835001	33836000	1000	1	2.63E-05	-0.9501507	13	1.3		
DHR13:46891001	13	46891001	46893000	2000	1	2.01E-05	0.7845898	20	1	Thsd7b	Extracellular Matrix
DHR13:68300001	13	68300001	68301000	1000	1	4.04E-05	-1.1019191	26	2.6	Hmcn1	Immune
DHR13:87014001	13	87014001	87017000	3000	1	8.25E-05	0.7633912	31	1.033		
DHR13:90268001	13	90268001	90270000	2000	1	9.45E-05	0.5762701	8	0.4	Cd84	Immune
DHR13:105353001	13	105353001	105354000	1000	1	4.75E-05	0.8158702	11	1.1		
DHR13:108882001	13	108882001	108883000	1000	1	6.81E-05	-1.5079757	24	2.4		
DHR13:110647001	13	110647001	110648000	1000	1	4.28E-05	-1.738728	6	0.6	LOC100362350	Metabolism
DHR14:29360001	14	29360001	29361000	1000	1	2.67E-05	-0.8947882	4	0.4		
DHR14:35174001	14	35174001	35175000	1000	1	9.56E-05	-0.9034933	22	2.2		
DHR14:36722001	14	36722001	36724000	2000	1	2.47E-05	-1.0714569	32	1.6	Usp46	Proteolysis
DHR14:47916001	14	47916001	47917000	1000	1	7.26E-05	-1.2041612	6	0.6		
DHR14:55010001	14	55010001	55011000	1000	1	7.86E-05	-0.8734271	4	0.4		
DHR14:57729001	14	57729001	57731000	2000	1	6.56E-05	-0.9018415	8	0.4		
DHR14:70310001	14	70310001	70311000	1000	1	3.11E-06	-1.1152364	6	0.6		
DHR14:74571001	14	74571001	74572000	1000	1	9.25E-05	-0.6885014	8	0.8		
DHR14:76516001	14	76516001	76517000	1000	1	4.65E-05	-1.0464357	4	0.4		
DHR14:78052001	14	78052001	78053000	1000	1	7.65E-05	0.7352863	1	0.1	Stk32b	Signaling
DHR14:78356001	14	78356001	78357000	1000	1	1.04E-06	-1.2911639	14	1.4	AABR07015812.1	
DHR14:82786001	14	82786001	82789000	3000	1	6.93E-05	-0.9841837	53	1.767	Ctbp1	Transcription
DHR14:83376001	14	83376001	83377000	1000	1	3.23E-05	-1.0061456	10	1	Sfi1	Cell Cycle
DHR14:93351001	14	93351001	93353000	2000	1	3.16E-05	-0.8715448	6	0.3		
DHR14:93400001	14	93400001	93402000	2000	1	9.39E-05	0.7632929	10	0.5		
DHR14:107136001	14	107136001	107137000	1000	1	3.45E-05	-0.8882157	9	0.9	Ehbp1	Unknown
DHR14:115334001	14	115334001	115335000	1000	1	5.40E-05	0.8628217	5	0.5	Erlec1	Cytoskeleton
DHR15:1106001	15	1106001	1107000	1000	1	7.26E-05	-0.8870425	0	0		
DHR15:3961001	15	3961001	3963000	2000	1	6.77E-05	-1.2629192	24	1.2	Camk2g	Signaling
DHR15:6631001	15	6631001	6634000	3000	1	2.08E-06	1.044509	20	0.667		
DHR15:43307001	15	43307001	43309000	2000	1	6.56E-05	-0.8653286	11	0.55	Adra1a	Receptor
DHR15:69736001	15	69736001	69737000	1000	1	4.35E-05	1.0173969	13	1.3		
DHR15:78514001	15	78514001	78515000	1000	1	2.34E-05	0.8684286	3	0.3		
DHR15:88825001	15	88825001	88826000	1000	1	5.04E-05	0.6907558	16	1.6	Mycbp2	Metabolism
DHR15:93142001	15	93142001	93143000	1000	1	4.36E-05	-1.0719872	10	1	Mycbp2	Metabolism
DHR15:95110001	15	95110001	95111000	1000	1	7.73E-05	-0.6480265	10	1	AABR07019230.1	
DHR15:95641001	15	95641001	95643000	2000	1	1.29E-05	-1.111961	26	1.3		
DHR15:110938001	15	110938001	110939000	1000	1	1.15E-05	0.8101246	8	0.8		
DHR16:17869001	16	17869001	17870000	1000	1	5.09E-05	0.6741976	6	0.6		
DHR16:32600001	16	32600001	32601000	1000	1	5.26E-05	0.7442612	7	0.7		
DHR16:38198001	16	38198001	38200000	2000	1	7.08E-06	1.072743	17	0.85		
DHR16:46447001	16	46447001	46448000	1000	1	9.47E-05	0.7213156	21	2.1		
DHR16:47962001	16	47962001	47963000	1000	1	1.40E-05	-1.0732803	3	0.3	SNORA70	
DHR16:49194001	16	49194001	49195000	1000	1	8.40E-05	-0.9553863	10	1	Helt	Transcription
DHR16:56893001	16	56893001	56894000	1000	1	7.80E-06	-0.96008	8	0.8	Msr1	Receptor
DHR16:74106001	16	74106001	74107000	1000	1	4.56E-06	-1.5653227	10	1	Plat	Proteolysis
DHR16:75890001	16	75890001	75891000	1000	1	2.80E-05	-1.0699924	22	2.2		
DHR16:79859001	16	79859001	79860000	1000	1	1.06E-05	0.8630993	11	1.1		
DHR16:85090001	16	85090001	85092000	2000	1	8.07E-06	0.7756307	13	0.65		
DHR16:87147001	16	87147001	87149000	2000	1	1.53E-05	-0.9335426	29	1.45		
DHR16:88794001	16	88794001	88795000	1000	1	3.99E-06	0.561653	6	0.6		
DHR16:89798001	16	89798001	89799000	1000	1	1.58E-05	0.7470677	3	0.3		
DHR17:21742001	17	21742001	21743000	1000	1	5.47E-05	0.8978024	3	0.3	Gcnt2	Golgi
DHR17:22909001	17	22909001	22911000	2000	1	1.23E-05	0.8156904	24	1.2		
DHR17:23253001	17	23253001	23254000	1000	1	5.11E-05	-1.0149416	12	1.2		
DHR17:23828001	17	23828001	23829000	1000	1	7.17E-06	-0.9796267	14	1.4	Gfod1	Metabolism
DHR17:25845001	17	25845001	25846000	1000	1	2.60E-05	0.8301899	7	0.7		
DHR17:48405001	17	48405001	48407000	2000	1	4.19E-05	-0.849485	5	0.25	Amph	Receptor
DHR17:56975001	17	56975001	56976000	1000	1	6.74E-05	-1.0116909	7	0.7	Cul2	Proteolysis
DHR17:57711001	17	57711001	57713000	2000	1	4.91E-05	0.6215405	13	0.65	LOC291276	
DHR17:57900001	17	57900001	57904000	4000	1	1.33E-05	1.276817	40	1	AABR07028157.1	
DHR17:63456001	17	63456001	63457000	1000	1	9.45E-06	0.7265613	1	0.1		
DHR17:75773001	17	75773001	75774000	1000	1	8.00E-05	-0.8473315	25	2.5	Usp6nl	Signaling
DHR17:82517001	17	82517001	82518000	1000	1	6.08E-05	-0.7482592	6	0.6	Malrd1	
DHR17:90264001	17	90264001	90265000	1000	1	1.97E-05	0.8206907	5	0.5	Gng4	Signaling
DHR18:13021001	18	13021001	13022000	1000	1	5.06E-05	0.6912515	9	0.9	Ccdc178	
DHR18:18602001	18	18602001	18603000	1000	1	6.49E-05	1.076374	7	0.7		
DHR18:41297001	18	41297001	41298000	1000	1	7.27E-05	-1.00226	10	1		
DHR18:44917001	18	44917001	44920000	3000	1	9.92E-05	-0.8704409	15	0.5	AABR07032057.1	
DHR18:46425001	18	46425001	46426000	1000	1	7.78E-05	0.6825438	3	0.3		

DHR18:52953001	18	52953001	52954000	1000	1	3.40E-06	-1.3533743	19	1.9	Slc12a2	Transport
DHR18:65447001	18	65447001	65448000	1000	1	7.16E-06	-0.9004353	10	1	Tcf4	Transcription
DHR18:66817001	18	66817001	66818000	1000	1	2.09E-05	-0.9435946	7	0.7	Dcc	Receptor
DHR18:69225001	18	69225001	69226000	1000	1	2.66E-05	-1.0129672	20	2		
DHR19:4462001	19	4462001	4463000	1000	1	4.82E-05	-0.9866398	7	0.7	AABR07042699.1	
DHR19:8215001	19	8215001	8216000	1000	1	8.15E-05	0.8913094	7	0.7		
DHR19:26013001	19	26013001	26015000	2000	1	1.25E-05	0.8819238	18	0.9	Gcdh;Klf1;Dnase2	Metabolism;Transcription
DHR19:27967001	19	27967001	27968000	1000	1	5.29E-05	0.9483039	0	0		
DHR20:14863001	20	14863001	14865000	2000	1	7.41E-05	0.9095876	24	1.2		
DHR20:25699001	20	25699001	25700000	1000	1	1.34E-05	-0.9004754	9	0.9	AABR07044900.1	
DHR20:28021001	20	28021001	28022000	1000	1	2.94E-05	0.8067374	11	1.1	Ranbp2	Signaling
DHR20:42208001	20	42208001	42209000	1000	1	3.21E-05	0.7993813	3	0.3		
DHR20:44468001	20	44468001	44469000	1000	1	7.15E-05	-0.9568818	18	1.8	Fyn	Transcription
DHR20:50146001	20	50146001	50147000	1000	1	9.99E-06	-1.261639	23	2.3		
DHRX:23037001	X	23037001	23038000	1000	1	1.77E-05	0.9750752	6	0.6		
DHRX:46381001	X	46381001	46382000	1000	1	7.48E-05	0.9211089	5	0.5		
DHRX:57587001	X	57587001	57588000	1000	1	8.22E-05	-1.6040594	3	0.3		
DHRX:78469001	X	78469001	78470000	1000	1	5.85E-05	1.280849	3	0.3		
DHRX:80440001	X	80440001	80446000	6000	1	9.96E-05	-0.7485979	35	0.583		
DHRX:90691001	X	90691001	90692000	1000	1	6.45E-06	-0.9645124	20	2		
DHRX:107063001	X	107063001	107064000	1000	1	5.34E-05	0.4571739	10	1		
DHRX:135008001	X	135008001	135009000	1000	1	2.27E-05	1.0827008	8	0.8	Zdhhc9	Transcription
DHRX:138098001	X	138098001	138099000	1000	1	5.22E-06	-1.8291863	9	0.9	Stk26;Frmd7	
DHRX:146479001	X	146479001	146480000	1000	1	1.08E-05	1.099456	4	0.4		
DHRX:155859001	X	155859001	155860000	1000	1	7.09E-05	0.7332377	23	2.3	Dkc1	Transcription
DHRY:2797001	Y	2797001	2799000	2000	1	8.57E-05	1.0358915	16	0.8		
