# Supplementary material for: Epigenome-wide association study for atrazine induced transgenerational DNA methylation and histone retention sperm epigenetic biomarkers for disease
Source: PLoS One. 2020 Dec 16;15(12):e0239380. doi: 10.1371/journal.pone.0239380 (PMC7743986; doi:10.1371/journal.pone.0239380)
Supplement: S10 Table — DHR name, chromosome, start, stop, length, number signature windows, minimum p-value, max log-fold change, CpG number, CpG density, gene annotation, and gene category are presented. (PDF) [file pone.0239380.s017.pdf]

Supplemental Table S10
DHR Site List Puberty p<1e-04

DHR Name	Chr	Start	Stop	Length	# Sig Win	minP	maxLFC	CpG #	CpG Density	Gene Annotation	Gene Category
DHR1:5155001	1	5155001	5156000	1000	1	3.09E-05	-0.9336586	11	1.1	Grm1	Receptor
DHR1:9841001	1	9841001	9842000	1000	1	4.95E-06	-0.9405637	31	3.1	AABR07000325.1	
DHR1:11564001	1	11564001	11566000	2000	1	8.40E-05	0.7070941	9	0.45		
DHR1:89364001	1	89364001	89365000	1000	1	1.60E-05	-0.8156997	29	2.9	Mag;Hamp;Usf2	Extracellular Matrix;Transcription
DHR1:101583001	1	101583001	101584000	1000	1	2.54E-05	0.8123357	9	0.9		
DHR1:103235001	1	103235001	103236000	1000	1	9.31E-05	-0.9724028	7	0.7	Ptpn5	Signaling
DHR1:112366001	1	112366001	112367000	1000	1	6.62E-05	0.6992338	3	0.3	Luzp2;Gabrg3	Receptor
DHR1:128244001	1	128244001	128246000	2000	1	5.65E-05	0.9503906	10	0.5	Mef2a	Transcription
DHR1:139882001	1	139882001	139883000	1000	1	5.46E-05	0.744703	2	0.2	Ntrk3	Receptor
DHR1:141842001	1	141842001	141843000	1000	1	1.99E-05	-1.157478	23	2.3	Zfp710;Fuom	Transcription
DHR1:150704001	1	150704001	150705000	1000	1	5.74E-05	0.7062181	4	0.4		
DHR1:155278001	1	155278001	155280000	2000	1	2.20E-05	-0.9476218	19	0.95	AABR07004765.1	
DHR1:165759001	1	165759001	165760000	1000	1	2.11E-05	0.6737219	9	0.9	Fam168a	
DHR1:175529001	1	175529001	175531000	2000	1	3.03E-05	-0.9765962	18	0.9		
DHR1:188087001	1	188087001	188088000	1000	1	8.42E-05	-0.9436519	9	0.9	Syt17	Unknown
DHR1:196647001	1	196647001	196649000	2000	1	3.91E-05	-0.8134942	10	0.5	U1	
DHR1:210032001	1	210032001	210033000	1000	1	7.19E-05	0.5789802	12	1.2		
DHR1:212182001	1	212182001	212183000	1000	1	9.60E-05	-0.842071	9	0.9	Adgra1	
DHR1:244388001	1	244388001	244389000	1000	1	2.77E-05	0.7622196	5	0.5		
DHR1:251849001	1	251849001	251850000	1000	1	2.47E-05	-1.1697681	5	0.5	Rnls	Metabolism
DHR1:253803001	1	253803001	253804000	1000	1	9.16E-05	0.7197009	5	0.5		
DHR1:260097001	1	260097001	260098000	1000	1	4.92E-05	0.982424	8	0.8	Ccnj	Cell Cycle
DHR1:273054001	1	273054001	273055000	1000	1	1.08E-05	0.7845816	7	0.7	AABR07006957.1	
DHR1:279535001	1	279535001	279537000	2000	1	6.66E-05	-1.3117591	28	1.4		
DHR2:5126001	2	5126001	5128000	2000	1	9.40E-05	0.6975703	12	0.6	Fam172a	Unknown
DHR2:8520001	2	8520001	8521000	1000	1	7.40E-06	0.7784126	3	0.3		
DHR2:8926001	2	8926001	8928000	2000	1	2.00E-05	-0.9687052	21	1.05	Adgrv1	
DHR2:14323001	2	14323001	14324000	1000	1	3.34E-06	-0.8347112	2	0.2		
DHR2:18625001	2	18625001	18627000	2000	1	2.66E-05	-0.9085444	10	0.5		
DHR2:22246001	2	22246001	22247000	1000	1	2.38E-06	0.8485503	16	1.6	Serinc5	Metabolism
DHR2:22522001	2	22522001	22523000	1000	1	3.11E-05	0.777757	4	0.4		
DHR2:27099001	2	27099001	27100000	1000	1	4.90E-05	-0.7686873	8	0.8	Arhgef26	
DHR2:28522001	2	28522001	28523000	1000	1	5.84E-05	0.7855832	11	1.1		
DHR2:34749001	2	34749001	34750000	1000	1	1.67E-05	0.7227253	6	0.6	Cwc27	Metabolism
DHR2:36027001	2	36027001	36028000	1000	1	6.84E-05	0.87182	6	0.6		
DHR2:43879001	2	43879001	43881000	2000	1	6.62E-05	0.8385432	9	0.45		
DHR2:62078001	2	62078001	62079000	1000	1	9.83E-05	-0.834548	18	1.8		
DHR2:63857001	2	63857001	63860000	3000	1	4.01E-05	-1.0577897	7	0.233		
DHR2:64816001	2	64816001	64818000	2000	1	3.84E-05	-0.9668587	8	0.4		
DHR2:80551001	2	80551001	80552000	1000	1	1.32E-05	-0.8753584	22	2.2	Trio	Signaling
DHR2:105763001	2	105763001	105765000	2000	1	6.98E-05	0.7602533	11	0.55		
DHR2:108599001	2	108599001	108600000	1000	1	3.62E-05	0.766323	3	0.3		
DHR2:115361001	2	115361001	115362000	1000	1	1.12E-05	0.6659032	6	0.6	Eif5a2;Rpl22l1	Translation
DHR2:123311001	2	123311001	123312000	1000	1	8.51E-06	-0.8147126	23	2.3	Bbs7	
DHR2:140528001	2	140528001	140529000	1000	1	9.22E-05	0.8010388	8	0.8	Naa15	Metabolism
DHR2:147650001	2	147650001	147652000	2000	1	3.16E-05	0.7795636	26	1.3	Wwtr1	Transcription
DHR2:152188001	2	152188001	152189000	1000	1	3.28E-05	-0.7797807	5	0.5		
DHR2:160268001	2	160268001	160269000	1000	1	5.24E-06	-1.0897063	5	0.5		
DHR2:160950001	2	160950001	160951000	1000	1	2.72E-05	-1.008015	8	0.8		
DHR2:177833001	2	177833001	177834000	1000	1	8.87E-05	-0.8354176	7	0.7	AABR07011956.1;Rapgef2	
DHR2:191761001	2	191761001	191762000	1000	1	4.50E-05	0.8211201	3	0.3		
DHR2:195806001	2	195806001	195807000	1000	1	2.80E-05	-0.9254687	11	1.1	Snx27	Endocytosis
DHR2:207611001	2	207611001	207612000	1000	1	8.11E-05	-0.8233835	7	0.7		
DHR2:216422001	2	216422001	216423000	1000	1	3.37E-05	0.6790657	1	0.1	Amy1a	Metabolism
DHR2:226384001	2	226384001	226385000	1000	1	5.27E-05	-1.0241353	4	0.4		
DHR2:228358001	2	228358001	228359000	1000	1	5.78E-05	0.7205898	13	1.3		
DHR2:237154001	2	237154001	237155000	1000	1	1.24E-05	-1.0800932	17	1.7	Dkk2	
DHR2:248138001	2	248138001	248139000	1000	1	7.55E-05	0.7850484	6	0.6		
DHR2:254500001	2	254500001	254501000	1000	1	3.49E-05	-0.8439104	8	0.8		
DHR2:264474001	2	264474001	264476000	2000	1	9.01E-05	-0.7257411	9	0.45		
DHR3:3214001	3	3214001	3215000	1000	1	8.71E-05	0.9579524	4	0.4	AABR07051244.2	
DHR3:4042001	3	4042001	4043000	1000	1	2.93E-05	-1.0427921	25	2.5	Egfl7;Mir126a;Agpat2	Signaling;Metabolism

DHR3:11279001	3	11279001	11281000	2000	1	7.54E-06	-0.9571156	19	0.95	Pomt1;Uck1;AABR07051438.1	Golgi;Signaling
DHR3:21097001	3	21097001	21100000	3000	1	5.03E-05	0.7214369	8	0.267		
DHR3:21108001	3	21108001	21112000	4000	1	4.39E-05	0.6623301	8	0.2	Olr423	
DHR3:21409001	3	21409001	21410000	1000	1	2.07E-05	0.7219653	5	0.5		
DHR3:21435001	3	21435001	21436000	1000	1	3.51E-05	0.4727582	9	0.9	LOC102551549	
DHR3:23972001	3	23972001	23977000	5000	2	5.04E-06	-1.1449465	26	0.52		
DHR3:24123001	3	24123001	24124000	1000	1	7.03E-05	0.9115041	9	0.9		
DHR3:24556001	3	24556001	24558000	2000	1	7.02E-05	-1.040141	8	0.4		
DHR3:37283001	3	37283001	37284000	1000	1	2.21E-05	0.7376448	7	0.7		
DHR3:46271001	3	46271001	46272000	1000	1	8.26E-05	-0.9016147	19	1.9	U6	
DHR3:49435001	3	49435001	49436000	1000	1	9.41E-05	0.7892513	1	0.1		
DHR3:50818001	3	50818001	50819000	1000	1	9.33E-06	-1.2650576	8	0.8	AABR07052389.1	
DHR3:73773001	3	73773001	73774000	1000	1	1.89E-06	0.9055012	7	0.7	Olr505	
DHR3:73863001	3	73863001	73864000	1000	1	5.24E-06	0.7634964	8	0.8	Olfr1085	
DHR3:81126001	3	81126001	81127000	1000	1	4.94E-05	-0.7976629	4	0.4	Phf21a	Metabolism
DHR3:87809001	3	87809001	87810000	1000	1	8.13E-05	-3.009151	4	0.4		
DHR3:89067001	3	89067001	89069000	2000	1	2.10E-05	-0.8090411	11	0.55		
DHR3:89249001	3	89249001	89252000	3000	1	7.21E-05	-3.1982349	13	0.433		
DHR3:90047001	3	90047001	90048000	1000	1	9.04E-06	-2.4805545	13	1.3		
DHR3:93418001	3	93418001	93419000	1000	1	9.47E-05	0.6744952	4	0.4	Cat	Metabolism
DHR3:110219001	3	110219001	110220000	1000	1	8.58E-05	-0.9477035	10	1	Eif2ak4	Signaling
DHR3:134686001	3	134686001	134687000	1000	1	5.63E-05	0.6243822	26	2.6	Flrt3	Receptor
DHR3:143425001	3	143425001	143426000	1000	1	8.99E-05	-0.9044893	5	0.5	P22k15	
DHR3:156072001	3	156072001	156073000	1000	1	2.35E-05	0.8408246	11	1.1		
DHR4:1920001	4	1920001	1921000	1000	1	1.48E-07	-0.9930811	2	0.2		
DHR4:7408001	4	7408001	7409000	1000	1	5.40E-06	-1.0164639	10	1		
DHR4:10903001	4	10903001	10904000	1000	1	6.52E-05	0.7592668	3	0.3	Phtf2	Transcription
DHR4:15521001	4	15521001	15522000	1000	1	8.60E-05	-0.9179903	6	0.6		
DHR4:35903001	4	35903001	35905000	2000	1	1.81E-05	-0.9590485	12	0.6		
DHR4:46883001	4	46883001	46884000	1000	1	8.83E-05	-1.2476278	4	0.4		
DHR4:72177001	4	72177001	72178000	1000	1	4.44E-05	0.4893903	5	0.5		
DHR4:82396001	4	82396001	82398000	2000	1	6.59E-05	0.7122823	12	0.6	AABR07060588.2	
DHR4:85048001	4	85048001	85049000	1000	1	8.43E-05	0.6604163	4	0.4	Znrf2	Transcription
DHR4:99892001	4	99892001	99893000	1000	1	6.02E-05	-0.796255	6	0.6	Polr1a	Transcription
DHR4:100425001	4	100425001	100426000	1000	1	5.93E-05	-1.0668342	11	1.1	Capg;Elmod3	Cytoskeleton;Signaling
DHR4:100931001	4	100931001	100932000	1000	1	5.27E-05	0.6983819	11	1.1	Dnah6	
DHR4:101524001	4	101524001	101525000	1000	1	1.77E-05	-0.6295147	12	1.2	AABR07060944.1	
DHR4:109446001	4	109446001	109448000	2000	1	6.70E-05	0.6242186	10	0.5	AC115202.2	
DHR4:116545001	4	116545001	116546000	1000	1	4.06E-05	-0.7960816	13	1.3	Exoc6b	Transport
DHR4:120250001	4	120250001	120251000	1000	1	4.93E-05	-0.7206943	8	0.8	Eefsec	Transcription
DHR4:121360001	4	121360001	121361000	1000	1	3.21E-05	0.8704744	7	0.7	Chchd6	Transcription
DHR4:123714001	4	123714001	123716000	2000	1	3.59E-06	-1.0251227	19	0.95	Slc6a6	Transport
DHR4:126780001	4	126780001	126782000	2000	1	2.62E-05	0.701352	28	1.4		
DHR4:131384001	4	131384001	131386000	2000	1	9.85E-06	-0.7927947	33	1.65	Foxp1	Transcription
DHR4:134135001	4	134135001	134136000	1000	1	1.38E-06	0.7574641	4	0.4		
DHR4:148061001	4	148061001	148062000	1000	1	6.75E-05	0.8096046	7	0.7		
DHR4:151767001	4	151767001	151768000	1000	1	3.69E-05	0.7503762	2	0.2		
DHR4:165082001	4	165082001	165083000	1000	1	9.66E-05	0.8252927	1	0.1		
DHR4:170495001	4	170495001	170496000	1000	1	3.73E-05	0.7275253	6	0.6		
DHR4:183140001	4	183140001	183141000	1000	1	6.38E-06	0.8244872	8	0.8		
DHR5:8454001	5	8454001	8455000	1000	1	9.85E-05	0.6894334	10	1	Cpa6	Protease
DHR5:20728001	5	20728001	20729000	1000	1	2.77E-05	-0.8380074	12	1.2		
DHR5:24907001	5	24907001	24908000	1000	1	8.98E-05	-0.8948757	4	0.4	RGD1559441	Extracellular Matrix
DHR5:29364001	5	29364001	29365000	1000	1	8.71E-05	0.6534237	5	0.5		
DHR5:40584001	5	40584001	40585000	1000	1	5.99E-05	0.7504012	4	0.4		
DHR5:60847001	5	60847001	60850000	3000	1	3.04E-05	-1.2671633	42	1.4	Frmpd1;Trmt10b	Signaling
DHR5:129685001	5	129685001	129686000	1000	1	7.49E-05	0.7626113	5	0.5	Faf1	Apoptosis
DHR5:133777001	5	133777001	133778000	1000	1	6.72E-05	0.6853848	9	0.9	Cmpk1;Y_RNA	Signaling
DHR5:134154001	5	134154001	134155000	1000	1	8.79E-06	0.8689867	4	0.4		
DHR5:158441001	5	158441001	158442000	1000	1	3.42E-05	-1.0779174	19	1.9	Klhdc7a;AABR07050253.1	
DHR6:543001	6	543001	544000	1000	1	4.99E-05	0.7581808	5	0.5		
DHR6:3841001	6	3841001	3842000	1000	1	6.64E-05	0.776314	6	0.6		
DHR6:4224001	6	4224001	4225000	1000	1	2.17E-06	-0.7947259	26	2.6		
DHR6:8671001	6	8671001	8672000	1000	1	9.04E-07	-1.2042905	23	2.3	Camkmt	
DHR6:13303001	6	13303001	13305000	2000	1	8.98E-05	0.8819138	8	0.4		
DHR6:33339001	6	33339001	33341000	2000	1	6.33E-05	0.6513194	14	0.7		
DHR6:51378001	6	51378001	51379000	1000	1	7.11E-05	0.6353885	14	1.4		
DHR6:81153001	6	81153001	81154000	1000	1	6.25E-05	-0.8290133	23	2.3		
DHR6:89291001	6	89291001	89292000	1000	1	7.53E-05	-0.9194084	42	4.2		

DHR6:133790001	6	133790001	133792000	2000	1	1.93E-05	0.2655133	24	1.2	AABR07065531.7;AABR07065531.31;AABR07065531.18;AABR07065531.27;AABR07065531.10;AABR07065531.32;AABR07065531.34;AABR07065531.23;AABR07065531.13;AABR07065531.15;AABR07065531.9;AABR07065531.8;AABR07065531.16;AABR07065531.21;AABR07065531.17;AABR07065531.12;AABR07065531.25;AABR07065531.19;AABR07065532.8	
DHR6:134485001	6	134485001	134486000	1000	1	3.11E-05	0.7123031	10	1		
DHR6:144222001	6	144222001	144223000	1000	1	5.82E-05	0.7738973	8	0.8	Esyt2	
DHR7:14234001	7	14234001	14235000	1000	1	1.66E-05	-1.0762098	10	1	Brd4	Transcription
DHR7:19105001	7	19105001	19106000	1000	1	1.76E-05	0.7442994	3	0.3		
DHR7:21221001	7	21221001	21222000	1000	1	7.45E-05	0.5194983	2	0.2		
DHR7:51546001	7	51546001	51548000	2000	1	1.94E-05	0.6216305	32	1.6		
DHR7:73150001	7	73150001	73152000	2000	1	7.28E-05	-1.0003964	32	1.6	Matn2	Cytoskeleton
DHR7:76919001	7	76919001	76922000	3000	1	2.81E-06	-1.7087091	35	1.167		
DHR7:77425001	7	77425001	77428000	3000	1	7.37E-05	-0.972225	27	0.9		
DHR7:84245001	7	84245001	84246000	1000	1	3.97E-05	0.650922	0	0		
DHR7:90620001	7	90620001	90621000	1000	1	3.01E-05	0.6830315	3	0.3		
DHR7:96497001	7	96497001	96498000	1000	1	3.77E-05	-0.885292	5	0.5		
DHR7:97487001	7	97487001	97489000	2000	1	1.35E-05	-1.0051517	21	1.05		
DHR7:101331001	7	101331001	101333000	2000	1	1.18E-05	-1.295617	26	1.3	AABR07058124.5	
DHR7:116991001	7	116991001	116992000	1000	1	9.93E-05	0.8035346	7	0.7	Zfp623;RNaseP_nuc;Zfp707l1	Transcription
DHR7:117163001	7	117163001	117164000	1000	1	1.23E-06	-0.9546225	13	1.3		
DHR7:122629001	7	122629001	122630000	1000	1	2.15E-05	0.9258863	8	0.8	St13;Xpnpep3	Cytoskeleton;Protease
DHR7:139520001	7	139520001	139521000	1000	1	5.22E-05	-1.5184878	19	1.9	Senp1	Protease
DHR7:144002001	7	144002001	144004000	2000	1	1.66E-05	-1.2073464	28	1.4		
DHR8:7755001	8	7755001	7756000	1000	1	4.63E-05	-0.7454177	3	0.3		
DHR8:8369001	8	8369001	8370000	1000	1	6.88E-05	-0.8663886	5	0.5	Cntn5	Extracellular Matrix
DHR8:19487001	8	19487001	19488000	1000	1	7.83E-07	-0.9132345	2	0.2		
DHR8:39300001	8	39300001	39301000	1000	1	4.17E-07	-0.9398162	12	1.2	AC133739.1;Fez1	
DHR8:48255001	8	48255001	48256000	1000	1	8.85E-05	-1.0390042	19	1.9	U6	
DHR8:55323001	8	55323001	55324000	1000	1	2.58E-05	0.7383409	10	1	Sik2	Signaling
DHR8:57075001	8	57075001	57076000	1000	1	8.48E-05	-0.7575195	7	0.7		
DHR8:68222001	8	68222001	68223000	1000	1	3.78E-06	0.9733047	12	1.2	Map2k5	Signaling
DHR8:94545001	8	94545001	94548000	3000	1	6.80E-05	-0.9387716	34	1.133	Snap91	Transport
DHR8:99761001	8	99761001	99763000	2000	1	2.28E-05	0.9983355	17	0.85	LOC102551265	
DHR8:112687001	8	112687001	112688000	1000	1	2.07E-05	-0.7924141	7	0.7		
DHR8:123650001	8	123650001	123651000	1000	1	1.62E-05	-0.928366	13	1.3		
DHR9:18376001	9	18376001	18377000	1000	1	9.79E-06	-0.9663058	10	1	AABR07066830.1	
DHR9:53542001	9	53542001	53543000	1000	1	3.92E-05	-0.884497	5	0.5	Inpp1	Signaling
DHR9:71071001	9	71071001	71072000	1000	1	5.03E-05	-0.9358815	12	1.2		
DHR9:72346001	9	72346001	72347000	1000	1	2.10E-05	0.7857237	4	0.4		
DHR9:108051001	9	108051001	108052000	1000	1	5.88E-05	0.7209405	5	0.5		
DHR9:110909001	9	110909001	110910000	1000	1	2.82E-05	0.7526035	14	1.4	Fbxl17	Proteolysis
DHR10:12169001	10	12169001	12170000	1000	1	3.24E-05	-0.9148833	9	0.9		
DHR10:13469001	10	13469001	13470000	1000	1	7.10E-05	0.6251012	4	0.4	Pdpk1	Signaling
DHR10:19405001	10	19405001	19406000	1000	1	7.38E-05	-0.7883837	20	2		
DHR10:28297001	10	28297001	28298000	1000	1	1.38E-05	0.688781	4	0.4		
DHR10:50983001	10	50983001	50984000	1000	1	7.20E-05	-0.7189666	12	1.2	Hs3st3a1	Metabolism
DHR10:53057001	10	53057001	53059000	2000	1	9.00E-05	-0.7149073	8	0.4		
DHR10:53919001	10	53919001	53920000	1000	1	1.67E-05	-0.809047	11	1.1	Myh8;7SK;AABR07029836.1	Cytoskeleton
DHR10:53930001	10	53930001	53932000	2000	1	4.35E-05	-0.8045287	19	0.95	7SK;AABR07029836.1	
DHR10:58924001	10	58924001	58925000	1000	1	3.71E-05	-0.891964	38	3.8	Tekt1	Cytoskeleton
DHR10:65190001	10	65190001	65191000	1000	1	5.79E-06	0.9265498	7	0.7	Nufip2	
DHR10:68448001	10	68448001	68449000	1000	1	2.65E-06	-1.0893421	15	1.5	Asic2	Transport
DHR10:72905001	10	72905001	72906000	1000	1	2.00E-05	-0.8555345	14	1.4	7SK;Ppm1d	Signaling
DHR10:89772001	10	89772001	89773000	1000	1	9.07E-05	-0.9136294	14	1.4	AABR07030482.1	
DHR10:97584001	10	97584001	97585000	1000	1	5.47E-05	0.6512479	9	0.9	Rgs9	Signaling
DHR10:100045001	10	100045001	100046000	1000	1	8.02E-05	-0.9024248	11	1.1		
DHR11:1392001	11	1392001	1393000	1000	1	1.89E-05	-0.8890277	7	0.7		
DHR11:22017001	11	22017001	22018000	1000	1	9.22E-05	0.6654906	7	0.7		
DHR11:40688001	11	40688001	40689000	1000	1	2.76E-06	0.6698408	13	1.3		
DHR11:57600001	11	57600001	57601000	1000	1	9.32E-07	-0.9875635	16	1.6		

DHR11:66551001	11	66551001	66552000	1000	1	5.08E-05	0.8170625	3	0.3	Stxbp5l	Transcription
DHR11:73735001	11	73735001	73736000	1000	1	1.10E-05	-1.3242521	14	1.4	Tmem44	
DHR11:80067001	11	80067001	80068000	1000	1	1.50E-06	-1.0878378	10	1	AABR07034586.1	
DHR12:9584001	12	9584001	9585000	1000	1	6.87E-05	-0.8661228	12	1.2		
DHR12:14607001	12	14607001	14608000	1000	1	6.46E-06	-0.8261638	0	0	AABR07035437.1	
DHR12:28099001	12	28099001	28100000	1000	1	2.40E-05	-1.1054795	16	1.6		
DHR12:30550001	12	30550001	30551000	1000	1	1.49E-05	0.7004274	6	0.6	Nipsnap2;AABR07036011.1	
DHR12:32164001	12	32164001	32165000	1000	1	7.96E-05	-0.7943894	13	1.3	Tmem132d	Unknown
DHR12:46661001	12	46661001	46662000	1000	1	1.67E-05	-0.7947359	26	2.6	Bicdl1	
DHR13:9428001	13	9428001	9429000	1000	1	7.88E-05	0.7041397	4	0.4		
DHR13:11737001	13	11737001	11739000	2000	1	3.85E-05	0.66282	2	0.1		
DHR13:19307001	13	19307001	19308000	1000	1	9.31E-05	-0.7738956	6	0.6		
DHR13:33835001	13	33835001	33836000	1000	1	6.57E-05	-0.8821113	13	1.3		
DHR13:36936001	13	36936001	36937000	1000	1	6.97E-06	-0.9389439	4	0.4		
DHR13:38431001	13	38431001	38432000	1000	1	5.54E-05	-0.8694689	4	0.4		
DHR13:47821001	13	47821001	47823000	2000	1	3.60E-05	-1.2456372	35	1.75	Mapkapk2	Signaling
DHR13:57309001	13	57309001	57310000	1000	1	1.60E-05	-0.8829637	7	0.7	Kcnt2	Transport
DHR13:57688001	13	57688001	57689000	1000	1	8.16E-05	-0.8317721	6	0.6		
DHR13:71077001	13	71077001	71078000	1000	1	5.10E-05	-1.0334669	11	1.1	Rgs8	Signaling
DHR13:104456001	13	104456001	104458000	2000	1	1.00E-05	-0.8881639	14	0.7		
DHR13:110969001	13	110969001	110970000	1000	1	1.69E-05	-0.8950231	10	1	Kcnh1	Transport
DHR13:112958001	13	112958001	112959000	1000	1	5.64E-05	-0.8072789	5	0.5		
DHR14:13148001	14	13148001	13149000	1000	1	1.96E-05	-0.7989907	5	0.5		
DHR14:39819001	14	39819001	39820000	1000	1	5.12E-05	-0.8353704	7	0.7		
DHR14:41197001	14	41197001	41198000	1000	1	3.35E-05	0.7478181	7	0.7		
DHR14:44438001	14	44438001	44439000	1000	1	2.16E-05	0.7101443	6	0.6	Smim14	
DHR14:46243001	14	46243001	46244000	1000	1	6.64E-05	-0.7383597	9	0.9		
DHR15:1522001	15	1522001	1523000	1000	1	1.51E-05	0.7575941	1	0.1	AABR07016845.1	
DHR15:2799001	15	2799001	2801000	2000	1	2.74E-06	-1.0286063	44	2.2	Dupd1	Signaling
DHR15:13928001	15	13928001	13929000	1000	1	5.97E-05	-0.8306099	2	0.2		
DHR15:37131001	15	37131001	37132000	1000	1	9.60E-05	-0.8364336	9	0.9		
DHR15:54710001	15	54710001	54711000	1000	1	6.40E-05	0.8674695	5	0.5	AABR07018373.1	
DHR15:55920001	15	55920001	55921000	1000	1	4.34E-05	-0.8256487	8	0.8		
DHR15:58769001	15	58769001	58770000	1000	1	4.63E-05	-0.9186508	16	1.6		
DHR15:61035001	15	61035001	61037000	2000	1	9.27E-05	-0.9371148	18	0.9		
DHR15:63085001	15	63085001	63086000	1000	1	5.24E-05	-0.9941729	5	0.5		
DHR15:63831001	15	63831001	63832000	1000	1	9.98E-06	-1.0117382	27	2.7		
DHR15:69562001	15	69562001	69563000	1000	1	3.43E-05	-0.9284601	7	0.7		
DHR15:105846001	15	105846001	105848000	2000	1	2.47E-05	0.7967297	5	0.25	AABR07019442.3;Rap2a	Signaling
DHR16:4343001	16	4343001	4344000	1000	1	9.17E-05	0.6825264	10	1		
DHR16:13736001	16	13736001	13737000	1000	1	8.87E-05	0.9722085	4	0.4		
DHR16:44780001	16	44780001	44782000	2000	1	1.40E-05	0.8102841	18	0.9	AABR07025662.1	
DHR16:47769001	16	47769001	47770000	1000	1	5.77E-05	-0.8808472	10	1		
DHR16:53902001	16	53902001	53903000	1000	1	6.04E-05	-0.9093382	5	0.5		
DHR16:58325001	16	58325001	58326000	1000	1	6.02E-05	0.6484251	5	0.5		
DHR16:69267001	16	69267001	69268000	1000	1	9.34E-05	-0.8282721	8	0.8	AABR07026240.2	
DHR17:5569001	17	5569001	5570000	1000	1	8.14E-05	-0.8880275	12	1.2	Agtpbp1	Signaling
DHR17:14273001	17	14273001	14274000	1000	1	9.92E-05	-0.6993613	13	1.3		
DHR17:19782001	17	19782001	19784000	2000	1	3.55E-05	-0.8430511	13	0.65	U6	
DHR17:30064001	17	30064001	30066000	2000	1	9.13E-05	0.7499699	14	0.7		
DHR17:30356001	17	30356001	30357000	1000	1	2.14E-05	-0.842081	6	0.6		
DHR17:36282001	17	36282001	36284000	2000	1	8.56E-06	-0.8670596	18	0.9		
DHR17:37362001	17	37362001	37363000	1000	1	8.76E-05	-1.0302338	8	0.8	LOC103694082	
DHR17:62375001	17	62375001	62376000	1000	1	3.74E-05	-0.9143731	5	0.5	Ccny	Cell Cycle
DHR17:65559001	17	65559001	65560000	1000	1	2.88E-05	-1.2443514	13	1.3	Ryr2	Receptor
DHR17:70154001	17	70154001	70155000	1000	1	1.49E-05	-0.9497314	25	2.5		
DHR17:70987001	17	70987001	70988000	1000	1	2.21E-05	-0.9881125	11	1.1	Prkcq	Binding Protein
DHR17:76606001	17	76606001	76607000	1000	1	1.11E-05	-0.9451548	14	1.4	Camk1d	Signaling
DHR17:78161001	17	78161001	78162000	1000	1	7.31E-05	-0.849075	12	1.2		
DHR17:85090001	17	85090001	85091000	1000	1	3.89E-05	0.9395575	2	0.2	Dnajc1	Protein Binding
DHR17:87014001	17	87014001	87015000	1000	1	1.13E-05	0.7004189	6	0.6	AABR07028816.1	
DHR17:87464001	17	87464001	87465000	1000	1	6.16E-05	-0.8068052	1	0.1	Etl4	Unknown
DHR18:4462001	18	4462001	4463000	1000	1	6.11E-05	-0.7879983	12	1.2		
DHR18:7714001	18	7714001	7715000	1000	1	8.90E-05	-0.7664229	6	0.6		
DHR18:10118001	18	10118001	10119000	1000	1	8.03E-05	0.8137922	2	0.2		
DHR18:14643001	18	14643001	14644000	1000	1	8.27E-06	-0.9096424	8	0.8	Dtna	Cytoskeleton
DHR18:15795001	18	15795001	15796000	1000	1	8.82E-05	1.163989	7	0.7		
DHR18:17494001	18	17494001	17496000	2000	1	4.38E-05	-1.0809595	16	0.8	RGD1562608	EST
DHR18:36252001	18	36252001	36253000	1000	1	5.64E-05	0.8009555	7	0.7	Prelid2	

DHR18:38715001	18	38715001	38716000	1000	1	7.60E-05	0.9902841	4	0.4		
DHR18:47882001	18	47882001	47883000	1000	1	1.21E-05	-0.9805379	11	1.1	Sncaip	
DHR18:48001001	18	48001001	48002000	1000	1	8.69E-05	-0.9350709	16	1.6		
DHR18:53316001	18	53316001	53317000	1000	1	7.69E-05	-0.7703121	10	1		
DHR18:55455001	18	55455001	55456000	1000	1	9.97E-05	0.8674506	9	0.9	AABR07032229.1;LOC100910979	Signaling
DHR18:57103001	18	57103001	57104000	1000	1	2.17E-05	-0.7440918	10	1	Afap1l1	
DHR18:59720001	18	59720001	59721000	1000	1	5.29E-05	0.6800027	4	0.4		
DHR18:63982001	18	63982001	63983000	1000	1	1.02E-05	-0.940405	22	2.2	Ldlrad4	Receptor
DHR18:68937001	18	68937001	68938000	1000	1	2.02E-05	1.0294361	5	0.5	AABR07032503.1	
DHR18:79528001	18	79528001	79529000	1000	1	4.15E-05	-1.0936859	5	0.5	Zfp236	Transcription
DHR19:5586001	19	5586001	5587000	1000	1	8.98E-05	0.4281325	12	1.2	U6	
DHR19:6030001	19	6030001	6031000	1000	1	2.93E-05	0.6958191	17	1.7	AABR07042733.2	
DHR19:17267001	19	17267001	17268000	1000	1	3.16E-05	-1.1462803	25	2.5		
DHR19:17385001	19	17385001	17386000	1000	1	8.23E-05	-0.7804321	27	2.7	Chd9	Transcription
DHR19:18052001	19	18052001	18054000	2000	1	7.52E-05	-0.8261353	21	1.05	AABR07043031.4;AABR07043031.3	
DHR19:23504001	19	23504001	23505000	1000	1	8.25E-05	-0.9131606	5	0.5		
DHR19:27537001	19	27537001	27539000	2000	1	2.39E-05	0.7429474	10	0.5	Olr1666	Receptor
DHR19:28019001	19	28019001	28021000	2000	1	8.30E-05	0.5472411	17	0.85		
DHR19:28180001	19	28180001	28181000	1000	1	1.02E-07	0.8236086	6	0.6		
DHR19:29785001	19	29785001	29786000	1000	1	5.41E-05	0.652282	4	0.4	Inpp4b	Signaling
DHR19:31431001	19	31431001	31432000	1000	1	4.76E-05	0.7767158	6	0.6		
DHR19:31897001	19	31897001	31898000	1000	1	1.95E-06	0.872074	13	1.3	Abce1;Otud4	Receptor;Transcription
DHR19:55466001	19	55466001	55468000	2000	1	7.42E-05	-0.9802133	25	1.25	Cbfa2t3	Transcription
DHR20:13661001	20	13661001	13662000	1000	1	4.28E-05	0.802281	17	1.7	RGD1563815;Vpreb3;RGD1564162;LOC103694872;LOC103694874	Immune
DHR20:32626001	20	32626001	32627000	1000	1	2.97E-06	-0.9313794	9	0.9	Gprc6a	Receptor
DHR20:36336001	20	36336001	36337000	1000	1	2.78E-05	0.7049879	6	0.6		
DHR20:39799001	20	39799001	39800000	1000	1	2.40E-05	-0.8732971	3	0.3		
DHR20:40138001	20	40138001	40139000	1000	1	8.88E-05	0.6469047	4	0.4		
DHR20:52382001	20	52382001	52384000	2000	1	5.70E-05	0.715094	13	0.65		
DHR20:54197001	20	54197001	54199000	2000	1	8.37E-05	-0.8919252	13	0.65	Grik2	Signaling
DHRX:26867001	X	26867001	26868000	1000	1	3.01E-05	1.0173796	5	0.5		
DHRX:37315001	X	37315001	37316000	1000	1	7.02E-05	0.9712914	8	0.8		
DHRX:50366001	X	50366001	50368000	2000	1	3.27E-07	-1.644543	5	0.25		
DHRX:59194001	X	59194001	59195000	1000	1	7.38E-05	0.9829343	15	1.5		
DHRX:67145001	X	67145001	67146000	1000	1	1.54E-05	-1.4712646	1	0.1		
DHRX:70059001	X	70059001	70060000	1000	1	8.78E-05	1.0688633	5	0.5	Eda	Signaling
DHRX:73483001	X	73483001	73484000	1000	1	2.22E-05	1.0299173	6	0.6	Zfp36l3;LOC103694491;AABR07039303.4	
DHRX:116523001	X	116523001	116524000	1000	1	9.93E-05	-0.6384664	6	0.6		
DHRX:116648001	X	116648001	116649000	1000	1	2.79E-05	1.0183987	7	0.7	Lhfpl1	Development
DHRX:133889001	X	133889001	133890000	1000	1	3.36E-05	0.9368191	6	0.6		
DHRX:135180001	X	135180001	135181000	1000	1	6.12E-05	0.902422	8	0.8	Bcorl1	
DHRX:148655001	X	148655001	148656000	1000	1	1.77E-06	0.877733	4	0.4		
