# Supplementary material for: Epigenome-wide association study for atrazine induced transgenerational DNA methylation and histone retention sperm epigenetic biomarkers for disease
Source: PLoS One. 2020 Dec 16;15(12):e0239380. doi: 10.1371/journal.pone.0239380 (PMC7743986; doi:10.1371/journal.pone.0239380)
Supplement: S11 Table — DHR name, chromosome, start, stop, length, number signature windows, minimum p-value, max log-fold change, CpG number, CpG density, gene annotation, and gene category are presented. (PDF) [file pone.0239380.s018.pdf]

Supplemental Table S11
DHR Site List Multiple Disease p<1e-04

DHR Name	Chr	Start	Stop	Length	# Sig Win	minP	maxLFC	CpG #	CpG Density	Gene Annotation	Gene Category
DHR1:4567001	1	4567001	4568000	1000	1	8.10E-06	-0.7107059	14	1.4	Adgb	
DHR1:7057001	1	7057001	7058000	1000	1	4.18E-05	-0.6025268	13	1.3	Stx11	Transport
DHR1:19767001	1	19767001	19768000	1000	1	7.59E-06	-0.7605921	9	0.9		
DHR1:24595001	1	24595001	24596000	1000	1	2.27E-05	-0.578821	11	1.1	1700020N01Rik	
DHR1:35770001	1	35770001	35771000	1000	1	5.89E-05	0.606762	15	1.5		
DHR1:37887001	1	37887001	37888000	1000	1	7.41E-05	0.5872691	8	0.8	AABR07001099.1;LOC102549842	Transcription
DHR1:46219001	1	46219001	46220000	1000	1	8.30E-05	0.654559	8	0.8	Arid1b	Transcription
DHR1:63710001	1	63710001	63712000	2000	1	6.45E-05	1.5553853	19	0.95		
DHR1:63833001	1	63833001	63835000	2000	1	9.38E-05	1.7047106	49	2.45	Lilrb3l	Receptor
DHR1:64074001	1	64074001	64075000	1000	1	2.13E-06	-1.0152878	3	0.3	Rps9;Tmc4;Pirb	Translation;Metabolism
DHR1:65211001	1	65211001	65213000	2000	1	4.63E-05	1.6561186	45	2.25		
DHR1:66695001	1	66695001	66696000	1000	1	5.48E-05	0.7057261	2	0.2	Vom1r57	Unknown
DHR1:77901001	1	77901001	77904000	3000	1	7.58E-05	0.8557726	36	1.2	Bicra	
DHR1:80230001	1	80230001	80232000	2000	1	6.04E-05	0.7959437	53	2.65	Fosb	Transcription
DHR1:93633001	1	93633001	93634000	1000	1	4.58E-05	-0.5934809	13	1.3		
DHR1:112180001	1	112180001	112181000	1000	1	9.85E-05	-0.9369084	8	0.8	Luzp2;Gabrg3	Receptor
DHR1:116379001	1	116379001	116380000	1000	1	3.36E-06	0.6109815	4	0.4		
DHR1:123484001	1	123484001	123486000	2000	1	4.09E-05	0.7075713	6	0.3	AABR07004061.1	
DHR1:124090001	1	124090001	124091000	1000	1	4.83E-05	-0.7193426	10	1		
DHR1:148444001	1	148444001	148448000	4000	1	5.44E-05	1.3747652	252	6.3	Vbp1	Protein Binding
DHR1:164205001	1	164205001	164206000	1000	1	9.97E-05	1.0415863	25	2.5	Mogat2	Metabolism
DHR1:215238001	1	215238001	215240000	2000	1	3.58E-05	1.2915728	37	1.85	AABR07006049.1;LOC103690160	
DHR1:226820001	1	226820001	226822000	2000	1	3.90E-05	0.9980868	39	1.95	AABR07072028.1	
DHR1:243715001	1	243715001	243716000	1000	1	7.41E-05	-0.5766562	8	0.8		
DHR1:244738001	1	244738001	244739000	1000	1	1.79E-05	-0.6687072	6	0.6	Smarca2	Transcription
DHR1:264502001	1	264502001	264509000	7000	1	7.06E-05	1.3713546	341	4.871	Pax2	Transcription
DHR1:275049001	1	275049001	275050000	1000	1	3.47E-05	0.6383136	3	0.3		
DHR1:279103001	1	279103001	279104000	1000	1	3.62E-05	0.6496813	15	1.5	AABR07007065.1	
DHR2:61836001	2	61836001	61837000	1000	1	8.38E-05	0.9598461	13	1.3		
DHR2:67263001	2	67263001	67266000	3000	1	4.85E-05	-0.6796106	11	0.367		
DHR2:67867001	2	67867001	67868000	1000	1	3.18E-05	0.7132122	10	1		
DHR2:78152001	2	78152001	78153000	1000	1	2.63E-05	0.7372818	7	0.7	Zfp622	
DHR2:110223001	2	110223001	110224000	1000	1	8.72E-05	-0.6969929	2	0.2		
DHR2:124701001	2	124701001	124703000	2000	1	2.59E-05	-0.5901118	18	0.9	AABR07010121.1	
DHR2:127951001	2	127951001	127952000	1000	1	3.13E-05	-0.7773208	5	0.5	RGD1565989	
DHR2:141442001	2	141442001	141443000	1000	1	9.85E-05	-0.5356702	5	0.5	Foxo1	Transcription
DHR2:144602001	2	144602001	144603000	1000	1	4.14E-05	0.6531531	11	1.1	Sohlh2;U6	
DHR2:147632001	2	147632001	147633000	1000	1	2.17E-06	-0.706909	9	0.9	Wwtr1	Transcription
DHR2:173021001	2	173021001	173022000	1000	1	1.88E-05	-0.5729636	4	0.4	AABR07011857.2	
DHR2:203241001	2	203241001	203243000	2000	1	7.75E-06	-0.6831528	29	1.45	Vtcn1	
DHR2:205488001	2	205488001	205489000	1000	1	2.65E-05	1.1445925	63	6.3	7SK;Sike1	
DHR2:224434001	2	224434001	224435000	1000	1	5.33E-05	-0.5810537	19	1.9		
DHR2:238706001	2	238706001	238708000	2000	1	8.18E-05	-0.6906014	22	1.1	Gpbp1l2	
DHR2:244644001	2	244644001	244645000	1000	1	7.60E-05	-0.627294	17	1.7	Stpg2	Development
DHR3:6280001	3	6280001	6301000	21000	1	7.04E-05	1.3940806	434	2.067	Rxra	Receptor
DHR3:8861001	3	8861001	8862000	1000	1	1.72E-06	0.7863421	12	1.2	Dolk	
DHR3:27928001	3	27928001	27931000	3000	1	6.81E-05	-0.9821447	14	0.467		
DHR3:28496001	3	28496001	28497000	1000	1	9.44E-07	-0.8081313	11	1.1	Kynu	Metabolism
DHR3:33849001	3	33849001	33851000	2000	1	4.63E-05	-0.6340995	13	0.65		
DHR3:36583001	3	36583001	36584000	1000	1	7.96E-05	-0.5388444	11	1.1	AABR07052091.1	
DHR3:117067001	3	117067001	117068000	1000	1	8.55E-05	-0.4632735	3	0.3	5S_rRNA	
DHR3:124512001	3	124512001	124513000	1000	1	2.82E-05	-0.6702592	11	1.1	Prnp	Development
DHR3:125978001	3	125978001	125979000	1000	1	8.17E-05	-0.492357	7	0.7		
DHR3:131080001	3	131080001	131081000	1000	1	6.45E-05	-0.6261035	7	0.7		
DHR3:150956001	3	150956001	150957000	1000	1	9.82E-05	0.607022	9	0.9	Ncoa6	Transcription
DHR3:155585001	3	155585001	155586000	1000	1	1.55E-05	0.5739602	10	1		
DHR3:170054001	3	170054001	170055000	1000	1	5.86E-05	1.6520431	5	0.5		
DHR3:172073001	3	172073001	172074000	1000	1	8.97E-05	0.576533	3	0.3		
DHR4:2549001	4	2549001	2550000	1000	1	3.79E-05	0.633349	5	0.5	Ube3c	Metabolism
DHR4:5504001	4	5504001	5506000	2000	1	8.26E-05	-0.6057582	52	2.6		
DHR4:40311001	4	40311001	40312000	1000	1	3.67E-05	0.7467051	10	1		
DHR4:81215001	4	81215001	81216000	1000	1	4.66E-05	0.6698127	12	1.2	Nfe2l3	Transcription
DHR4:98015001	4	98015001	98016000	1000	1	3.45E-05	-0.6963593	6	0.6		
DHR4:110478001	4	110478001	110479000	1000	1	7.80E-05	-0.5196003	4	0.4		
DHR4:121253001	4	121253001	121260000	7000	1	7.12E-05	1.3792784	107	1.529	Plxna1	Receptor
DHR4:124328001	4	124328001	124329000	1000	1	4.58E-05	-0.6302718	8	0.8	Prickle2	Cytoskeleton
DHR4:132940001	4	132940001	132941000	1000	1	2.52E-05	-0.6046166	10	1	AABR07061542.1	

DHR4:139245001	4	139245001	139246000	1000	1	8.63E-05	-0.6096676	1	0.1		
DHR4:153514001	4	153514001	153518000	4000	1	7.69E-05	1.207551	100	2.5	Mical3	
DHR4:182221001	4	182221001	182222000	1000	1	1.61E-05	-0.6781355	12	1.2		
DHR4:183375001	4	183375001	183377000	2000	1	4.45E-05	-0.6194308	83	4.15	Caprin2	Transport
DHR5:6170001	5	6170001	6171000	1000	1	1.09E-05	-0.6253182	18	1.8	Sulf1	Metabolism
DHR5:9767001	5	9767001	9769000	2000	1	6.09E-05	-0.6732647	21	1.05		
DHR5:12752001	5	12752001	12753000	1000	1	3.77E-06	-0.6486385	10	1	AABR07046961.1	
DHR5:14322001	5	14322001	14323000	1000	1	1.67E-05	-0.6079278	4	0.4	Atp6v1h	Transport
DHR5:64467001	5	64467001	64468000	1000	1	3.59E-05	1.2318121	24	2.4	LOC108348074	
DHR5:144470001	5	144470001	144472000	2000	1	5.19E-05	1.1865975	47	2.35	Ago1	Transcription
DHR5:148345001	5	148345001	148356000	11000	1	5.02E-05	1.298587	242	2.2	Pef1;Hcrtr1	Signaling;Receptor
DHR5:163443001	5	163443001	163444000	1000	1	9.14E-05	0.6914907	12	1.2		
DHR6:11780001	6	11780001	11781000	1000	1	4.16E-05	0.6897074	3	0.3		
DHR6:27430001	6	27430001	27432000	2000	1	2.65E-05	0.5965369	25	1.25	Otof;Drc1	Transport
DHR6:37774001	6	37774001	37776000	2000	1	1.73E-05	0.6971304	19	0.95		
DHR6:51770001	6	51770001	51771000	1000	1	9.13E-05	0.6246846	2	0.2		
DHR6:67296001	6	67296001	67298000	2000	1	3.02E-06	-0.7637376	4	0.2		
DHR6:107588001	6	107588001	107589000	1000	1	3.26E-05	0.7050116	3	0.3	AC094055.1;Acot6;Dnal1	Cytoskeleton
DHR6:130595001	6	130595001	130596000	1000	1	6.58E-05	0.620442	10	1		
DHR6:132724001	6	132724001	132725000	1000	1	8.71E-05	0.5903507	15	1.5	Yy1	Transcription
DHR6:141679001	6	141679001	141680000	1000	1	7.96E-05	-0.7523598	3	0.3		
DHR7:2487001	7	2487001	2488000	1000	1	4.56E-05	0.661356	4	0.4	Ptges3	
DHR7:51817001	7	51817001	51819000	2000	1	3.50E-05	-0.8934344	16	0.8	Otogl	Unknown
DHR7:65271001	7	65271001	65272000	1000	1	1.83E-05	-0.757805	4	0.4	Hmga2	Transcription
DHR7:89372001	7	89372001	89373000	1000	1	5.65E-05	0.5305596	10	1		
DHR7:96145001	7	96145001	96146000	1000	1	7.86E-05	-0.6343368	4	0.4		
DHR7:107852001	7	107852001	107854000	2000	1	1.27E-05	0.6167453	17	0.85	AC091481.1	
DHR7:116057001	7	116057001	116059000	2000	1	6.96E-05	0.5819219	30	1.5	LOC100910068;Gml	
DHR8:17588001	8	17588001	17589000	1000	1	8.16E-05	1.1594329	50	5		
DHR8:38255001	8	38255001	38256000	1000	1	6.24E-05	0.8709752	4	0.4		
DHR8:48123001	8	48123001	48124000	1000	1	9.54E-05	1.1614419	11	1.1	Nectin1	
DHR8:55110001	8	55110001	55112000	2000	1	5.50E-06	-0.7761425	29	1.45	Dixdc1	Signaling
DHR8:57573001	8	57573001	57574000	1000	1	2.57E-05	-0.5211169	8	0.8		
DHR8:59113001	8	59113001	59114000	1000	1	5.12E-05	0.5803528	15	1.5	AC112328.1;Sh2d7;Cib2	Signaling
DHR8:85450001	8	85450001	85451000	1000	1	4.76E-05	0.617115	16	1.6	Ick	Signaling
DHR8:125613001	8	125613001	125614000	1000	1	4.06E-05	-0.6435649	11	1.1	Rbms3	Epigenetic
DHR9:194001	9	194001	195000	1000	1	4.21E-05	-0.6145198	6	0.6		
DHR9:37272001	9	37272001	37273000	1000	1	3.61E-05	-0.5759204	7	0.7		
DHR9:41538001	9	41538001	41539000	1000	1	3.48E-05	-0.8490988	4	0.4		
DHR9:77710001	9	77710001	77712000	2000	1	5.79E-05	-0.5823852	10	0.5	Spag16	Cytoskeleton
DHR10:3652001	10	3652001	3653000	1000	1	5.52E-06	0.8936111	6	0.6	Cpped1	Signaling
DHR10:6555001	10	6555001	6556000	1000	1	9.08E-05	0.6019098	4	0.4		
DHR10:25396001	10	25396001	25397000	1000	1	3.91E-05	-0.6499899	9	0.9		
DHR10:35785001	10	35785001	35786000	1000	1	6.54E-05	0.7060289	7	0.7	Maml1	Transcription
DHR10:37312001	10	37312001	37316000	4000	1	6.36E-05	1.5585208	266	6.65	Jade2	
DHR10:44346001	10	44346001	44347000	1000	1	5.34E-05	-0.6691205	7	0.7	Olr1435	Receptor
DHR10:54132001	10	54132001	54133000	1000	1	3.57E-05	-0.6800589	5	0.5	Gas7	Transcription
DHR10:55413001	10	55413001	55415000	2000	1	1.49E-05	0.8366452	16	0.8	Myh10;Ndel1	Cytoskeleton;Cell Cycle
DHR10:77037001	10	77037001	77038000	1000	1	4.57E-06	-0.7383198	13	1.3		
DHR10:90638001	10	90638001	90640000	2000	1	8.39E-07	0.783859	11	0.55	Meioc;Ccdc43	
DHR10:101370001	10	101370001	101373000	3000	1	4.45E-05	1.0664311	66	2.2		
DHR11:3053001	11	3053001	3055000	2000	1	1.74E-05	0.8178528	13	0.65		
DHR11:40781001	11	40781001	40782000	1000	1	6.32E-05	0.5537709	15	1.5		
DHR11:40888001	11	40888001	40889000	1000	1	9.34E-05	0.5779444	7	0.7		
DHR11:51244001	11	51244001	51245000	1000	1	2.47E-07	0.7637744	7	0.7		
DHR11:54590001	11	54590001	54591000	1000	1	7.42E-05	-0.6240446	9	0.9		
DHR11:67246001	11	67246001	67247000	1000	1	1.55E-05	-0.7570761	5	0.5	Casr;7SK	Receptor
DHR11:78433001	11	78433001	78434000	1000	1	2.82E-05	0.6531551	14	1.4	Tp63	Transcription
DHR11:86990001	11	86990001	86994000	4000	1	7.55E-05	1.4073901	128	3.2	Rtn4r	Receptor
DHR12:13894001	12	13894001	13896000	2000	1	8.42E-05	1.0939974	52	2.6	Tnrc18	Transcription
DHR12:24165001	12	24165001	24166000	1000	1	9.30E-05	0.7771461	22	2.2	Ccl26	
DHR12:39646001	12	39646001	39647000	1000	1	9.21E-05	0.9675001	12	1.2	Anapc7;Arpc3	Cell Cycle;Cytoskeleton
DHR13:24633001	13	24633001	24634000	1000	1	1.54E-05	-0.6590088	5	0.5		
DHR13:44200001	13	44200001	44201000	1000	1	7.41E-05	0.9509641	10	1	Tmem163	Unknown
DHR13:65319001	13	65319001	65320000	1000	1	2.21E-05	0.8415614	0	0		
DHR13:83422001	13	83422001	83423000	1000	1	1.11E-05	-0.7690951	17	1.7	Tbx19	Transcription
DHR13:102220001	13	102220001	102221000	1000	1	1.72E-05	-0.6785258	13	1.3		
DHR13:113779001	13	113779001	113780000	1000	1	6.91E-05	0.7313767	5	0.5	Mir29b2;Mir3556b;Cd46	Immune
DHR14:34427001	14	34427001	34429000	2000	1	7.39E-05	0.90331	20	1		
DHR14:34477001	14	34477001	34479000	2000	1	3.81E-05	-0.665004	18	0.9	Clock	Cell Cycle
DHR14:45171001	14	45171001	45172000	1000	1	6.36E-05	-0.5651528	14	1.4	Klf3	Transcription
DHR14:105865001	14	105865001	105866000	1000	1	5.93E-05	-0.6918883	13	1.3		

DHR14:107908001	14	107908001	107910000	2000	1	9.22E-06	0.6890592	22	1.1	AABR07016628.1	
DHR15:8181001	15	8181001	8184000	3000	1	7.77E-05	-0.6210421	61	2.033	Ube2e1;Nkiras1;Rpl15	Metabolism;Signaling;Translation
DHR15:74261001	15	74261001	74262000	1000	1	6.05E-06	-0.6533015	8	0.8		
DHR15:97870001	15	97870001	97871000	1000	1	1.68E-05	-0.7214282	3	0.3		
DHR15:110925001	15	110925001	110926000	1000	1	3.19E-05	-0.6978495	1	0.1		
DHR16:16190001	16	16190001	16191000	1000	1	8.50E-05	-0.6620755	4	0.4		
DHR16:29562001	16	29562001	29563000	1000	1	8.11E-05	-0.8251824	1	0.1		
DHR16:50657001	16	50657001	50658000	1000	1	9.50E-05	-0.5579316	5	0.5		
DHR16:62399001	16	62399001	62400000	1000	1	8.77E-06	-0.6458226	9	0.9		
DHR16:74589001	16	74589001	74590000	1000	1	6.32E-05	0.7592795	10	1	Tpte2;AABR07026382.1	Signaling
DHR17:38576001	17	38576001	38578000	2000	1	8.34E-05	0.7603739	41	2.05		
DHR17:42620001	17	42620001	42621000	1000	1	5.79E-05	-0.613019	10	1	Cmahp	
DHR17:48765001	17	48765001	48766000	1000	1	1.18E-05	-0.6664279	15	1.5	Vps41	Transport
DHR17:49848001	17	49848001	49850000	2000	1	1.02E-05	0.6287074	17	0.85	Cdk13	Signaling
DHR17:72939001	17	72939001	72940000	1000	1	6.23E-05	-0.5998206	18	1.8		
DHR17:74920001	17	74920001	74921000	1000	1	4.57E-05	-0.7662315	11	1.1		
DHR17:75155001	17	75155001	75156000	1000	1	7.27E-05	0.5634646	24	2.4		
DHR18:15291001	18	15291001	15292000	1000	1	2.92E-06	-0.8129838	18	1.8	LOC498826;Trappc8	
DHR18:37813001	18	37813001	37815000	2000	1	3.12E-06	-0.7404515	16	0.8	Dpysl3	Metabolism
DHR18:41579001	18	41579001	41580000	1000	1	4.88E-05	0.665933	6	0.6		
DHR18:59576001	18	59576001	59577000	1000	1	1.04E-05	-0.6341504	6	0.6		
DHR18:76735001	18	76735001	76736000	1000	1	4.54E-05	1.4898582	26	2.6	Txnl4a;RGD1560212	
DHR19:45254001	19	45254001	45255000	1000	1	9.96E-05	0.5599936	9	0.9		
DHR19:48968001	19	48968001	48970000	2000	1	2.50E-05	0.6135647	19	0.95	AABR07043940.1	
DHR19:53330001	19	53330001	53331000	1000	1	8.37E-05	1.2389236	24	2.4		
DHR19:53963001	19	53963001	53967000	4000	1	9.00E-05	1.3741607	100	2.5		
DHR20:2052001	20	2052001	2053000	1000	1	3.04E-05	-0.602228	10	1	RT1-M4;AC108572.6;RT1-M6-1	Immune
DHR20:18519001	20	18519001	18521000	2000	1	7.53E-05	0.9215741	23	1.15		
DHR20:42000001	20	42000001	42002000	2000	1	6.69E-05	1.4545144	15	0.75		
DHR20:42316001	20	42316001	42317000	1000	1	6.26E-05	-0.526354	14	1.4		
DHR20:44021001	20	44021001	44022000	1000	1	6.66E-05	0.6578663	13	1.3		
DHRX:48109001	X	48109001	48110000	1000	1	2.68E-05	0.8620462	4	0.4		
DHRX:51935001	X	51935001	51936000	1000	1	7.95E-05	0.9124652	9	0.9	Dmd	Development
DHRX:106854001	X	106854001	106855000	1000	1	2.07E-05	1.2203468	45	4.5		
DHRX:146956001	X	146956001	146957000	1000	1	2.62E-06	1.0923527	1	0.1		
