# Supplementary material for: Epigenome-wide association study for atrazine induced transgenerational DNA methylation and histone retention sperm epigenetic biomarkers for disease
Source: PLoS One. 2020 Dec 16;15(12):e0239380. doi: 10.1371/journal.pone.0239380 (PMC7743986; doi:10.1371/journal.pone.0239380)
Supplement: S12 Table — DHR site list for atrazine versus control sperm DHRs at p<1e-04. DHR name, chromosome, start, stop, length, number signature windows, minimum p-value, max log-fold change, CpG number, CpG density, gene annotation, and gene category are presented. (PDF) [file pone.0239380.s019.pdf]

Supplemental Table S12
DMR Site List Atrazine versus Control Sperm DHRs p<1e-04

DHR Name	Chr	Start	Stop	Length	# Sig Win	minP	maxLFC	CpG #	CpG Density	Gene Annotation	Gene Category
DHR1:8670001	1	8670001	8671000	1000	1	4.68E-05	0.3804552	15	1.5	Adgrg6	
DHR1:19057001	1	19057001	19058000	1000	1	2.22E-05	0.5709254	16	1.6	Lama2	Extracellular Matrix
DHR1:26261001	1	26261001	26265000	4000	1	8.30E-05	-0.4060834	67	1.675		
DHR1:26617001	1	26617001	26622000	5000	1	7.07E-05	-0.4682669	132	2.64		
DHR1:26751001	1	26751001	26754000	3000	1	4.20E-06	-0.6205155	30	1		
DHR1:26967001	1	26967001	26969000	2000	1	5.89E-05	-0.4845083	13	0.65		
DHR1:26991001	1	26991001	26994000	3000	1	9.53E-05	-0.3209871	59	1.967		
DHR1:30721001	1	30721001	30723000	2000	1	6.87E-05	0.5035121	9	0.45	Rspo3	
DHR1:34238001	1	34238001	34240000	2000	1	6.41E-05	0.4260447	33	1.65		
DHR1:36964001	1	36964001	36965000	1000	1	4.01E-05	0.473788	4	0.4	AABR07001064.1	
DHR1:38317001	1	38317001	38318000	1000	1	9.30E-06	-0.6005736	3	0.3		
DHR1:38926001	1	38926001	38927000	1000	1	3.97E-05	-0.4438118	4	0.4	AABR07001233.1	
DHR1:39011001	1	39011001	39018000	7000	1	8.34E-05	-0.2973449	75	1.071	AABR07001233.1	
DHR1:41741001	1	41741001	41742000	1000	1	5.46E-05	-0.5865211	13	1.3	AABR07001382.1	
DHR1:41775001	1	41775001	41776000	1000	1	3.02E-05	-0.5974704	11	1.1	AABR07001382.1	
DHR1:51534001	1	51534001	51535000	1000	1	5.96E-05	0.4798119	10	1	AABR07001555.1	
DHR1:53804001	1	53804001	53805000	1000	1	8.48E-05	-0.5094693	1	0.1	Afdn	
DHR1:54166001	1	54166001	54174000	8000	1	9.89E-05	-0.3591433	51	0.637		
DHR1:54393001	1	54393001	54396000	3000	1	9.92E-06	-0.5273576	44	1.467		
DHR1:55408001	1	55408001	55412000	4000	1	9.56E-05	-0.4518132	31	0.775	AABR07001700.1	
DHR1:55981001	1	55981001	55989000	8000	1	5.37E-05	-0.2847325	59	0.738		
DHR1:72235001	1	72235001	72236000	1000	1	1.70E-05	-0.4748153	10	1	Vom1r38	
DHR1:73732001	1	73732001	73734000	2000	1	3.85E-05	-0.5063322	99	4.95	Leng9;Leng8;Ttyh1	Transport;Receptor
DHR1:78292001	1	78292001	78294000	2000	1	7.55E-06	-0.7068095	17	0.85	Sae1	Metabolism
DHR1:78449001	1	78449001	78450000	1000	1	2.98E-05	-0.7494831	17	1.7	Npas1;Arhgap35	Transcription;Signaling
DHR1:78890001	1	78890001	78891000	1000	1	5.17E-05	-0.564107	17	1.7	Pnma8a	
DHR1:80308001	1	80308001	80314000	6000	1	4.94E-05	-0.7596314	114	1.9	Ercc2;Mir343;Klc3;Ckm	;Signaling
DHR1:80370001	1	80370001	80371000	1000	1	2.63E-06	-0.5406296	8	0.8	Mark4;U1;AABR07002677.2	Signaling
DHR1:80439001	1	80439001	80442000	3000	1	3.97E-05	-1.1648387	74	2.467	Nkpd1;Ppp1r37	
DHR1:80457001	1	80457001	80462000	5000	1	2.45E-05	-0.6269404	42	0.84	Ppp1r37	
DHR1:80643001	1	80643001	80645000	2000	1	7.56E-05	-0.6166042	32	1.6	Nectin2	
DHR1:82916001	1	82916001	82920000	4000	1	3.12E-07	-0.8817576	61	1.525	RGD1565655	
DHR1:83456001	1	83456001	83459000	3000	1	8.14E-05	-0.4230568	14	0.467		
DHR1:83987001	1	83987001	83989000	2000	1	8.41E-05	-0.5418756	20	1	Egln2;Rab4b;Mia	anscription
DHR1:84584001	1	84584001	84587000	3000	1	2.94E-05	-0.5795153	35	1.167	AABR07071891.1	
DHR1:84914001	1	84914001	84915000	1000	1	4.52E-06	-0.5265645	7	0.7		
DHR1:88168001	1	88168001	88171000	3000	1	3.97E-05	-0.6651678	52	1.733	Catsperg;Kcnk6	Transport
DHR1:91370001	1	91370001	91371000	1000	1	5.82E-05	-0.5468735	13	1.3	Cebpa	Transcription
DHR1:95746001	1	95746001	95749000	3000	1	2.00E-05	-0.4067668	35	1.167		
DHR1:96231001	1	96231001	96235000	4000	1	6.76E-05	-0.3961176	42	1.05		
DHR1:96240001	1	96240001	96243000	3000	1	6.67E-05	-0.4080428	57	1.9		
DHR1:96328001	1	96328001	96331000	3000	1	6.54E-05	-0.4614996	55	1.833		
DHR1:96515001	1	96515001	96516000	1000	1	8.10E-06	-0.4933311	11	1.1		
DHR1:97687001	1	97687001	97692000	5000	1	1.02E-05	0.6743139	19	0.38		
DHR1:102784001	1	102784001	102785000	1000	1	2.05E-05	0.4117273	2	0.2	Saa4	Transport
DHR1:106223001	1	106223001	106225000	2000	1	6.96E-06	0.4402913	15	0.75	Nell1	Development
DHR1:108022001	1	108022001	108026000	4000	1	2.21E-05	0.4349815	21	0.525		
DHR1:108416001	1	108416001	108418000	2000	1	6.20E-05	0.4567489	8	0.4		
DHR1:114337001	1	114337001	114339000	2000	1	8.51E-05	0.4432205	12	0.6	Cyfip1;U6;Nipa2	Signaling
DHR1:123209001	1	123209001	123212000	3000	1	2.12E-05	-0.4537574	27	0.9		
DHR1:149108001	1	149108001	149110000	2000	1	8.12E-05	0.4172262	9	0.45	Vom2r42	
DHR1:153599001	1	153599001	153600000	1000	1	4.42E-05	0.4067613	6	0.6	Fzd4	Receptor
DHR1:167902001	1	167902001	167904000	2000	1	5.22E-05	0.46042	13	0.65	Olr60;AC096030.3;Olr59	Receptor
DHR1:179493001	1	179493001	179495000	2000	1	1.50E-05	-0.4018969	31	1.55		
DHR1:179502001	1	179502001	179504000	2000	1	2.28E-05	-0.6269054	14	0.7		
DHR1:180426001	1	180426001	180429000	3000	1	6.70E-05	-0.5091748	17	0.567		
DHR1:181481001	1	181481001	181485000	4000	1	1.98E-06	-0.5629556	43	1.075		
DHR1:181505001	1	181505001	181509000	4000	1	1.27E-06	-0.5938285	29	0.725		
DHR1:181764001	1	181764001	181767000	3000	1	2.00E-05	-0.7727468	21	0.7		
DHR1:182205001	1	182205001	182213000	8000	1	3.22E-05	-0.3941317	77	0.963		
DHR1:182455001	1	182455001	182458000	3000	1	6.60E-06	-0.4643969	58	1.933		
DHR1:182832001	1	182832001	182834000	2000	1	5.41E-05	-0.3653539	24	1.2	AABR07005416.1;Ssty1	
DHR1:182845001	1	182845001	182850000	5000	1	3.41E-05	-0.4896444	65	1.3	AABR07005416.1;Ssty1	

DHR1:183067001	1	183067001	183070000	3000	1	8.37E-05	-0.9366932	29	0.967		
DHR1:186965001	1	186965001	186966000	1000	1	4.85E-05	0.4494236	10	1		
DHR1:190695001	1	190695001	190696000	1000	1	7.39E-05	-0.5968321	12	1.2	Vwa3a	Unknown
DHR1:195983001	1	195983001	195987000	4000	1	5.17E-05	0.2915997	34	0.85		
DHR1:201389001	1	201389001	201390000	1000	1	2.37E-06	-0.6160919	15	1.5	Btbd16	
DHR1:201788001	1	201788001	201789000	1000	1	7.81E-05	-0.6621506	13	1.3	LOC691970	
DHR1:204038001	1	204038001	204041000	3000	1	6.34E-05	-0.6086997	33	1.1	Cpxm2	Proteolysis
DHR1:205831001	1	205831001	205832000	1000	1	1.30E-05	-0.6238907	19	1.9	Dhx32	Transcription
DHR1:211005001	1	211005001	211006000	1000	1	5.63E-05	-0.5433621	6	0.6		
DHR1:211298001	1	211298001	211300000	2000	1	3.05E-05	-0.4841266	24	1.2		
DHR1:212090001	1	212090001	212092000	2000	1	8.81E-05	-0.4824067	19	0.95		
DHR1:212372001	1	212372001	212377000	5000	1	4.77E-05	-0.4195482	82	1.64	Zfp511	
DHR1:213035001	1	213035001	213036000	1000	1	4.37E-05	-0.5040178	6	0.6	Olr300	
DHR1:214920001	1	214920001	214922000	2000	1	8.28E-05	-0.6848126	11	0.55	Brsk2	Signaling
DHR1:217175001	1	217175001	217177000	2000	1	4.51E-05	-0.5391136	12	0.6	Shank2	Protein Binding
DHR1:220241001	1	220241001	220243000	2000	1	5.74E-05	-0.630322	24	1.2	mrpl11	Translation
DHR1:221008001	1	221008001	221011000	3000	1	4.02E-05	-0.9742718	131	4.367	Rela;Sipa1;Pcnx3	Transcription;Signaling
DHR1:221763001	1	221763001	221764000	1000	1	9.43E-05	-0.5602884	19	1.9	Pygm;Rasgrp2	Signaling
DHR1:224059001	1	224059001	224060000	1000	1	3.18E-05	-0.6027529	12	1.2		
DHR1:224233001	1	224233001	224235000	2000	1	6.92E-05	-0.4869263	36	1.8		
DHR1:227147001	1	227147001	227148000	1000	1	5.70E-05	-0.4803286	5	0.5	Ms4a8	Receptor
DHR1:237390001	1	237390001	237391000	1000	1	2.99E-05	0.4171137	3	0.3		
DHR1:241816001	1	241816001	241817000	1000	1	2.81E-05	-0.4608614	11	1.1	Fam189a2	Development
DHR1:253744001	1	253744001	253746000	2000	1	5.74E-05	-0.4747915	4	0.2		
DHR1:271975001	1	271975001	271976000	1000	1	6.38E-05	0.7066802	7	0.7		
DHR1:276206001	1	276206001	276207000	1000	1	7.26E-05	0.435647	4	0.4	Gucy2g	
DHR1:277477001	1	277477001	277479000	2000	1	1.92E-05	-0.7152662	22	1.1		
DHR2:5693001	2	5693001	5694000	1000	1	2.86E-05	-0.7009178	8	0.8		
DHR2:6115001	2	6115001	6117000	2000	1	9.21E-05	0.4632171	12	0.6		
DHR2:6311001	2	6311001	6312000	1000	1	6.69E-06	0.3895053	11	1.1		
DHR2:11682001	2	11682001	11683000	1000	1	6.30E-05	0.438652	10	1	Mef2c	Transcription
DHR2:11842001	2	11842001	11844000	2000	1	3.43E-05	0.4057026	19	0.95		
DHR2:11933001	2	11933001	11934000	1000	1	6.06E-05	0.3945933	8	0.8		
DHR2:15642001	2	15642001	15645000	3000	3	2.15E-07	-1.6856366	10	0.333		
DHR2:16014001	2	16014001	16020000	6000	1	1.48E-05	-0.5226858	58	0.967		
DHR2:21216001	2	21216001	21217000	1000	1	2.34E-05	-0.5923246	19	1.9		
DHR2:21898001	2	21898001	21899000	1000	1	3.64E-05	-0.4573789	6	0.6	Msh3	DNA Repair
DHR2:25908001	2	25908001	25909000	1000	1	5.55E-05	0.4632981	16	1.6	Arhgef28	Signaling
DHR2:26331001	2	26331001	26332000	1000	1	9.03E-05	0.4760357	11	1.1	Iqgap2	Signaling
DHR2:51316001	2	51316001	51317000	1000	1	7.09E-06	0.4101966	10	1		
DHR2:52518001	2	52518001	52520000	2000	1	1.31E-05	0.4706756	12	0.6	Zfp131;U6	Transcription
DHR2:52592001	2	52592001	52595000	3000	1	4.51E-05	0.5218366	18	0.6	AABR07008298.2	
DHR2:64444001	2	64444001	64446000	2000	1	7.64E-06	0.53287	2	0.1		
DHR2:67496001	2	67496001	67497000	1000	1	4.12E-06	0.3508236	10	1		
DHR2:77523001	2	77523001	77524000	1000	1	2.25E-05	0.4521311	11	1.1		
DHR2:77680001	2	77680001	77682000	2000	1	2.87E-05	0.520999	31	1.55	AABR07008898.1	
DHR2:82795001	2	82795001	82796000	1000	1	6.09E-06	0.5152154	4	0.4		
DHR2:86811001	2	86811001	86812000	1000	1	8.30E-05	0.4269401	4	0.4		
DHR2:87091001	2	87091001	87092000	1000	1	5.93E-05	0.5015157	2	0.2		
DHR2:89055001	2	89055001	89057000	2000	1	9.53E-05	0.4041715	13	0.65	Ralyl	Transcription
DHR2:94963001	2	94963001	94964000	1000	1	7.51E-05	0.4216411	10	1	AABR07009377.1	
DHR2:96654001	2	96654001	96658000	4000	1	4.79E-05	0.4425783	29	0.725	Pkia	Proteolysis
DHR2:97496001	2	97496001	97499000	3000	1	1.22E-05	0.4823117	17	0.567		
DHR2:97822001	2	97822001	97823000	1000	1	2.36E-05	0.4411373	3	0.3		
DHR2:103807001	2	103807001	103809000	2000	1	2.81E-07	0.5527064	18	0.9		
DHR2:106108001	2	106108001	106111000	3000	1	4.14E-05	0.4758681	13	0.433		
DHR2:108949001	2	108949001	108951000	2000	1	7.87E-05	-0.5250975	49	2.45		
DHR2:111473001	2	111473001	111474000	1000	1	4.90E-05	0.4232531	2	0.2	Nlgn1	Signaling
DHR2:117333001	2	117333001	117336000	3000	1	9.12E-05	0.4231882	26	0.867		
DHR2:123215001	2	123215001	123217000	2000	1	7.95E-05	0.482393	30	1.5		
DHR2:127929001	2	127929001	127930000	1000	1	8.22E-05	0.4629272	4	0.4	RGD1565989	
DHR2:139669001	2	139669001	139670000	1000	1	9.36E-05	0.4122752	8	0.8		
DHR2:140395001	2	140395001	140396000	1000	1	2.67E-06	-0.4828026	14	1.4	Elf2	Transcription
DHR2:145035001	2	145035001	145037000	2000	1	9.56E-05	0.3737275	13	0.65		
DHR2:156782001	2	156782001	156794000	12000	1	1.25E-06	-0.302873	144	1.2	AABR07011031.1	
DHR2:157031001	2	157031001	157036000	5000	1	1.02E-07	-0.6155275	91	1.82	AABR07011057.1	
DHR2:159162001	2	159162001	159166000	4000	1	7.88E-05	-0.4791516	39	0.975		
DHR2:159629001	2	159629001	159630000	1000	1	4.59E-05	-0.6076837	5	0.5		
DHR2:159859001	2	159859001	159865000	6000	1	1.69E-06	-0.7549089	66	1.1		

DHR2:159905001	2	159905001	159907000	2000	1	1.30E-06	-0.5604597	15	0.75		
DHR2:160085001	2	160085001	160090000	5000	1	9.87E-05	-0.8234083	50	1		
DHR2:160192001	2	160192001	160194000	2000	1	4.59E-05	-0.4647925	20	1		
DHR2:160196001	2	160196001	160197000	1000	1	4.96E-05	-0.7603028	11	1.1		
DHR2:160225001	2	160225001	160226000	1000	1	1.48E-05	-0.6043268	8	0.8		
DHR2:160258001	2	160258001	160259000	1000	1	1.80E-05	-0.5267023	10	1		
DHR2:160296001	2	160296001	160301000	5000	1	1.95E-05	-0.4270408	61	1.22	AABR07011278.1	
DHR2:160302001	2	160302001	160303000	1000	1	4.42E-05	-0.5125601	6	0.6	AABR07011278.1	
DHR2:160431001	2	160431001	160433000	2000	1	2.40E-05	-0.4737576	18	0.9		
DHR2:160474001	2	160474001	160481000	7000	1	8.91E-05	-0.4274013	104	1.486		
DHR2:160491001	2	160491001	160493000	2000	1	6.59E-05	-0.4189077	20	1		
DHR2:160717001	2	160717001	160724000	7000	1	7.02E-05	-0.440863	118	1.686		
DHR2:160780001	2	160780001	160783000	3000	1	1.30E-05	-0.5150712	51	1.7		
DHR2:161168001	2	161168001	161175000	7000	1	4.66E-05	-0.493407	72	1.029		
DHR2:161400001	2	161400001	161408000	8000	1	2.93E-06	-0.484603	103	1.288		
DHR2:161695001	2	161695001	161697000	2000	1	3.14E-05	-0.2939525	13	0.65		
DHR2:161970001	2	161970001	161972000	2000	1	1.14E-05	-0.5132922	24	1.2		
DHR2:162275001	2	162275001	162279000	4000	1	1.05E-05	-0.5053126	33	0.825		
DHR2:163065001	2	163065001	163069000	4000	2	5.20E-06	-0.6914427	68	1.7		
DHR2:163379001	2	163379001	163381000	2000	1	4.98E-05	-0.6238933	14	0.7		
DHR2:163523001	2	163523001	163528000	5000	2	2.93E-05	-0.4091288	60	1.2		
DHR2:163635001	2	163635001	163638000	3000	1	9.15E-06	-0.5289469	37	1.233		
DHR2:163950001	2	163950001	163956000	6000	1	5.85E-05	-0.4694837	43	0.717		
DHR2:168992001	2	168992001	168995000	3000	1	8.73E-05	0.4874857	16	0.533		
DHR2:170573001	2	170573001	170574000	1000	1	1.21E-05	0.4730109	5	0.5		
DHR2:175641001	2	175641001	175643000	2000	1	2.24E-05	0.3609948	17	0.85		
DHR2:184093001	2	184093001	184094000	1000	1	1.63E-05	0.4483411	10	1		
DHR2:188445001	2	188445001	188447000	2000	1	9.44E-05	-0.6233681	45	2.25	Pklr;Hcn3	Signaling;Receptor
DHR2:189317001	2	189317001	189318000	1000	1	6.19E-05	-0.5549083	22	2.2	Atp8b2;U1	EST
DHR2:196989001	2	196989001	196990000	1000	1	9.56E-06	-0.5124286	10	1		
DHR2:197049001	2	197049001	197051000	2000	1	3.09E-05	-0.3969513	28	1.4		
DHR2:197071001	2	197071001	197079000	8000	1	1.59E-05	-0.4464092	92	1.15		
DHR2:197590001	2	197590001	197595000	5000	1	6.05E-05	-0.3717147	58	1.16		
DHR2:201653001	2	201653001	201654000	1000	1	3.62E-05	-0.629658	7	0.7		
DHR2:201739001	2	201739001	201740000	1000	1	1.09E-05	-0.5948143	10	1		
DHR2:214822001	2	214822001	214824000	2000	1	1.08E-05	0.4451967	14	0.7		
DHR2:215241001	2	215241001	215243000	2000	1	1.70E-05	1.5707347	18	0.9		
DHR2:216938001	2	216938001	216939000	1000	1	1.87E-05	0.505761	12	1.2	Col11a1	Cytoskeleton
DHR2:222850001	2	222850001	222851000	1000	1	5.54E-05	0.4069	8	0.8		
DHR2:223341001	2	223341001	223342000	1000	1	1.37E-06	-1.1659085	2	0.2		
DHR2:227482001	2	227482001	227483000	1000	1	1.32E-05	0.4900968	4	0.4	Sec24d	Transport
DHR2:227581001	2	227581001	227582000	1000	1	6.24E-06	0.4621375	12	1.2	Mettl14;AABR07013207.1	Epigenetic
DHR2:231002001	2	231002001	231007000	5000	1	5.72E-05	0.430539	43	0.86	Camk2d	Signaling
DHR2:233581001	2	233581001	233582000	1000	1	5.42E-05	0.4520561	8	0.8		
DHR2:237140001	2	237140001	237141000	1000	1	6.91E-05	0.459437	14	1.4	Dkk2	
DHR2:245087001	2	245087001	245089000	2000	1	8.89E-05	0.4146709	17	0.85		
DHR2:251025001	2	251025001	251027000	2000	1	9.52E-05	0.443378	31	1.55	Odf2l	
DHR2:252763001	2	252763001	252764000	1000	1	7.53E-05	0.3748939	17	1.7	Ttll7	Cytoskeleton
DHR2:256407001	2	256407001	256408000	1000	1	1.75E-05	0.5906544	4	0.4		
DHR2:258686001	2	258686001	258687000	1000	1	7.31E-05	-0.4218567	21	2.1		
DHR2:261367001	2	261367001	261381000	14000	1	1.26E-05	-0.8037585	416	2.971	Fpgt	Signaling
DHR2:262403001	2	262403001	262406000	3000	1	6.05E-05	0.3826	20	0.667		
DHR2:262700001	2	262700001	262701000	1000	1	4.40E-05	0.4871765	8	0.8		
DHR2:264884001	2	264884001	264885000	1000	1	3.59E-05	0.404069	6	0.6	Lrrc40	Unknown
DHR3:4657001	3	4657001	4658000	1000	1	9.21E-07	-0.879207	23	2.3		
DHR3:4867001	3	4867001	4870000	3000	1	9.89E-05	-0.7009501	84	2.8	Med22;Rpl7a;SNORD24;SNORD36;Surf1;LOC100912042	Transcription;Translation;Development
DHR3:7124001	3	7124001	7128000	4000	1	8.21E-05	-0.7903759	74	1.85	Ralgds;Cel	Signaling;Metabolism
DHR3:7176001	3	7176001	7178000	2000	1	8.48E-05	-0.6393487	25	1.25	U6	
DHR3:9363001	3	9363001	9365000	2000	1	7.35E-05	-0.9752569	31	1.55	Fibcd1l1	
DHR3:16695001	3	16695001	16697000	2000	1	3.73E-06	0.3913182	7	0.35	AABR07051551.1	
DHR3:16758001	3	16758001	16762000	4000	1	9.73E-07	0.4685003	18	0.45	AABR07051551.1;AABR07051548.1;AABR07051548.2	
DHR3:16789001	3	16789001	16797000	8000	1	5.85E-05	0.4810613	25	0.312	AABR07051551.1	
DHR3:16828001	3	16828001	16834000	6000	2	1.84E-05	0.464546	18	0.3	AABR07051551.1	
DHR3:16872001	3	16872001	16875000	3000	1	3.57E-06	0.6837743	14	0.467	AABR07051551.1	
DHR3:16930001	3	16930001	16936000	6000	1	1.07E-06	0.4756443	27	0.45		
DHR3:16937001	3	16937001	16952000	15000	1	4.03E-05	0.4279688	72	0.48	AABR07051555.1	
DHR3:16954001	3	16954001	16959000	5000	1	7.23E-05	0.3858975	20	0.4	AABR07051555.1	

DHR3:16963001	3	16963001	16973000	10000	1	5.50E-05	0.4384363	45	0.45	AABR07051555.1	
DHR3:17086001	3	17086001	17094000	8000	1	1.43E-05	0.4757956	25	0.312	AABR07051562.1	
DHR3:21554001	3	21554001	21561000	7000	1	8.88E-05	0.4330728	48	0.686	AABR07051793.1	
DHR3:27147001	3	27147001	27149000	2000	1	9.16E-05	0.4622943	8	0.4		
DHR3:27712001	3	27712001	27713000	1000	1	2.90E-05	0.4847466	2	0.2		
DHR3:28117001	3	28117001	28119000	2000	1	6.94E-06	0.5692045	13	0.65		
DHR3:36777001	3	36777001	36779000	2000	1	1.39E-05	0.4587245	8	0.4		
DHR3:37461001	3	37461001	37462000	1000	1	3.78E-05	0.4993642	5	0.5	Nmi	Transcription
DHR3:41777001	3	41777001	41778000	1000	1	5.72E-05	0.4415542	9	0.9	5S_rRNA;AABR07052199.1	
DHR3:48477001	3	48477001	48480000	3000	1	3.05E-05	0.4259105	28	0.933	Fap	Protease
DHR3:53346001	3	53346001	53347000	1000	1	3.18E-05	0.4482839	5	0.5		
DHR3:64136001	3	64136001	64137000	1000	1	1.08E-05	0.4829277	8	0.8		
DHR3:70649001	3	70649001	70650000	1000	1	7.64E-06	0.4417925	2	0.2		
DHR3:71780001	3	71780001	71782000	2000	1	3.64E-05	0.4399612	16	0.8	Calcrl	Receptor
DHR3:71901001	3	71901001	71903000	2000	1	8.54E-05	-0.4660473	31	1.55	Tfpi	Signaling
DHR3:76030001	3	76030001	76032000	2000	1	6.11E-05	0.3976421	14	0.7	Olr596	Receptor
DHR3:78091001	3	78091001	78093000	2000	1	9.30E-05	0.4782952	10	0.5	Olr691	
DHR3:83416001	3	83416001	83417000	1000	1	1.15E-05	0.4465138	3	0.3		
DHR3:83945001	3	83945001	83949000	4000	1	9.52E-05	0.3851235	21	0.525		
DHR3:92734001	3	92734001	92735000	1000	1	2.80E-05	0.4284397	9	0.9	Cd44	
DHR3:96597001	3	96597001	96601000	4000	1	6.09E-05	-0.3619384	33	0.825		
DHR3:96611001	3	96611001	96613000	2000	1	7.89E-05	-0.3784708	28	1.4		
DHR3:96644001	3	96644001	96648000	4000	1	8.44E-05	-0.4114962	55	1.375		
DHR3:99614001	3	99614001	99615000	1000	1	1.36E-05	0.5373476	4	0.4		
DHR3:101535001	3	101535001	101537000	2000	1	2.08E-05	0.5090502	6	0.3	Fibin	
DHR3:102882001	3	102882001	102883000	1000	1	9.38E-05	0.4425503	4	0.4		
DHR3:131258001	3	131258001	131259000	1000	1	7.27E-05	0.4954607	2	0.2		
DHR3:133342001	3	133342001	133343000	1000	1	3.56E-05	-0.3229799	12	1.2		
DHR3:133495001	3	133495001	133501000	6000	1	4.11E-05	-0.4118417	101	1.683		
DHR3:133654001	3	133654001	133661000	7000	1	4.58E-05	-0.4246032	89	1.271		
DHR3:142686001	3	142686001	142687000	1000	1	9.88E-05	0.39429	7	0.7		
DHR3:143772001	3	143772001	143779000	7000	1	8.22E-05	-0.5222587	66	0.943	P22k15	
DHR3:150913001	3	150913001	150915000	2000	1	7.61E-07	-0.614538	37	1.85	Tp53inp2;Mir6334;Ncoa6	Transcription
DHR3:151170001	3	151170001	151171000	1000	1	7.98E-05	-0.5214297	7	0.7	Trpc4ap	Development
DHR3:153951001	3	153951001	153954000	3000	1	6.44E-05	-0.7115622	39	1.3	LOC100911217	
DHR3:160310001	3	160310001	160311000	1000	1	9.91E-05	-0.519222	12	1.2	Rims4	Signaling
DHR3:161432001	3	161432001	161434000	2000	1	4.25E-05	-1.0736514	105	5.25	Slc12a5	Metabolism
DHR3:163351001	3	163351001	163359000	8000	1	1.21E-05	-1.1101437	172	2.15	Prex1	
DHR3:164046001	3	164046001	164051000	5000	1	8.07E-05	-0.8208567	95	1.9	B4galt5	Metabolism
DHR4:11808001	4	11808001	11810000	2000	1	3.33E-05	0.4472086	11	0.55		
DHR4:22362001	4	22362001	22364000	2000	1	5.32E-05	0.4882796	19	0.95	Abcb1a	Transport
DHR4:33083001	4	33083001	33084000	1000	1	1.67E-05	-0.6162234	7	0.7		
DHR4:33129001	4	33129001	33130000	1000	1	1.80E-05	-0.5969516	8	0.8		
DHR4:34255001	4	34255001	34256000	1000	1	5.03E-05	0.4946941	4	0.4	Mios	
DHR4:44118001	4	44118001	44119000	1000	1	8.81E-05	0.474846	5	0.5	Tfec	Transcription
DHR4:44210001	4	44210001	44211000	1000	1	6.11E-05	0.4411344	14	1.4	AC127142.1	
DHR4:48100001	4	48100001	48101000	1000	1	8.04E-05	0.5078478	4	0.4		
DHR4:55095001	4	55095001	55097000	2000	1	6.26E-06	0.5310341	8	0.4	Grm8	Receptor
DHR4:61848001	4	61848001	61851000	3000	1	1.79E-05	-0.7593276	31	1.033	Akr1b7	Metabolism
DHR4:62200001	4	62200001	62201000	1000	1	2.77E-05	0.5633288	12	1.2	AC103335.1	
DHR4:65073001	4	65073001	65075000	2000	1	5.96E-05	-0.8501031	14	0.7	AABR07060261.1	
DHR4:73685001	4	73685001	73686000	1000	1	6.03E-05	0.4746593	1	0.1		
DHR4:82985001	4	82985001	82986000	1000	1	7.97E-05	0.46544	3	0.3	Jazf1	
DHR4:84109001	4	84109001	84110000	1000	1	4.12E-05	-0.5754842	15	1.5	Cpvl	Protease
DHR4:85805001	4	85805001	85806000	1000	1	2.84E-05	0.4828992	9	0.9		
DHR4:86024001	4	86024001	86025000	1000	1	3.59E-05	0.4723127	5	0.5		
DHR4:86584001	4	86584001	86585000	1000	1	8.48E-05	0.4404349	4	0.4	Pde1c	Metabolism
DHR4:87004001	4	87004001	87006000	2000	1	4.79E-05	-0.538979	19	0.95		
DHR4:90064001	4	90064001	90065000	1000	1	7.65E-07	0.6693546	5	0.5	AABR07060700.1	
DHR4:91481001	4	91481001	91482000	1000	1	9.01E-05	0.4228645	7	0.7	Ccser1	
DHR4:92223001	4	92223001	92224000	1000	1	8.48E-05	0.4175083	4	0.4		
DHR4:93295001	4	93295001	93297000	2000	1	4.62E-07	0.5435527	8	0.4		
DHR4:98686001	4	98686001	98687000	1000	1	8.06E-05	0.4650443	12	1.2	Eif2ak3	Signaling
DHR4:111172001	4	111172001	111174000	2000	1	1.27E-05	0.5430612	8	0.4	Lrrtm4	Receptor
DHR4:119871001	4	119871001	119872000	1000	1	1.07E-05	-0.4707475	9	0.9	Copg1;AC111943.2	
DHR4:122013001	4	122013001	122015000	2000	1	7.42E-05	-0.491223	12	0.6	Vom1r100	
DHR4:127688001	4	127688001	127690000	2000	1	9.04E-05	0.3367385	21	1.05	Suclg2	Metabolism
DHR4:130423001	4	130423001	130424000	1000	1	3.95E-06	0.5176893	16	1.6	Mitf	Transcription

DHR4:133860001	4	133860001	133862000	2000	1	2.72E-05	-0.4862948	16	0.8	Pdzrn3	Transcription
DHR4:134809001	4	134809001	134811000	2000	1	2.93E-05	0.6007197	9	0.45	Cntn3	Cytoskeleton
DHR4:135059001	4	135059001	135061000	2000	1	9.88E-05	0.4981595	10	0.5	Cntn3	Cytoskeleton
DHR4:141430001	4	141430001	141431000	1000	1	1.95E-05	0.5279067	2	0.2		
DHR4:143038001	4	143038001	143039000	1000	1	6.23E-06	0.4918151	8	0.8	Grm7	Receptor
DHR4:160430001	4	160430001	160431000	1000	1	6.63E-05	0.3640669	9	0.9	AABR07062069.1	
DHR4:162680001	4	162680001	162682000	2000	1	6.18E-05	0.6891031	11	0.55	AABR07062152.1	
DHR4:166637001	4	166637001	166640000	3000	1	7.08E-05	-0.6280936	91	3.033		
DHR5:19925001	5	19925001	19926000	1000	1	1.67E-05	0.4899086	16	1.6	Tox	Immune
DHR5:30540001	5	30540001	30541000	1000	1	7.40E-05	0.4201931	3	0.3		
DHR5:36707001	5	36707001	36708000	1000	1	8.95E-05	0.5451136	8	0.8		
DHR5:42932001	5	42932001	42934000	2000	1	5.82E-05	0.4284425	8	0.4		
DHR5:43372001	5	43372001	43373000	1000	1	1.97E-06	0.557317	6	0.6		
DHR5:45993001	5	45993001	45997000	4000	1	6.16E-06	0.5181887	32	0.8		
DHR5:50247001	5	50247001	50248000	1000	1	4.11E-05	0.4541683	10	1	Smim8;AABR07047843.1	
DHR5:50388001	5	50388001	50390000	2000	1	4.88E-05	0.4868778	11	0.55	Cga	Hormone
DHR5:51622001	5	51622001	51623000	1000	1	2.92E-05	0.5729321	8	0.8		
DHR5:52629001	5	52629001	52630000	1000	1	8.55E-05	0.5052012	7	0.7		
DHR5:70083001	5	70083001	70084000	1000	1	4.78E-05	0.3751129	4	0.4		
DHR5:70695001	5	70695001	70696000	1000	1	4.34E-05	0.4373523	16	1.6		
DHR5:82986001	5	82986001	82987000	1000	1	6.96E-05	0.4967419	4	0.4		
DHR5:83867001	5	83867001	83868000	1000	1	7.84E-06	0.5012364	3	0.3		
DHR5:87257001	5	87257001	87266000	9000	3	7.41E-06	0.568506	45	0.5		
DHR5:92114001	5	92114001	92115000	1000	1	3.60E-05	0.4012043	13	1.3		
DHR5:92339001	5	92339001	92340000	1000	1	8.40E-06	0.4332458	8	0.8		
DHR5:95362001	5	95362001	95364000	2000	1	8.46E-05	0.4285867	13	0.65	AABR07048878.1	
DHR5:96462001	5	96462001	96464000	2000	1	1.06E-05	0.4342535	8	0.4		
DHR5:99356001	5	99356001	99357000	1000	1	5.94E-05	0.5336994	6	0.6	AABR07049002.1	
DHR5:100348001	5	100348001	100350000	2000	1	1.77E-05	0.4863453	13	0.65		
DHR5:106845001	5	106845001	106847000	2000	1	8.52E-05	0.4471347	12	0.6		
DHR5:109224001	5	109224001	109226000	2000	1	4.20E-05	0.4126411	12	0.6		
DHR5:121137001	5	121137001	121141000	4000	1	8.95E-05	-0.3522804	41	1.025		
DHR5:121185001	5	121185001	121203000	18000	1	7.24E-05	-0.3676303	197	1.094		
DHR5:130842001	5	130842001	130843000	1000	1	5.35E-05	0.4272152	6	0.6		
DHR5:131468001	5	131468001	131469000	1000	1	6.70E-05	-0.8089603	6	0.6		
DHR5:135907001	5	135907001	135909000	2000	1	3.51E-06	-0.5307256	25	1.25	Eif2b3	Translation
DHR5:142556001	5	142556001	142559000	3000	1	8.78E-05	-0.7324155	105	3.5	AABR07049886.1	
DHR5:142599001	5	142599001	142602000	3000	1	7.54E-05	-0.6807891	55	1.833	AABR07049886.3	
DHR5:143075001	5	143075001	143076000	1000	1	2.03E-05	-0.5431543	11	1.1	Snip1;Meaf6	
DHR5:146425001	5	146425001	146427000	2000	1	5.35E-05	-0.5517965	25	1.25	Csmd2	Unknown
DHR5:149804001	5	149804001	149806000	2000	1	5.36E-05	-0.5226802	20	1		
DHR5:167287001	5	167287001	167290000	3000	1	1.41E-05	-0.4479197	115	3.833	Eno1	Metabolism
DHR5:169684001	5	169684001	169687000	3000	1	1.33E-05	-0.8019627	37	1.233	Nphp4	Development
DHR6:2787001	6	2787001	2790000	3000	1	9.37E-05	0.3873379	35	1.167	U6	
DHR6:5322001	6	5322001	5324000	2000	1	1.17E-05	0.5168173	11	0.55		
DHR6:28444001	6	28444001	28445000	1000	1	5.55E-06	0.4646232	5	0.5	Efr3b	
DHR6:30594001	6	30594001	30595000	1000	1	1.17E-05	-0.2811586	8	0.8		
DHR6:30646001	6	30646001	30649000	3000	1	7.41E-06	-0.6661034	30	1	AABR07063424.1;AABR07063425.3;AABR07063425.2;AABR07063425.1	
DHR6:31043001	6	31043001	31073000	30000	6	4.68E-06	-0.7624002	709	2.363	AABR07063462.1	
DHR6:39266001	6	39266001	39268000	2000	1	9.83E-05	0.4276007	24	1.2		
DHR6:45050001	6	45050001	45051000	1000	1	9.02E-05	0.461322	1	0.1		
DHR6:45406001	6	45406001	45407000	1000	1	7.71E-05	0.4585651	4	0.4		
DHR6:49588001	6	49588001	49590000	2000	1	8.04E-05	0.3791156	6	0.3		
DHR6:58465001	6	58465001	58466000	1000	1	7.58E-05	0.4305306	9	0.9	Etv1	Transcription
DHR6:61415001	6	61415001	61418000	3000	1	8.10E-05	0.4286223	23	0.767	Lrrn3	Receptor
DHR6:63728001	6	63728001	63730000	2000	1	9.15E-05	0.4818473	14	0.7		
DHR6:70268001	6	70268001	70269000	1000	1	3.77E-05	0.6105049	6	0.6	AABR07064231.1	
DHR6:74317001	6	74317001	74319000	2000	1	6.00E-05	0.4619317	33	1.65		
DHR6:74542001	6	74542001	74543000	1000	1	5.47E-05	-0.4779822	22	2.2		
DHR6:88264001	6	88264001	88266000	2000	1	6.09E-05	0.510246	11	0.55		
DHR6:89405001	6	89405001	89406000	1000	1	7.00E-05	0.4274346	6	0.6		
DHR6:90266001	6	90266001	90268000	2000	1	8.52E-05	0.5163847	7	0.35	AABR07064681.1	
DHR6:117220001	6	117220001	117221000	1000	1	9.31E-05	0.4385891	11	1.1		
DHR6:127103001	6	127103001	127106000	3000	1	3.48E-05	-0.6583814	34	1.133	Prima1	
DHR6:127582001	6	127582001	127583000	1000	1	5.92E-05	-0.3903742	8	0.8	AC094636.1	
DHR6:133110001	6	133110001	133111000	1000	1	3.15E-05	0.5287185	9	0.9		
DHR6:138140001	6	138140001	138143000	3000	1	1.68E-05	-0.700938	36	1.2	Ighm	
DHR6:142263001	6	142263001	142264000	1000	1	3.73E-05	-0.7641878	8	0.8		

DHR6:145839001	6	145839001	145840000	1000	1	6.30E-05	0.4797017	6	0.6	Dnah11	Cytoskeleton
DHR7:705001	7	705001	707000	2000	1	6.05E-05	-0.2083215	17	0.85	5_8S_rRNA	
DHR7:925001	7	925001	926000	1000	1	3.61E-05	-0.4288739	11	1.1		
DHR7:1609001	7	1609001	1611000	2000	1	2.79E-05	-0.3758416	31	1.55		
DHR7:2999001	7	2999001	3001000	2000	1	6.94E-05	-0.6182885	32	1.6	Erbb3	Transcription
DHR7:3113001	7	3113001	3114000	1000	1	7.63E-05	-0.5304993	10	1	Rab5b	Signaling
DHR7:5286001	7	5286001	5288000	2000	1	4.59E-05	-0.8066878	43	2.15		
DHR7:10257001	7	10257001	10258000	1000	1	8.94E-05	-0.9859319	8	0.8	AABR07055812.1	
DHR7:14879001	7	14879001	14881000	2000	1	9.87E-05	-0.6238619	42	2.1	Cyp4f40	Metabolism
DHR7:17262001	7	17262001	17263000	1000	1	5.78E-05	-0.5243742	10	1		
DHR7:25323001	7	25323001	25325000	2000	1	1.05E-05	-0.576423	30	1.5		
DHR7:40063001	7	40063001	40069000	6000	3	4.58E-09	1.0561414	49	0.817		
DHR7:43279001	7	43279001	43280000	1000	1	6.52E-06	0.3927491	13	1.3		
DHR7:47527001	7	47527001	47528000	1000	1	5.64E-05	0.4786199	14	1.4	Tmtc2	Unknown
DHR7:48602001	7	48602001	48604000	2000	1	4.86E-05	0.464714	5	0.25	AABR07056953.1	
DHR7:48694001	7	48694001	48696000	2000	1	4.00E-06	0.5567858	22	1.1		
DHR7:50495001	7	50495001	50496000	1000	1	6.06E-05	0.4496714	6	0.6	Syt1	Transport
DHR7:62581001	7	62581001	62582000	1000	1	5.42E-05	-0.5114173	12	1.2		
DHR7:62803001	7	62803001	62804000	1000	1	6.43E-05	-0.4558493	11	1.1		
DHR7:68498001	7	68498001	68500000	2000	1	3.63E-05	0.4845584	13	0.65	Slc16a7	Metabolism
DHR7:71706001	7	71706001	71707000	1000	1	6.69E-06	0.5170298	5	0.5	Cpq	Metabolism
DHR7:75988001	7	75988001	75989000	1000	1	8.22E-05	-0.5079763	13	1.3		
DHR7:79655001	7	79655001	79659000	4000	1	1.14E-05	0.5088216	28	0.7	Zfpm2	Transcription
DHR7:80162001	7	80162001	80163000	1000	1	2.90E-05	0.4498009	8	0.8	AABR07057611.1	
DHR7:87084001	7	87084001	87086000	2000	1	6.54E-05	0.4075046	7	0.35	AABR07057765.1	
DHR7:97054001	7	97054001	97055000	1000	1	7.58E-05	0.4769308	2	0.2	Slc22a22	
DHR7:98133001	7	98133001	98134000	1000	1	4.86E-05	-0.522209	14	1.4	AABR07058011.1	
DHR7:99469001	7	99469001	99470000	1000	1	8.03E-05	-0.5462634	13	1.3		
DHR7:105533001	7	105533001	105534000	1000	1	1.43E-07	0.4623827	4	0.4	Adcy8	Signaling
DHR7:106683001	7	106683001	106685000	2000	1	7.69E-05	0.4068159	11	0.55	Hhla1	
DHR7:108569001	7	108569001	108570000	1000	1	5.01E-05	0.4235393	5	0.5		
DHR7:116683001	7	116683001	116684000	1000	1	8.33E-05	-0.5437512	10	1	Gli4;Top1mt	Transcription
DHR7:119263001	7	119263001	119264000	1000	1	1.89E-05	-0.5221224	5	0.5	Cacng2	Transport
DHR7:121057001	7	121057001	121059000	2000	1	5.62E-05	-1.0542493	110	5.5	Cbx6	Transcription
DHR7:121637001	7	121637001	121638000	1000	1	2.61E-05	-0.8224591	9	0.9		
DHR7:122041001	7	122041001	122042000	1000	1	8.22E-06	-0.5272261	8	0.8	Tnrc6b	Apoptosis
DHR7:122631001	7	122631001	122632000	1000	1	6.93E-05	-0.521408	9	0.9	St13;Xpnpep3	Cytoskeleton;Protease
DHR7:123198001	7	123198001	123199000	1000	1	7.64E-05	-0.4586074	8	0.8	Pmm1	Metabolism
DHR7:123676001	7	123676001	123678000	2000	1	1.16E-05	-0.5591298	16	0.8	Tcf20	Transcription
DHR7:130286001	7	130286001	130290000	4000	1	7.06E-05	-0.6162703	128	3.2	Sbf1;Adm2	Signaling
DHR7:139355001	7	139355001	139359000	4000	2	3.51E-05	-0.6884321	84	2.1	Vdr	Receptor
DHR7:140322001	7	140322001	140324000	2000	1	8.87E-05	-0.824207	49	2.45	Cacnb3;Ddx23	Signaling;Transcription
DHR7:142131001	7	142131001	142132000	1000	1	4.49E-05	-0.9862821	75	7.5	Csrnp2;Tfcp2	Transcription
DHR7:142364001	7	142364001	142368000	4000	1	5.35E-05	-0.5615724	102	2.55		
DHR8:1339001	8	1339001	1340000	1000	1	2.70E-05	-0.6788385	8	0.8		
DHR8:10878001	8	10878001	10879000	1000	1	3.34E-05	-0.6179736	1	0.1		
DHR8:15666001	8	15666001	15670000	4000	1	8.96E-05	-0.5077667	21	0.525		
DHR8:49734001	8	49734001	49737000	3000	1	2.63E-05	-0.5278753	36	1.2	Dscaml1	Development
DHR8:51235001	8	51235001	51238000	3000	1	2.22E-05	-0.5915865	24	0.8		
DHR8:52336001	8	52336001	52337000	1000	1	9.90E-05	-0.5196749	13	1.3		
DHR8:55060001	8	55060001	55062000	2000	1	9.77E-05	-0.4789422	19	0.95	Nkapd1;Pih1d2;Dlat	Signaling;Metabolism
DHR8:57983001	8	57983001	57984000	1000	1	6.40E-06	-0.5721772	27	2.7	Poglut3;RGD1311251	EST
DHR8:61267001	8	61267001	61269000	2000	1	3.79E-05	-0.7419111	21	1.05	Lingo1	Development
DHR8:64422001	8	64422001	64423000	1000	1	9.96E-05	-0.518883	10	1	Celf6	Transcription
DHR8:66946001	8	66946001	66948000	2000	1	3.05E-05	-0.4807054	18	0.9	Paqr5	Receptor
DHR8:68026001	8	68026001	68029000	3000	1	9.10E-05	-0.6185795	57	1.9	U6;Skor1	
DHR8:107338001	8	107338001	107339000	1000	1	7.44E-05	-0.6335326	18	1.8	Pik3cb	Signaling
DHR8:112479001	8	112479001	112480000	1000	1	9.01E-05	-0.4275569	10	1	AABR07071374.1	
DHR8:117132001	8	117132001	117134000	2000	1	8.87E-05	-0.4873862	28	1.4	Usp4	Proteolysis
DHR8:117179001	8	117179001	117181000	2000	1	1.20E-05	-0.5797603	30	1.5	Usp4;Ccdc36;LOC498675	Proteolysis
DHR8:118253001	8	118253001	118255000	2000	1	8.71E-05	-0.4795587	30	1.5	Smarcc1	Transcription
DHR8:123366001	8	123366001	123367000	1000	1	7.12E-05	-0.4802265	11	1.1	Stt3b	
DHR8:126928001	8	126928001	126929000	1000	1	4.75E-05	-0.4272702	9	0.9		
DHR9:1859001	9	1859001	1861000	2000	1	2.50E-05	-0.6654662	29	1.45	Plcl2	Signaling
DHR9:7563001	9	7563001	7565000	2000	1	2.14E-06	-0.4747437	14	0.7		
DHR9:11250001	9	11250001	11251000	1000	1	1.58E-05	-0.6368263	13	1.3	Uxs1	Metabolism
DHR9:11871001	9	11871001	11874000	3000	1	9.48E-05	-0.3840806	27	0.9		
DHR9:13922001	9	13922001	13924000	2000	1	9.33E-06	-0.6580251	31	1.55		

DHR9:18997001	9	18997001	18999000	2000	1	1.37E-05	0.4327435	19	0.95		
DHR9:31839001	9	31839001	31840000	1000	1	2.22E-05	0.4696654	5	0.5	Adgrb3	
DHR9:32190001	9	32190001	32191000	1000	1	7.80E-05	0.3899967	5	0.5		
DHR9:38391001	9	38391001	38392000	1000	1	8.78E-05	-0.4426665	8	0.8	Zfp451	Transcription
DHR9:47876001	9	47876001	47877000	1000	1	6.06E-05	0.4695414	7	0.7		
DHR9:48131001	9	48131001	48133000	2000	1	3.94E-05	0.4305734	14	0.7		
DHR9:51240001	9	51240001	51241000	1000	1	7.45E-05	-0.5671291	9	0.9		
DHR9:69147001	9	69147001	69149000	2000	1	9.40E-05	0.4621549	18	0.9	Pard3b	Cell Junction
DHR9:101228001	9	101228001	101240000	12000	1	9.32E-05	0.5663035	88	0.733	Vom1r64	
DHR9:103947001	9	103947001	103948000	1000	1	2.44E-05	0.5987049	7	0.7		
DHR9:105097001	9	105097001	105098000	1000	1	4.09E-05	0.440047	5	0.5		
DHR9:105864001	9	105864001	105866000	2000	1	4.39E-05	0.4695085	21	1.05		
DHR9:121916001	9	121916001	121918000	2000	1	4.40E-06	0.4641558	13	0.65	Tyms	Metabolism
DHR10:419001	10	419001	424000	5000	1	4.25E-05	-0.4563139	44	0.88		
DHR10:6969001	10	6969001	6970000	1000	1	4.59E-05	-0.4775991	13	1.3	Usp7	Protease
DHR10:15508001	10	15508001	15509000	1000	1	8.59E-05	-0.5654982	19	1.9	Mrpl28	Transcription
DHR10:21486001	10	21486001	21487000	1000	1	2.75E-06	-0.4837285	16	1.6		
DHR10:38562001	10	38562001	38563000	1000	1	6.77E-05	-0.5400753	27	2.7		
DHR10:38829001	10	38829001	38833000	4000	1	6.54E-05	-0.746952	44	1.1	Shroom1;AC114017.1;Sowaha	Receptor
DHR10:39222001	10	39222001	39223000	1000	1	1.40E-07	-1.062553	5	0.5	Slc22a5	Transport
DHR10:43581001	10	43581001	43582000	1000	1	7.14E-05	-0.4658037	13	1.3	Gemin5	
DHR10:44385001	10	44385001	44386000	1000	1	7.47E-05	-0.7370187	11	1.1	Olr1436	Receptor
DHR10:44498001	10	44498001	44499000	1000	1	1.23E-05	-0.5301301	5	0.5	LOC501698	
DHR10:44629001	10	44629001	44631000	2000	1	3.11E-07	-0.6202538	9	0.45	Olr1448	Receptor
DHR10:44720001	10	44720001	44723000	3000	1	6.35E-05	-0.4911905	13	0.433	Olr1454;Olr1456	Receptor
DHR10:45312001	10	45312001	45313000	1000	1	6.56E-05	-0.5336243	14	1.4	Trim17;Trim11	Metabolism
DHR10:47253001	10	47253001	47256000	3000	1	3.66E-06	-0.4907386	35	1.167		
DHR10:49469001	10	49469001	49470000	1000	1	4.93E-05	-0.5082416	12	1.2	Tekt3	Cytoskeleton
DHR10:53364001	10	53364001	53366000	2000	1	1.62E-06	-0.6091009	21	1.05		
DHR10:55053001	10	55053001	55055000	2000	1	8.23E-05	-0.6055647	20	1	Pik3r5	Signaling
DHR10:55394001	10	55394001	55397000	3000	1	6.58E-05	-0.6031913	53	1.767	Myh10	Cytoskeleton
DHR10:55712001	10	55712001	55715000	3000	1	4.07E-05	-0.69223	54	1.8	Hes7;Aloxe3	Transcription
DHR10:55940001	10	55940001	55942000	2000	1	5.52E-06	-0.7240275	114	5.7	Kcnab3;RGD1563441;Chd3;Gm22442	Transport;Epigenetic
DHR10:59482001	10	59482001	59485000	3000	1	3.82E-05	-0.4798645	36	1.2	Zzef1	Transcription
DHR10:63956001	10	63956001	63958000	2000	1	5.51E-05	-0.576017	15	0.75	Doc2b;U6	Metabolism
DHR10:65274001	10	65274001	65275000	1000	1	5.98E-05	-0.6268093	10	1	Eral1	Signaling
DHR10:75404001	10	75404001	75406000	2000	1	6.71E-05	-0.7023983	25	1.25		
DHR10:82904001	10	82904001	82912000	8000	1	3.96E-05	-0.6698408	123	1.538		
DHR10:84094001	10	84094001	84095000	1000	1	6.30E-05	-0.5190813	4	0.4		
DHR10:85715001	10	85715001	85719000	4000	1	2.21E-07	-0.8737201	64	1.6	Cwc25;LOC691189;Rpl23;Snora21	Translation;Unknown
DHR10:88185001	10	88185001	88188000	3000	1	9.43E-05	-0.6738424	62	2.067		
DHR10:88825001	10	88825001	88827000	2000	1	2.17E-05	-0.5141787	21	1.05	Stat3	Transcription
DHR10:94674001	10	94674001	94677000	3000	1	5.73E-05	-0.5654894	21	0.7	Ern1	Signaling
DHR10:95068001	10	95068001	95071000	3000	1	1.45E-06	-0.6297526	24	0.8	Smurf2	Metabolism
DHR10:95439001	10	95439001	95441000	2000	1	7.94E-05	-0.4164557	35	1.75	AABR07030603.2;AABR07030603.1	
DHR10:100925001	10	100925001	100926000	1000	1	7.14E-05	-0.518516	10	1		
DHR10:102387001	10	102387001	102391000	4000	1	4.16E-05	-0.793816	95	2.375	Sdk2	Development
DHR10:104749001	10	104749001	104750000	1000	1	5.34E-05	-0.8365907	18	1.8	Acox1;AABR07072203.1	Metabolism
DHR10:107940001	10	107940001	107944000	4000	1	4.78E-05	-0.8695367	116	2.9		
DHR10:110976001	10	110976001	110977000	1000	1	2.91E-05	-0.5075309	7	0.7		
DHR11:12081001	11	12081001	12083000	2000	1	4.13E-05	0.4357056	15	0.75		
DHR11:14551001	11	14551001	14552000	1000	1	2.99E-05	0.4373175	13	1.3	AABR07033271.1	
DHR11:15710001	11	15710001	15711000	1000	1	9.28E-05	0.4188188	5	0.5		
DHR11:16481001	11	16481001	16489000	8000	5	1.63E-07	-0.9912189	60	0.75	U6	
DHR11:23439001	11	23439001	23441000	2000	1	5.38E-05	0.47611	7	0.35		
DHR11:27425001	11	27425001	27426000	1000	1	9.82E-05	-0.6236373	13	1.3		
DHR11:28203001	11	28203001	28206000	3000	1	5.98E-06	0.4680375	30	1		
DHR11:35462001	11	35462001	35463000	1000	1	4.40E-05	0.4163733	8	0.8		
DHR11:38022001	11	38022001	38029000	7000	2	1.55E-05	-0.8600918	113	1.614	Mx2	Immune
DHR11:38862001	11	38862001	38864000	2000	1	6.27E-06	-0.5679126	12	0.6		
DHR11:38921001	11	38921001	38922000	1000	1	3.82E-05	-0.4540341	6	0.6		
DHR11:39067001	11	39067001	39069000	2000	1	2.12E-05	-0.322118	15	0.75		
DHR11:39126001	11	39126001	39130000	4000	1	1.61E-05	-0.2886679	44	1.1		
DHR11:39190001	11	39190001	39191000	1000	1	4.42E-07	-0.3900751	13	1.3	5_8S_rRNA	
DHR11:47698001	11	47698001	47700000	2000	1	1.26E-05	0.4717327	11	0.55		
DHR11:47783001	11	47783001	47785000	2000	1	1.21E-05	0.4846971	19	0.95	AABR07034008.1	
DHR11:50106001	11	50106001	50107000	1000	1	9.91E-05	0.4210059	8	0.8		
DHR11:54486001	11	54486001	54487000	1000	1	4.23E-05	0.4825316	4	0.4		

DHR11:55046001	11	55046001	55047000	1000	1	7.48E-06	0.4529118	13	1.3	Morc1	
DHR11:58608001	11	58608001	58610000	2000	1	1.33E-06	0.5266918	11	0.55		
DHR11:60426001	11	60426001	60427000	1000	1	9.73E-06	0.4893238	12	1.2		
DHR11:63230001	11	63230001	63233000	3000	1	9.66E-05	0.434361	16	0.533	AABR07034295.1	
DHR11:66658001	11	66658001	66659000	1000	1	7.39E-05	0.5059203	9	0.9	Polq	Transcription
DHR11:72413001	11	72413001	72414000	1000	1	9.37E-05	0.5193098	6	0.6		
DHR11:72461001	11	72461001	72462000	1000	1	4.72E-05	-0.4937094	17	1.7		
DHR11:80625001	11	80625001	80626000	1000	1	5.05E-05	0.4434784	6	0.6	AABR07034598.1	
DHR11:83396001	11	83396001	83397000	1000	1	8.80E-05	-0.5468358	9	0.9		
DHR11:87189001	11	87189001	87190000	1000	1	3.38E-05	-0.9171114	6	0.6		
DHR12:3109001	12	3109001	3111000	2000	1	2.53E-06	-0.6049028	12	0.6		
DHR12:3137001	12	3137001	3148000	11000	1	4.83E-05	-0.3221349	136	1.236	AABR07035005.1	
DHR12:4751001	12	4751001	4753000	2000	1	8.88E-05	-0.5145101	17	0.85	AABR07035107.1	
DHR12:5888001	12	5888001	5889000	1000	1	2.74E-06	-0.6444191	11	1.1		
DHR12:7115001	12	7115001	7117000	2000	1	5.34E-05	-0.7807133	29	1.45	Metazoa_SRP;AABR07035218.2	
DHR12:7118001	12	7118001	7121000	3000	1	8.73E-05	-0.6834745	69	2.3	Metazoa_SRP;AABR07035218.2	
DHR12:10359001	12	10359001	10364000	5000	1	4.19E-05	-0.6356062	111	2.22	Wasf3;AABR07035320.3;AABR07035320.2	Cytoskeleton
DHR12:10491001	12	10491001	10493000	2000	1	1.29E-05	-0.6062598	28	1.4	AABR07035338.1	
DHR12:11390001	12	11390001	11392000	2000	1	2.10E-05	-0.590713	28	1.4	Kpna7	Unknown
DHR12:14154001	12	14154001	14160000	6000	1	7.24E-05	-0.6132519	96	1.6	Radil;AABR07035428.2;AABR07035428.1	Signaling
DHR12:16082001	12	16082001	16087000	5000	1	7.29E-05	-1.0372341	147	2.94	Ttyh3	Unknown
DHR12:20660001	12	20660001	20668000	8000	1	6.05E-05	0.644482	39	0.487	RGD1561730;RGD1560281	Immune
DHR12:23325001	12	23325001	23327000	2000	1	3.24E-06	-0.7390164	19	0.95	Cux1	Development
DHR12:23541001	12	23541001	23543000	2000	1	5.06E-05	-0.5697154	29	1.45	Prkrip1;Orai2	Proteolysis;Metabolism
DHR12:24482001	12	24482001	24484000	2000	1	8.00E-05	-0.6291315	42	2.1	Fzd9;Baz1b	Signaling;Metabolism
DHR12:25478001	12	25478001	25480000	2000	1	2.99E-05	-0.6334276	53	2.65	Gtf2i	Transcription
DHR12:28906001	12	28906001	28907000	1000	1	8.50E-05	-0.4530472	12	1.2	AABR07035946.1	
DHR12:30747001	12	30747001	30748000	1000	1	9.02E-05	-0.8228048	25	2.5	Sfswap	
DHR12:31530001	12	31530001	31534000	4000	1	4.85E-05	-0.5799978	60	1.5	Rimbp2	Unknown
DHR12:35041001	12	35041001	35043000	2000	1	3.72E-05	-0.702361	13	0.65		
DHR12:35125001	12	35125001	35128000	3000	1	8.92E-05	-0.5533903	32	1.067		
DHR12:35253001	12	35253001	35256000	3000	1	8.76E-07	-0.6877609	25	0.833		
DHR12:35265001	12	35265001	35277000	12000	1	1.00E-05	-0.4522738	132	1.1		
DHR12:35508001	12	35508001	35511000	3000	1	5.25E-05	-0.4899021	31	1.033		
DHR12:35576001	12	35576001	35578000	2000	1	2.31E-05	-0.6613287	20	1		
DHR12:36042001	12	36042001	36059000	17000	1	3.93E-06	-0.638846	244	1.435	Tmem132b	Unknown
DHR12:36064001	12	36064001	36066000	2000	1	9.28E-05	-0.4918152	24	1.2	Tmem132b	Unknown
DHR12:36138001	12	36138001	36139000	1000	1	3.24E-05	-0.4757575	12	1.2	Tmem132b	Unknown
DHR12:36550001	12	36550001	36553000	3000	1	1.13E-05	-0.6905297	46	1.533	Aacs	Metabolism
DHR12:36869001	12	36869001	36874000	5000	1	4.46E-05	-0.6175342	247	4.94	Ncor2	Transcription
DHR12:37660001	12	37660001	37663000	3000	1	4.16E-05	-0.4847651	46	1.533	7SK;Cdk2ap1	Transcription
DHR12:38039001	12	38039001	38045000	6000	1	4.16E-05	-0.8654843	98	1.633	Hip1r	Cytoskeleton
DHR12:38402001	12	38402001	38404000	2000	1	5.27E-05	-0.5235437	23	1.15	Clip1	Cytoskeleton
DHR12:40977001	12	40977001	40978000	1000	1	1.28E-05	-0.6293011	11	1.1	AABR07036376.1	
DHR12:46704001	12	46704001	46710000	6000	1	6.12E-05	-0.6575805	137	2.283	Bicdl1;Rab35;AC123425.1	Signaling
DHR12:49168001	12	49168001	49172000	4000	1	2.65E-05	-0.5594818	37	0.925		
DHR12:49173001	12	49173001	49178000	5000	1	7.50E-05	-0.7003741	66	1.32		
DHR12:49733001	12	49733001	49734000	1000	1	8.29E-07	-0.531855	15	1.5	Grk3	
DHR12:50773001	12	50773001	50774000	1000	1	9.15E-05	-0.682476	28	2.8		
DHR12:51573001	12	51573001	51574000	1000	1	2.87E-05	-0.5586989	17	1.7	Ttc28	Unknown
DHR13:4805001	13	4805001	4806000	1000	1	8.43E-05	-0.5168377	6	0.6		
DHR13:5211001	13	5211001	5212000	1000	1	1.80E-05	-0.6242111	2	0.2		
DHR13:7976001	13	7976001	7977000	1000	1	2.69E-05	-0.4673386	5	0.5		
DHR13:9435001	13	9435001	9436000	1000	1	9.45E-05	-0.5244961	3	0.3		
DHR13:12237001	13	12237001	12238000	1000	1	5.60E-05	-0.431116	9	0.9		
DHR13:25745001	13	25745001	25748000	3000	1	4.88E-05	0.4817168	17	0.567	Relch	
DHR13:34115001	13	34115001	34116000	1000	1	9.52E-05	-0.49077	4	0.4		
DHR13:42063001	13	42063001	42065000	2000	1	7.08E-05	0.4252311	18	0.9		
DHR13:55564001	13	55564001	55565000	1000	1	5.77E-05	0.4591947	9	0.9	Nek7	Signaling
DHR13:57106001	13	57106001	57107000	1000	1	3.59E-05	0.4716868	4	0.4		
DHR13:59737001	13	59737001	59740000	3000	1	7.96E-05	0.4757132	17	0.567		
DHR13:63799001	13	63799001	63800000	1000	1	5.60E-05	0.4407598	5	0.5	Brinp3	
DHR13:73300001	13	73300001	73303000	3000	1	1.78E-05	-0.545809	34	1.133	Acbd6	Metabolism
DHR13:73426001	13	73426001	73429000	3000	1	1.18E-05	-1.0097885	35	1.167	Qsox1	Metabolism
DHR13:74103001	13	74103001	74105000	2000	1	6.26E-05	-0.4986654	21	1.05		
DHR13:74217001	13	74217001	74218000	1000	1	1.53E-06	-0.6046458	4	0.4	Abl2	Signaling

DHR13:81171001	13	81171001	81172000	1000	1	1.34E-05	0.5098987	8	0.8	Prrx1	Transcription
DHR13:93806001	13	93806001	93807000	1000	1	8.29E-05	-0.4465967	14	1.4	Wdr64	
DHR13:99354001	13	99354001	99355000	1000	1	5.81E-05	-0.5968078	12	1.2	AABR07021930.1	
DHR13:100557001	13	100557001	100558000	1000	1	2.09E-05	-0.5184975	4	0.4	Srp9	Signaling
DHR13:101889001	13	101889001	101891000	2000	1	5.99E-05	-0.4856573	12	0.6		
DHR13:106235001	13	106235001	106236000	1000	1	2.14E-05	0.5268206	4	0.4		
DHR13:110556001	13	110556001	110557000	1000	1	7.72E-05	-0.6957068	13	1.3	AABR07022162.2	
DHR14:15730001	14	15730001	15732000	2000	1	5.91E-05	0.4461452	7	0.35	RGD1561226	EST
DHR14:19601001	14	19601001	19603000	2000	1	2.35E-05	0.4645919	32	1.6		
DHR14:23733001	14	23733001	23734000	1000	1	4.26E-07	0.7831734	4	0.4		
DHR14:27198001	14	27198001	27200000	2000	1	7.54E-05	0.4952549	4	0.2		
DHR14:28777001	14	28777001	28780000	3000	1	5.19E-06	0.5317462	37	1.233	Adgrl3	
DHR14:30237001	14	30237001	30239000	2000	1	8.78E-05	0.3693913	18	0.9		
DHR14:31084001	14	31084001	31085000	1000	1	4.41E-05	0.4465399	2	0.2		
DHR14:31532001	14	31532001	31533000	1000	1	7.87E-05	0.4243219	5	0.5		
DHR14:41150001	14	41150001	41151000	1000	1	6.84E-05	0.4681568	8	0.8		
DHR14:45510001	14	45510001	45512000	2000	1	5.60E-05	0.4551086	17	0.85		
DHR14:46521001	14	46521001	46556000	35000	1	1.25E-05	-0.4634549	1181	3.374	AABR07015055.1;AABR07015057.1;AABR07015055.2;AABR07015056.1	
DHR14:46619001	14	46619001	46627000	8000	1	2.71E-05	-0.4330295	106	1.325	AABR07015076.1	
DHR14:54393001	14	54393001	54394000	1000	1	2.51E-05	0.5056636	2	0.2		
DHR14:62744001	14	62744001	62746000	2000	1	1.18E-06	0.4699353	14	0.7		
DHR14:94799001	14	94799001	94803000	4000	1	5.34E-05	-0.5658147	71	1.775		
DHR14:94873001	14	94873001	94880000	7000	1	1.52E-05	-0.6276372	76	1.086		
DHR14:95303001	14	95303001	95305000	2000	2	1.67E-06	-0.7581728	38	1.9		
DHR14:95337001	14	95337001	95340000	3000	1	8.29E-05	-0.3913601	46	1.533		
DHR14:95529001	14	95529001	95533000	4000	1	3.13E-05	-0.6041799	43	1.075		
DHR14:95556001	14	95556001	95563000	7000	2	8.38E-06	-0.5404642	28	0.4		
DHR14:95747001	14	95747001	95752000	5000	1	8.82E-05	-0.5857555	61	1.22		
DHR14:95757001	14	95757001	95758000	1000	1	2.15E-06	-0.5746308	5	0.5		
DHR14:96275001	14	96275001	96276000	1000	1	8.08E-06	-0.4860703	9	0.9		
DHR14:96543001	14	96543001	96546000	3000	1	1.35E-05	-0.5178531	28	0.933	AABR07016310.2	
DHR14:97029001	14	97029001	97038000	9000	1	1.41E-05	-0.4800422	134	1.489		
DHR14:97041001	14	97041001	97045000	4000	1	9.04E-05	-0.6489034	44	1.1		
DHR14:97291001	14	97291001	97294000	3000	1	1.23E-05	-0.4783968	36	1.2		
DHR14:101126001	14	101126001	101128000	2000	1	1.84E-05	-0.7731014	37	1.85		
DHR14:101319001	14	101319001	101321000	2000	1	8.22E-06	-0.7427514	13	0.65		
DHR14:101347001	14	101347001	101348000	1000	1	1.00E-06	-0.5800859	8	0.8		
DHR14:101557001	14	101557001	101558000	1000	1	6.50E-05	-0.7118065	23	2.3		
DHR14:101706001	14	101706001	101708000	2000	1	7.00E-05	-0.4272256	8	0.4		
DHR14:101966001	14	101966001	101968000	2000	1	1.82E-05	-0.7662069	25	1.25		
DHR14:102169001	14	102169001	102172000	3000	1	2.95E-05	-0.3906128	27	0.9		
DHR14:105501001	14	105501001	105502000	1000	1	9.04E-05	0.470244	2	0.2	AABR07016589.2	
DHR14:107446001	14	107446001	107448000	2000	1	1.48E-06	-0.5737468	43	2.15		
DHR14:111923001	14	111923001	111924000	1000	1	9.15E-05	0.4414435	5	0.5		
DHR14:115244001	14	115244001	115245000	1000	1	3.83E-06	0.530047	6	0.6	Psme4	Proteolysis
DHR15:3097001	15	3097001	3098000	1000	1	3.25E-05	-0.595515	7	0.7	Adk	Signaling
DHR15:3579001	15	3579001	3580000	1000	1	7.19E-05	-0.4192256	17	1.7		
DHR15:4229001	15	4229001	4230000	1000	1	6.74E-05	-0.5035402	6	0.6	Ppp3cb	Signaling
DHR15:12757001	15	12757001	12759000	2000	1	1.64E-05	-0.6278904	36	1.8	AABR07017145.2	
DHR15:16952001	15	16952001	16953000	1000	1	7.33E-05	-0.5358317	4	0.4		
DHR15:19829001	15	19829001	19832000	3000	1	4.95E-05	-0.5125365	43	1.433	Fermt2;U6	
DHR15:21081001	15	21081001	21087000	6000	1	1.95E-05	-0.4094847	78	1.3		
DHR15:21156001	15	21156001	21162000	6000	1	9.13E-05	-0.4340003	72	1.2		
DHR15:21274001	15	21274001	21279000	5000	1	3.80E-05	-0.4625142	26	0.52		
DHR15:21775001	15	21775001	21777000	2000	1	4.38E-05	-0.4377	15	0.75		
DHR15:22114001	15	22114001	22115000	1000	1	1.58E-05	-0.6058247	33	3.3		
DHR15:22522001	15	22522001	22528000	6000	1	6.02E-05	-0.4354511	51	0.85		
DHR15:22675001	15	22675001	22676000	1000	1	6.77E-05	-0.6438794	3	0.3		
DHR15:22693001	15	22693001	22695000	2000	1	8.90E-05	-0.5250068	9	0.45		
DHR15:22745001	15	22745001	22747000	2000	1	6.01E-05	-0.5522025	25	1.25		
DHR15:22798001	15	22798001	22799000	1000	1	4.82E-07	-0.9551251	10	1		
DHR15:22876001	15	22876001	22879000	3000	1	8.97E-05	-0.5776482	23	0.767		
DHR15:22887001	15	22887001	22890000	3000	1	1.24E-05	-0.5701533	41	1.367		
DHR15:23056001	15	23056001	23061000	5000	1	8.31E-05	-0.4408419	41	0.82		
DHR15:32712001	15	32712001	32713000	1000	1	1.96E-05	-0.5698142	5	0.5	AABR07017902.1	
DHR15:34428001	15	34428001	34430000	2000	1	1.11E-07	-0.6405287	21	1.05	Dhrs1;Nop9;Cideb	Metabolism;Unknown
DHR15:34492001	15	34492001	34495000	3000	1	5.16E-05	-0.9844773	119	3.967	Nfatc4	Transcription
DHR15:39447001	15	39447001	39448000	1000	1	7.40E-05	-0.5637189	2	0.2		

DHR15:39710001	15	39710001	39711000	1000	1	3.07E-06	-0.54753	8	0.8	Setdb2	Transcription
DHR15:40307001	15	40307001	40309000	2000	1	6.73E-05	-0.4486295	21	1.05	Atp8a2	Transport
DHR15:40856001	15	40856001	40857000	1000	1	9.76E-05	-0.472292	19	1.9		
DHR15:45991001	15	45991001	45992000	1000	1	2.62E-05	-0.5939258	12	1.2		
DHR15:47794001	15	47794001	47795000	1000	1	5.79E-05	-0.5824786	11	1.1	Msra	Metabolism
DHR15:59197001	15	59197001	59198000	1000	1	4.76E-05	0.4737984	4	0.4		
DHR15:64403001	15	64403001	64404000	1000	1	6.26E-05	0.4253744	8	0.8		
DHR15:67588001	15	67588001	67589000	1000	1	8.49E-05	0.4141638	10	1	Pcdh17	Cytoskeleton
DHR15:72086001	15	72086001	72087000	1000	1	7.84E-05	0.4549044	3	0.3	AABR07018764.1	
DHR15:73390001	15	73390001	73391000	1000	1	6.14E-07	0.5690872	4	0.4		
DHR15:79706001	15	79706001	79708000	2000	1	4.09E-05	0.474346	9	0.45		
DHR15:86070001	15	86070001	86071000	1000	1	9.41E-05	0.5667427	9	0.9	Tbc1d4	
DHR15:87076001	15	87076001	87078000	2000	1	1.22E-05	0.505868	10	0.5		
DHR15:90762001	15	90762001	90764000	2000	1	3.92E-05	0.4466914	27	1.35	Mycbp2	Metabolism
DHR15:91491001	15	91491001	91492000	1000	1	3.05E-05	0.4671429	14	1.4	Mycbp2;AABR07019155.1	Metabolism
DHR15:93332001	15	93332001	93336000	4000	1	5.75E-05	0.3990148	34	0.85	Mycbp2	Metabolism
DHR15:93420001	15	93420001	93422000	2000	1	4.31E-05	0.4430962	5	0.25	Mycbp2	Metabolism
DHR15:97358001	15	97358001	97360000	2000	1	7.25E-05	0.5068684	6	0.3		
DHR15:101626001	15	101626001	101628000	2000	1	5.94E-05	0.4174541	17	0.85		
DHR15:101978001	15	101978001	101982000	4000	1	9.19E-06	0.4579006	28	0.7		
DHR15:103055001	15	103055001	103056000	1000	1	5.32E-05	0.4960281	9	0.9	Gpc6	Extracellular Matrix
DHR15:105433001	15	105433001	105434000	1000	1	3.77E-05	0.6963617	15	1.5		
DHR15:105744001	15	105744001	105745000	1000	1	2.24E-05	0.6357999	41	4.1	Mbnl2	Transcription
DHR15:106898001	15	106898001	106899000	1000	1	7.47E-05	-0.5587027	12	1.2		
DHR15:106936001	15	106936001	106939000	3000	1	4.30E-06	-0.6619385	19	0.633		
DHR16:543001	16	543001	549000	6000	1	2.65E-05	-0.4839605	40	0.667	AABR07024473.2;AABR07024468.1	
DHR16:568001	16	568001	573000	5000	1	4.97E-05	-0.4057317	43	0.86	AABR07024473.2;AABR07024468.1	
DHR16:574001	16	574001	577000	3000	1	8.71E-05	-0.4435458	25	0.833	AABR07024473.2;AABR07024468.1	
DHR16:641001	16	641001	648000	7000	2	1.36E-06	-0.5120154	58	0.829		
DHR16:650001	16	650001	669000	19000	5	4.36E-07	-0.4565384	114	0.6		
DHR16:1865001	16	1865001	1866000	1000	1	6.15E-05	-0.8127797	10	1	Zmiz1	Metabolism
DHR16:7594001	16	7594001	7596000	2000	1	5.16E-05	-0.5088479	35	1.75	Mettl6;Eaf1	Metabolism;Apoptosis
DHR16:20599001	16	20599001	20603000	4000	1	2.80E-05	-0.4607272	69	1.725	Ssbp4;Isyna1;Ell	Translation;Metabolism
DHR16:24894001	16	24894001	24895000	1000	1	5.83E-05	0.5447131	3	0.3		
DHR16:28764001	16	28764001	28765000	1000	1	7.40E-05	0.3941535	7	0.7		
DHR16:30033001	16	30033001	30036000	3000	1	5.54E-05	-0.569818	35	1.167		
DHR16:30193001	16	30193001	30200000	7000	1	6.38E-05	-0.5614167	98	1.4		
DHR16:30306001	16	30306001	30310000	4000	1	5.89E-05	-0.4882065	38	0.95		
DHR16:30607001	16	30607001	30609000	2000	1	2.03E-05	-0.4701435	19	0.95		
DHR16:32790001	16	32790001	32791000	1000	1	5.85E-05	0.3905343	10	1		
DHR16:45428001	16	45428001	45430000	2000	1	3.59E-05	0.4200871	25	1.25		
DHR16:51615001	16	51615001	51617000	2000	1	3.58E-05	0.4512871	12	0.6		
DHR16:54378001	16	54378001	54380000	2000	1	4.73E-05	-0.4917905	30	1.5	Mtus1;Pdgfrl;AABR07025896.1	Receptor
DHR16:55421001	16	55421001	55423000	2000	1	1.60E-05	0.4537393	4	0.2		
DHR16:58263001	16	58263001	58264000	1000	1	7.90E-05	0.4824112	8	0.8		
DHR16:64288001	16	64288001	64289000	1000	1	5.77E-05	0.448284	6	0.6		
DHR16:67157001	16	67157001	67159000	2000	1	1.50E-05	0.488523	17	0.85		
DHR16:71350001	16	71350001	71352000	2000	1	2.05E-06	-1.2979331	33	1.65		
DHR16:88036001	16	88036001	88037000	1000	1	2.82E-05	0.5443805	6	0.6		
DHR16:88225001	16	88225001	88227000	2000	1	9.61E-05	0.506743	6	0.3		
DHR17:5736001	17	5736001	5738000	2000	1	7.48E-05	-0.5594227	40	2		
DHR17:6076001	17	6076001	6077000	1000	1	8.32E-07	-0.5577953	25	2.5	Ntrk2	Receptor
DHR17:8907001	17	8907001	8909000	2000	1	4.42E-05	-0.6308315	34	1.7	Catsper3	Metabolism
DHR17:11818001	17	11818001	11820000	2000	1	8.29E-06	-0.5679151	18	0.9		
DHR17:23941001	17	23941001	23943000	2000	1	2.22E-05	-0.506965	27	1.35		
DHR17:30308001	17	30308001	30311000	3000	1	6.74E-05	-0.9116695	45	1.5		
DHR17:33485001	17	33485001	33486000	1000	1	1.67E-05	0.5282707	4	0.4	Gmds	Metabolism
DHR17:42857001	17	42857001	42861000	4000	2	1.32E-05	0.840564	16	0.4	Prl3d2	Hormone
DHR17:52481001	17	52481001	52484000	3000	1	7.61E-05	-0.4718694	36	1.2	Gli3	Transcription
DHR17:53754001	17	53754001	53756000	2000	1	7.32E-05	-0.4902126	33	1.65	Hecw1	Protease
DHR17:57678001	17	57678001	57680000	2000	1	2.89E-05	0.9034668	22	1.1	RGD1564347	Metabolism
DHR17:62279001	17	62279001	62280000	1000	1	3.86E-05	-0.5825984	7	0.7		
DHR17:69924001	17	69924001	69925000	1000	1	1.92E-05	0.4137454	5	0.5		
DHR17:75184001	17	75184001	75186000	2000	1	8.31E-06	0.4642887	15	0.75	AABR07028568.1	
DHR17:76245001	17	76245001	76246000	1000	1	1.58E-05	-0.6339486	15	1.5	Upf2	Transcription
DHR17:80236001	17	80236001	80237000	1000	1	9.56E-05	0.4590845	10	1		
DHR17:80865001	17	80865001	80866000	1000	1	6.13E-05	0.4235281	11	1.1	Trdmt1	Transcription
DHR17:88141001	17	88141001	88142000	1000	1	9.25E-05	-0.5933134	10	1	AABR07028839.1	
DHR18:5245001	18	5245001	5247000	2000	1	8.45E-05	-0.5967681	41	2.05	Zfp521	

DHR18:8528001	18	8528001	8529000	1000	1	8.36E-05	0.4351465	3	0.3		
DHR18:15550001	18	15550001	15551000	1000	1	9.90E-05	0.4748125	10	1	Ttr;AABR07031489.1	Receptor
DHR18:21537001	18	21537001	21539000	2000	1	1.04E-06	0.5400239	11	0.55		
DHR18:40656001	18	40656001	40658000	2000	1	7.54E-05	0.2858047	15	0.75		
DHR18:50621001	18	50621001	50622000	1000	1	1.82E-05	0.4689113	9	0.9		
DHR18:76484001	18	76484001	76487000	3000	1	8.51E-05	-0.8865815	92	3.067		
DHR18:84794001	18	84794001	84796000	2000	1	4.73E-05	0.4468308	17	0.85		
DHR18:87282001	18	87282001	87283000	1000	1	3.79E-05	0.4862753	3	0.3		
DHR19:1602001	19	1602001	1603000	1000	1	4.15E-05	0.4209654	9	0.9	LOC102556295	
DHR19:4792001	19	4792001	4795000	3000	1	7.81E-05	0.4267148	13	0.433		
DHR19:5299001	19	5299001	5301000	2000	1	7.22E-06	0.4658809	6	0.3		
DHR19:5818001	19	5818001	5825000	7000	1	5.61E-05	-0.7553435	128	1.829		
DHR19:6161001	19	6161001	6162000	1000	1	2.07E-05	0.4604377	6	0.6	Cdh8	Extracellular Matrix
DHR19:7280001	19	7280001	7282000	2000	1	9.96E-05	0.5109114	16	0.8		
DHR19:20554001	19	20554001	20555000	1000	1	9.93E-05	-0.4859733	7	0.7	AABR07043098.1	
DHR19:21249001	19	21249001	21251000	2000	1	7.75E-05	-0.6161844	25	1.25	N4bp1	Receptor
DHR19:24758001	19	24758001	24762000	4000	1	9.05E-05	-0.6579094	66	1.65	Dnajb1	Transcription
DHR19:26372001	19	26372001	26373000	1000	1	6.23E-05	-0.8026614	4	0.4		
DHR19:29209001	19	29209001	29210000	1000	1	7.97E-05	-0.6151003	4	0.4		
DHR19:33609001	19	33609001	33610000	1000	1	7.18E-06	-0.6921809	6	0.6		
DHR19:36854001	19	36854001	36855000	1000	1	8.27E-05	-0.4526758	8	0.8		
DHR19:39037001	19	39037001	39038000	1000	1	5.62E-06	-0.5358303	9	0.9	Tango6	
DHR19:41160001	19	41160001	41161000	1000	1	8.24E-05	-0.555345	17	1.7	Hydin	Unknown
DHR19:42305001	19	42305001	42306000	1000	1	8.30E-05	-0.5102768	15	1.5	5S_rRNA	
DHR19:50419001	19	50419001	50420000	1000	1	2.81E-05	-1.1015196	9	0.9		
DHR19:51929001	19	51929001	51932000	3000	1	8.93E-05	-0.5657233	36	1.2	Cdh13;AABR07044001.1	Extracellular Matrix
DHR19:55329001	19	55329001	55330000	1000	1	7.61E-05	-0.9083826	30	3	Piezo1	
DHR19:55761001	19	55761001	55762000	1000	1	3.22E-05	-0.5070783	14	1.4		
DHR20:132001	20	132001	138000	6000	1	7.83E-06	-0.382309	90	1.5		
DHR20:2647001	20	2647001	2651000	4000	1	7.90E-05	-0.5669647	61	1.525	AABR07044322.1;C4a	Immune
DHR20:3140001	20	3140001	3165000	25000	16	6.46E-09	-0.9779821	239	0.956	AABR07044362.6;AABR07044362.1;AABR07044362.3;RT1-N2;RT1-S2;AABR07044362.5;AABR07044362.2;RT1-N3;AABR07044362.4	Immune
DHR20:4662001	20	4662001	4681000	19000	1	4.00E-05	-0.3188671	330	1.737	RT1-CE1;AABR07044397.1;RT1-CE4;AABR07044404.1	Immune
DHR20:4800001	20	4800001	4803000	3000	1	4.26E-05	-0.3950519	38	1.267	RT1-CE1;RT1-CE4;AABR07044404.1;RT1-CE7;Ddx39b;AABR07044405.1;SNORD83	Immune
DHR20:4894001	20	4894001	4936000	42000	2	8.50E-06	-0.4504208	764	1.819	RT1-CE1;RT1-CE4;RT1-CE7;AABR07044408.1;RT1-CE5;AABR07044408.3;AABR07044408.2;RT1-CE3	Immune
DHR20:5366001	20	5366001	5381000	15000	1	4.22E-05	-0.4929526	241	1.607	RT1-A1;RT1-A2	Immune
DHR20:5407001	20	5407001	5423000	16000	1	7.39E-05	-0.5372567	303	1.894	RT1-A1;Vps52	Immune;Transport
DHR20:9703001	20	9703001	9706000	3000	1	2.86E-05	-0.5938015	68	2.267	Umodl1	
DHR20:11662001	20	11662001	11664000	2000	1	5.11E-05	-0.4997894	16	0.8	LOC690478	EST
DHR20:12016001	20	12016001	12018000	2000	1	8.96E-05	-0.5004431	22	1.1	Adarb1	Epigenetic
DHR20:18215001	20	18215001	18216000	1000	1	4.17E-06	-1.1140906	24	2.4		
DHR20:18423001	20	18423001	18424000	1000	1	8.07E-05	-0.6022827	22	2.2		
DHR20:19024001	20	19024001	19025000	1000	1	4.95E-05	-0.9439858	6	0.6		
DHR20:30366001	20	30366001	30370000	4000	1	5.86E-05	-0.8768017	81	2.025	Unc5b	Receptor
DHR20:54662001	20	54662001	54663000	1000	1	2.44E-05	0.4854957	6	0.6		
DHRX:409001	X	409001	410000	1000	1	5.00E-05	-0.7799524	24	2.4		
DHRX:2582001	X	2582001	2583000	1000	1	2.49E-05	-0.6977669	3	0.3	Slc9a7	Metabolism
DHRX:3291001	X	3291001	3293000	2000	1	9.06E-05	-0.7043606	17	0.85		
DHRX:3960001	X	3960001	3962000	2000	1	5.41E-05	-0.7240409	6	0.3		
DHRX:5590001	X	5590001	5592000	2000	1	3.78E-07	-0.7667474	19	0.95		
DHRX:7100001	X	7100001	7101000	1000	1	8.03E-05	-0.8662789	2	0.2		
DHRX:7143001	X	7143001	7146000	3000	1	8.35E-06	-0.8711457	22	0.733		
DHRX:9325001	X	9325001	9327000	2000	1	2.76E-05	-0.7427343	15	0.75		
DHRX:11515001	X	11515001	11516000	1000	1	2.44E-06	-0.9099343	14	1.4		
DHRX:11809001	X	11809001	11810000	1000	1	1.02E-05	-0.8137696	8	0.8		
DHRX:12043001	X	12043001	12044000	1000	1	1.26E-05	-0.6834855	11	1.1		
DHRX:12678001	X	12678001	12680000	2000	1	1.74E-05	-0.7818344	26	1.3		
DHRX:17033001	X	17033001	17034000	1000	1	1.90E-05	-0.7300822	8	0.8	Bmp15	Growth Factors & Cytokines
DHRX:17146001	X	17146001	17147000	1000	1	2.64E-05	-0.4956809	4	0.4		

DHRX:19678001	X	19678001	19680000	2000	1	6.70E-05	-0.691324	10	0.5		
DHRX:26459001	X	26459001	26461000	2000	1	8.04E-05	-0.8279015	9	0.45	Amelx;AABR07037645.1	Extracellular Matrix
DHRX:26728001	X	26728001	26729000	1000	1	4.04E-06	-0.7584762	7	0.7		
DHRX:29046001	X	29046001	29047000	1000	1	2.49E-06	-0.9234529	5	0.5		
DHRX:32909001	X	32909001	32914000	5000	2	5.92E-07	-1.5964717	31	0.62		
DHRX:33537001	X	33537001	33538000	1000	1	6.70E-05	-0.7032024	3	0.3	Syap1	
DHRX:33863001	X	33863001	33864000	1000	1	2.07E-05	-0.7734254	7	0.7		
DHRX:35649001	X	35649001	35651000	2000	1	2.28E-05	-0.9243424	23	1.15	Cdkl5	Signaling
DHRX:36085001	X	36085001	36087000	2000	1	1.50E-06	-0.7643459	21	1.05		
DHRX:43970001	X	43970001	43971000	1000	1	7.31E-05	-0.6851447	3	0.3		
DHRX:46238001	X	46238001	46240000	2000	1	8.90E-06	-0.8601831	8	0.4		
DHRX:48667001	X	48667001	48668000	1000	1	5.85E-05	-0.6874541	5	0.5		
DHRX:48881001	X	48881001	48882000	1000	1	9.23E-05	-0.6720393	1	0.1		
DHRX:51020001	X	51020001	51021000	1000	1	9.62E-05	-0.7305884	5	0.5		
DHRX:54206001	X	54206001	54207000	1000	1	3.34E-05	-0.8530365	8	0.8		
DHRX:61979001	X	61979001	61980000	1000	1	5.60E-05	-0.5812179	3	0.3		
DHRX:65801001	X	65801001	65802000	1000	1	4.23E-05	-0.6829067	1	0.1	Gpr165;AABR07039000.1	Signaling
DHRX:69028001	X	69028001	69032000	4000	1	6.51E-05	-0.7574415	40	1	AABR07039133.3	
DHRX:72078001	X	72078001	72079000	1000	1	3.78E-05	-0.9464603	42	4.2	Rps4x;AABR07039214.2	Translation
DHRX:72668001	X	72668001	72671000	3000	1	7.03E-05	-0.7675731	49	1.633	AABR07039229.1	
DHRX:72678001	X	72678001	72679000	1000	1	2.58E-05	-0.5173752	11	1.1	AABR07039229.1;Dmrtc1c1	
DHRX:75662001	X	75662001	75663000	1000	1	9.27E-05	-0.7011358	6	0.6		
DHRX:79739001	X	79739001	79740000	1000	1	4.28E-05	-0.7234565	8	0.8	LOC103690878	
DHRX:85129001	X	85129001	85130000	1000	1	2.04E-05	-0.6881814	3	0.3	AABR07039799.1	
DHRX:86547001	X	86547001	86548000	1000	1	2.90E-07	-0.8218582	4	0.4		
DHRX:93653001	X	93653001	93656000	3000	1	6.12E-05	-0.5998787	38	1.267		
DHRX:94868001	X	94868001	94869000	1000	1	7.34E-05	-0.742361	3	0.3		
DHRX:95042001	X	95042001	95043000	1000	1	1.65E-05	-0.7914831	4	0.4		
DHRX:95520001	X	95520001	95521000	1000	1	7.86E-05	-0.7179737	4	0.4		
DHRX:99317001	X	99317001	99322000	5000	1	5.75E-05	-0.6602065	11	0.22		
DHRX:102393001	X	102393001	102394000	1000	1	6.28E-06	-0.7674057	7	0.7		
DHRX:103984001	X	103984001	103985000	1000	1	9.64E-05	-0.5646001	28	2.8		
DHRX:104376001	X	104376001	104380000	4000	1	1.43E-05	-0.792894	22	0.55		
DHRX:112793001	X	112793001	112795000	2000	1	3.02E-05	-0.6508275	21	1.05	Col4a5	
DHRX:117807001	X	117807001	117808000	1000	1	9.32E-06	-0.7912844	2	0.2		
DHRX:119996001	X	119996001	119997000	1000	1	6.17E-05	-0.8981733	3	0.3		
DHRX:120742001	X	120742001	120743000	1000	1	2.33E-05	-0.7000908	3	0.3		
DHRX:123140001	X	123140001	123141000	1000	1	8.90E-05	-0.6703509	8	0.8		
DHRX:124039001	X	124039001	124041000	2000	1	4.34E-05	-0.4784763	12	0.6	LOC691272;AABR07041246.1	
DHRX:126839001	X	126839001	126840000	1000	1	6.78E-05	-0.4640379	7	0.7		
DHRX:127740001	X	127740001	127741000	1000	1	6.27E-05	-0.7643886	3	0.3	Gria3	Signaling
DHRX:131695001	X	131695001	131696000	1000	1	7.86E-05	-0.7580742	6	0.6		
DHRX:131856001	X	131856001	131857000	1000	1	4.80E-05	-0.683287	4	0.4		
DHRX:133645001	X	133645001	133646000	1000	1	3.77E-05	-0.8055269	1	0.1		
DHRX:136395001	X	136395001	136396000	1000	1	7.98E-05	-0.7778358	15	1.5		
DHRX:136901001	X	136901001	136902000	1000	1	1.82E-05	-0.7236842	4	0.4	Olr1766	
DHRX:140740001	X	140740001	140742000	2000	1	2.47E-05	-0.8685691	18	0.9		
DHRX:142170001	X	142170001	142172000	2000	1	2.47E-05	-0.6764609	15	0.75	Fgf13	Growth Factors & Cytokines
DHRX:142932001	X	142932001	142933000	1000	1	6.02E-06	-0.7092303	8	0.8		
DHRX:151328001	X	151328001	151330000	2000	1	1.04E-05	-0.8847983	23	1.15		
DHRX:156695001	X	156695001	156696000	1000	1	5.87E-06	-0.7591137	5	0.5	Mecp2	Transcription
DHRX:157164001	X	157164001	157167000	3000	1	7.96E-05	-0.6738611	12	0.4	Pnck;Dusp9	Signaling
DHRX:157618001	X	157618001	157619000	1000	1	4.22E-06	-0.7314174	6	0.6	AABR07042471.1	
DHRX:158890001	X	158890001	158891000	1000	1	7.64E-05	-0.7401845	3	0.3	Ints6l	
